# Frequent Plastic Usage Behavior and Lack of Microplastic Awareness Correlates with Cognitive Decline: A Cross-Sectional Survey

**DOI:** 10.3390/ijerph23010067

**Published:** 2026-01-01

**Authors:** Pukovisa Prawiroharjo, Anyelir Nielya Mutiara Putri, Noryanto Ikhromi, Aldithya Fakhri, Elizabeth Divina, Rani Permata, Aileen Gabrielle, Violine Martalia, Agustyno Zulys

**Affiliations:** 1 Department of Neurology, Faculty of Medicine, Universitas Indonesia, Cipto Mangunkusumo National Central Hospital, Jakarta 10430, Indonesia; 2 Department of Neurology, Universitas Indonesia Hospital, Depok 16424, Indonesia; 3 Brainz Research and Innovation Center, Jakarta 12310, Indonesiaranipermata99@gmail.com (R.P.);; 4 Faculty of Medicine, Universitas Indonesia, Jakarta 10430, Indonesia; 5 Department of Chemistry, Faculty of Mathematics and Science, Universitas Indonesia, Depok 16424, Indonesia

**Keywords:** microplastics, plastic consumption, cognitive function, awareness, risk perception, Jakarta

## Abstract

**Highlights:**

**Public health relevance—How does this work relate to a public health issue?**

- Microplastics are persistent environmental contaminants that enter the human body primarily through ingestion, posing potential neurotoxic risks via oxidative stress and inflammation mechanisms.
- This study investigates the emerging intersection of environmental health and neurology by examining how daily plastic usage behaviors correlate with neurocognitive function in an adult population.

**Public health significance—Why is this work of significance to public health?**

- Multivariate analysis indicates that high consumption of single-use plastics is significantly associated with a 41% increase regarding the risk of suspected cognitive impairment (OR = 1.409).
- The study identifies a critical gap in health literacy, where low awareness of microplastics correlates with higher consumption of single-use plastics, thus potentially increasing exposure to neurotoxic contaminants.

**Public health implications—What are the key implications or messages for practitioners, policy makers and/or researchers in public health?**

- Public health strategies must integrate environmental sustainability with neurological well-being, emphasizing reducing the consumption of single-use plastics as a preventative measure for cognitive health.
- Effective interventions require structural policy changes, such as phasing out virgin plastics and enforcing industry transparency, rather than relying solely on individual awareness, which was not found to be an independent predictor of cognitive status.

**Abstract:**

Introduction: Microplastics (MPs) are widespread environmental pollutants with possible neurotoxic effects. Exploring links between plastic use, MP awareness, and cognition is key for assessing public health risks. Objective: To examine correlations between plastic consumption, MP awareness and attitudes, and neurocognitive function among Greater Jakarta residents. Methods: A cross-sectional survey of 562 adults used a validated e-questionnaire covering plastic usage, MP knowledge, attitudes, risk perception, and cognition via the Ascertain Dementia 8 (AD-8). Analyses included chi-square, *t*-tests, and logistic regression. Results: Suspected cognitive impairment (AD-8 ≥ 2) was observed in 44.5% of respondents. High single-use plastic consumption correlated with worse cognition (*p* = 0.032), while reusable plastic use showed no association (*p* = 0.605). Awareness of MPs was relatively low, with 19.4% of respondents having never heard of them. Awareness and knowledge also varied significantly by age (*p* = 0.007), gender (*p* = 0.004), and education level (*p* = 0.027). Positive attitudes and higher risk perception aligned with greater awareness (*p* < 0.001) but not cognitive scores. Risk perception influenced bottled water use (*p* = 0.009), with low-risk groups consuming more. Conclusions: Frequent single-use plastic consumption is linked to poorer cognition, while MP awareness and risk perception do not directly affect cognitive outcomes. Educational strategies may enhance awareness and reduce exposure.

## 1. Introduction

Microplastics (MPs) represent a class of persistent environmental contaminants derived either from the progressive fragmentation of macroplastic debris through physicochemical and biological degradation processes or from direct environmental release of manufactured microplastic particles [1]. Global plastic production has exhibited consistent annual growth, escalating from 250 million metric tons in 2009 to 335 million metric tons by 2016 [2]. Current worldwide estimates indicate that in 2015, approximately 55% of post-consumer plastic waste was improperly disposed into natural environments, with only 25% undergoing controlled incineration and 20% entering recycling streams [3]. The environmental persistence of MPs in marine ecosystems demonstrates considerable variability in degradation kinetics, where reduced degradation rates enhance both their environmental residence time and bioavailability to aquatic organisms across multiple trophic levels, including teleost fish, crustaceans, chelonians, and zooplankton [4,5]. This trophic transfer culminates in bioaccumulation within higher organisms, including humans, with potential implications for public health. Furthermore, empirical evidence demonstrates that MPs function as transport vectors for both heavy metals and hydrophobic organic contaminants, facilitating their mobilization within ecosystems and subsequent bioaccumulation in biota [6]. Human exposure pathways extend beyond dietary ingestion to include transdermal absorption and pulmonary intake of airborne particulate matter [7].

Analytical studies have documented MP contamination across diverse consumable products, including culinary salts, potable water, alcoholic beverages, and various agricultural and marine foodstuffs [8,9]. Preliminary investigations in Indonesia have detected MP contamination in both natural and artificial water reservoirs, including commercially bottled water, underscoring the ubiquity of anthropogenic particulate contamination in modern environments. This pervasive environmental presence raises significant public health concerns, particularly given the current absence of standardized assessment tools for evaluating plastic consumption patterns within the Indonesian population. The gastrointestinal system represents the primary target organ for MP toxicity due to its role as the major portal of entry. Experimental animal models have demonstrated that MP exposure induces neurotoxic effects, manifested through elevated oxidative stress markers in cerebral tissues, disruption of neurotransmitter homeostasis, and increased susceptibility to neurodegenerative pathologies, peripheral nervous system dysfunction, and behavioral modifications [10,11,12].

## 2. Materials and Methods

### 2.1. Study Design

This research employed a cross-sectional observational design using an electronic questionnaire to assess patterns of plastic usage, awareness of microplastics, and associated neurocognitive complaints. The study was conducted in the Greater Jakarta area (Jabodetabek) and utilized online data collection methods to enable broad participation while ensuring accessibility and standardization. The online format allowed rapid distribution and response gathering during the data collection period in early 2025.

### 2.2. Participants

The target population of this study was defined as all Indonesian residents potentially exposed to microplastics via oral ingestion, while the accessible population was limited to residents in the Greater Jakarta region. The research subjects were individuals from the accessible population who met the study’s inclusion and exclusion criteria. Participants were recruited through convenience sampling using digital platforms, such as social media and community groups. A total of 562 participants completed the full questionnaire and were included in the analysis.

Inclusion criteria included: (1) aged 18 years or older; (2) current residency in the Greater Jakarta area; (3) ability to understand and respond in Bahasa Indonesia; and (4) willingness to participate, confirmed through digital informed consent. Exclusion criteria included: (1) incomplete responses, and (2) self-reported history of major neurocognitive disorders, such as dementia or traumatic brain injury.

### 2.3. Data Collection

Data were collected through a structured electronic questionnaire designed in Bahasa Indonesia and distributed via an online survey platform. The instrument comprised five main components: (1) sociodemographic characteristics (e.g., age, sex, education level, income); (2) patterns of plastic usage, including frequency and types of plastic consumed in daily life; (3) awareness and perceptions regarding microplastics, including perceived health risks; (4) self-reported health complaints that may be associated with microplastic exposure; and (5) a brief self-assessment of neurocognitive function adapted from a validated screening tool appropriate for general populations.

The questionnaire underwent content and previously validated by public health and neurology experts. It demonstrated strong validity for the knowledge (r = 0.379, *p* = 0.039), behavior (r = 0.726, *p* < 0.001), and attitude domains (r = 0.385, *p* = 0.036), with acceptable internal consistency (α = 0.623) [13]. The questionnaire required approximately 10–15 min to complete, and built-in validation settings prevented submission of incomplete forms. All responses were stored securely in password-protected and encrypted databases accessible only to the research team. We will attach the evidence of the collected survey responses, excluding respondents’ confidential information, in the Supplementary Dataset S1.

### 2.4. Statistical Analysis

Descriptive statistics were used to summarize sociodemographic characteristics, plastic usage patterns, awareness levels, and health complaints. Continuous variables were presented as means and standard deviations, while categorical variables were reported as frequencies and percentages. Associations between plastic consumption or awareness and neurocognitive complaints were assessed using chi-square tests or independent *t*-tests, depending on the nature of the variables. Multivariate analysis was performed to identify independent predictors of neurocognitive complaints, adjusting for potential confounders such as age, education, and income. All analyses were conducted using SPSS version 26, with a significance level set at *p* < 0.05.

### 2.5. Ethical Considerations

This study received ethical approval from the Health Research Ethics Committee of Faculty of Medicine Universitas Indonesia and was conducted in accordance with the Declaration of Helsinki. All participants provided informed consent through the electronic platform before accessing the questionnaire. Anonymity and confidentiality were strictly maintained throughout the study process. Data were stored securely and used solely for research purposes, with no identifying information collected that could be traced back to individual participants.

## 3. Results

### 3.1. Demographic Characteristics

This survey involved 562 respondents from Jakarta (Table 1), with the proportion of female to male respondents being 62.46% (*n* = 351) and 37.54% (*n* = 211), respectively. Most respondents were young (median [min–max]: 29 [19–69] years), with 58.72% of participants (*n* = 330) aged 30 years or below. To facilitate analysis, educational levels were categorized as low (high school or lower), medium (diploma/D3 and bachelor’s degree/D4/S1), and high (master’s degree/S2 or doctoral degree/S3), with proportions of 26.51% (*n* = 149), 62.81% (*n* = 353), and 10.68% (*n* = 60), respectively. The sample in this study was predominantly from the young age group and medium education level (*n* = 234, 41.63%); this should be considered when interpreting the results due to potential bias. Some respondents reported a history of chronic diseases, including hypertension (*n* = 8), diabetes mellitus (*n* = 4), and HIV (*n* = 2).

### 3.2. Cognitive Function of Subjects

Cognitive function was assessed using the Ascertain Dementia 8 (AD-8), a simple questionnaire designed to screen for cognitive impairment. It was administered via self-report and adapted for electronic administration. The AD-8 consists of eight questions covering domains such as decision-making, activity levels, conversational repetition, learning ability, memory function, financial management, and daily thinking processes. The AD-8 has been validated for use as a self-administered cognitive impairment (CI) screening tool with a cut-off score of 2 out of 8 was applied to classify mild cognitive impairment (MCI), dementia, and overall cognitive impairment (MCI or dementia). In individuals with a score ≥ 2, cognitive impairment is suspected, and further examination is required to confirm it. The participant-administered (self-reported) AD-8 demonstrated 57% sensitivity and 71% specificity for detecting MCI, and 82% sensitivity and 75% specificity for detecting dementia. In this study, 312 participants (55.52%) scored below 2 on the AD-8, while the remaining 250 (44.48%) scored 2 or higher [14].

### 3.3. Plastic Usage Behavior

**Figure 1:**
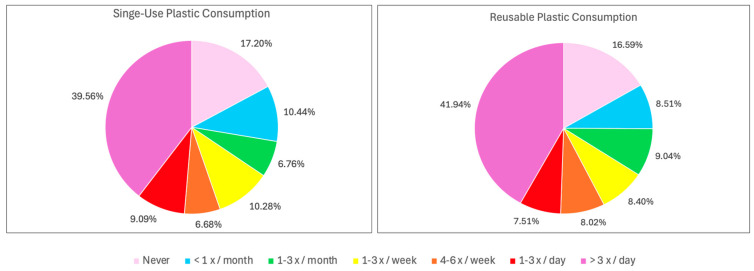
Consumption patterns of single use plastics and reusable plastics.

Plastic use behavior was assessed based on participants’ habits in using single-use and reusable plastics. For analytical purposes, plastic use behavior was categorized into three levels: low (never, <1 x/month), moderate (1–3 x/month, 1–3 x/week, 4–6 x/week), and high (1–3 x/day and >3 x/day). Analysis of participants’ plastic usage revealed clear behavioral trends for both single-use and reusable plastics, as shown in Figure 1.

The analysis revealed distinct demographic patterns in plastic consumption behaviors. Education level emerged as a significant factor associated with single-use plastic consumption (*p* = 0.001). No significant associations were found between reusable plastic consumption and demographic variables such as gender, age, or education level (Table 2).

### 3.4. Microplastics Awareness and Knowledge

A total of 19.40% of respondents (n = 109) had never heard of microplastics, and 32.74% (n = 184) had never sought information about them. Detailed responses regarding microplastics awareness and knowledge are presented in Supplementary Table S1. Table 3 presents the distribution of awareness and knowledge toward microplastics across different demographic characteristics, analyzed using the Chi-square test. Age shows a significant association with awareness and knowledge (*p* = 0.007), where respondents aged ≤30 years dominate the “good” category (63.89%), while those aged ≥50 years are more prevalent in the “bad” category (15.82%). Gender is also significantly related (*p* = 0.004); males account for most respondents with “bad” knowledge (71.9%), whereas females are more evenly distributed, with 46.11% having “good” knowledge. Education level further demonstrates a significant relationship (*p* = 0.027), with higher education (75.0%) being strongly associated with better awareness and knowledge compared to lower education (25.0%), where 34.5% fall into the “bad” category. These findings highlight that younger age, female gender, and higher education level are correlated with better awareness and knowledge about microplastics.

### 3.5. Attitudes Toward Microplastics

Respondents’ attitudes toward microplastics were evaluated through multiple questions in Supplementary Table S2, with cutoff scores of >60 indicating good, 40–60 indicating intermediate, and <40 indicating poor attitudes [13]. Table 4 presents the attitudes towards microplastics based on demographic characteristics, including age, gender, and education level. The majority of respondents with good attitudes towards microplastics were aged ≤30 years (57.56%), followed by those aged 31–49 years (34.68%) and ≥50 years (7.74%). Age showed a significant association with attitudes (*p* = 0.037), indicating younger respondents were more likely to have positive attitudes. In terms of gender, males (59.77%) demonstrated a higher proportion of good attitudes compared to females (39.85%), but the association was not statistically significant (*p* = 0.360). Education level showed a strong and significant association with attitudes (*p* < 0.001), with respondents having higher education levels accounting for 67.52% of those with good attitudes, compared to only 32.47% among those with lower education levels. These findings suggest that age and education significantly influence attitudes towards microplastics, whereas gender does not.

### 3.6. Risk Perception of Microplastics

Risk perception was assessed based on multiple items in Supplementary Table S3. Table 5 describes the risk perception of microplastics based on age, gender, and education level. Among respondents with a high perception of microplastic risk (n = 160), the majority were aged ≤ 30 years (56.87%), followed by those aged 31–49 years (36.87%), and ≥50 years (6.25%). However, age was not significantly associated with risk perception (*p* = 0.344). Gender distribution showed that 30.20% of females and 25.59% of males had high risk perception, but this difference was not statistically significant (*p* = 0.289). Education level showed that respondents with higher education accounted for the majority (79.37%) of those with high-risk perception, compared to only 20.62% from the lower education group, though this association was also not significant (*p* = 0.184). Overall, none of the demographic factors (age, gender, education) showed a statistically significant relationship with risk perception of microplastics.

### 3.7. Correlation of Awareness, Attitude, and Risk Perception with Plastic Usage Pattern

Table 6 presents the association between plastic usage patterns and three psychosocial variables: awareness and knowledge, attitude, and risk perception related to microplastics. Statistical analysis using the Chi-square test revealed a significant relationship between the use of reusable plastic and awareness and knowledge (*p* = 0.044), indicating that individuals who use reusable plastics tend to have higher awareness and knowledge about microplastics. However, no significant associations were found between plastic usage patterns and attitude (*p* = 0.561 for single-use; *p* = 0.768 for reusable) or risk perception (*p* = 0.576 for single-use; *p* = 0.329 for reusable). These findings suggest that while awareness and knowledge may influence behavioral choices like using reusable plastics, attitudes and risk perceptions might be shaped by other factors beyond usage patterns.

### 3.8. Cognitive Function of Subjects

Cognitive function was assessed using the Ascertain Dementia 8 (AD-8), a simple questionnaire designed to screen for cognitive impairment. The AD-8 consists of eight questions covering domains such as decision-making, activity levels, conversational repetition, learning ability, memory function, financial management, and daily thinking processes. In individuals with a score ≥ 2, cognitive impairment is suspected, and further examination is required to confirm it. In this study, as shown in Table 7, 313 participants (55.52%) scored below 2 on the AD-8, while the remaining 248 (44.48%) scored 2 or higher. 

### 3.9. Correlation of Cognitive Function and Plastic Usage Behavior

An analysis of single use plastic consumption and cognitive function. Table 8 illustrates the correlation between cognitive function and plastic usage patterns, specifically single-use and reusable plastic consumption. The analysis reveals a statistically significant association between single-use plastic usage and cognitive function (*p* = 0.032). Participants with normal cognitive function were more likely to fall into the medium (37.1%) and high (13.0%) single-use plastic categories, whereas those with cognitive impairment were more concentrated in the medium (30.8%) and low (12.2%) usage groups. This suggests that higher single-use plastic consumption might be associated with better cognitive performance, although causality cannot be inferred.

On the other hand, no significant association was found between reusable plastic usage and cognitive function (*p* = 0.605). Both groups, those with normal cognition and those with cognitive impairment, were similarly distributed across low, medium, and high usage levels. This indicates that reusable plastic usage does not appear to have a meaningful relationship with cognitive status in this study population.

Table 9 explores the relationship between cognitive function and three psychosocial domains, awareness and knowledge, attitude, and risk perception, within the context of plastic usage patterns. The results show a statistically significant association between awareness and knowledge and cognitive function (*p* = 0.014). Participants with low awareness and knowledge were more likely to exhibit cognitive impairment (n = 33) than those with normal cognitive function (n = 11), suggesting that lower levels of awareness may be linked to poorer cognitive status.

In contrast, no statistically significant associations were observed for attitude (*p* = 0.110) or risk perception (*p* = 0.065), although the latter approached significance. For both variables, the distribution of cognitive function across low, medium, and high levels was relatively balanced. These findings suggest that while awareness and knowledge may be an important factor associated with cognitive status, attitude and risk perception appear to play a less direct role in cognitive function within this study population.

### 3.10. Multivariate Analysis

Multivariate logistic regression analysis was conducted to identify factors independently associated with suspected cognitive impairment, as measured by the AD-8 screening tool. As shown in Table 10, after controlling age, education, awareness, attitude, and risk perception, the results showed that higher levels of single-use plastic consumption were significantly associated with an increased likelihood of cognitive impairment (OR = 1.409, 95% CI: 1.032–1.924, *p* = 0.031). This suggests that individuals with greater exposure to single-use plastic products had approximately 41% higher odds of reporting cognitive complaints compared to those with lower consumption. In contrast, higher educational attainment was found to be a protective factor, reducing the odds of cognitive impairment by about 18.5% (OR = 0.815, 95% CI: 0.695–0.954, *p* = 0.011). Similarly, the variable coded as age (VAR00001) was also significantly protective (OR = 0.739, 95% CI: 0.562–0.971, *p* = 0.030), although the direction of this association should be interpreted cautiously and may depend on the coding scheme used for age. On the other hand, awareness, attitude, and risk perception toward microplastics were not significantly associated with cognitive status in the adjusted model (all *p* > 0.05), indicating that while these psychosocial factors may influence behavior, they do not appear to have a direct independent effect on cognitive outcomes within this dataset.

## 4. Discussion

In this cross-sectional survey of 562 adults residing in Greater Jakarta, we observed four interrelated domains linking plastic exposure and neurocognitive health: (1) higher single-use plastic consumption was associated with greater odds of suspected cognitive impairment on the AD-8 (*p* = 0.032), whereas reusable plastic use showed no such relationship; (2) awareness/knowledge of microplastics was uneven, roughly one in five respondents had never heard the term, and lower awareness was associated with higher consumption of single-use plastic; (3) more favorable attitudes toward reducing microplastic exposure clustered in mid-age and higher-education strata and were associated with lower bottled water use; and (4) lower risk perception correlated with higher bottled water consumption, and risk perception, attitudes, and awareness were strongly interrelated. Collectively, these findings support an upstream behavioral pathway: awareness, attitudes, risk perception, and consumption behaviors, that may eventually translate into differential microplastic exposure burdens and, plausibly, neurocognitive outcomes.

### 4.1. Cognitive Function and Plastic Use Behavior

Participants in the highest tier of single-use plastic consumption exhibited a larger proportion of abnormal AD-8 scores compared with those in the low-consumption tier (*p* = 0.032). Age and education were also associated with cognitive status (older age and lower education linked with more impairment), underscoring the need to consider confounding factor. Reusable plastic frequency was not related to AD-8 performance. These data suggest that intensity and type of plastic engagement, particularly disposable food and beverage interfaces, may matter more than mere plastic contact frequency. However, it is important to note that the self-reported AD-8 remains a subjective measure, which may introduce bias into the results.

Multiple analytic studies now document substantial MPs loads in commercially bottled water. A 2024 high-throughput imaging study reported a mean of ~240,000 plastic particles per liter across leading brands, ~90% in the nanoplastic range, orders of magnitude above earlier microplastic counts. Heating, storage duration, and cap liner materials further influence shedding. High dependence on single-use, ready-to-consume products therefore represents a plausible exposure pathway aligning with our behavioral signal. Reviews integrating gut–brain axis data indicate that orally ingested MPs disrupt intestinal barrier integrity, alter microbiota, provoke systemic inflammation, and secondarily open the BBB, facilitating neuroimmune activation. Within the brain, microglial phagocytosis of plastics triggers pro-inflammatory cascades (TNF-α, IL-1β, IL-6) and reactive oxygen species, ultimately affecting synaptic function and neuronal survival. This finding suggests that excessive exposure to plastics, potentially through ingestion of microplastics or associated chemical additives, might contribute to neurocognitive complaints [15,16,17].

This causality cannot be established due to the cross-sectional design. The observed association could reflect reverse causation (individuals with early cognitive change relying more on convenience packaging) or unmeasured lifestyle factors (diet quality, socioeconomic stress, sleep, comorbidities) correlated with both plastic use and cognition. The young skew of the sample and reliance on self-reported behaviors introduce additional measurement error. Still, the specificity for single-use (vs. reusable) plastics and alignment with known high-exposure matrices strengthen biological plausibility [18,19,20,21,22].

### 4.2. Awareness of Microplastics

About 20% of respondents had never heard of microplastics, and one-third had never sought information on the topic. While most recognized macroplastic fragmentation and industrial sources, fewer identified secondary household sources (synthetic textiles, personal care products, airborne particles) or contaminated food items like salt and seafood. These gaps indicate a need for targeted education on common exposure routes in Indonesia. Digital media remains a key channel for younger audiences, but older or less connected groups may require community-based outreach. Low awareness was linked to higher single-use plastic consumption. Although awareness was not directly associated with AD-8 scores, its influence on consumption patterns highlights its preventive importance. Public health messaging should emphasize practical household measures, such as using filtered water, avoiding heating food in plastic, selecting low-plastic salt or seafood, and using fabric filters for laundry. Incorporating these strategies into primary care counseling and neurology outreach may strengthen public awareness and reduce exposure [23,24,25,26,27].

### 4.3. Attitudes Towards Microplastics

Respondents showed positive attitudes, particularly among those aged 31–49 and those with higher education. Positive attitudes were associated with lower reusable plastic consumption, though many respondents with favorable attitudes still reported high plastic use, reflecting the “intention–behavior gap.” Barriers such as cost, convenience, and perceived tap water safety likely affect behavior. Behavioral interventions should pair positive messaging with structural supports, such as affordable water filtration, non-plastic refill stations, and clear product labeling. Social norm campaigns and nudges (default non-plastic options in public services) could help translate attitudes into practice [25,26,28,29,30].

### 4.4. Risk Perceptions of Microplastics

Risk perception did not differ significantly by demographic factors but correlated with behaviors. Low-risk individuals consumed more reusable plastic products while those with high-risk perception had lower consumption. Risk perception aligned with both attitudes and awareness, forming a clear cascade from information to behavior. While AD-8 scores overall did not correlate with risk perception, a specific cognitive item was linked to risk ratings among those with suspected impairment. This suggests cognitive decline may influence risk appraisal, warranting further longitudinal research [27,28,29,30,31,32,33,34,35,36].

## 5. Conclusions

This study reveals a noteworthy link between single-use plastic consumption and self-reported cognitive impairment among adults in the Jakarta area. Although the cross-sectional design limits causal inference, the observed pattern is consistent with emerging experimental evidence suggesting that microplastics, through mechanisms such as oxidative stress and neuroinflammation, may exert neurotoxic effects. Public awareness and knowledge of microplastics varied significantly across age, gender, and education levels. Individuals with lower awareness were more likely to report higher consumption of single-use plastics, indicating a potential gap in health literacy that could shape everyday environmental exposures. Notably, many respondents expressed concern about microplastics, yet these attitudes did not always translate into reduced plastic use, highlighting an “intention–behavior” gap influenced by factors such as cost, convenience, and perceived risk.

After adjusting for potential confounders in the multivariable model, the key variables central to our hypothesis—awareness, attitude, and risk perception—were not statistically significant predictors of cognitive impairment (*p* > 0.05 for all). This suggests that future interventions should extend beyond simply raising awareness, as public understanding of plastic pollution may already be relatively high. Instead, efforts should focus on translating awareness into sustained behavioral change through accessible health education, stronger regulatory policies on plastic use, and preventive strategies addressing both individual behaviors and broader social–environmental determinants of health.

Based on the survey findings, several policy recommendations can be proposed. First, phasing out virgin plastic production through national reduction targets should be prioritized, alongside incentives to promote the use of environmentally friendly alternative materials. Refill and reuse packaging systems should be strengthened by providing fiscal or regulatory support for industries adopting sustainable practices. Establishing quality standards for microplastics in food and the environment is essential to safeguard public health and ecosystems, while advancing water purification technologies capable of removing microplastics is critical to ensuring safe drinking water. Furthermore, industry transparency should be mandated to provide clear information on the chemical composition and pollution risks associated with plastic packaging. Finally, improving waste management at the local level requires enhancing collection, sorting, and treatment systems through active collaboration between communities and local governments to achieve effective and sustainable waste management.

In conclusion, increasing public awareness of microplastic-related risks and reducing dependence on single-use plastics are not only vital environmental actions but may also contribute to the preservation of cognitive health. Our findings highlight the need for integrated public health strategies that unite environmental sustainability with neurological well-being.

## Figures and Tables

**Table 1:** Subject Demographics.

Characteristics	Value (*n* = 562)
Age (median [min–max])	29 (19–69)
Gender (*n* [%])	
Female	351 (62.46)
Male	211 (37.54)
Marital status (*n* [%])	
Marry	211 (37.54)
Not married	323 (57.47)
Widow/Widower	28 (4.98)
Expenses (*n* [%])	
IDR 354,000	41 (7.30%)
IDR 354,000–532,000	25 (4.45%)
IDR 532,000–1,200,000	103 (18.33%)
IDR 1,200,000–6,000,000	293 (52.14%)
>IDR 6,000,000	100 (17.79%)
Education level	
Elementary school	8 (1.42)
Junior high school	12 (2.14)
Senior high school	129 (22.95)
Diploma degree	41 (7.30)
Bachelor’s degree	312 (55.52)
Master’s degree	52 (9.25)
Doctoral degree	8 (1.42)

**Table 2:** Correlation of subject’s demographics with perceived risk level of single-use and reusable items. Analysis using Chi-Square.

Characteristics	Single-Use				Reusable			
	Low (*n* = 44)	Medium (*n =* 381)	High (*n* = 136)	*p* Value	Low (*n* = 182)	Medium (*n* = 193)	High (*n* = 267)	*p* Value
Age				0.001 *				0.391 *
≤30 (*n* = 330)	15 (34.1%)	233 (61.2%)	82 (60.3%)		100 (54.9%)	152 (78.8%)	78 (29.2%)	
31–49 (*n* = 183)	19 (43.2%)	118 (31.0%)	45 (33.1%)		61 (33.5%)	86 (44.6%)	35 (13.1%)	
≥50 (*n* = 49)	10 (22.7%)	30 (7.9%)	9 (6.6%)		21 (11.5%)	19 (9.8%)	9 (3.4%)	
Gender				0.669 *				0.184 *
Male (*n* = 211)	19 (43.2%)	139 (36.5%)	52 (38.2%)		71 (39.0%)	102 (52.8%)	37 (13.9%)	
Female (*n* = 351)	25 (56.8%)	242 (63.5%)	48 (35.3%)		111 (61.0%)	155 (80.3%)	85 (31.8%)	
Education Level				0.001 *				0.266 *
Low (*n* = 149)	6 (13.6%)	78 (20.5%)	45 (33.1%)		35 (19.2%)	62 (32.1%)	32 (12.0%)	
High (*n* = 413)	35 (79.5%)	295 (77.4%)	82 (60.3%)		141 (77.5%)	188 (97.4%)	83 (31.1%)	

* *p* < 0.05 was considered statistically significant. Bold values in the table indicate statistically significant *p*-values. Non-significant values are not bolded.

**Table 3:** Awareness and knowledge toward microplastics analyzed using Chi-Square.

Characteristics	Awareness and Knowledge			*p*-Value
	Good (*n* = 180)	Intermediate (*n* = 243)	Bad (*n* = 139)	
Age (*n* [%])				0.007 *
≤30	115 (63.89%)	145 (59.67%)	70 (50.35%)	
31–49	56 (31.11%)	80 (32.92%)	47 (33.81%)	
≥50	9 (0.05%)	18 (7.40%)	22 (15.82%)	
Gender (*n* [%])				0.004 *
Male	97 (53.8%)	154 (63.3%)	100 (71.9%)	
Female	83 (46.11%)	89 (36.6%)	39 (28.0%)	
Education level (*n* [%])				0.027 *
Low (*n* = 149)	45 (25.0%)	56 (23.0%)	48 (34.5%)	
High (*n* = 413)	135 (75.0%)	187 (77.0%)	91 (65.4%)	

* *p* < 0.05 was considered statistically significant. Bold values in the table indicate statistically significant *p*-values. Non-significant values are not bolded.

**Table 4:** Attitudes towards Microplastics analyzed using Chi-Square.

Characteristics	Attitude Toward of Microplastics			*p*-Value
	Good (*n* = 271)	Intermediate (*n* = 176)	Bad *(n* = 115)	
Age (*n* [%])				0.037 *
≤30 (*n* = 330)	156 (57.56%)	115 (65.34%)	59 (51.30%)	
31–49 (*n* = 183)	94 (34.68%)	50 (28.40%)	39 (17.78%)	
≥50 (*n* = 49)	21 (7.74%)	11 (6.25%)	17 (14.78%)	
Gender (*n* [%])				0.360
Male (*n* = 211)	162 (59.77%)	117 (66.47%)	72 (62.60%)	
Female (*n* = 351)	108 (39.85%)	59 (33.52%)	43 (37.39%)	
Education level (*n* [%])				<0.001 *
Low (*n* = 149)	88 (32.47%)	23 (13.06%)	38 (33.04%)	
High (*n* = 413)	183 (67.52%)	153 (86.93%)	77 (66.95%)	

* *p* < 0.05 was considered statistically significant. Bold values in the table indicate statistically significant *p*-values. Non-significant values are not bolded.

**Table 5:** Risk Perception of Microplastics analyzed using Chi-Square.

Characteristics	Risk Perception of Microplastic			*p*-Value
	High (*n* = 160)	Intermediate (*n* = 202)	Low (*n* = 200)	
Age (*n* [%])				0.344
≤30 (*n* = 330)	91 (56.87)	118 (58.41)	121 (60.5)	
31–49 (*n* = 183)	59 (36.87)	67 (33.16)	57 (28.5)	
≥50 (*n* = 49)	10 (6.25)	17 (8.41%)	22 (11)	
Gender (*n* [%])				0.289
Male (*n* = 211)	54 (25.59)	118 (43.8%)	127 (63.5)	
Female (*n* = 351)	106 (30.20)	84 (42.2%)	73 (36.5)	
Education level (*n* [%])				0.184
Low (*n* = 149)	33 (20.62)	62 (30.69)	54 (27.0%)	
High (*n* = 413)	127 (79.37)	140 (69.30)	146 (73%)	

**Table 6:** Awareness, Attitude, and Risk Perception with Plastic Usage Pattern analyzed using Chi-Square.

Variable	Single-Use Plastic (*p*-Value)	Reusable Plastic (*p*-Value)
Awareness and knowledge	0.205	0.044 *
Attitude	0.561	0.768
Risk perception	0.576	0.329

* *p* < 0.05 was considered statistically significant. Bold values in the table indicate statistically significant *p*-values. Non-significant values are not bolded.

**Table 7:** Correlation of cognitive function with subject demographics.

Characteristics	Cognitive Function		*p*-Value
	Normal (AD-8 0–1) (*n* = 313)	Cognitive Impairment (AD-8 ≥ 2) (*n* = 248)	
Age (median [min–max])	24 (19-54)	31 (23-69)	<0.001 *
Gender (*n* [%])			0.945 **
Male (*n* = 211)	130 (41.7%)	81 (32.4%)	
Female (*n* = 351)	182 (58.3%)	169 (67.6%)	
Education level (*n* [%])			0.008 **
Low (*n* = 149)	65 (20.8%)	84 (33.6%)	
High (*n* = 413)	247 (79.2%)	166 (66.4%)	

* Analysis using Mann–Whitney; ** Analysis using Chi-Square; *p* < 0.05 was considered statistically significant. Bold values in the table indicate statistically significant *p*-values. Non-significant values are not bolded.

**Table 8:** Correlation of cognitive function with plastic usage pattern analyzed using Chi- Square.

Plastic Usage Pattern		Cognitive Function		*p*-Value
		Normal (AD-8 0–1) (*n* = 313)	Cognitive Decline (AD-8 ≥ 2) (*n* = 248)	
Single use plastic	Low (*n* = 44)	32 (5.7%)	12 (12.2%)	0.032 *
	Medium (*n* = 381)	208 (37.1%)	173 (30.8%)	
	High (*n* = 136)	73 (13.0%)	63 (11.2%)	
Reusable use plastic	Low (*n* = 182)	60 (19.2%)	42 (16.8%)	0.605
	Medium (*n* = 193)	104 (33.3%)	89 (35.6%)	
	High (*n* = 267)	148 (47.4%)	119 (47.6%)	

* *p* < 0.05 was considered statistically significant. Bold values in the table indicate statistically significant *p*-values. Non-significant values are not bolded.

**Table 9:** Correlation of cognitive function with psychosocial domains analyzed using Chi-Square.

Psychosocial Domains		Cognitive Function		*p*-Value
		Normal (AD-8 0–1) (*n* = 313)	Cognitive Decline (AD-8 ≥ 2) (*n* = 248)	
Awareness and knowledge	Low (*n* = 44)	11	33	0.014 *
	Medium (*n* = 381)	212	169	
	High (*n* = 136)	68	68	
Attitude	Low (*n* = 182)	95	87	0.110
	Medium (*n* = 193)	82	111	
	High (*n* = 267)	136	131	
Risk perception	Low (*n* = 200)	102	98	0.065 *
	Medium (*n* = 202)	110	92	
	High (*n* = 160)	101	59	

* *p* < 0.05 was considered statistically significant. Bold values in the table indicate statistically significant *p*-values. Non-significant values are not bolded.

**Table 10:** Multivariate Logistic Regression Analysis.

Variables	B	S.E	Wald	*p*-Value	95% CI
Age	−0.303	0.139	4.722	0.030 *	0.562–0.971
Education level	−0.205	0.081	6.456	0.011 *	0.695–0.954
Single-use plastic usage	0.343	0.159	4.658	0.031 *	1.032–1.924
Awareness and knowledge of Microplastics	0.310	0.089	0.122	0.727	0.867–1.228
Attitude toward microplastics	−0.035	0.034	1.104	0.293	0.904–1.031
Risk perception of microplastic	0.005	0.023	0.039	0.843	0.904–1.031

* *p* < 0.05 was considered statistically significant. Bold values in the table indicate statistically significant *p*-values. Non-significant values are not bolded.

## Data Availability

The datasets generated and/or analyzed during this study are available from the corresponding author upon reasonable request.

## Supplementary Materials

The following supporting information can be downloaded at: https://www.mdpi.com/article/10.3390/ijerph23010067/s1, Table S1: Ascertain Dementia-8 (AD-8) score, Table S2: Awareness and Knowledge toward Microplastics, Table S3. Attitudes toward Microplastics, Dataset S1.
