# Supplementary material for: Frequent Plastic Usage Behavior and Lack of Microplastic Awareness Correlates with Cognitive Decline: A Cross-Sectional Survey
Source: Int J Environ Res Public Health. 2026 Jan 1;23(1):67. doi: 10.3390/ijerph23010067 (PMC12841581; doi:10.3390/ijerph23010067)
Supplement: Supplementary file 1 [file ijerph-23-00067-s001.zip › supplementary dataset S1.pdf]

Timestamp		Score	Kesediaan responder	Kesediaan kedatangan Apakah Anda berkenan bila dilakukan pemeriksaan lanjutan dengan pengambilan sampel urine, feses, dan darah? Pemeriksaan ini akan dilakukan di rumah sakit yang berada di Jakarta. Untuk kesediaan Anda akan kami berikan sebuah hadiah apresiasi kedatangan.	Usia	Tempat lahir	Jenis kelamin	Jika sudah menika	Jika Ya, apa jen	Pekerjaan	Pengeluaran pe	Tingkat Pendidi	Berat badan	Tinggi badan		Apakah Anda m (co: epilepsi, st	Apakah Anda m	apakah anda be	Pernahkah And	Darimana Anda	Apakah Anda p
27/2023 1:17:44	1	5 / 6	Saya bersedia untuk m	Ya	48	Jakarta	Perempuan	TIDAK		Freelance	Rp532.000 – Rp	D4/S1	58 kg	168 cm					Ya, Sering	Internet, Jurna li	Terkadang (>50%
7/2023 16:51:32	2	2 / 6	Saya bersedia untuk m	Ya	38	Jakarta	Perempuan	TIDAK		Guru	Rp1.200.000 - R	D4/S1	62	155		Tidak	Tidak		Ya, Sering	Buku, Brosur Inf	Terkadang (>50%
1/2023 19:15:08	3	1 / 6	Saya bersedia untuk m	Ya	64	Jakarta	Perempuan	TIDAK		Swasta	> Rp. 6.000.000	S2	62	162		Tidak ada	Tidak ada	Tidak	Ya, sering	Lainnya	Jarang (<50%)
9/2023 14:58:49	4	1 / 6	Saya bersedia untuk m	Ya	40	Jakarta	Perempuan	TIDAK	-	Dokter	> Rp. 6.000.000	S2	53	163		Tidak ada	Tidqk ada	belum menikah /	Ya, sering	Buku, Lainnya	Tidak Pernah
8/2023 21:18:41	5	1 / 6	Saya bersedia untuk m	Ya	53	Tulungagung	Laki - laki	TIDAK		Sopir	Rp1.200.000 - R	SD/MI	54 kg	170 cm		Tidak	Tidak	Tidak	Ya, sering	Teman dan keral	Selau
1/2023 16:09:26	6	4 / 6	Saya bersedia untuk m	Ya	19	Semarang	Perempuan	TIDAK		Mahasiswa	Rp. 354.000.00 -	D3	45	157				belum menikah /	Ya, sering	Teman dan keral	Terkadang (>50%
1/2023 12:35:08	7	2 / 6	Saya bersedia untuk m	Ya	26	padang	Perempuan	BELUM MENIKAH		pelajar	Rp532.000 – Rp	SMA/SMU	54	162				belum menikah /	Ya, sering	Internet, Jurna li	Terkadang (>50%
8/2023 11:48:16	8	3 / 6	Saya bersedia untuk m	Ya	27	Sukoharjo	Perempuan	BELUM MENIKAH		Mahasiswa	Rp1.200.000 - R	S2	66	157				belum menikah /	Ya, sering	Teman dan keral	Selau
3/2023 20:28:02	9	2 / 6	Saya bersedia untuk m	Ya	50	Bogor	Laki - laki	TIDAK		Karyawan swast	Rp1.200.000 - R	D4/S1	62	170		-	-		Ya, Sering	Internet, Televisi	Terkadang (>50%
1/2023 12:05:09	10	5 / 6	Saya bersedia untuk m	Ya	29	Wonogiri	Perempuan	TIDAK		Swasta	> Rp. 6.000.000	S2	55	160		Tidak	Tidak	Ya	Ya, sering	Teman dan keral	Selau
1/2023 18:17:10	11	3 / 6	Saya bersedia untuk m	Ya	50	Kediri	Perempuan	TIDAK	Tidak	ASN	> Rp. 6.000.000	S3	50	150		Tidak ada	Tidak ada	Tidak	Ya, sering	Internet	Jarang (<50%)
1/2023 19:06:09	12	3 / 6	Saya bersedia untuk m	Ya	31	Aceh	Perempuan	TIDAK		Ppds	< Rp. 354.000.0	D4/S1	80	160		Tidak	Tidak	belum menikah /	Tidak	Lainnya	Tidak Pernah
8/2023 12:31:16	13	2 / 6	Saya bersedia untuk m	Ya	24	Jakarta	Perempuan	BELUM MENIKAH		Pekerja lepas	Rp1.200.000 - R	D4/S1	57	170		Sakit kepala menahun		belum menikah /	Ya, kadang - kad	Teman dan keral	Jarang (<50%)
2/2023 17:49:07	14	2 / 6	Saya bersedia untuk m	Ya	25	Bekasi	Perempuan	TIDAK		Freelance	Rp532.000 – Rp	D4/S1	52	162					Ya, Sering	Internet, Sosial M	Terkadang (>50%
1/2023 18:34:16	15	3 / 6		Ya	29	Jakarta	Perempuan	TIDAK		PPDS	> Rp. 6.000.000	D4/S1	62	158		tidak	tidak	belum menikah /	Tidak	Lainnya	Tidak Pernah
9/2023 15:04:53	16	3 / 6	Saya bersedia untuk m	Ya	27	Jakarta	Laki - laki	BELUM MENIKAH		Dokter Umum	Rp532.000 – Rp	D4/S1	85	167		Tidak ada	Tidak ada	belum menikah /	Ya, kadang - kad	Internet, Televisi	Terkadang (>50%
2/2023 21:37:22	17	3 / 6	Saya bersedia untuk m	Ya	24	Jakarta	Laki - laki	TIDAK	Tidak	Freelance	> Rp. 6.000.000	D4/S1	59	158		Tidak	Tidak		Ya, Sering	Internet, Buku, T	Jarang (<50%)
5/2023 14:04:55	18	4 / 6	Saya bersedia untuk m	Ya	49	Magelang	Perempuan	TIDAK		Peneliti	Rp1.200.000 - R	S3	69	155		Tidak	Asma, cleft lips and palate		Ya, Sering	Internet	Terkadang (>50%
6/2023 14:53:33	19	3 / 6	Saya bersedia untuk m	Ya	28	Magelang	Perempuan	YA	Suntik	Ibu Rumah Tang	Rp1.200.000 - R	SMA/SMU	48	155		tidak ada	tidak ada	belum menikah /	Ya, sering	Internet, Buku, R	Terkadang (>50%
1/2023 16:22:31	20	2 / 6	Saya bersedia untuk m	Ya	28	Surabaya	Perempuan	TIDAK		Dokter	> Rp. 6.000.000	S2	40	156				belum menikah /	Ya, sering	Internet	Terkadang (>50%
2/2023 19:41:08	21	1 / 6	Saya bersedia untuk m	Ya	23	Jakart	Perempuan	TIDAK		Wirausaha	Rp1.200.000 - R	D4/S1	65	167				belum menikah /	Tidak	Lainnya	Tidak Pernah
8/2023 12:27:49	22	2 / 6		Ya	58	Boyolali	Perempuan	TIDAK		Mengurus rumah	Rp1.200.000 - R	D4/S1	72	156		Tidak	Tidak		Ya, kadang - kad	Internet, Televisi	Jarang (<50%)
7/2023 19:52:38	23	2 / 6	Saya bersedia untuk m	Ya	28	Talang tinggi	Perempuan	TIDAK		IRT	Rp1.200.000 - R	D4/S1	70	155				Tidak	Ya, kadang - kad	Internet, Televisi	Jarang (<50%)
0/9/2023 0:20:04	24	2 / 6	Saya bersedia untuk m	Ya	44	Jakarta	Perempuan	TIDAK		Dosen	Rp1.200.000 - R	S2	66	157				Tidak	Ya, kadang - kad	Internet, Sosial M	Jarang (<50%)
2/8/2023 9:43:23	25	2 / 6	Saya bersedia untuk m	Ya	27	Lhoksukon	Laki - laki	BELUM MENIKAH		Karyawan Swast	Rp1.200.000 - R	D4/S1	62	170				belum menikah /	Ya, sering	Jurna lingkungan	Terkadang (>50%
0/2023 13:08:03	26	1 / 6	Saya bersedia untuk m	Ya	60	Jakarta	Perempuan	TIDAK	-	Ibu rumah tangg	Rp532.000 – Rp	SMA/SMU	61 kg	143 cm		obat etrostop (bu	-	Tidak	Tidak	Lainnya	Tidak Pernah
6/2023 14:42:39	27	1 / 6		Ya	28	Medan	Perempuan	TIDAK	Masih masa nifa	Ibu rumah tangg	Rp532.000 – Rp	SMA/SMU	55	153		Nyeri dada sebe	Tidak	Tidak	Ya, kadang - kad	Internet	Terkadang (>50%
1/2023 17:05:43	28	3 / 6	Saya bersedia untuk m	Ya	67	Jakarta	Perempuan	TIDAK		Pensiun (dosen)	> Rp. 6.000.000	S3	58 kg	161 cm		Ya	Cacat 4 jari di ta	belum menikah /	Ya, sering	Teman dan keral	Tidak Pernah
0/2/2023 1:02:19	29	0 / 6	Saya bersedia untuk m	Ya	35	Jakarta	Laki - laki	TIDAK		Wiraswasta	Rp532.000 – Rp	SMA/SMU	50	170		Tidak	Tidak		Ya, kadang - kad	Internet	Jarang (<50%)
14/2023 8:41:59	30	2 / 6	Saya bersedia untuk m	Ya	44	Soroako, Sulawe	Perempuan	TIDAK		Wirausaha	Rp1.200.000 - R	D4/S1	43	163		Tidak	Tidak		Ya, Kadang - kad	Internet	Jarang (<50%)
1/2023 15:53:11	31	3 / 6	Saya bersedia untuk m	Ya	33	Rumbai	Laki - laki	TIDAK		Dokter	< Rp. 354.000.0	D4/S1	174	75		tidak	Tidak	Tidak	Ya, sering	Teman dan keral	Terkadang (>50%
0/2023 14:42:02	32	2 / 6	Saya bersedia untuk m	Ya	22	Jakarta	Perempuan	BELUM MENIKAH		Freelance peneli	Rp1.200.000 - R	D4/S1	66	163		-	Alergi	belum menikah /	Ya, kadang - kad	Teman dan keral	Jarang (<50%)
5/2023 18:03:50	33	1 / 6	Saya bersedia untuk m	Ya	21	Palembang	Perempuan	TIDAK		Mahasiswa	Rp1.200.000 - R	D4/S1	48	157					Tidak	Sosial Media	Tidak Pernah
1/2023 13:23:01	34	2 / 6	Saya bersedia untuk m	Ya	23	Halmahera Timu	Laki - laki	TIDAK	Tidak	Mahasiswa	< Rp. 354.000.0	D4/S1	49	157		Tidak	Tidak		Ya, Kadang - kad	Internet, Sosial M	Jarang (<50%)
4/2023 20:47:02	35	1 / 6	Saya bersedia untuk m	Ya	35	Jakarta	Perempuan	TIDAK		Karyawab swast	> Rp. 6.000.000	D4/S1	52	160		Tidak	Tidak		Ya, Kadang - kad	Lainnya	Tidak Pernah
6/2023 17:39:18	36	2 / 6	Saya bersedia untuk m	Ya	38	Jakarta	Perempuan	YA	IUD	Karyawan swast	Rp1.200.000 - R	SMA/SMU	65	168		Tidak	Tidak	Tidak	Ya, sering	Internet	Jarang (<50%)
6/2023 15:21:39	37	1 / 6	Saya bersedia untuk m	Ya	21	Jakarta	Perempuan	TIDAK		Mahasiswa	< Rp. 354.000.0	D3	59	159				belum menikah /	Ya, kadang - kad	Internet, Sosial M	Terkadang (>50%
8/2023 11:13:01	38	2 / 6	Saya bersedia untuk m	Ya	22	Tangerang, Indo	Perempuan	BELUM MENIKAH		Mahasiswa	Rp. 354.000.00 -	SMA/SMU	85	168				Tidak	Tidak	Sosial Media	Tidak Pernah
2/6/2023 0:06:31	39	2 / 6	Saya bersedia untuk m	Ya	27	Jakarta	Perempuan	TIDAK		Staf NGO	> Rp. 6.000.000	S2	75	167				belum menikah /	Ya, sering	Buku, Surat Kab	Selau
2/8/2023 9:09:32	40	3 / 6	Saya bersedia untuk m	Ya	36	koto baru	Laki - laki	TIDAK		Pegawai pemeri	Rp1.200.000 - R	S2	62	170		-	-	Ya	Ya, kadang - kad	Internet, Sosial M	Terkadang (>50%
2/9/2023 6:34:55	41	3 / 6	Saya bersedia untuk m	Ya	24	Bangkalan	Laki - laki	BELUM MENIKAH		Wiraswasta	Rp1.200.000 - R	SMA/SMU	78kg	168cm		Tidak ada	Tidak ada	belum menikah /	Ya, sering	Teman dan keral	Terkadang (>50%
7/2023 22:42:05	42	5 / 6	Saya bersedia untuk m	Ya	34	Bandung	Laki - laki	TIDAK	N/A	Karyawan lemba	> Rp. 6.000.000	S2	92	169		N/A	N/A		Ya, Sering	Teman dan keral	Selau
9/2023 21:18:56	43	3 / 6		Ya	30	Jakarta	Perempuan	TIDAK		Karyawan Swast	> Rp. 6.000.000	D3	49kg	155cm		Tidak ada	Tidak ada	Ya	Ya, sering	Internet, Buku, B	Terkadang (>50%
0/2023 22:14:44	44	2 / 6	Saya bersedia untuk m	Ya	30	Wamena	Laki - laki	TIDAK		PNS	> Rp. 6.000.000	D4/S1	63	168		-	-	Tidak	Ya, sering	Internet, Jurna li	Jarang (<50%)
8/2023 10:47:32	45	5 / 6	Saya bersedia untuk m	Ya	35	Pontianak	Perempuan	TIDAK		Ibu Rumah Tang	Rp1.200.000 - R	D4/S1	48	153				Tidak	Ya, sering	Internet, Sosial M	Terkadang (>50%
0/2023 10:16:03	46	1 / 6	Saya bersedia untuk m	Ya	43	Jakarta	Perempuan	YA	Alat kontrasepsi	Pegawai swasta	> Rp. 6.000.000	D4/S1	89	161		Tidak	Tidak	Ya	Tidak	Lainnya	Tidak Pernah
0/2023 13:03:24	47	2 / 6	Saya bersedia untuk m	Ya	40	Rangkasbitung	Perempuan	YA	suntik 3 bulan	IRT	Rp1.200.000 - R	SMP/MTS	63 kg	151		tidak	tidak	Ya	Tidak	Lainnya	Tidak Pernah
6/2023 15:33:46	48	2 / 6		Ya	31	Majalengka	Laki - laki	TIDAK		Project Manager	Rp1.200.000 - R	D3	80	168		Tidak	Tidak		Ya, Sering	Internet	Terkadang (>50%
9/2023 15:16:30	49	2 / 6	Saya bersedia untuk m	Ya	25	Bandung	Laki - laki	TIDAK		Wirausaha	Rp1.200.000 - R	SMA/SMU	75	170					Ya, Sering	Internet, Buku	Selau
0/8/2023 3:53:45	50	2 / 6	Saya bersedia untuk m	Ya	30	sukabumi	Perempuan	YA	pil	buruh harian lep	Rp1.200.000 - R	SMP/MTS	52	153		tidak ada	tidak ada	belum menikah /	Ya, kadang - kad	Internet	Jarang (<50%)
9/2023 14:49:43	51	2 / 6	Saya bersedia untuk m	Ya	56	Medwn	Laki - laki	TIDAK		Swastw	Rp1.200.000 - R	D4/S1	77 Kg	165 cm		Penyakit jantung	Tidak	Tidak	Ya, sering	Internet	Terkadang (>50%
23/2023 8:53:06	52	2 / 6	Saya bersedia untuk m	Ya	22	Jakarta	Laki - laki	BELUM MENIKAH		Mahasiswa	Rp1.200.000 - R	D4/S1	65	168				belum menikah /	Ya, kadang - kad	Internet, Buku, R	Jarang (<50%)
0/2023 18:43:34	53	2 / 6	Saya bersedia untuk m	Ya	24	Jakarta	Laki - laki	BELUM MENIKAH		Karyawan Swast	Rp532.000 – Rp	SMA/SMU	78	178		Tidak ada	Tidak ada	belum menikah /	Ya, kadang - kad	Internet, Sosial M	Terkadang (>50%
18/2023 7:00:58	54	2 / 6	Saya bersedia untuk m	Ya	30	Bogor	Laki - laki	TIDAK		Freelancer	Rp532.000 – Rp	S2	73	175		Tidak ada	Tidak ada		Ya, Sering	Teman dan keral	Terkadang (>50%
8/2023 23:08:45	55	1 / 6		Ya	52	Jakarta	Laki - laki	TIDAK		Swasta	Rp1.200.000 - R	D3	75	168					Tidak	Sosial Media	Tidak Pernah
0/1/2023 1:31:58	56	3 / 6	Saya bersedia untuk m	Ya	30	Bekasi	Laki - laki	TIDAK	Tidak	Wirausaha	Rp1.200.000 - R	SMA/SMU	58	163		Tidak	Tidak		Ya, kadang - kad	Internet, Buku, S	Jarang (<50%)
3/2023 13:22:59	57	1 / 6	Saya bersedia untuk m	Ya	21	Bekasi	Perempuan	TIDAK		Mahasiswa	Rp1.200.000 - R	D4/S1	65	160				belum menikah /	Ya, kadang - kad	Internet, Jurna li	Jarang (<50%)
1/2023 16:10:24	58	2 / 6	Saya bersedia untuk m	Ya	28	Depok	Perempuan	TIDAK		Mahasiswa	Rp1.200.000 - R	D4/S1	Mahasiswa	156		Tidak	Tidak	belum menikah /	Tidak	Lainnya	Tidak Pernah
6/2023 19:09:12	59	2 / 6	Saya bersedia untuk m	Ya	22	Bekasi	Perempuan	TIDAK		Pelajar	Rp1.200.000 - R	D4/S1	53	160				belum menikah /	Tidak	Teman dan keral	Tidak Pernah
9/2023 16:30:36	60	2 / 6	Saya bersedia untuk m	Ya	27	Tangerang	Laki - laki	BELUM MENIKAH		Dokter Umum	> Rp. 6.000.000	D4/S1	84	188		Tidak ada	Tidak ada	belum menikah /	Ya, sering	Internet, Televisi	Jarang (<50%)
7/2023 20:23:19	61	3 / 6	Saya bersedia untuk m	Ya	24	Bogor	Laki - laki	BELUM MENIKAH		Senior Seller	Rp532.000 – Rp	D4/S1	80	176		Tidak ada	Tidak ada	belum menikah /	Ya, sering	Teman dan keral	Terkadang (>50%
9/2023 23:14:49	62	4 / 6	Saya bersedia untuk m	Ya	24	Jakarta	Laki - laki	TIDAK		Karyawan	Rp532.000 – Rp	D4/S1	85	180					Ya, Kadang - kad	Internet, Sosial M	Tidak Pernah
13/2023 5:41:10	63	0 / 6	Saya bersedia untuk m	Ya	34	Dki jakaarta	Laki - laki	TIDAK		Freelandce	< Rp. 354.000.0	SMA/SMU	63	179cm		Tidak	Tidak		Ya, Kadang - kad	Internet, Buku, T	Terkadang (>50%
13/2023 7:20:17	64	2 / 6	Saya bersedia untuk m	Ya	31	Jakarta	Perempuan	TIDAK		Freelancer	> Rp. 6.000.000	D4/S1	55	158					Ya, Sering	Internet, Televisi	Terkadang (>50%
15/2023 1:21:11	65	1 / 6	Saya bersedia untuk m	Ya	51	Jakarta	Laki - laki	TIDAK		Pekerja proyek	Rp532.000 – Rp	SMA/SMU	165 kg	168cm		Tidak	Tidak		Ya, Kadang - kad	Internet	Tidak Pernah
7/2023 23:22:03	66	4 / 6	Saya bersedia untuk m	Ya	29	Tegal	Laki - laki	TIDAK		Freelancee	> Rp. 6.000.000	D4/S1	58	180					Ya, Sering	Teman dan keral	Selau
1/2023 16:42:56	67	2 / 6	Saya bersedia untuk m	Ya	45	Batam	Perempuan	TIDAK		Karyawan Swast	Rp1.200.000 - R	D4/S1	68	168		Tidak	Tidak		Ya, kadang - kad	Internet, Sosial M	Terkadang (>50%
8/2023 18:49:48	68	2 / 6	Saya bersedia untuk m	Ya	28	Tomvang	Laki - laki	TIDAK		Mhasiswa	Rp532.000 – Rp	S2	50	220		Tidak ada	Tidak ada	belum menikah /	Ya, kadang - kad	Internet	Tidak Pernah
0/2023 11:08:55	69	2 / 6	Saya bersedia untuk m	Ya	28	Tangerang	Laki - laki	TIDAK		Buruh pabrik	Rp1.200.000 - R	SMA/SMU	53	175				Tidak	Ya, sering	Jurna lingkungan	Terkadang (>50%
7/2023 18:36:24	70	3 / 6	Saya bersedia untuk m	Ya	30	Jakarta	Perempuan	TIDAK		Pegawai Swasta	Rp1.200.000 - R	D4/S1	68	163				belum menikah /	Ya, kadang - kad	Internet, Jurna li	Jarang (<50%)
7/2023 19:22:03	71	2 / 6	Saya bersedia untuk m	Ya	29	Tasikmalaya	Laki - laki	TIDAK		Swasta	Rp1.200.000 - R	D3	70	173		Tidak	Tidak	Tidak	Ya, sering	Internet, Jurna li	Terkadang (>50%
4/2023 19:58:45	72	5 / 6	Saya bersedia untuk m	Ya	21	Jakarta	Laki - laki	TIDAK		Mahasiswa	< Rp. 354.000.0	D3	120	185					Ya, Sering	Teman dan keral	Selau
7/2023 10:47:41	73	4 / 6	Saya bersedia untuk m	Ya	23	Jombang	Perempuan	TIDAK		Staf/Pegawai	Rp1.200.000 - R	D4/S1	47	150		Tidak	Tidak	belum menikah /	Ya, kadang - kad	Internet, Jurna li	Jarang (<50%)
9/2023 12:21:43	74	1 / 6	Saya bersedia untuk m	Ya	40	Jakarta	Perempuan	TIDAK		IRT	Rp1.200.000 - R	S2	51	150		tidak	tidak	Ya	Ya, sering	Internet, Brosur I	Jarang (<50%)
1/2023 21:33:45	75	2 / 6	Saya bersedia untuk m	Ya	33	Kab. Donggala	Perempuan	BELUM MENIKAH		Pekerja sosial	Rp1.200.000 - R	D4/S1	42	152		Tidak	Tidak	belum menikah /	Ya, kadang - kad	Teman dan keral	Tidak Pernah
1/2023 15:29:37	76	3 / 6		Ya	23	Bogor	Perempuan	TIDAK		Marketing	Rp532.000 – Rp	D4/S1	47	158		Tidak ada	Tidak ada		Ya, sering	Internet, Buku, S	Terkadang (>50%
6/2023 17:58:28	77	1 / 6	Saya bersedia untuk m	Ya	24	Karanganyar	Laki - laki	TIDAK	Tidak mengguna	Mahasiswa	Rp1.200.000 - R	D4/S1	97	172		Gangguan Peny	Tidak	belum menikah /	Ya, kadang - kad	Internet, Sosial M	Tidak Pernah
5/2023 13:17:38	78	1 / 6	Saya bersedia untuk m	Ya	21	Demak	Perempuan	TIDAK	Tidak	Mahasiswa	Rp. 354.000.00 -	D3	47	153		Tidak	Tidak		Ya, Kadang - kad	Internet, Sosial M	Jarang (<50%)
20/2023 9:22:34	79	2 / 6	Saya tidak bersedia un	Ya	23	Surabaya	Laki - laki	BELUM MENIKAH		Swasta	Rp532.000 – Rp	D4/S1	70	155				belum menikah /	Tidak	Internet	Terkadang (>50%
8/2023 17:30:05	80	1 / 6	Saya bersedia untuk m	Ya	22	Jakarta	Laki - laki	BELUM MENIKAH		Freelance	Rp532.000 – Rp	D4/S1	63	174				belum menikah /	Ya, sering	Internet, Buku, S	Jarang (<50%)
2/2023 22:42:39	81	3 / 6	Saya bersedia untuk m	Ya	21	Sukabumi	Perempuan	TIDAK		Freelance	Rp532.000 – Rp	SMA/SMU	40	155		Tidak	Tidak		Ya, Sering	Teman dan keral	Selau
4/2023 22:08:19	82	0 / 6	Saya bersedia untuk m	Ya	61	Jakarta	Laki - laki	TIDAK		Driver online mo	Rp532.000 – Rp	D3	42,6 kg	158		Tidak ada yg me	Tidak ada		Tidak	Sosial Media	Jarang (<50%)
7/2023 20:04:10	83	2 / 6	Saya bersedia untuk m	Ya	39	Jakarta	Laki - laki	TIDAK		Swasta	Rp1.200.000 - R	S2	80	178		Tidak ada			Ya, Sering	Internet, Televisi	Selau
18/2023 8:32:40	84	3 / 6	Saya bersedia untuk m	Ya	31	Blora	Laki - laki	TIDAK		Freelance	Rp1.200.000 - R	D4/S1	79	167					Ya, Kadang - kad	Internet, Surat K	Terkadang (>50%
29/2023 1:20:49	85	3 / 6	Saya bersedia untuk m	Ya	28	Kota Tangerang	Laki - laki	TIDAK	Alat kontrasepsi	Wirausaha	Rp1.200.000 - R	D4/S1	84 kg	178		Anxiety	Tidak ada		Ya, sering	Internet, Buku, J	Terkadang (>50%
5/2023 19:49:31	86	2 / 6	Saya bersedia untuk m	Ya	61	Bogor	Laki - laki	TIDAK		Pensiunan	Rp1.200.000 - R	D4/S1	75	163		Tdk ada	Tdk ada	Tidak	Ya, kadang - kad	Sosial Media	Terkadang (>50%
6/2023 14:26:09	87	1 / 6	Saya bersedia untuk m	Ya	24	Bogor	Perempuan	YA	KB 3 bulan seka	Ibu Rumah Tang	Rp532.000 – Rp	SMP/MTS	50	152		Tidak	Tidak	Tidak	Ya, sering	Internet, Televisi	Jarang (<50%)
0/9/2023 6:59:23	88	2 / 6	Saya bersedia untuk m	Ya	59	Lampung	Laki - laki	TIDAK		Swasta	> Rp. 6.000.000	S2	86	170		Diabetes & hypertensi		Ya	Ya, sering	Internet, Televisi	Terkadang (>50%
0/2023 11:40:17	89	0 / 6	Saya bersedia untuk m	Ya	32	Tangerang	Perempuan	TIDAK	Tidak	Pegawai swasta	Rp1.200.000 - R	D3	50	157		Tidak	Tidak	Tidak	Ya, sering	Internet, Sosial M	Terkadang (>50%
0/2023 16:04:14	90	1 / 6	Saya bersedia untuk m	Ya	29	Jakarta	Laki - laki	TIDAK	Belum Nikah	Tranportasi Logi	Rp1.200.000 - R	D4/S1	70	174		Tidak Ada	Tidak Ada	belum menikah /	Ya, kadang - kad	Internet	Jarang (<50%)
1/2023 16:12:00	91	4 / 6		Ya	36	jakarta	Perempuan	TIDAK		dokter	Rp1.200.000 - R	D4/S1	52	155		tiroid		Tidak	Ya, kadang - kad	Sosial Media	Jarang (<50%)
7/2023 18:42:15	92	2 / 6	Saya bersedia untuk m	Ya	27	Wonosobo	Perempuan	BELUM MENIKAH		Pelayanan Tekni	Rp532.000 – Rp	D4/S1	68	167		Tidak ada	Tidak ada	belum menikah /	Ya, kadang - kad	Internet, Sosial M	Terkadang (>50%
7/2023 19:04:40	93	2 / 6	Saya bersedia untuk m	Ya	28	Bandar Lampung	Perempuan	TIDAK	Tidak	Ibu rumah tangg	< Rp. 354.000.0	D4/S1	52	161		Tidak	Tidak	Tidak	Ya, kadang - kad	Teman dan keral	Terkadang (>50%
0/2023 10:31:31	94	1 / 6	Saya bersedia untuk m	Ya	44	Jakarta	Laki - laki	YA	Kondom	PNS	Rp1.200.000 - R	S2	58	167		Tidak ada	Tidak ada	Tidak	Ya, kadang - kad	Internet	Jarang (<50%)
0/2023 10:31:46	95	2 / 6	Saya bersedia untuk m	Ya	44	Jakarta	Perempuan	TIDAK		Wirausaha	Rp1.200.000 - R	D4/S1	84	163		Hnp	Tidak	Tidak	Tidak	Lainnya	Tidak Pernah
0/2023 17:22:01	96	2 / 6	Saya bersedia untuk m	Ya	35	Jakarta	Perempuan	TIDAK		ASN	> Rp. 6.000.000	D4/S1	72	165		Tisak	Tidak	Ya	Ya, kadang - kad	Internet, Televisi	Jarang (<50%)
11/2023 0:27:48	97	2 / 6	Saya bersedia untuk m	Ya	26	Jakarta	Perempuan	BELUM MENIKAH		Karyawan swast	> Rp. 6.000.000	D4/S1	55	162				belum menikah /	Ya, sering	Internet, Buku, S	Jarang (<50%)
6/2023 19:47:26	98	2 / 6	Saya bersedia untuk m	Ya	25	Bogor	Perempuan	TIDAK		Mahasiswa	Rp1.200.000 - R	S2	65	171		Anemia defisiensi Fe			Ya, Sering	Internet, Buku, T	Terkadang (>50%
0/2023 22:20:54	99	2 / 6	Saya bersedia untuk m	Ya	42	Bogor	Laki - laki	TIDAK		Driver online	Rp1.200.000 - R	D3	75	168		Tiak	Tidak	Ya	Ya, kadang - kad	Internet, Sosial M	Terkadang (>50%
6/2023 21:36:25	100	3 / 6	Saya bersedia untuk m	Ya	23	Jakarta	Laki - laki	TIDAK		Mahasiswa/Pela	Rp1.200.000 - R	D4/S1	67	178				belum menikah /	Ya, kadang - kad	Internet, Televisi	Jarang (<50%)
9/2023 12:47:25	101	1 / 6	Saya bersedia untuk m	Ya	35	Gunungkidul	Perempuan	TIDAK		Pns	> Rp. 6.000.000	D3	54	158				Ya	Tidak	Lainnya	Tidak Pernah
9/2023 15:05:02	102	1 / 6	Saya bersedia untuk m	Ya	27	Jakarta	Perempuan	TIDAK		Dokter Umun	Rp1.200.000 - R	S2	66	152				belum menikah /	Ya, kadang - kad	Sosial Media	Tidak Pernah
8/2023 21:09:32	103	1 / 6	Saya bersedia untuk m	Ya	29	Jakarta	Perempuan	TIDAK		Baru selesai kuli	> Rp. 6.000.000	S2	55 kg	164 cm				Ya	Ya, kadang - kad	Internet, Sosial M	Jarang (<50%)
6/2023 18:52:06	104	2 / 6	Saya bersedia untuk m	Ya	24	Pekanbaru	Perempuan	TIDAK		Quality Assuranc	Rp1.200.000 - R	D4/S1	50	159		Tidak	Tidak		Ya, Sering	Internet, Televisi	Jarang (<50%)
1/2023 18:46:31	105	2 / 6	Saya bersedia untuk m	Ya	26	Jakarta	Laki - laki	TIDAK		Driver Ojek Onlir	Rp532.000 – Rp	D4/S1	70	165		Tidak	Tidak	belum menikah /	Ya, sering	Sosial Media	Jarang (<50%)
6/2023 14:45:17	106	2 / 6	Saya bersedia untuk m	Ya	21	Tasikmalaya	Perempuan	YA	KB Suntik 3 Bula	Ibu Rumah Tang	Rp532.000 – Rp	SMA/SMU	78 Kg	165 Cm				Tidak	Ya, kadang - kad	Sosial Media	Terkadang (>50%
2/6/2023 7:56:49	107	1 / 6	Saya bersedia untuk m	Ya	38	Lhokseumawe	Laki - laki	TIDAK		Dokter	> Rp. 6.000.000	S2	69	168		Asam urat (obat	Alhamdulillaah ti	Tidak	Tidak	Internet	Tidak Pernah
20/2023 8:50:28	108	1 / 6	Saya bersedia untuk m	Ya	33	Bogor	Perempuan	YA	Inplan	Karyawan swast	Rp1.200.000 - R	SMA/SMU	60	158		Tidak	Tidak	Tidak	Ya, sering	Sosial Media	Terkadang (>50%
5/2023 15:26:37	109	1 / 6	Saya bersedia untuk m	Ya	26	Indramayy	Perempuan	TIDAK		Tenaga pendidik	Rp1.200.000 - R	D4/S1	49	145		Tidak ada	Tidak ada		Ya, Sering	Internet, Sosial M	Terkadang (>50%
12/2023 9:49:08	110	1 / 6	Saya bersedia untuk m	Ya	30	Pearaja Simasor	Perempuan	TIDAK		Jobseeker	Rp1.200.000 - R	D4/S1	63	159		Tidak	Tidak		Ya, Kadang - kad	Internet, Televisi	Jarang (<50%)
4/2023 22:51:34	111	2 / 6	Saya bersedia untuk m	Ya	53	Jakarta	Perempuan	TIDAK		Buruh	Rp532.000 – Rp	SMP/MTS	75	150 cm		Tidak			Tidak	Sosial Media	Tidak Pernah
16/2023 6:00:11	112	2 / 6	Saya bersedia untuk m	Ya	23	Jakarta	Laki - laki	TIDAK		Mahasiswa	Rp532.000 – Rp	SMA/SMU	58	171		Tidak	Tidak		Ya, Kadang - kad	Internet, Sosial M	Jarang (<50%)
6/2023 14:24:49	113	1 / 6	Saya bersedia untuk m	Ya	22	Jepara	Laki - laki	TIDAK	-	Mahasiswa	< Rp. 354.000.0	D4/S1	56	170		Tidak	Tidak		Ya, Kadang - kad	Internet	Jarang (<50%)
1/2023 16:10:30	114	2 / 6	Saya bersedia untuk m	Ya	38	Lampung selatan	Laki - laki	TIDAK	None	Travel consultan	> Rp. 6.000.000	SMA/SMU	68	168		None	None		Ya, sering	Internet, Sosial M	Terkadang (>50%
5/2023 15:40:09	115	2 / 6	Saya bersedia untuk m	Ya	31	CIMAHI	Perempuan	TIDAK		IRaT	Rp1.200.000 - R	D4/S1	53	156		Tidak	Tidak	Ya	Ya, kadang - kad	Internet	Jarang (<50%)
1/2023 19:13:12	116	5 / 6	Saya bersedia untuk m	Ya	27	Jakarta	Perempuan	TIDAK		Mahasiswa	Rp1.200.000 - R	D4/S1	56	160				belum menikah /	Ya, kadang - kad	Internet	Jarang (<50%)
1/2023 19:31:48	117	2 / 6	Saya bersedia untuk m	Ya	29	Jakarta	Perempuan	TIDAK		Residen	Rp1.200.000 - R	D4/S1	52	154				Ya	Ya, kadang - kad	Internet	Tidak Pernah
1/2023 19:35:13	118	1 / 6	Saya bersedia untuk m	Ya	29	jakarta	Perempuan	TIDAK		dokter	Rp532.000 – Rp	D4/S1	56	159		tidak	tidak	belum menikah /	Tidak	Lainnya	Tidak Pernah
8/2023 10:14:15	119	2 / 6	Saya bersedia untuk m	Ya	27	Jakarta	Perempuan	BELUM MENIKAH		Karyawan	> Rp. 6.000.000	D4/S1	52	160				belum menikah /	Ya, kadang - kad	Internet, Sosial M	Jarang (<50%)
8/2023 16:26:33	120	2 / 6	Saya bersedia untuk m	Ya	34	Dompu	Laki - laki	TIDAK		Karyawan swast	> Rp. 6.000.000	D4/S1	79	180		Tidak	Tidak	Tidak	Ya, sering	Internet, Sosial M	Jarang (<50%)
0/2023 22:20:36	121	2 / 6	Saya bersedia untuk m	Ya	34	Jakarta	Laki - laki	BELUM MENIKAH		Wirausaha	Rp. 354.000.00 -	SMA/SMU	150	170		Hipertensi	Tidak	belum menikah /	Ya, sering	Internet, Buku	Terkadang (>50%
3/2023 23:15:25	122	2 / 6	Saya bersedia untuk m	Ya	17	balikpapan	Perempuan	TIDAK		Mahasiswa	Rp532.000 – Rp	D4/S1	44kg	157kg		sinusitis	-	Tidak	Ya, sering	Teman dan keral	Terkadang (>50%
9/2023 15:12:52	123	2 / 6	Saya bersedia untuk m	Ya	26	Jakarta	Perempuan	TIDAK	-	Dokter manajem	> Rp. 6.000.000	D4/S1	57	167		Tidak	Tidak	Tidak	Ya, kadang - kad	Teman dan keral	Jarang (<50%)
8/2023 17:39:24	124	2 / 6	Saya bersedia untuk m	Ya	27	Tasikmalaya	Perempuan	TIDAK		Karyawan Swast	Rp1.200.000 - R	D4/S1	62	163		Tidak	Tidak	Tidak	Ya, kadang - kad	Internet, Sosial M	Jarang (<50%)
2/9/2023 9:31:21	125	3 / 6	Saya bersedia untuk m	Ya	21	Sragen	Perempuan	BELUM MENIKAH		Mahasiswa	Rp532.000 – Rp	SMA/SMU	66	156				belum menikah /	Ya, sering	Teman dan keral	Terkadang (>50%
0/2023 13:06:24	126	2 / 6	Saya bersedia untuk m	Ya	68	Madiun	Perempuan	TIDAK		Ibu rumah tangg	Rp1.200.000 - R	SD/MI	50 kg	150cm				Tidak	Tidak	Lainnya	Tidak Pernah
1/2023 11:06:44	127	2 / 6	Saya bersedia untuk m	Ya	28	Jakarta	Laki - laki	TIDAK		Karyawan Swast	Rp532.000 – Rp	S2	58	170					Ya, Sering	Sosial Media	Jarang (<50%)
4/2023 11:26:54	128	1 / 6	Saya bersedia untuk m	Ya	49	Cilacap	Laki - laki	TIDAK		Barber man	Rp1.200.000 - R	D4/S1	73	168					Ya, Sering	Internet, Radio,	Jarang (<50%)
5/2023 18:50:13	129	1 / 6	Saya bersedia untuk m	Ya	38	Jakarta	Perempuan	TIDAK		ASN	> Rp. 6.000.000	D4/S1	44	155		Tidak	Tidak	belum menikah /	Ya, sering	Internet, Lainnya	Tidak Pernah
6/2023 16:44:16	130	2 / 6	Saya bersedia untuk m	Ya	27	Karanganyar	Perempuan	YA	Implan	Freelance	Rp532.000 – Rp	D4/S1	69	155		tidak	Tidak	Tidak	Ya, sering	Internet, Sosial M	Terkadang (>50%
6/2023 22:53:27	131	2 / 6	Saya bersedia untuk m	Ya	20	Jakarta	Laki - laki	TIDAK		Freelance	< Rp. 354.000.0	SMA/SMU	52	167				belum menikah /	Ya, sering	Internet	Terkadang (>50%
18/2023 8:22:39	132	2 / 6	Saya bersedia untuk m	Ya	25	Subang	Perempuan	TIDAK		Perawat	Rp1.200.000 - R	D4/S1	53	159		Tidak ada	Tidak ada	belum menikah /	Ya, sering	Internet, Sosial M	Terkadang (>50%
1/2023 15:47:08	133	4 / 6	Saya bersedia untuk m	Ya	32	bekasi	Perempuan	TIDAK		dokter	> Rp. 6.000.000	D4/S1	60	161				Tidak	Tidak	Lainnya	Tidak Pernah
1/8/2023 0:17:20	134	3 / 6	Saya bersedia untuk m	Ya	27	Palembang	Perempuan	YA	IUD	Dokter	Rp1.200.000 - R	D4/S1	42	156		Tidak	Tidak	belum menikah /	Ya, sering	Internet, Sosial M	Jarang (<50%)
3/2023 10:36:13	135	2 / 6	Saya bersedia untuk m	Ya	28	Bogor	Perempuan	BELUM MENIKAH		Dokter Umum	> Rp. 6.000.000	D4/S1	47	148		tidak	tidak	belum menikah /	Ya, sering	Teman dan keral	Jarang (<50%)
5/2023 12:44:38	136	2 / 6	Saya bersedia untuk m	Ya	29	Kotim	Laki - laki	TIDAK		Dokter	Rp1.200.000 - R	D4/S1	80	163				Ya	Ya, kadang - kad	Buku, Sosial Me	Tidak Pernah
0/2023 23:13:20	137	2 / 6	Saya bersedia untuk m	Ya	22	Malang	Perempuan	TIDAK		Mahasiswa	Rp1.200.000 - R	D4/S1	48 kg	158 cm		asma	-	belum menikah /	Ya, sering	Internet	Terkadang (>50%
4/2023 10:42:04	138	5 / 6	Saya bersedia untuk m	Ya	21	Madiun	Perempuan	TIDAK	-	Mahasiswa	Rp532.000 – Rp	D4/S1	42	152		Tidak	Tidak		Tidak	Sosial Media	Tidak Pernah
7/2023 21:28:30	139	3 / 6	Saya bersedia untuk m	Ya	33	Surakarta	Laki - laki	TIDAK		PNS	Rp1.200.000 - R	S2	40	160					Ya, Kadang - kad	Internet, Televisi	Terkadang (>50%
1/2023 21:39:19	140	1 / 6	Saya bersedia untuk m	Ya	30	Batam	Perempuan	TIDAK		PPDS	Rp1.200.000 - R	D4/S1	62	165		tidak ada	tidak ada	belum menikah /	Ya, kadang - kad	Internet	Jarang (<50%)
2/8/2023 7:44:37	141	3 / 6	Saya bersedia untuk m	Ya	50	Jakarta	Laki - laki	TIDAK		Swasta	> Rp. 6.000.000	D4/S1	70	173				Tidak	Ya, sering	Internet, Sosial M	Terkadang (>50%
4/2023 17:13:32	142	1 / 6	Saya bersedia untuk m	Ya	36	Padang	Perempuan	TIDAK		Karyawan	Rp1.200.000 - R	SMA/SMU	70	160					Ya, Kadang - kad	Internet	Jarang (<50%)
5/2023 22:44:56	143	3 / 6	Saya bersedia untuk m	Ya	25	Jakarta	Laki - laki	YA	Sutra	Radiografer	< Rp. 354.000.0	D3	46	171		Tidak ada	Tidak ada		Ya, Sering	Sosial Media	Tidak Pernah
8/2023 18:10:25	144	2 / 6	Saya bersedia untuk m	Ya	27	Temanggung	Laki - laki	TIDAK		Guru	Rp. 354.000.00 -	D4/S1	60	170					Ya, Sering	Internet	Tidak Pernah
8/2023 18:38:02	145	0 / 6	Saya bersedia untuk m	Ya	28	Tuban	Laki - laki	TIDAK		Wiraswasta	Rp532.000 – Rp	SMA/SMU	55	163		Tidak ada	Tidak	belum menikah /	Ya, sering	Jurna lingkungan	Terkadang (>50%
15/2023 8:49:24	146	2 / 6	Saya bersedia untuk m	Ya	54	Jakarta	Perempuan	TIDAK		Swasta	Rp. 354.000.00 -	D4/S1	49	50		tidak	tidak		Ya, Kadang - kad	Sosial Media	Tidak Pernah
8/2023 18:06:38	147	2 / 6	Saya bersedia untuk m	Ya	30	Solo	Perempuan	TIDAK		Karyawan	> Rp. 6.000.000	D4/S1	65	165		Tidak	Tidak	belum menikah /	Ya, kadang - kad	Internet, Surat K	Terkadang (>50%
1/3/2023 4:56:32	148	2 / 6	Saya bersedia untuk m	Ya	29	jakarta	Perempuan	TIDAK		ppds	Rp532.000 – Rp	D4/S1	80	155				belum menikah /	Tidak	Lainnya	Tidak Pernah
8/2023 18:39:46	149	5 / 6	Saya bersedia untuk m	Ya	32	Gorontalo	Perempuan	BELUM MENIKAH		Dokter umum	Rp1.200.000 - R	D4/S1	76	164		Kolesterol	Tidak ada	belum menikah /	Ya, sering	Internet, Sosial M	Jarang (<50%)
7/2023 23:59:10	150	1 / 6		Ya	22	Malang	Perempuan	YA		Dokter muda/ Ko	> Rp. 6.000.000	D4/S1	57	162		rhinitis alergi		belum menikah /	Ya, kadang - kad	Internet, Sosial M	Tidak Pernah
0/2023 13:05:23	151	1 / 6	Saya bersedia untuk m	Ya	52	Jakarta	Perempuan	TIDAK		IRT	Rp1.200.000 - R	SMP/MTS	85	-		-	-	Tidak	Ya, kadang - kad	Lainnya	Tidak Pernah
0/2023 13:05:57	152	1 / 6	Saya bersedia untuk m	Ya	53	Jakarta	Perempuan	TIDAK		Ibu rumah tangg	Rp1.200.000 - R	SMA/SMU	70	150				Tidak	Tidak	Lainnya	Tidak Pernah
2/2023 18:46:16	153	3 / 6	Saya bersedia untuk m	Ya	22	Jakarta	Laki - laki	TIDAK		Mahasiswa	Rp532.000 – Rp	D4/S1	105	174					Ya, Kadang - kad	Teman dan keral	Terkadang (>50%
0/6/2023 8:54:35	154	0 / 6	Saya bersedia untuk m	Ya	40	Jakarta	Perempuan	TIDAK		Wiraswasta	Rp1.200.000 - R	SMA/SMU	90	160		Diabetes		Ya	Tidak	Lainnya	Terkadang (>50%
9/2023 23:54:51	155	1 / 6	Saya bersedia untuk m	Ya	25	Bogor	Perempuan	TIDAK		Dokter umum	> Rp. 6.000.000	D4/S1	48	148		-	-	Ya	Ya, kadang - kad	Internet, Sosial M	Tidak Pernah
0/2023 13:59:45	156	3 / 6	Saya bersedia untuk m	Ya	43	Gunungkidul	Perempuan	TIDAK		Ibu rumah tangg	Rp1.200.000 - R	SMA/SMU	55	150		Tidak	Tidak	Ya	Ya, kadang - kad	Sosial Media	Jarang (<50%)
11/2023 5:36:03	157	2 / 6	Saya bersedia untuk m	Ya	23	Yogyakarta	Laki - laki	TIDAK		Mahasiswa	Rp532.000 – Rp	SMA/SMU	52	169		Tidak	Tidak	Tidak	Ya, sering	Jurna lingkungan	Terkadang (>50%
1/2023 16:52:25	158	0 / 6	Saya bersedia untuk m	Ya	31	Surabaya	Perempuan	TIDAK		Dokter	Rp. 354.000.00 -	S2	50	156				belum menikah /	Tidak	Lainnya	Tidak Pernah
1/2023 20:58:04	159	1 / 6	Saya bersedia untuk m	Ya	29	Batam	Perempuan	TIDAK		Dokter	< Rp. 354.000.0	D4/S1	55 kg	155 cm		Tidak ada	Tidak ada	belum menikah /	Ya, kadang - kad	Internet, Jurna li	Jarang (<50%)
0/2023 22:07:26	160	1 / 6	Saya bersedia untuk m	Ya	27	Blora	Laki - laki	BELUM MENIKAH		Mahasiswa	Rp1.200.000 - R	D4/S1	69	162				belum menikah /	Ya, sering	Internet, Televisi	Jarang (<50%)
21/2023 5:36:10	161	1 / 6	Saya bersedia untuk m	Ya	47	kediri	Laki - laki	YA		Konsultan	Rp1.200.000 - R	SMA/SMU	80	155		tidak	tidak	Tidak	Tidak	Jurna lingkungan	Tidak Pernah
9/2023 15:05:05	162	1 / 6		Ya	26	Pandeglang	Perempuan	TIDAK	-	Dokter Umum	Rp1.200.000 - R	D4/S1	54	158		Tidak	Tidak	belum menikah /	Ya, kadang - kad	Teman dan keral	Tidak Pernah
9/2023 15:19:44	163	2 / 6	Saya bersedia untuk m	Ya	27	Medan	Laki - laki	TIDAK		Dokter Umum	> Rp. 6.000.000	D4/S1	67 kg	164 cm		-	-	Tidak	Ya, sering	Internet, Televisi	Jarang (<50%)
7/2023 23:59:01	164	4 / 6	Saya bersedia untuk m	Ya	29	Barru, Sulawesi	Perempuan	BELUM MENIKAH		Mahasiswa	Rp532.000 – Rp	D4/S1	72	158		tidak	tidak	belum menikah /	Ya, kadang - kad	Internet, Sosial M	Terkadang (>50%
0/2023 13:02:48	165	1 / 6	Saya bersedia untuk m	Ya	53	Jakarta	Perempuan	TIDAK		IRT	Rp. 354.000.00 -	SMP/MTS	60	160				Tidak	Ya, kadang - kad	Lainnya	Tidak Pernah
13/2023 2:49:43	166	4 / 6	Saya bersedia untuk m	Ya	26	Jakarta	Laki - laki	TIDAK		Freelance	> Rp. 6.000.000	D4/S1	76	183		tidak ada	tidak ada		Ya, sering	Internet, Jurna li	Terkadang (>50%
3/2023 23:06:16	167	2 / 6	Saya bersedia untuk m	Ya	29	Jakarta	Laki - laki	TIDAK		pedagang	Rp. 354.000.00 -	SMA/SMU	90 kg	168 cm		tidak ada	tidak ada		Ya, Kadang - kad	Internet	Jarang (<50%)
5/2023 19:08:02	168	2 / 6	Saya bersedia untuk m	Ya	40	Jakarta	Laki - laki	TIDAK		Pengangguran	< Rp. 354.000.0	D4/S1	106	183		Diabetes Melitus	Tidak ada.		Ya, Sering	Internet, Buku, B	Tidak Pernah
8/2023 17:17:35	169	1 / 6	Saya bersedia untuk m	Ya	35	Loang	Laki - laki	TIDAK		Swasta	Rp1.200.000 - R	S2	64	169		Tidak	Tidak		Ya, Sering	Lainnya	Terkadang (>50%
21/2023 9:30:09	170	1 / 6	Saya bersedia untuk m	Ya	32	Majalengka	Laki - laki	TIDAK		Pengangguran	Rp532.000 – Rp	SMA/SMU	110	172					Ya, Kadang - kad	Internet	Terkadang (>50%
6/2023 10:48:54	171	1 / 6	Saya bersedia untuk m	Ya	32	Pangkalpinang	Perempuan	YA		Karyawan Swast	Rp1.200.000 - R	D4/S1	75	168		Tidak ada	Tidak ada	belum menikah /	Ya, sering	Internet, Sosial M	Jarang (<50%)
20/2023 1:32:10	172	1 / 6	Saya bersedia untuk m	Ya	27	Jakarta	Laki - laki	YA	Implan	Serabutan	< Rp. 354.000.0	SMA/SMU	53	165		Tidak ada	Gak ada	Ya	Tidak	Sosial Media	Tidak Pernah
7/2023 14:15:35	173	4 / 6	Saya bersedia untuk m	Ya	26	Palembang	Perempuan	TIDAK		Dokter	> Rp. 6.000.000	D4/S1	58	156		tidak	tidak	Tidak	Ya, sering	Internet, Buku, S	Selau
6/2023 21:25:13	174	2 / 6		Ya	26	Koto Panjang	Perempuan	TIDAK		-	Rp532.000 – Rp	D3	161	58				belum menikah /	Ya, kadang - kad	Internet, Sosial M	Jarang (<50%)
6/2023 22:06:59	175	2 / 6	Saya bersedia untuk m	Ya	25	Batam	Perempuan	TIDAK	-	Farmasi	Rp1.200.000 - R	D3	50	160		Tidak	Tidak	Ya	Ya, kadang - kad	Sosial Media	Jarang (<50%)
17/2023 0:49:03	176	1 / 6	Saya bersedia untuk m	Ya	23	Jakarta	Perempuan	TIDAK	Tidak ada	Karyawan swast	> Rp. 6.000.000	SMA/SMU	40	158		Tidak	Tidak	belum menikah /	Tidak	Lainnya	Tidak Pernah
19/2023 0:19:53	177	2 / 6	Saya bersedia untuk m	Ya	25	Sukoharjo	Perempuan	TIDAK	-	Pegawai swasta	Rp1.200.000 - R	D3	67	158		Tidak ada	Tidak ada	belum menikah /	Ya, kadang - kad	Internet, Brosur I	Jarang (<50%)
5/2023 14:17:06	178	1 / 6	Saya bersedia untuk m	Ya	22	Jakarta	Laki - laki	TIDAK		Mahasiswa	Rp1.200.000 - R	D4/S1	70	174		-	-	belum menikah /	Ya, kadang - kad	Sosial Media	Tidak Pernah
26/2023 6:31:41	179	2 / 6	Saya bersedia untuk m	Ya	27	Jakarta	Laki - laki	TIDAK		Karyawan Swast	> Rp. 6.000.000	D4/S1	77	171		Tidak ada	Tidak ada	Tidak	Ya, kadang - kad	Internet, Brosur I	Tidak Pernah
2/1/2023 9:05:00	180	2 / 6	Saya bersedia untuk m	Ya	32	Surabaya	Perempuan	BELUM MENIKAH		Dosen	Rp1.200.000 - R	S2	51,5	154,5		-	-	belum menikah /	Ya, sering	Internet, Radio	Jarang (<50%)
5/2023 15:07:30	181	3 / 6	Saya bersedia untuk m	Ya	29	CIANJUR	Laki - laki	YA	KONDOM	KARYAWAN SW	> Rp. 6.000.000	D4/S1	81 KG	170 KG		TIDAK ADA	TIDAK ADA	Tidak	Ya, kadang - kad	Internet, Brosur I	Jarang (<50%)
5/2023 18:42:18	182	2 / 6	Saya bersedia untuk m	Ya	48	Jakarta	Laki - laki	YA	IUD	Dosen	> Rp. 6.000.000	S3	90	170		-	-	Tidak	Tidak	Lainnya	Tidak Pernah
8/2023 10:47:28	183	2 / 6	Saya bersedia untuk m	Ya	26	Malang	Perempuan	BELUM MENIKAH		Mahasiswa	Rp532.000 – Rp	D4/S1	49	158		-	-	belum menikah /	Ya, kadang - kad	Sosial Media	Jarang (<50%)
8/2023 12:23:40	184	2 / 6	Saya bersedia untuk m	Ya	25	Jakarta	Perempuan	BELUM MENIKAH		Guru Privat	< Rp. 354.000.0	SMA/SMU	55 kg	145 cm		Tidak	Tidak	belum menikah /	Ya, kadang - kad	Sosial Media	Jarang (<50%)
10/2024 2:47:37	185	2 / 6	Saya bersedia untuk m	Ya	23	Balikpapan	Perempuan	TIDAK		Mahasiswa	Rp1.200.000 - R	D4/S1	47	153,5 cm				belum menikah /	Ya, sering	Teman dan keral	Terkadang (>50%
15/2023 4:58:52	186	2 / 6	Saya bersedia untuk m	Ya	22	Bekasi	Laki - laki	TIDAK		Mahasiswa	< Rp. 354.000.0	D4/S1	60 kg	170 cm		Tidak ada	Tidak ada		Ya, Kadang - kad	Internet, Sosial M	Jarang (<50%)
6/2023 19:03:15	187	2 / 6	Saya bersedia untuk m	Ya	23	Bogor	Perempuan	TIDAK		Karyawan swast	Rp1.200.000 - R	D4/S1	54	160					Ya, Kadang - kad	Internet, Sosial M	Jarang (<50%)
11/2023 6:35:04	188	1 / 6	Saya bersedia untuk m	Ya	54	Tulungagung	Laki - laki	TIDAK		Wiraswasta	Rp1.200.000 - R	SMA/SMU	90	178		Hipertensi	Tidak	Ya	Tidak	Sosial Media	Tidak Pernah
1/2023 16:17:05	189	1 / 6	Saya bersedia untuk m	Ya	28	Bogor	Perempuan	TIDAK		PPDS	Rp1.200.000 - R	D4/S1	43	152				belum menikah /	Tidak	Lainnya	Tidak Pernah
9/2023 10:44:57	190	2 / 6	Saya bersedia untuk m	Ya	20	Bekasi	Perempuan	BELUM MENIKAH		-	< Rp. 354.000.0	SMA/SMU	41	161				belum menikah /	Ya, kadang - kad	Internet	Jarang (<50%)
9/2023 20:20:50	191	1 / 6	Saya bersedia untuk m	Ya	23	Depok	Laki - laki	BELUM MENIKAH		Mahasiswa	Rp1.200.000 - R	D4/S1	65	170		Tidak	Tidak	belum menikah /	Ya, kadang - kad	Internet, Buku, L	Jarang (<50%)

0/2023 21:16:52	192	3 / 6	Saya bersedia untuk m	Ya		23	Pasuruan	Perempuan	BELUM MENIKAH	-	Mahasiswi	Rp1.200.000 - R	S2	45	160	___	Tidak ada	Tidak ada	belum menikah /	Ya, sering	Internet, Sosial M	Terkadang (>50%
2/8/2023 6:25:46	193	3 / 6	Saya bersedia untuk m	Ya		28	Jakarta	Laki - laki	BELUM MENIKAH		Staff administras	Rp1.200.000 - R	D4/S1	68	166	___	Tidak	Tidak	belum menikah /	Ya, sering	Sosial Media	Terkadang (>50%
2/8/2023 9:30:28	194	1 / 6	Saya bersedia untuk m	Ya		27	Serang	Perempuan	BELUM MENIKAH		Swasta	Rp532.000 – Rp	S2	65	150	___	Tidak ada	Tidak ada	belum menikah /	Ya, sering	Internet, Jurna li	Jarang (<50%)
0/2023 10:31:52	195	2 / 6	Saya bersedia untuk m	Ya		27	Sengkang	Perempuan	BELUM MENIKAH		Guru	Rp532.000 – Rp	D4/S1	42	150	___	-	-	belum menikah /	Ya, kadang - kad	Teman dan keral	Jarang (<50%)
7/2023 23:19:27	196	1 / 6		Ya		54	Pati	Perempuan	TIDAK		Wiraswasta	> Rp. 6.000.000	D4/S1	68 kg	160 cm	___	Jantung bocor	Tidak		Ya, Kadang - kad	Televisi	Tidak Pernah
0/6/2023 6:28:34	197	2 / 6	Saya bersedia untuk m	Ya		25	Bogor	Perempuan	YA	Kb 3 bulan suntil	Ibu rumah tangg	Rp1.200.000 - R	SMA/SMU	51	160	___	Tidak	Tidak	Tidak	Ya, sering	Internet, Sosial M	Terkadang (>50%
9/2023 12:05:25	198	3 / 6	Saya bersedia untuk m	Ya		28	Depok	Laki - laki	TIDAK		Pegawai Swasta	> Rp. 6.000.000	D4/S1	78	160	___	tidak	tidak	belum menikah /	Ya, sering	Internet, Buku, S	Terkadang (>50%
9/2023 19:33:06	199	2 / 6	Saya bersedia untuk m	Ya		26	bekasi	Perempuan	YA	kondom	irt	Rp1.200.000 - R	SMA/SMU	45	165	___	kurang duit		Tidak	Tidak	Lainnya	Terkadang (>50%
6/2023 11:10:16	200	1 / 6	Saya bersedia untuk m	Ya		24	Pemalang, Jawa	Perempuan	TIDAK		Petugas Ambula	Rp1.200.000 - R	D4/S1	57	158	___	Tidak	Tidak	belum menikah /	Ya, kadang - kad	Internet, Lainnya	Tidak Pernah
20/2023 2:08:13	201	1 / 6	Saya bersedia untuk m	Ya		30	Salatiga	Perempuan	YA	Suntik 1bulan	Karyawan swast	Rp1.200.000 - R	SMA/SMU	54	153	___	Tidak	Tidak	Ya	Tidak	Internet, Sosial M	Tidak Pernah
1/2023 23:06:55	202	2 / 6	Saya bersedia untuk m	Ya		36	jakarta	Perempuan	YA	IUD	dokter	> Rp. 6.000.000	D4/S1	65	165	___	-	-	Tidak	Tidak	Teman dan keral	Jarang (<50%)
8/2023 18:56:50	203	2 / 6	Saya bersedia untuk m	Ya		31	Duri	Perempuan	TIDAK		IRT	Rp1.200.000 - R	D4/S1	65	158	___	Tidak	Tidak	Tidak	Ya, sering	Internet, Radio, T	Terkadang (>50%
5/2023 17:50:57	204	4 / 6	Saya bersedia untuk m	Ya		34	Dumai	Perempuan	TIDAK		Dokter	Rp1.200.000 - R	S2	74	160	___	Tidak ada	Tidak ada	belum menikah /	Ya, sering	Internet, Sosial M	Terkadang (>50%
8/2023 16:32:53	205	2 / 6	Saya bersedia untuk m	Ya		23	Banyuwangi	Perempuan	BELUM MENIKAH	-	Mahasiswa	Rp532.000 – Rp	D4/S1	60	150	___	-	-	belum menikah /	Ya, kadang - kad	Internet, Televisi	Tidak Pernah
0/2023 11:21:39	206	3 / 6	Saya bersedia untuk m	Ya		34	Bandung	Perempuan	TIDAK		Karyawan swast	< Rp. 354.000.0	D4/S1	63	149	___	Tidak	Tidak	Ya	Ya, kadang - kad	Teman dan keral	Jarang (<50%)
0/2023 13:06:38	207	0 / 6	Saya bersedia untuk m	Ya		60	Jakarta	Perempuan	TIDAK		Ibu rumah tangg	Rp1.200.000 - R	SD/MI	53,5 kg	145 cm	___	asam urat	-	belum menikah /	Tidak	Lainnya	Tidak Pernah
0/2023 13:10:18	208	3 / 6	Saya bersedia untuk m	Ya		52	Jakarta	Perempuan	TIDAK		IRT	Rp532.000 – Rp	SMA/SMU	73 kg	151 cm	___	Asam Urat		belum menikah /	Tidak	Lainnya	Tidak Pernah
0/2023 17:20:23	209	2 / 6	Saya bersedia untuk m	Ya		27	Tulungagung	Perempuan	BELUM MENIKAH		Wiraswasta	Rp1.200.000 - R	D4/S1	58	156	___	Tidak	Tidak	belum menikah /	Ya, sering	Teman dan keral	Terkadang (>50%
28/2023 4:40:42	210	1 / 6	Saya bersedia untuk m	Ya		22	Bekasi	Perempuan	TIDAK		Mahasiswa	Rp532.000 – Rp	D4/S1	50	150	___	Tidak	Tidak		Ya, Kadang - kad	Internet, Buku, J	Terkadang (>50%
2/2023 18:57:31	211	2 / 6	Saya bersedia untuk m	Ya		24	garut	Laki - laki	TIDAK		Karyawan	Rp1.200.000 - R	D4/S1	50	157	___				Ya, Kadang - kad	Internet, Sosial M	Terkadang (>50%
7/2023 10:29:38	212	2 / 6	Saya bersedia untuk m	Ya		26	Cirebon	Perempuan	YA	Suntik	Ibu rumah tangg	> Rp. 6.000.000	D4/S1	50	154	___	Lambung	Tidak ada	Ya	Ya, sering	Sosial Media	Terkadang (>50%
11/2023 1:12:15	213	1 / 6	Saya bersedia untuk m	Ya		32	Jakarta	Perempuan	YA	Suntik	Mengurus rumah	Rp1.200.000 - R	SMA/SMU	52	162	___	Tidak ada	Tidak	Tidak	Tidak	Sosial Media	Tidak Pernah
1/2023 15:49:47	214	3 / 6	Saya bersedia untuk m	Ya		26	Jakarta	Laki - laki	TIDAK		Dokter Umum	> Rp. 6.000.000	S2	77	164	___			belum menikah /	Ya, kadang - kad	Internet, Sosial M	Jarang (<50%)
1/2023 19:22:10	215	2 / 6	Saya bersedia untuk m	Ya		31	Bukittinggi	Laki - laki	TIDAK		Dokter PPDS	Rp1.200.000 - R	S2	75 Kg	178 cm	___	Tidak	Tidak	belum menikah /	Ya, sering	Internet, Brosur	Jarang (<50%)
6/2023 17:58:50	216	1 / 6	Saya bersedia untuk m	Ya		22	Surabaya	Laki - laki	YA		Mahasiswa	Rp1.200.000 - R	D4/S1	65	167	___	tidak ada	tidak ada	belum menikah /	Tidak	Lainnya	Tidak Pernah
6/2023 20:11:51	217	0 / 6	Saya bersedia untuk m	Ya		21	Bandung	Perempuan	TIDAK		Mahasiswa Koas	Rp1.200.000 - R	D4/S1	79	167	___			belum menikah /	Tidak	Lainnya	Tidak Pernah
9/2023 22:45:28	218	2 / 6	Saya bersedia untuk m	Ya		30	Bogor	Perempuan	TIDAK		Dokter umum	Rp1.200.000 - R	S2	58	159	___	Tidak ada	Tidak ada	belum menikah /	Ya, kadang - kad	Internet	Tidak Pernah
8/2023 12:55:22	219	3 / 6	Saya bersedia untuk m	Ya		30	Jakarta	Perempuan	BELUM MENIKAH		Graphic designe	> Rp. 6.000.000	D4/S1	62	160	___	Sakit kulit (eksim	Tidak ada	belum menikah /	Ya, sering	Internet, Televisi	Terkadang (>50%
0/2023 23:25:25	220	2 / 6	Saya bersedia untuk m	Ya		29	Jakarta	Laki - laki	BELUM MENIKAH	Tidak	Pegawai Pemeri	Rp1.200.000 - R	SMA/SMU	80	170	___	Tidak ada	Tidak ada	belum menikah /	Ya, sering	Internet, Sosial M	Terkadang (>50%
17/2023 3:36:25	221	2 / 6	Saya bersedia untuk m	Ya		29	Cimahi	Laki - laki	TIDAK		Pegawai Swasta	> Rp. 6.000.000	D4/S1	80	163	___	-	-	belum menikah /	Ya, sering	Teman dan keral	Tidak Pernah
0/2023 17:40:21	222	3 / 6	Saya bersedia untuk m	Ya		21	Kota Bandung	Perempuan	TIDAK		Mahasiswa	Rp532.000 – Rp	D4/S1	59	155	___				Ya, Kadang - kad	Internet, Buku, T	Terkadang (>50%
7/2023 21:14:02	223	3 / 6	Saya bersedia untuk m	Ya		26	Pati	Laki - laki	TIDAK		Pegawai swasta	Rp1.200.000 - R	D4/S1	85	185	___		Asthma		Ya, Sering	Internet, Buku, J	Selau
8/2023 20:15:48	224	1 / 6	Saya bersedia untuk m	Ya		49	Banyumas	Laki - laki	TIDAK		Suwasta	Rp1.200.000 - R	SMA/SMU	50kg	160	___	Alhamdulillah tid	Alhamdulillah tidak		Ya, Sering	Internet, Brosur	Terkadang (>50%
0/2023 16:09:55	225	1 / 6	Saya bersedia untuk m	Ya		37	Jakarta	Perempuan	YA	Steril/ tubektomi	Ibu Rumah Tang	Rp1.200.000 - R	D3	62	155	___	tidak	Tidak	Tidak	Ya, kadang - kad	Internet	Tidak Pernah
1/2023 15:56:42	226	4 / 6	Saya bersedia untuk m	Ya		28	Pontianak	Laki - laki	TIDAK		Residen	Rp1.200.000 - R	D4/S1	65	165	___			Tidak	Ya, sering	Teman dan keral	Jarang (<50%)
1/2023 16:47:35	227	2 / 6	Saya bersedia untuk m	Ya		33	Balikpapan	Laki - laki	YA	IUD	Dokter	Rp1.200.000 - R	D4/S1	110	173	___	tidak ada	tidak ada	Tidak	Ya, sering	Teman dan keral	Jarang (<50%)
1/2023 19:15:35	228	3 / 6	Saya bersedia untuk m	Ya		31	Sumenep	Laki - laki	TIDAK	-	Mahasiswa	Rp1.200.000 - R	D4/S1	76	176	___	Tidak ada	Tidak ada	belum menikah /	Ya, kadang - kad	Teman dan keral	Tidak Pernah
2/8/2023 5:49:29	229	2 / 6	Saya bersedia untuk m	Ya		29	Ciamis	Laki - laki	BELUM MENIKAH		Freelance	Rp1.200.000 - R	D4/S1	50	165	___	Tidak Ada	Tidak Ada	belum menikah /	Ya, sering	Sosial Media	Terkadang (>50%
5/2023 20:33:51	230	2 / 6	Saya bersedia untuk m	Ya		60	Jakarta	Perempuan	TIDAK		Ibu Rumah Tang	Rp1.200.000 - R	D4/S1	53 kg	155 cm	___			Tidak	Ya, sering	Internet, Sosial M	Terkadang (>50%
23/2023 11:06:31	231	2 / 6	Saya bersedia untuk m	Ya		32	Dongkala	Perempuan	BELUM MENIKAH		Karyawan swast	Rp532.000 – Rp	D3	56	170	___	TDK	Tdk	belum menikah /	Ya, kadang - kad	Internet	Jarang (<50%)
7/2023 19:38:19	232	1 / 6	Saya bersedia untuk m	Ya		21	Labuapi Lombok	Perempuan	BELUM MENIKAH	-	Mahasiswa	< Rp. 354.000.0	D4/S1	39	160 an	___	Tidak ada	Tidak ada	belum menikah /	Ya, sering	Internet	Terkadang (>50%
8/2023 13:09:06	233	3 / 6	Saya bersedia untuk m	Ya		29	Jakarta	Perempuan	BELUM MENIKAH		Karyawan Swast	Rp1.200.000 - R	D3	52	162	___			belum menikah /	Ya, sering	Internet, Surat K	Jarang (<50%)
6/2023 21:18:39	234	2 / 6	Saya bersedia untuk m	Ya		23	Meunafa	Laki - laki	TIDAK		wiraswasta	Rp532.000 – Rp	SMA/SMU	64	173	___	tidak ada	tidak ada	Tidak	Ya, kadang - kad	Teman dan keral	Tidak Pernah
7/2023 23:02:33	235	2 / 6	Saya bersedia untuk m	Ya		23	Tangerang	Laki - laki	BELUM MENIKAH		Pegawai	Rp532.000 – Rp	D4/S1	65	162	___			belum menikah /	Ya, sering	Teman dan keral	Jarang (<50%)
8/2023 13:31:02	236	2 / 6	Saya bersedia untuk m	Ya		19	Lampung	Perempuan	BELUM MENIKAH		Mahasiswi & free	< Rp. 354.000.0	D4/S1	50	150	___	-	Mudah cemas	belum menikah /	Ya, kadang - kad	Internet, Sosial M	Tidak Pernah
1/2023 11:57:16	237	1 / 6	Saya bersedia untuk m	Ya		35	Batang	Perempuan	YA		Karyawan swast	Rp1.200.000 - R	D4/S1	75	167	___	Tidak	Tidak		Ya, Kadang - kad	Internet, Sosial M	Jarang (<50%)
14/2023 6:50:10	238	0 / 6	Saya bersedia untuk m	Ya		47	Jakarta	Perempuan	TIDAK		Ibu rumah tangg	Rp1.200.000 - R	SMA/SMU	72kg	159	___	Tidak	Tidak		Tidak	Sosial Media	Jarang (<50%)
6/2023 19:32:43	239	0 / 6	Saya bersedia untuk m	Ya		37	Cilacap	Laki - laki	TIDAK		Mandor proyek	Rp1.200.000 - R	SMA/SMU	70	152	___	Tidak ada	Kaki x		Ya, Kadang - kad	Internet, Televisi	Jarang (<50%)
18/2023 6:35:14	240	2 / 6	Saya bersedia untuk m	Ya		25	Sumedang	Laki - laki	TIDAK		Kaeyawan swas	Rp1.200.000 - R	SMA/SMU	67	170	___				Ya, Sering	Internet, Sosial M	Terkadang (>50%
6/2023 17:53:17	241	2 / 6	Saya bersedia untuk m	Ya		45	Jakarta	Laki - laki	TIDAK		Freelance	Rp532.000 – Rp	D3	57	173	___	Tidak	Tidak	Tidak	Ya, sering	Internet, Sosial M	Terkadang (>50%
6/2023 18:35:04	242	1 / 6	Saya bersedia untuk m	Ya		32	Yogyakarta	Perempuan	YA	Suntik	ibu rumah tangg	Rp1.200.000 - R	D3	85	156	___	Tidak	Tidak	Ya	Tidak	Lainnya	Jarang (<50%)
7/2023 18:31:09	243	2 / 6	Saya bersedia untuk m	Ya		29	Jakarta	Laki - laki	TIDAK		Karyawan Swast	Rp1.200.000 - R	D4/S1	125kg	169cm	___	Tidak	Tidak	belum menikah /	Tidak	Internet	Tidak Pernah
7/2023 20:53:04	244	1 / 6	Saya bersedia untuk m	Ya		27	Tangerang	Perempuan	YA	KB alami	Ibu Rumah Tang	Rp1.200.000 - R	SMA/SMU	55	163	___	Tidak ada	Tidak ada	Ya	Ya, kadang - kad	Sosial Media	Terkadang (>50%
0/9/2023 5:31:08	245	5 / 6	Saya bersedia untuk m	Ya		27	Tanjung Harapan	Perempuan	YA	KB suntik 3 bula	Karyawan swast	Rp1.200.000 - R	D4/S1	53	163	___	Anemia	Tidak	Ya	Ya, sering	Internet, Buku, B	Selau
0/9/2023 6:24:54	246	0 / 6	Saya bersedia untuk m	Ya		36	jakarta	Perempuan	TIDAK		ibu rumah tangg	Rp1.200.000 - R	SMA/SMU	61	160	___	tidak ada	tidak ada	Ya	Ya, kadang - kad	Internet, Televisi	Tidak Pernah
9/2023 17:50:09	247	2 / 6	Saya bersedia untuk m	Ya		31	Sragen	Perempuan	TIDAK		Wirausaha	Rp1.200.000 - R	SMA/SMU	50	160	___	Tidak ada	Tidak ada	belum menikah /	Ya, kadang - kad	Internet, Sosial M	Terkadang (>50%
10/2023 7:26:58	248	1 / 6	Saya bersedia untuk m	Ya		36	Lampung	Laki - laki	TIDAK		Buruh	Rp1.200.000 - R	SMA/SMU	62	165	___	Tidak	Tidak	belum menikah /	Tidak	Sosial Media	Tidak Pernah
0/2023 14:47:36	249	1 / 6	Saya bersedia untuk m	Ya		30	Jakarta	Laki - laki	TIDAK		Karyawan	Rp1.200.000 - R	SMA/SMU	64	168	___			Tidak	Ya, kadang - kad	Internet, Sosial M	Jarang (<50%)
9/2023 14:12:06	250	2 / 6	Saya bersedia untuk m	Ya		33	Pinang tinggi	Perempuan	YA	Suntik	Irt	Rp1.200.000 - R	SMA/SMU	52	153	___	Tidak	Tidak	Tidak	Ya, kadang - kad	Internet	Terkadang (>50%
0/2023 17:58:15	251	3 / 6	Saya bersedia untuk m	Ya		28	Salatiga	Perempuan	YA	IUD	IRT	< Rp. 354.000.0	SMA/SMU	60kg	159	___	Tidak	Tidak	belum menikah /	Ya, kadang - kad	Internet, Sosial M	Terkadang (>50%
0/2023 23:21:16	252	2 / 6	Saya bersedia untuk m	Ya		28	Indramayu	Perempuan	TIDAK		Karyawan Swast	Rp1.200.000 - R	SMA/SMU	80	165	___	Tidak Ada	Tidak Ada	belum menikah /	Ya, kadang - kad	Internet, Buku, J	Terkadang (>50%
1/2023 15:54:18	253	2 / 6	Saya bersedia untuk m	Ya		31	Jambi	Laki - laki	TIDAK		Domter	Rp1.200.000 - R	D4/S1	80	178	___			Ya	Ya, kadang - kad	Internet, Sosial M	Jarang (<50%)
1/2023 16:03:06	254	1 / 6	Saya bersedia untuk m	Ya		32	Jakarta	Perempuan	YA	IUD	Dokter	> Rp. 6.000.000	D4/S1	65	163	___	Tidak	Tidak	Tidak	Tidak	Lainnya	Tidak Pernah
1/2023 16:26:43	255	1 / 6	Saya bersedia untuk m	Ya		34	Bandung	Perempuan	YA	IUD	Dokter	> Rp. 6.000.000	D4/S1	54	153	___	Tidak ada	Tidak ada	belum menikah /	Tidak	Teman dan keral	Tidak Pernah
1/2023 16:27:26	256	2 / 6	Saya bersedia untuk m	Ya		34	Jakarta	Perempuan	TIDAK		PPDS	Rp1.200.000 - R	D4/S1	49 kg	160cm	___	Asma, dermatitis	Tidak ada	belum menikah /	Ya, sering	Internet	Terkadang (>50%
1/2023 16:50:25	257	2 / 6	Saya bersedia untuk m	Ya		36	Lanny Jaya-Pap	Laki - laki	TIDAK	-	Dokter	Rp532.000 – Rp	D4/S1	85 KG	165 cm	___	tidak ada	tidak	Tidak	Tidak	Lainnya	Tidak Pernah
1/2023 16:55:19	258	1 / 6	Saya bersedia untuk m	Ya		29	Kediri	Perempuan	TIDAK	-	Dokter	Rp1.200.000 - R	D4/S1	50	158	___	tidak	tidak	Ya	Tidak	Lainnya	Tidak Pernah
1/2023 16:56:51	259	3 / 6	Saya bersedia untuk m	Ya		30	Selong	Perempuan	TIDAK	-	Mahasiswa	Rp1.200.000 - R	D4/S1	48	160	___	Tidak ada	Tidak ada	belum menikah /	Ya, kadang - kad	Lainnya	Jarang (<50%)
1/2023 18:14:16	260	3 / 6	Saya bersedia untuk m	Ya		30	Pontianak	Laki - laki	TIDAK	-	Pelajar	Rp532.000 – Rp	D4/S1	80	172	___	Tidak ada	Tidak ada	Tidak	Tidak	Teman dan keral	Tidak Pernah
1/2023 18:32:09	261	2 / 6	Saya bersedia untuk m	Ya		28	Tulungagung	Perempuan	TIDAK	Tidak ada	Ppds	Rp1.200.000 - R	D4/S1	70	168	___	Tidak ada	Tidak ada	Tidak	Tidak	Lainnya	Tidak Pernah
1/2023 19:12:07	262	2 / 6	Saya bersedia untuk m	Ya		28	Bandung	Perempuan	TIDAK		Dokter	Rp1.200.000 - R	D4/S1	84	160	___	-	-	Tidak	Tidak	Internet	Jarang (<50%)
6/2023 17:42:04	263	2 / 6	Saya bersedia untuk m	Ya		22	Pontianak	Perempuan	TIDAK		Mahasiswa	Rp1.200.000 - R	D4/S1	53	162	___			belum menikah /	Ya, sering	Internet, Brosur	Tidak Pernah
8/2023 11:29:40	264	2 / 6	Saya bersedia untuk m	Ya		21	Jakarta	Perempuan	BELUM MENIKAH		Freelance	Rp532.000 – Rp	D3	75	163	___	Tidak	Tidak	belum menikah /	Ya, kadang - kad	Internet, Sosial M	Tidak Pernah
8/2023 17:55:54	265	3 / 6	Saya bersedia untuk m	Ya		34	Jakarta	Laki - laki	TIDAK		Dokter	> Rp. 6.000.000	S2	78	177	___			Tidak	Ya, kadang - kad	Teman dan keral	Tidak Pernah
9/2023 21:46:43	266	2 / 6	Saya bersedia untuk m	Ya		24	Jakarta	Perempuan	BELUM MENIKAH		Karyawan Swast	Rp1.200.000 - R	D4/S1	51	168	___	Tidak ada.	Tidak ada.	belum menikah /	Ya, kadang - kad	Internet	Tidak Pernah
9/2023 14:49:47	267	2 / 6	Saya bersedia untuk m	Ya		33	Jakarta	Perempuan	TIDAK		Dokter	> Rp. 6.000.000	D4/S1	95	160	___	Tidak	Tidak	Tidak	Ya, kadang - kad	Internet, Sosial M	Jarang (<50%)
7/2023 18:17:42	268	3 / 6	Saya bersedia untuk m	Ya		21	24	Laki - laki	BELUM MENIKAH		Mahasiswa	Rp532.000 – Rp	D4/S1	59	175	___	Tidak	Tidak	belum menikah /	Tidak	Sosial Media	Tidak Pernah
7/2023 18:57:57	269	2 / 6	Saya bersedia untuk m	Ya		21	Jakarta	Laki - laki	BELUM MENIKAH		Mahasiswa	< Rp. 354.000.0	SMA/SMU	50 kg	165 CM	___	-	-	belum menikah /	Ya, kadang - kad	Internet, Sosial M	Selau
2/8/2023 8:52:57	270	1 / 6	Saya bersedia untuk m	Ya		47	Jakarta	Laki - laki	TIDAK		Dokter gigi	> Rp. 6.000.000	S2	88 kg	175 cm	___	_	_	Tidak	Ya, kadang - kad	Internet, Radio	Jarang (<50%)
8/2023 12:28:45	271	2 / 6	Saya bersedia untuk m	Ya		23	Yogyakarta	Perempuan	TIDAK	Belum menikah	Tenaga kesehata	Rp1.200.000 - R	D4/S1	47	155	___	-	-	Tidak	Ya, sering	Internet, Buku, J	Jarang (<50%)
8/2023 12:49:36	272	2 / 6	Saya bersedia untuk m	Ya		42	surabaya	Perempuan	TIDAK		pegawai swasta	Rp1.200.000 - R	D3	70	165	___			Tidak	Ya, kadang - kad	Internet, Sosial M	Terkadang (>50%
8/2023 14:11:53	273	2 / 6	Saya bersedia untuk m	Ya		22	Karawang	Perempuan	BELUM MENIKAH		Pegawai PBJ D.	Rp1.200.000 - R	D4/S1	62	159	___	tidak	tidak	belum menikah /	Ya, sering	Internet, Sosial M	Terkadang (>50%
8/2023 18:24:16	274	2 / 6	Saya bersedia untuk m	Ya		32	Karawang	Laki - laki	TIDAK		Wiraswasta	Rp1.200.000 - R	D4/S1	75	173	___	Varikokel		Ya	Ya, kadang - kad	Sosial Media	Jarang (<50%)
10/2023 4:32:22	275	2 / 6	Saya bersedia untuk m	Ya		27	Bandung	Perempuan	TIDAK		Guru	Rp532.000 – Rp	D4/S1	75kg	158cm	___	Tidak	Tidak	Ya	Ya, kadang - kad	Internet, Sosial M	Jarang (<50%)
0/2023 10:40:34	276	2 / 6	Saya bersedia untuk m	Ya		35	Sidoarjo	Perempuan	TIDAK		Mengurus Ruma	> Rp. 6.000.000	D4/S1	62	155	___	tidak	tidak	Tidak	Ya, sering	Internet	Jarang (<50%)
0/2023 13:02:15	277	1 / 6	Saya bersedia untuk m	Ya		60	Jakarta	Laki - laki	TIDAK		Ibu Rumah Tang	> Rp. 6.000.000	SD/MI	80	150	___	Kaki pegal pegal	-	Ya	Ya, kadang - kad	Lainnya	Tidak Pernah
3/2023 19:10:21	278	3 / 6	Saya bersedia untuk m	Ya		46	Jakarta	Perempuan	BELUM MENIKAH		Freelancer	Rp1.200.000 - R	D4/S1	57 kg	158 cm	___	Tidak	Tidak	belum menikah /	Ya, sering	Teman dan keral	Jarang (<50%)
9/2023 22:55:03	279	1 / 6	Saya bersedia untuk m	Ya		31	Cianjur	Perempuan	TIDAK		Bidan	Rp1.200.000 - R	D3	51	158	___	Tidak	Tidak	Ya	Ya, kadang - kad	Internet	Tidak Pernah
2/2023 12:49:57	280	3 / 6	Saya bersedia untuk m	Ya		34	Flores Timur	Perempuan	TIDAK		Sedang Mencari	Rp532.000 – Rp	D4/S1	50	155	___	Tidak	Tidak		Ya, Kadang - kad	Sosial Media	Terkadang (>50%
7/2023 22:48:15	281	3 / 6	Saya bersedia untuk m	Ya		39	Jakarta	Perempuan	TIDAK		Freelancer	> Rp. 6.000.000	D4/S1	56	160	___	Anovulasi	Tidak		Ya, Sering	Internet	Selau
6/2023 18:16:40	282	1 / 6	Saya bersedia untuk m	Ya		67	Jojakarta	Perempuan	TIDAK		Pensiun	Rp1.200.000 - R	D4/S1	56 kg	157 cm	___	Tidak	Tidak		Tidak	Sosial Media	Tidak Pernah
0/6/2023 8:21:08	283	4 / 6	Saya bersedia untuk m	Ya		30	Purworejo	Perempuan	TIDAK		Ibu rumah tangg	Rp1.200.000 - R	SMA/SMU	78	165	___	Tidak	Tidak	Tidak	Ya, sering	Internet, Jurna li	Terkadang (>50%
7/2023 15:04:47	284	2 / 6	Saya bersedia untuk m	Ya		23	Brebes	Laki - laki	TIDAK		Buruh Pabrik	Rp532.000 – Rp	SMA/SMU	62	173	___	Urtikaria		Tidak	Tidak	Internet	Tidak Pernah
0/9/2023 2:28:35	285	2 / 6	Saya bersedia untuk m	Ya		24	Grobogan	Perempuan	TIDAK	-	Mahasiswa	Rp532.000 – Rp	D4/S1	40	150	___	Tidak	Tidak	belum menikah /	Ya, sering	Jurna lingkungan	Selau
0/9/2023 3:08:20	286	2 / 6	Saya bersedia untuk m	Ya		28	Bogor	Laki - laki	TIDAK		Karyawan swast	Rp1.200.000 - R	SMA/SMU	80	170	___			Tidak	Ya, kadang - kad	Internet, Brosur	Terkadang (>50%
9/2023 11:54:00	287	2 / 6	Saya bersedia untuk m	Ya		26	Jakarta	Perempuan	TIDAK		Dokter	Rp1.200.000 - R	D4/S1	58	157	___	tidak	tidak	belum menikah /	Ya, sering	Internet, Televisi	Tidak Pernah
1/2023 16:42:27	288	2 / 6	Saya bersedia untuk m	Ya		30	Wonosobo	Perempuan	TIDAK		Dokter	> Rp. 6.000.000	D4/S1	59	165	___	Tidak ada	Tidak ada	Tidak	Ya, kadang - kad	Internet	Tidak Pernah
1/2023 18:25:54	289	3 / 6	Saya bersedia untuk m	Ya		34	Ngawi	Perempuan	YA	IUD	Dokter	> Rp. 6.000.000	D4/S1	63	157	___	Tudaj	Tidak	Tidak	Ya, kadang - kad	Internet, Televisi	Jarang (<50%)
1/2023 19:09:06	290	3 / 6		Ya		30	Karanganyar	Laki - laki	TIDAK		PPDS	> Rp. 6.000.000	D4/S1	83	167	___	-	-	Tidak	Ya, sering	Internet	Terkadang (>50%
1/2/2023 4:52:59	291	1 / 6	Saya bersedia untuk m	Ya		30	Jakarta	Perempuan	TIDAK	-	-	Rp1.200.000 - R	D4/S1	57	157	___	-	-	belum menikah /	Tidak	Lainnya	Tidak Pernah
1/2/2023 9:21:39	292	1 / 6	Saya bersedia untuk m	Ya		28	surabaya	Perempuan	TIDAK		residen	> Rp. 6.000.000	D4/S1	56	165	___	SLE	tidak ada	belum menikah /	Ya, kadang - kad	Internet	Tidak Pernah
8/2023 14:44:13	293	2 / 6	Saya bersedia untuk m	Ya		32	Pemalang	Laki - laki	TIDAK		Pengajar	Rp1.200.000 - R	D4/S1	59	174	___	Tidak	Tidak	Tidak	Ya, sering	Internet, Buku, T	Jarang (<50%)
0/2023 10:28:08	294	1 / 6	Saya bersedia untuk m	Ya		65	Palembang	Laki - laki	TIDAK		Swasta	< Rp. 354.000.0	D3	61	65	___	Tidak	Tidak	Tidak	Tidak	Teman dan keral	Tidak Pernah
30/2023 8:00:03	295	2 / 6	Saya bersedia untuk m	Ya		27	Sidoarjo	Perempuan	TIDAK	-	Dokter Umum	Rp1.200.000 - R	S2	59	151	___	-	-	Ya	Ya, sering	Internet, Sosial M	Jarang (<50%)
6/2023 10:09:43	296	2 / 6	Saya bersedia untuk m	Ya		38	Bogor	Perempuan	TIDAK		PNS	> Rp. 6.000.000	S2	59	160	___	radang otak	tidak	Ya	Ya, sering	Internet, Buku, S	Jarang (<50%)
8/2023 16:52:04	297	2 / 6	Saya bersedia untuk m	Ya		26	Pekanbaru	Laki - laki	BELUM MENIKAH	-	Karyawan Swast	Rp1.200.000 - R	D4/S1	67	178	___	Tidak	Tidak	belum menikah /	Ya, kadang - kad	Internet, Buku	Jarang (<50%)
0/2023 12:53:57	298	2 / 6	Saya bersedia untuk m	Ya		46	Bogor	Perempuan	YA	Sterile perempua	IRt	Rp. 354.000.00 -	SMP/MTS	70	150	___	Tidak ada	Tidak ada	Ya	Ya, kadang - kad	Buku	Jarang (<50%)
0/2023 13:07:40	299	2 / 6	Saya bersedia untuk m	Ya		51	Jakarta	Perempuan	TIDAK		Irt	Rp532.000 – Rp	SMP/MTS	58	150	___	-	-	Tidak	Tidak	Lainnya	Tidak Pernah
0/2023 13:15:12	300	4 / 6	Saya bersedia untuk m	Ya		49	Sidoarjo	Perempuan	TIDAK		Ibu Rumah Tang	Rp1.200.000 - R	SMP/MTS	55	145	___			Tidak	Ya, kadang - kad	Teman dan keral	Tidak Pernah
0/2023 13:25:54	301	3 / 6	Saya bersedia untuk m	Ya		59	Jakarta	Perempuan	TIDAK		Dagang	< Rp. 354.000.0	SMA/SMU	59	147	___	Gula, kalium		belum menikah /	Tidak	Lainnya	Tidak Pernah
0/2024 17:17:42	302	4 / 6	Saya bersedia untuk m	Ya		30	Jakarta	Laki - laki	TIDAK		Dokter Residen	> Rp. 6.000.000	D4/S1	100	175	___			Tidak	Ya, sering	Internet	Jarang (<50%)
5/2023 19:47:49	303	2 / 6	Saya bersedia untuk m	Ya		23	Sleman	Laki - laki	TIDAK		Pengusaha	Rp1.200.000 - R	D4/S1	51	174	___	-	-		Ya, Sering	Internet, Buku, J	Terkadang (>50%
14/2023 0:24:43	304	1 / 6	Saya bersedia untuk m	Ya		25	Tangerang	Laki - laki	TIDAK		Freelance	Rp532.000 – Rp	SMA/SMU	62	172	___	Tidak ada	Tidak ada		Ya, Kadang - kad	Internet, Sosial M	Jarang (<50%)
5/2023 13:49:59	305	1 / 6	Saya bersedia untuk m	Ya		47	Jakarta	Laki - laki	TIDAK		Wiraswasta	< Rp. 354.000.0	D4/S1	93	171	___	HIV	Tidak		Ya, Kadang - kad	Teman dan keral	Terkadang (>50%
5/2023 21:59:47	306	1 / 6	Saya bersedia untuk m	Ya		23	Jakarta	Laki - laki	TIDAK		Karyawan Swast	Rp532.000 – Rp	SMA/SMU	65	167	___	HIV	Tidak		Tidak	Sosial Media	Tidak Pernah
8/2023 14:06:02	307	2 / 6	Saya bersedia untuk m	Ya		30	Medan	Laki - laki	TIDAK		Serabutan	Rp1.200.000 - R	D4/S1	60	170	___			Tidak	Ya, sering	Internet, Brosur	Terkadang (>50%
0/9/2023 2:02:46	308	2 / 6	Saya bersedia untuk m	Ya		32	Purbalingga	Perempuan	YA	kondom	freelance	Rp1.200.000 - R	SMA/SMU	56	158	___	tidak ada	tidak ada	belum menikah /	Ya, sering	Internet, Sosial M	Terkadang (>50%
0/9/2023 7:10:59	309	1 / 6	Saya bersedia untuk m	Ya		46	Serui	Laki - laki	TIDAK		wartawan	Rp1.200.000 - R	D3	75	170	___	malaria	tidak	Tidak	Ya, kadang - kad	Internet, Buku, S	Terkadang (>50%
10/2023 9:42:06	310	1 / 6	Saya bersedia untuk m	Ya		29	Bogor	Laki - laki	TIDAK	Tidak	Kuli bangunan	Rp532.000 – Rp	SMA/SMU	64	170	___	Tidak	Tidak	belum menikah /	Tidak	Internet	Jarang (<50%)
11/2023 0:25:35	311	3 / 6	Saya bersedia untuk m	Ya		25	Jakarta	Perempuan	TIDAK		Beauty Advisor	Rp1.200.000 - R	D4/S1	60	158	___			Ya	Ya, sering	Teman dan keral	Tidak Pernah
1/2023 21:41:31	312	1 / 6	Saya bersedia untuk m	Ya		36	Jakarta	Perempuan	YA	Spiral	Ibu rumah tangg	Rp1.200.000 - R	SMA/SMU	67	160	___	Tidak	Tidak	Ya	Ya, sering	Internet, Sosial M	Jarang (<50%)
21/2023 11:33:13	313	1 / 6	Saya bersedia untuk m	Ya		29	Bogor	Laki - laki	TIDAK		Tidak bekerja	Rp532.000 – Rp	SMA/SMU	45	165	___	Tidak ada	Tidak ada	belum menikah /	Ya, kadang - kad	Internet	Jarang (<50%)
9/2023 16:26:22	314	2 / 6	Saya bersedia untuk m	Ya		27	Ujung Pandang	Perempuan	BELUM MENIKAH		Dokter	Rp1.200.000 - R	D4/S1	76	159	___			belum menikah /	Tidak	Lainnya	Jarang (<50%)
5/2023 23:44:53	315	4 / 6	Saya bersedia untuk m	Ya		24	Pontianak	Perempuan	BELUM MENIKAH		Swasta	Rp1.200.000 - R	S3	45	158	___	Tidak	Tidak	belum menikah /	Ya, sering	Internet, Jurna li	Selau
0/2023 13:15:11	316	1 / 6	Saya bersedia untuk m	Ya		53	Jakarta	Perempuan	BELUM MENIKAH		Ibu rumah tangg	Rp532.000 – Rp	SD/MI	60	150	___			belum menikah /	Tidak	Lainnya	Tidak Pernah
0/2023 13:15:22	317	1 / 6	Saya bersedia untuk m	Ya		50	Jakarta	Perempuan	YA	Suntik 3 bulan se	Ibu rumah tangg	Rp532.000 – Rp	SD/MI	55	150	___			Tidak	Tidak	Lainnya	Tidak Pernah
5/2024 11:42:43	318	3 / 6	Saya bersedia untuk m	Ya		23	Bandung	Perempuan	BELUM MENIKAH		Mahasiswa	> Rp. 6.000.000	SMA/SMU	50	160	___			belum menikah /	Ya, sering	Teman dan keral	Terkadang (>50%
6/2023 23:57:46	319	1 / 6	Saya bersedia untuk m	Ya		25	Bogor	Perempuan	TIDAK		Karyawan swast	Rp1.200.000 - R	D4/S1	57	165	___	Tidak	Tidak		Ya, Sering	Internet, Sosial M	Jarang (<50%)
1/2023 20:04:34	320	0 / 6	Saya bersedia untuk m	Ya		23	Seram	Perempuan	TIDAK	-	Mahasiswa	< Rp. 354.000.0	SMA/SMU	48 kg	159 cmn	___	Tidak	Tidak		Ya, Kadang - kad	Internet, Sosial M	Selau
2/2023 10:58:30	321	1 / 6	Saya bersedia untuk m	Ya		52	Tarutung	Perempuan	TIDAK		Wiraswasta	Rp1.200.000 - R	D4/S1	61	153	___	Tidak	Tidak		Ya, Sering	Sosial Media	Terkadang (>50%
2/2023 18:47:58	322	1 / 6	Saya bersedia untuk m	Ya		22	Bogor	Laki - laki	TIDAK	Tidak	Karyawan Kontra	Rp. 354.000.00 -	SMA/SMU	59	170	___	Tidak	Tidak		Ya, Kadang - kad	Internet	Terkadang (>50%
4/2023 23:47:14	323	3 / 6	Saya bersedia untuk m	Ya		47	Jakarta	Perempuan	TIDAK		Guru	Rp1.200.000 - R	D4/S1	80	165	___	Tidak	Tidak		Ya, Kadang - kad	Sosial Media	Jarang (<50%)
8/2023 22:34:52	324	1 / 6	Saya bersedia untuk m	Ya		22	Bogor	Perempuan	TIDAK	Tidak	Pedagang	< Rp. 354.000.0	SMA/SMU	40	150	___	Tidak	Tidak		Ya, Kadang - kad	Internet	Jarang (<50%)
19/2023 7:01:28	325	2 / 6	Saya bersedia untuk m	Ya		29	depok	Perempuan	TIDAK		ibu rumah tangg	Rp532.000 – Rp	SMA/SMU	75	165	___	tidk	tidak		Ya, Sering	Internet, Sosial M	Terkadang (>50%
8/2023 15:06:00	326	2 / 6	Saya bersedia untuk m	Ya		38	Sukabumi	Laki - laki	YA	Andalan	Swasta	Rp1.200.000 - R	SMA/SMU	75	168	___	Tidak	Tidak		Ya, sering	Sosial Media	Jarang (<50%)
1/2023 14:28:16	327	4 / 6	Saya bersedia untuk m	Ya		27	Tanjung selor	Laki - laki	TIDAK		Sektor kesehata	Rp1.200.000 - R	D4/S1	98	163	___	Tidak	Tidak		Ya, kadang - kad	Internet	Jarang (<50%)
2/2023 11:01:28	328	3 / 6	Saya bersedia untuk m	Ya		27	Jakarta	Perempuan	TIDAK	-	Swasta	Rp532.000 – Rp	SMA/SMU	59	158	___				Ya, kadang - kad	Internet	Tidak Pernah
6/2023 23:32:44	329	2 / 6	Saya bersedia untuk m	Ya		45	BANYUWANGI	Laki - laki	TIDAK		Wiraswasta	< Rp. 354.000.0	SMA/SMU	75	178	___	Tidak ada	Tidak ada	Tidak	Ya, sering	Internet	Selau
0/7/2023 7:45:11	330	1 / 6	Saya bersedia untuk m	Ya		32	Cianjur	Perempuan	TIDAK		Ibu rumah tangg	Rp1.200.000 - R	SMA/SMU	80 kg	155 cm	___	Tidak	Tidak	Ya	Ya, kadang - kad	Internet, Sosial M	Tidak Pernah
7/2023 18:01:56	331	2 / 6	Saya bersedia untuk m	Ya		26	Wonogiri	Perempuan	YA	Implan	Ibu rumah tangg	Rp1.200.000 - R	SMA/SMU	55	160	___	Tidak ada	Tidak ada	belum menikah /	Tidak	Sosial Media	Jarang (<50%)
7/2023 19:29:41	332	1 / 6	Saya bersedia untuk m	Ya		46	Jakarta	Laki - laki	TIDAK		Wiraswasta	Rp. 354.000.00 -	SMP/MTS	78	169	___	Tidak	Tidak	Tidak	Ya, kadang - kad	Internet, Televisi	Terkadang (>50%
7/2023 20:34:53	333	1 / 6	Saya bersedia untuk m	Ya		34	Ciamis	Perempuan	TIDAK		Irt	Rp1.200.000 - R	SMA/SMU	68	163	___			Tidak	Ya, kadang - kad	Internet	Selau
8/2023 14:18:34	334	3 / 6	Saya bersedia untuk m	Ya		29	Purworejo	Perempuan	YA	Kondom	Ibu Rumah Tang	Rp1.200.000 - R	D4/S1	60	157	___	Tidak ada	Tidak ada	Ya	Ya, sering	Internet, Sosial M	Terkadang (>50%
0/9/2023 2:30:14	335	2 / 6	Saya bersedia untuk m	Ya		33	Karawang	Perempuan	TIDAK		Ibu rumah tangg	Rp1.200.000 - R	SMA/SMU	80	164	___			Tidak	Ya, kadang - kad	Internet, Jurna li	Terkadang (>50%
9/2023 16:07:05	336	1 / 6	Saya bersedia untuk m	Ya		41	Bekasi	Laki - laki	TIDAK		Karyawan Swast	Rp1.200.000 - R	SMA/SMU	60kg	171	___	Tidak	Tidak	Tidak	Ya, kadang - kad	Internet, Lainnya	Tidak Pernah
0/2023 10:28:47	337	1 / 6	Saya bersedia untuk m	Ya		38	jakarta	Perempuan	YA	IUD	Dokter Umum PI	Rp1.200.000 - R	D4/S1	60	153	___	tidak ada	tidak ada	Tidak	Ya, kadang - kad	Internet, Sosial M	Jarang (<50%)
0/2023 10:29:17	338	0 / 6	Saya bersedia untuk m	Ya		27	Indramayu	Perempuan	TIDAK		IRT	Rp1.200.000 - R	D4/S1	41	152	___			Tidak	Ya, kadang - kad	Internet	Tidak Pernah
21/2023 3:02:07	339	1 / 6	Saya bersedia untuk m	Ya		41	Bandar Lampung	Laki - laki	TIDAK		Wirausaha	> Rp. 6.000.000	D4/S1	77	171	___	Hipertensi	Tidak	belum menikah /	Tidak	Internet	Tidak Pernah
21/2023 11:24:13	340	2 / 6	Saya bersedia untuk m	Ya		27	Jakarta	Perempuan	TIDAK		Driver ojol	< Rp. 354.000.0	SMA/SMU	53kg	160cm	___			belum menikah /	Tidak	Sosial Media	Jarang (<50%)
1/2023 15:52:18	341	4 / 6	Saya bersedia untuk m	Ya		35	Mataram	Perempuan	YA	IUD	PNS	Rp1.200.000 - R	D4/S1	58	149	___	Tidak ada	Tidak ada	belum menikah /	Ya, kadang - kad	Internet	Jarang (<50%)
1/2023 16:00:09	342	1 / 6	Saya bersedia untuk m	Ya		31	Medan	Laki - laki	TIDAK	Tidak ada	PPDS	< Rp. 354.000.0	D4/S1	87	174 cm	___	Tidak	Tidak	Tidak	Ya, kadang - kad	Internet	Jarang (<50%)
1/2023 16:20:42	343	2 / 6	Saya bersedia untuk m	Ya		26	Manggar	Perempuan	TIDAK		dokter	< Rp. 354.000.0	D4/S1	58	153	___	-	-	belum menikah /	Tidak	Lainnya	Tidak Pernah
2/2023 19:32:48	344	3 / 6	Saya bersedia untuk m	Ya		31	bandung	Perempuan	TIDAK	Tidak	Residen	> Rp. 6.000.000	D4/S1	62	155	___	Tidak ada	Tidak	belum menikah /	Tidak	Lainnya	Tidak Pernah
8/2023 21:23:00	345	1 / 6	Saya bersedia untuk m	Ya		25	Brebes	Perempuan	TIDAK	-	Ahli Gizi	Rp1.200.000 - R	D4/S1	53	158	___	tidak ada	tidak	Tidak	Ya, kadang - kad	Internet	Jarang (<50%)
28/2023 11:55:21	346	3 / 6	Saya bersedia untuk m	Ya		24	Bekasi	Perempuan	BELUM MENIKAH		Bidan	Rp1.200.000 - R	D3	55	156	___	-	-	belum menikah /	Ya, sering	Teman dan keral	Jarang (<50%)
5/2023 13:58:59	347	2 / 6	Saya bersedia untuk m	Ya		28	Jakarta	Laki - laki	TIDAK		PPDS	> Rp. 6.000.000	D4/S1	87	170	___	Tidak ada	Tidak ada	belum menikah /	Ya, kadang - kad	Internet	Tidak Pernah
0/2023 13:16:08	348	2 / 6	Saya bersedia untuk m	Ya		45	Jakarta	Perempuan	TIDAK		Ibu Rumah Tang	Rp532.000 – Rp	SMA/SMU	88	160	___	Tidak	Tidak	Ya	Ya, sering	Internet	Terkadang (>50%
11/2023 9:45:20	349	3 / 6	Saya bersedia untuk m	Ya		25	Jakarta	Perempuan	BELUM MENIKAH		Perawat	Rp1.200.000 - R	D4/S1	55	155	___			belum menikah /	Ya, sering	Lainnya	Jarang (<50%)

Menurut Anda,	Menurut Anda,	Menurut penda	Menurut penda	Apakah mikrop	Apakah Anda m	Menurut penda	Air minum dalam kemasan plas contoh: kemasan minuman kak		Makanan jadi d	Skor	Total	Air Minum Dalan	Air Minum isi ula	Seberapa serin	Seberapa serin	Seberapa serin	Seberapa serin	Seberapa serin	Seberapa serin	Seberapa serin	Seberapa serin	Seberapa serin
Serat, Partikel B	Kurang dari 1 m	Produksi industri	Air Tawar, Air La	Ya	Ya	Mulut (saluran c	Tidak pernah	0	Tidak pernah	0	0	>3x sehari	Tidak pernah	4-6x sepekan	Tidak pernah	Tidak pernah	Tidak pernah	Tidak pernah	Tidak pernah	>3x sehari	Tidak pernah	>3x sehari
Partikel Tidak Be	Kurang dari 1 m	Produksi industri	Udara, Tanah, B	Ya	Ya	Mulut (saluran c	Tidak pernah	0	Tidak pernah	0	0	Tidak pernah	>3x sehari	Tidak pernah	Tidak pernah	>3x sehari	1-3x sehari	4-6x sepekan	1-3x sepekan	Tidak pernah	Tidak pernah	Tidak pernah
Partikel tidak be	Kurang dari 1 m	Penggunaan pro	Air Laut	Ya	Tidak	Mulut (saluran c	Tidak pernah	0	Tidak pernah	0	0	Tidak pernah	Tidak pernah	1-3x sepekan	Tidak pernah	1-3x sepekan	1-3x sepekan	Tidak pernah	Tidak pernah	Tidak pernah	Tidak pernah	1-3x sebulan
Serat, Partikel b	Kurang dari 1 m	Penggunaan pro	Produk hewani s	Tidak tahu	Tidak	Hidung (Saluran	Tidak pernah	0	Tidak pernah	0	0	1-3x sehari	>3x sehari	Tidak pernah	Tidak pernah	Tidak pernah	Tidak pernah	Tidak pernah	Tidak pernah	Tidak pernah	Tidak pernah	Tidak pernah
Serat, Partikel b	Sekitar 1 - 5 cm	Produksi industri	Air Tawar, Air La	Ya	Ya	Mulut (saluran c	<1x sebulan	1	Tidak pernah	0	1	<1x sebulan	Tidak pernah	1-3x sebulan	Tidak pernah	1-3x sebulan	1-3x sebulan	Tidak pernah	Tidak pernah	Tidak pernah	Tidak pernah	Tidak pernah
Film	Kurang dari 1 m	Produksi industri	Air Tawar, Air La	Ya	Ya	Mulut (saluran c	1-3x sebulan	2	Tidak pernah	0	2	Tidak pernah	Tidak pernah	1-3x sepekan	Tidak pernah	<1x sebulan	<1x sebulan	Tidak pernah	<1x sebulan	<1x sebulan	Tidak pernah	Tidak pernah
Partikel tidak be	Kurang dari 1 m	Produksi industri	Air Tawar, Air La	Tidak tahu	Ya	Mulut (saluran c	1-3x sebulan	2	Tidak pernah	0	2	1-3x sebulan	Tidak pernah	<1x sebulan	<1x sebulan	<1x sebulan	<1x sebulan	Tidak pernah	Tidak pernah	1-3x sepekan	Tidak pernah	<1x sebulan
Serat, Partikel b	Kurang dari 1 m	Produksi industri	Air Tawar, Air La	Ya	Ya	Mulut (saluran c	1-3x sebulan	2	Tidak pernah	0	2	<1x sebulan	Tidak pernah	<1x sebulan	Tidak pernah	Tidak pernah	1-3x sebulan	Tidak pernah	Tidak pernah	1-3x sebulan	Tidak pernah	<1x sebulan
Serat	Kurang dari 1 m	Penggunaan pro	Air Laut, Biota L	Ya	Ya	Mulut (saluran c	<1x sebulan	1	<1x sebulan	1	2	1-3x sebulan	Tidak pernah	<1x sebulan	<1x sebulan	1-3x sebulan	<1x sebulan	Tidak pernah	<1x sebulan	1-3x sepekan	1-3x sebulan	1-3x sepekan
Serat, Partikel b	Kurang dari 1 m	Produksi industri	Air Tawar, Air La	Ya	Ya	Mulut (saluran c	<1x sebulan	1	<1x sebulan	1	2	4-6x sepekan	1-3x sehari	1-3x sebulan	Tidak pernah	Tidak pernah	1-3x sebulan	Tidak pernah	<1x sebulan	1-3x sebulan	Tidak pernah	1-3x sepekan
Film	Kurang dari 1 m	Penggunaan pro	Air Laut	Ya	Ya	Mulut (saluran c	<1x sebulan	1	<1x sebulan	1	2	>3x sehari	Tidak pernah	<1x sebulan	<1x sebulan	Tidak pernah	Tidak pernah	Tidak pernah	Tidak pernah	Tidak pernah	Tidak pernah	Tidak pernah
Serat	Kurang dari 1 m	Pencucian pakai	Air Tawar, Air La	Tidak tahu	Ya	Mulut (saluran c	<1x sebulan	1	<1x sebulan	1	2	>3x sehari	Tidak pernah	1-3x sebulan	1-3x sebulan	<1x sebulan	<1x sebulan	<1x sebulan	<1x sebulan	<1x sebulan	<1x sebulan	<1x sebulan
Serat, Partikel b	Kurang dari 1 m	Produksi industri	Air Tawar, Air La	Ya	Tidak	Mulut (saluran c	<1x sebulan	1	<1x sebulan	1	2	1-3x sepekan	Tidak pernah	<1x sebulan	<1x sebulan	<1x sebulan	<1x sebulan	<1x sebulan	<1x sebulan	<1x sebulan	<1x sebulan	1-3x sepekan
Serat, Film	Kurang dari 1 m	Produksi industri	Air Tawar, Air La	Ya	Ya	Mulut (saluran c	1-3x sepekan	3	Tidak pernah	0	3	<1x sebulan	1-3x sebulan	1-3x sebulan	1-3x sebulan	4-6x sepekan	<1x sebulan	1-3x sebulan	1-3x sebulan	<1x sebulan	1-3x sebulan	1-3x sebulan
Serat	Kurang dari 1 m	Produksi industri	Air Tawar, Air La	Tidak tahu	Tidak	Mulut (saluran c	1-3x sepekan	3	Tidak pernah	0	3	>3x sehari	Tidak pernah	Tidak pernah	Tidak pernah	4-6x sepekan	<1x sebulan	1-3x sebulan	4-6x sepekan	4-6x sepekan	1-3x sebulan	>3x sehari
Serat, Partikel ti	Kurang dari 1 m	Penggunaan pro	Air Laut	Ya	Ya	Mulut (saluran c	1-3x sepekan	3	Tidak pernah	0	3	1-3x sebulan	Tidak pernah	4-6x sepekan	<1x sebulan	1-3x sepekan	<1x sebulan	1-3x sehari	1-3x sepekan	>3x sehari	4-6x sepekan	>3x sehari
Serat	Kurang dari 1 m	Produksi industri	Air Tawar, Air La	Ya	Ya	Mulut (saluran c	1-3x sebulan	2	<1x sebulan	1	3	1-3x sebulan	1-3x sebulan	4-6x sepekan	1-3x sebulan	1-3x sebulan	4-6x sepekan	1-3x sepekan	4-6x sepekan	1-3x sepekan	1-3x sepekan	1-3x sebulan
Serat, Partikel B	Kurang dari 1 m	Produksi industri	Air Tawar, Air La	Tidak Tahu	Ya	Mulut (saluran c	1-3x sebulan	2	<1x sebulan	1	3	<1x sebulan	Tidak pernah	<1x sebulan	>3x sehari	<1x sebulan	<1x sebulan	<1x sebulan	<1x sebulan	<1x sebulan	<1x sebulan	<1x sebulan
Partikel tidak be	Kurang dari 1 m	Produksi industri	Air Tawar, Air La	Ya	Ya	Mulut (saluran c	1-3x sebulan	2	<1x sebulan	1	3	1-3x sebulan	<1x sebulan	1-3x sebulan	<1x sebulan	<1x sebulan	<1x sebulan	Tidak pernah	1-3x sebulan	1-3x sebulan	<1x sebulan	1-3x sebulan
Serat	Kurang dari 1 m	Pelepasan serat	Air Laut	Ya	Ya	Mulut (saluran c	1-3x sebulan	2	<1x sebulan	1	3	Tidak pernah	1-3x sebulan	1-3x sebulan	<1x sebulan	<1x sebulan	<1x sebulan	<1x sebulan	<1x sebulan	1-3x sebulan	1-3x sebulan	1-3x sebulan
Partikel bulat, Pa	Sekitar 1 - 5 cm	Produksi industri	Udara	Ya	Ya	Mulut (saluran c	1-3x sebulan	2	<1x sebulan	1	3	<1x sebulan	<1x sebulan	<1x sebulan	<1x sebulan	<1x sebulan	<1x sebulan	<1x sebulan	1-3x sebulan	1-3x sepekan	1-3x sebulan	1-3x sepekan
Serat	Kurang dari 1 m	Produksi industri	Air Laut, Tanah,	Ya	Ya	Hidung (Saluran	<1x sebulan	1	1-3x sebulan	2	3	Tidak pernah	Tidak pernah	1-3x sebulan	1-3x sebulan	1-3x sebulan	<1x sebulan	Tidak pernah	Tidak pernah	Tidak pernah	Tidak pernah	Tidak pernah
Serat, Partikel b	Kurang dari 1 m	Produksi industri	Air Tawar, Air La	Ya	Ya	Mulut (saluran c	<1x sebulan	1	1-3x sebulan	2	3	1-3x sepekan	1-3x sebulan	<1x sebulan	Tidak pernah	<1x sebulan	<1x sebulan	Tidak pernah	1-3x sebulan	>3x sehari	<1x sebulan	>3x sehari
Serat	Kurang dari 1 m	Produksi industri	Air Tawar, Air La	Ya	Ya	Mulut (saluran c	<1x sebulan	1	1-3x sebulan	2	3	1-3x sepekan	1-3x sepekan	<1x sebulan	<1x sebulan	<1x sebulan	1-3x sebulan	Tidak pernah	1-3x sebulan	1-3x sepekan	Tidak pernah	<1x sebulan
Film	Kurang dari 1 m	Produksi industri	Udara	Ya	Ya	Hidung (Saluran	<1x sebulan	1	1-3x sebulan	2	3	>3x sehari	>3x sehari	<1x sebulan	Tidak pernah	<1x sebulan	<1x sebulan	Tidak pernah	<1x sebulan	1-3x sepekan	Tidak pernah	1-3x sehari
Partikel bulat	Kurang dari 1 m	Penggunaan pro	Air Tawar, Air La	Ya	Tidak	Hidung (Saluran	<1x sebulan	1	1-3x sebulan	2	3	>3x sehari	Tidak pernah	Tidak pernah	4-6x sepekan	1-3x sehari	1-3x sepekan	Tidak pernah	1-3x sebulan	Tidak pernah	Tidak pernah	1-3x sebulan
Serat	Kurang dari 1 m	Pelepasan serat	Air Tawar	Tidak tahu	Tidak	Kulit	Tidak pernah	0	1-3x sepekan	3	3	1-3x sepekan	1-3x sehari	4-6x sepekan	4-6x sepekan	1-3x sepekan	1-3x sepekan	1-3x sebulan	1-3x sebulan	1-3x sepekan	Tidak pernah	<1x sebulan
Partikel tidak be	Kurang dari 1 m	Produksi industri	Air Tawar, Air La	Ya	Ya	Mulut (saluran c	Tidak pernah	0	1-3x sepekan	3	3	>3x sehari	1-3x sehari	1-3x sebulan	1-3x sebulan	1-3x sebulan	1-3x sepekan	1-3x sehari	Tidak pernah	4-6x sepekan	<1x sebulan	>3x sehari
Partikel tidak be	Sekitar 1 - 5 cm	Penggunaan pro	Air Laut	Ya	Tidak	Hidung (Saluran	4-6x sepekan	4	Tidak pernah	0	4	1-3x sepekan	>3x sehari	Tidak pernah	Tidak pernah	>3x sehari	1-3x sebulan	<1x sebulan	<1x sebulan	<1x sebulan	<1x sebulan	>3x sehari
Partikel Bulat	Kurang dari 1 m	Pencucian pakai	Air Tawar, Air La	Ya	Ya	Mulut (saluran c	1-3x sepekan	3	<1x sebulan	1	4	1-3x sepekan	Tidak pernah	Tidak pernah	<1x sebulan	Tidak pernah	Tidak pernah	Tidak pernah	Tidak pernah	Tidak pernah	Tidak pernah	Tidak pernah
Serat	Kurang dari 1 m	Produksi industri	Air Tawar, Air La	Ya	Ya	Mulut (saluran c	1-3x sepekan	3	<1x sebulan	1	4	>3x sehari	Tidak pernah	1-3x sebulan	1-3x sepekan	1-3x sepekan	1-3x sebulan	1-3x sepekan	1-3x sebulan	4-6x sepekan	1-3x sepekan	1-3x sepekan
Partikel tidak be	Kurang dari 1 m	Produksi industri	Air Tawar, Air La	Ya	Ya	Mulut (saluran c	1-3x sepekan	3	<1x sebulan	1	4	4-6x sepekan	Tidak pernah	1-3x sebulan	Tidak pernah	1-3x sepekan	1-3x sepekan	<1x sebulan	<1x sebulan	1-3x sepekan	<1x sebulan	1-3x sepekan
Partikel Tidak Be	Kurang dari 1 m	Produksi industri	Air Tawar, Udara	Ya	Tidak	Mulut (saluran c	1-3x sebulan	2	1-3x sebulan	2	4	Tidak pernah	Tidak pernah	1-3x sebulan	1-3x sebulan	1-3x sepekan	1-3x sebulan	Tidak pernah	1-3x sebulan	4-6x sepekan	1-3x sebulan	Tidak pernah
Serat, Partikel B	Kurang dari 1 m	Produksi industri	Air Tawar, Air La	Ya	Ya	Mulut (saluran c	1-3x sebulan	2	1-3x sebulan	2	4	1-3x sepekan	1-3x sepekan	1-3x sepekan	1-3x sepekan	1-3x sepekan	1-3x sebulan	<1x sebulan	<1x sebulan	1-3x sebulan	<1x sebulan	1-3x sebulan
Partikel Tidak Be	Kurang dari 1 m	Produksi industri	Air Laut, Biota L	Ya	Tidak	Hidung (Saluran	1-3x sebulan	2	1-3x sebulan	2	4	Tidak pernah	Tidak pernah	Tidak pernah	Tidak pernah	<1x sebulan	<1x sebulan	<1x sebulan	<1x sebulan	<1x sebulan	<1x sebulan	<1x sebulan
Serat	Kurang dari 1 m	Pelepasan serat	Air Laut	Ya	Ya	Mulut (saluran c	1-3x sebulan	2	1-3x sebulan	2	4	Tidak pernah	4-6x sepekan	1-3x sebulan	<1x sebulan	<1x sebulan	1-3x sebulan	<1x sebulan	1-3x sebulan	1-3x sepekan	1-3x sepekan	4-6x sepekan
Serat, Partikel ti	Sekitar 1 - 5 cm	Pencucian pakai	Air Tawar, Air La	Ya	Ya	Mulut (saluran c	1-3x sebulan	2	1-3x sebulan	2	4	1-3x sepekan	Tidak pernah	4-6x sepekan	1-3x sepekan	<1x sebulan	1-3x sebulan	1-3x sebulan	1-3x sepekan	1-3x sebulan	1-3x sebulan	1-3x sepekan
Serat	Kurang dari 1 m	Produksi industri	Biota Laut	Ya	Tidak	Mulut (saluran c	1-3x sebulan	2	1-3x sebulan	2	4	1-3x sebulan	Tidak pernah	<1x sebulan	1-3x sebulan	<1x sebulan	<1x sebulan	Tidak pernah	Tidak pernah	Tidak pernah	Tidak pernah	Tidak pernah
Serat, Film	Kurang dari 1 m	Pencucian pakai	Air Laut, Udara,	Ya	Ya	Mulut (saluran c	1-3x sebulan	2	1-3x sebulan	2	4	1-3x sebulan	Tidak pernah	1-3x sebulan	1-3x sebulan	1-3x sebulan	1-3x sebulan	<1x sebulan	1-3x sebulan	4-6x sepekan	Tidak pernah	1-3x sebulan
Partikel tidak be	Kurang dari 1 m	Produksi industri	Air Tawar, Air La	Ya	Ya	Mulut (saluran c	1-3x sebulan	2	1-3x sebulan	2	4	1-3x sehari	<1x sebulan	Tidak pernah	1-3x sepekan	1-3x sebulan	<1x sebulan	Tidak pernah	<1x sebulan	1-3x sepekan	1-3x sebulan	1-3x sehari
Partikel tidak be	Kurang dari 1 m	Produksi industri	Air Tawar, Air La	Ya	Ya	Mulut (saluran c	1-3x sebulan	2	1-3x sebulan	2	4	1-3x sehari	1-3x sehari	1-3x sepekan	>3x sehari	<1x sebulan	1-3x sebulan	1-3x sepekan	<1x sebulan	>3x sehari	1-3x sebulan	>3x sehari
Serat, Partikel B	Kurang dari 1 m	Produksi industri	Air Tawar, Air La	Ya	Ya	Mulut (saluran c	<1x sebulan	1	1-3x sepekan	3	4	1-3x sepekan	>3x sehari	4-6x sepekan	4-6x sepekan	1-3x sebulan	Tidak pernah	Tidak pernah	Tidak pernah	1-3x sepekan	<1x sebulan	1-3x sepekan
Serat, Partikel ti	Kurang dari 1 m	Produksi industri	Air Tawar, Air La	Ya	Ya	Mulut (saluran c	<1x sebulan	1	1-3x sepekan	3	4	<1x sebulan	1-3x sepekan	4-6x sepekan	1-3x sepekan	1-3x sepekan	1-3x sepekan	4-6x sepekan	4-6x sepekan	4-6x sepekan	4-6x sepekan	4-6x sepekan
Partikel bulat, Pa	Kurang dari 1 m	Produksi industri	Air Laut, Biota L	Ya	Ya	Mulut (saluran c	<1x sebulan	1	1-3x sepekan	3	4	>3x sehari	Tidak pernah	1-3x sebulan	1-3x sepekan	<1x sebulan	<1x sebulan	<1x sebulan	<1x sebulan	<1x sebulan	<1x sebulan	<1x sebulan
Serat, Partikel b	Kurang dari 1 m	Produksi industri	Air Tawar, Air La	Ya	Ya	Mulut (saluran c	<1x sebulan	1	1-3x sepekan	3	4	<1x sebulan	>3x sehari	<1x sebulan	1-3x sepekan	1-3x sebulan	1-3x sebulan	1-3x sepekan	1-3x sepekan	>3x sehari	1-3x sebulan	1-3x sepekan
Serat	Kurang dari 1 m	Penggunaan pro	Tanah	Tidak tahu	Tidak	Kulit	<1x sebulan	1	1-3x sepekan	3	4	<1x sebulan	Tidak pernah	Tidak pernah	1-3x sepekan	<1x sebulan	<1x sebulan	<1x sebulan	<1x sebulan	4-6x sepekan	<1x sebulan	<1x sebulan
Serat	Kurang dari 1 m	Produksi industri	Air Laut, Udara	Ya	Ya	Hidung (Saluran	>3x sehari	6	1-3x sehari	5	11	Tidak pernah	Tidak pernah	<1x sebulan	4-6x sepekan	Tidak pernah	Tidak pernah	Tidak pernah	Tidak pernah	Tidak pernah	Tidak pernah	Tidak pernah
Serat	Kurang dari 1 m	Pelepasan serat	Air Tawar, Air La	Ya	Ya	Mulut (saluran c	Tidak pernah	0	4-6x sepekan	4	4	1-3x sebulan	Tidak pernah	1-3x sebulan	1-3x sebulan	1-3x sepekan	1-3x sebulan	Tidak pernah	1-3x sepekan	4-6x sepekan	1-3x sebulan	1-3x sebulan
Serat, Partikel B	Kurang dari 1 m	Produksi industri	Air Tawar, Air La	Ya	Ya	Mulut (saluran c	1-3x sehari	5	Tidak pernah	0	5	Tidak pernah	Tidak pernah	>3x sehari	1-3x sehari	>3x sehari	>3x sehari	>3x sehari	>3x sehari	>3x sehari	>3x sehari	>3x sehari
Partikel bulat	Kurang dari 1 m	Penggunaan pro	Tanah	Ya	Ya	Mulut (saluran c	1-3x sehari	5	Tidak pernah	0	5	1-3x sehari	1-3x sehari	<1x sebulan	<1x sebulan	1-3x sepekan	<1x sebulan	<1x sebulan	1-3x sehari	1-3x sebulan	<1x sebulan	1-3x sepekan
Film	Kurang dari 1 m	Produksi industri	Air Tawar, Tanah	Ya	Ya	Mulut (saluran c	1-3x sehari	5	Tidak pernah	0	5	>3x sehari	Tidak pernah	Tidak pernah	>3x sehari	Tidak pernah	Tidak pernah	Tidak pernah	Tidak pernah	Tidak pernah	Tidak pernah	Tidak pernah
Partikel tidak be	Kurang dari 1 m	Produksi industri	Air Laut, Biota L	Ya	Ya	Mulut (saluran c	1-3x sehari	5	Tidak pernah	0	5	>3x sehari	Tidak pernah	1-3x sehari	1-3x sebulan	4-6x sepekan	1-3x sebulan	Tidak pernah	1-3x sebulan	1-3x sepekan	1-3x sebulan	1-3x sepekan
Serat	Kurang dari 1 m	Produksi industri	Produk hewani s	Ya	Ya	Hidung (Saluran	4-6x sepekan	4	<1x sebulan	1	5	1-3x sebulan	Tidak pernah	1-3x sebulan	1-3x sepekan	1-3x sehari	4-6x sepekan	4-6x sepekan	1-3x sepekan	1-3x sepekan	4-6x sepekan	1-3x sebulan
Partikel Tidak Be	Kurang dari 1 m	Produksi industri	Air Tawar, Air La	Ya	Ya	Mulut (saluran c	1-3x sepekan	3	1-3x sebulan	2	5	1-3x sebulan	Tidak pernah	1-3x sebulan	1-3x sepekan	4-6x sepekan	1-3x sepekan	1-3x sebulan	1-3x sebulan	4-6x sepekan	Tidak pernah	1-3x sebulan
Serat	Kurang dari 1 m	Pelepasan serat	Tanah	Tidak Tahu	Tidak	Hidung (Saluran	1-3x sepekan	3	1-3x sebulan	2	5	1-3x sehari	>3x sehari	<1x sebulan	1-3x sepekan	1-3x sehari	1-3x sepekan	Tidak pernah	1-3x sebulan	1-3x sehari	1-3x sepekan	>3x sehari
Serat, Partikel b	Kurang dari 1 m	Produksi industri	Air Tawar, Air La	Ya	Ya	Mulut (saluran c	1-3x sepekan	3	1-3x sebulan	2	5	>3x sehari	Tidak pernah	1-3x sebulan	<1x sebulan	1-3x sepekan	1-3x sebulan	1-3x sebulan	1-3x sebulan	1-3x sehari	1-3x sepekan	4-6x sepekan
Partikel tidak be	Sekitar 1 - 5 cm	Produksi industri	Air Laut	Ya	Ya	Mulut (saluran c	1-3x sepekan	3	1-3x sebulan	2	5	>3x sehari	Tidak pernah	1-3x sebulan	1-3x sepekan	1-3x sepekan	1-3x sebulan	Tidak pernah	1-3x sebulan	1-3x sepekan	<1x sebulan	>3x sehari
Serat	Kurang dari 1 m	Pelepasan serat	Produk hewani s	Tidak tahu	Ya	Mulut (saluran c	1-3x sepekan	3	1-3x sebulan	2	5	1-3x sebulan	Tidak pernah	1-3x sepekan	4-6x sepekan	1-3x sepekan	1-3x sepekan	4-6x sepekan	1-3x sepekan	Tidak pernah	Tidak pernah	Tidak pernah
Serat	Kurang dari 1 m	Produksi industri	Air Tawar, Air La	Ya	Ya	Mulut (saluran c	1-3x sepekan	3	1-3x sebulan	2	5	>3x sehari	Tidak pernah	1-3x sebulan	1-3x sebulan	1-3x sepekan	1-3x sebulan	1-3x sebulan	1-3x sebulan	1-3x sebulan	1-3x sebulan	1-3x sebulan
Serat, Partikel b	Kurang dari 1 m	Produksi industri	Air Tawar, Air La	Ya	Ya	Mulut (saluran c	1-3x sepekan	3	1-3x sebulan	2	5	>3x sehari	Tidak pernah	1-3x sebulan	1-3x sebulan	1-3x sepekan	1-3x sebulan	<1x sebulan	<1x sebulan	4-6x sepekan	1-3x sepekan	4-6x sepekan
Serat, Partikel b	Sekitar 1 - 5 cm	Produksi industri	Air Tawar, Air La	Ya	Ya	Mulut (saluran c	1-3x sepekan	3	1-3x sebulan	2	5	Tidak pernah	>3x sehari	<1x sebulan	4-6x sepekan	1-3x sebulan	1-3x sepekan	1-3x sehari	1-3x sepekan	<1x sebulan	<1x sebulan	Tidak pernah
Serat, Partikel B	Kurang dari 1 m	Produksi industri	Air Tawar, Air La	Ya	Ya	Mulut (saluran c	1-3x sebulan	2	1-3x sepekan	3	5	>3x sehari	1-3x sehari	1-3x sepekan	<1x sebulan	<1x sebulan	<1x sebulan	<1x sebulan	1-3x sehari	1-3x sehari	Tidak pernah	4-6x sepekan
Serat, Partikel Ti	Sekitar 1 - 5 cm	Produksi industri	Tanah	Ya	Tidak	Hidung (Saluran	1-3x sebulan	2	1-3x sepekan	3	5	1-3x sepekan	Tidak pernah	Tidak pernah	Tidak pernah	Tidak pernah	Tidak pernah	Tidak pernah	Tidak pernah	Tidak pernah	Tidak pernah	Tidak pernah
Partikel Bulat	Kurang dari 1 m	Penggunaan pro	Tanah	Ya	Ya	Mulut (saluran c	1-3x sebulan	2	1-3x sepekan	3	5	>3x sehari	1-3x sehari	1-3x sebulan	1-3x sebulan	1-3x sepekan	1-3x sepekan	1-3x sepekan	1-3x sebulan	<1x sebulan	1-3x sepekan	1-3x sebulan
Partikel Bulat, Pa	Kurang dari 1 m	Penggunaan pro	Tanah	Ya	Tidak	Mulut (saluran c	1-3x sebulan	2	1-3x sepekan	3	5	1-3x sebulan	Tidak pernah	Tidak pernah	1-3x sebulan	Tidak pernah	Tidak pernah	Tidak pernah	Tidak pernah	Tidak pernah	Tidak pernah	Tidak pernah
Partikel Bulat, Pa	Kurang dari 1 m	Produksi industri	Air Tawar, Air La	Ya	Ya	Mulut (saluran c	1-3x sebulan	2	1-3x sepekan	3	5	1-3x sebulan	Tidak pernah	1-3x sepekan	1-3x sebulan	<1x sebulan	Tidak pernah	Tidak pernah	1-3x sebulan	1-3x sebulan	1-3x sebulan	1-3x sebulan
Serat, Partikel ti	Kurang dari 1 m	Produksi industri	Air Tawar, Air La	Ya	Ya	Mulut (saluran c	1-3x sebulan	2	1-3x sepekan	3	5	1-3x sebulan	<1x sebulan	4-6x sepekan	1-3x sebulan	1-3x sepekan	1-3x sebulan	<1x sebulan	1-3x sepekan	1-3x sebulan	<1x sebulan	1-3x sebulan
Partikel tidak be	Kurang dari 1 m	Penggunaan pro	Air Tawar, Air La	Ya	Ya	Mulut (saluran c	1-3x sebulan	2	1-3x sepekan	3	5	>3x sehari	Tidak pernah	1-3x sepekan	1-3x sepekan	1-3x sepekan	1-3x sebulan	<1x sebulan	<1x sebulan	<1x sebulan	<1x sebulan	>3x sehari
Serat, Partikel b	Kurang dari 1 m	Pelepasan serat	Air Tawar, Air La	Ya	Ya	Mulut (saluran c	1-3x sebulan	2	1-3x sepekan	3	5	1-3x sehari	Tidak pernah	4-6x sepekan	1-3x sebulan	1-3x sepekan	1-3x sebulan	<1x sebulan	4-6x sepekan	1-3x sepekan	<1x sebulan	1-3x sepekan
Partikel tidak be	Kurang dari 1 m	Produksi industri	Air Tawar, Air La	Ya	Ya	Mulut (saluran c	1-3x sebulan	2	1-3x sepekan	3	5	>3x sehari	Tidak pernah	1-3x sepekan	1-3x sebulan	1-3x sepekan	4-6x sepekan	Tidak pernah	4-6x sepekan	>3x sehari	1-3x sebulan	>3x sehari
Partikel tidak be	Kurang dari 1 m	Penggunaan pro	Air Tawar, Air La	Ya	Ya	Mulut (saluran c	1-3x sebulan	2	1-3x sepekan	3	5	>3x sehari	Tidak pernah	1-3x sepekan	4-6x sepekan	<1x sebulan	1-3x sepekan	<1x sebulan	<1x sebulan	1-3x sebulan	Tidak pernah	>3x sehari
Partikel Tidak Be	Kurang dari 1 m	Produksi industri	Air Tawar, Air La	Ya	Ya	Mulut (saluran c	<1x sebulan	1	4-6x sepekan	4	5	Tidak pernah	1-3x sehari	4-6x sepekan	1-3x sepekan	1-3x sebulan	1-3x sepekan	Tidak pernah	1-3x sepekan	Tidak pernah	Tidak pernah	1-3x sehari
Serat, Partikel b	Kurang dari 1 m	Produksi industri	Air Tawar, Air La	Ya	Ya	Mulut (saluran c	<1x sebulan	1	4-6x sepekan	4	5	1-3x sebulan	Tidak pernah	1-3x sepekan	1-3x sepekan	1-3x sebulan	<1x sebulan	<1x sebulan	4-6x sepekan	1-3x sepekan	Tidak pernah	<1x sebulan
Partikel tidak be	Kurang dari 1 m	Produksi industri	Air Tawar, Air La	Ya	Tidak	Mulut (saluran c	<1x sebulan	1	4-6x sepekan	4	5	Tidak pernah	>3x sehari	<1x sebulan	<1x sebulan	<1x sebulan	1-3x sebulan	Tidak pernah	4-6x sepekan	>3x sehari	1-3x sebulan	Tidak pernah
Serat, Partikel b	Kurang dari 1 m	Produksi industri	Air Tawar, Air La	Ya	Ya	Mulut (saluran c	<1x sebulan	1	4-6x sepekan	4	5	<1x sebulan	Tidak pernah	Tidak pernah	1-3x sepekan	1-3x sebulan	<1x sebulan	<1x sebulan	4-6x sepekan	4-6x sepekan	1-3x sebulan	<1x sebulan
Partikel bulat	Kurang dari 1 m	Penggunaan pro	Air Tawar, Air La	Ya	Ya	Mulut (saluran c	1-3x sehari	5	<1x sebulan	1	6	1-3x sehari	Tidak pernah	1-3x sehari	<1x sebulan	1-3x sehari	Tidak pernah	Tidak pernah	Tidak pernah	1-3x sehari	Tidak pernah	Tidak pernah
Partikel bulat	Kurang dari 1 m	Penggunaan pro	Air Tawar, Air La	Ya	Tidak	Mulut (saluran c	1-3x sehari	5	<1x sebulan	1	6	1-3x sebulan	Tidak pernah	1-3x sepekan	1-3x sepekan	1-3x sebulan	<1x sebulan	Tidak pernah	Tidak pernah	Tidak pernah	Tidak pernah	1-3x sebulan
Film	Sekitar 1 - 5 cm	Produksi industri	Air Laut, Udara,	Ya	Ya	Mulut (saluran c	4-6x sepekan	4	1-3x sebulan	2	6	Tidak pernah	1-3x sehari	1-3x sebulan	4-6x sepekan	<1x sebulan	<1x sebulan	<1x sebulan	1-3x sebulan	1-3x sebulan	1-3x sebulan	<1x sebulan
Partikel tidak be	Kurang dari 1 m	Penggunaan pro	Tanah	Ya	Ya	Kulit	4-6x sepekan	4	1-3x sebulan	2	6	1-3x sepekan	4-6x sepekan	1-3x sepekan	4-6x sepekan	1-3x sebulan	<1x sebulan	<1x sebulan	1-3x sepekan	1-3x sebulan	Tidak pernah	<1x sebulan
Partikel tidak be	Kurang dari 1 m	Produksi industri	Air Tawar, Air La	Tidak tahu	Tidak	Mulut (saluran c	4-6x sepekan	4	1-3x sebulan	2	6	>3x sehari	1-3x sebulan	1-3x sepekan	4-6x sepekan	1-3x sebulan	1-3x sebulan	Tidak pernah	Tidak pernah	Tidak pernah	Tidak pernah	Tidak pernah
Serat, Partikel B	Kurang dari 1 m	Produksi industri	Air Laut, Tanah	Ya	Ya	Mulut (saluran c	1-3x sepekan	3	1-3x sepekan	3	6	Tidak pernah	1-3x sebulan	1-3x sepekan	1-3x sepekan	1-3x sepekan	1-3x sepekan	1-3x sepekan	1-3x sepekan	1-3x sepekan	1-3x sepekan	1-3x sepekan
Serat	Sekitar 1 - 5 cm	Produksi industri	Tanah	Ya	Tidak	Mulut (saluran c	1-3x sepekan	3	1-3x sepekan	3	6	>3x sehari	>3x sehari	1-3x sepekan	Tidak pernah	1-3x sehari	1-3x sebulan	<1x sebulan	<1x sebulan	1-3x sepekan	Tidak pernah	1-3x sehari
Partikel Tidak Be	Kurang dari 1 m	Pelepasan serat	Air Laut, Tanah,	Ya	Ya	Mulut (saluran c	1-3x sepekan	3	1-3x sepekan	3	6	>3x sehari	Tidak pernah	1-3x sehari	1-3x sebulan	1-3x sebulan	1-3x sepekan	<1x sebulan	1-3x sepekan	>3x sehari	1-3x sebulan	>3x sehari
Serat, Partikel B	Kurang dari 1 m	Produksi industri	Air Tawar, Air La	Ya	Ya	Mulut (saluran c	1-3x sepekan	3	1-3x sepekan	3	6	>3x sehari	1-3x sebulan	1-3x sebulan	>3x sehari	4-6x sepekan	4-6x sepekan	1-3x sebulan	1-3x sepekan	4-6x sepekan	Tidak pernah	<1x sebulan
Serat, Partikel b	Kurang dari 1 m	Produksi industri	Air Tawar, Air La	Ya	Ya	Mulut (saluran c	1-3x sepekan	3	1-3x sepekan	3	6	1-3x sepekan	4-6x sepekan	1-3x sepekan	4-6x sepekan	1-3x sebulan	1-3x sebulan	<1x sebulan	4-6x sepekan	<1x sebulan	<1x sebulan	1-3x sebulan
Partikel tidak be	Kurang dari 1 m	Pelepasan serat	Biota Laut	Ya	Ya	Mulut (saluran c	1-3x sepekan	3	1-3x sepekan	3	6	>3x sehari	<1x sebulan	1-3x sehari	1-3x sehari	Tidak pernah	Tidak pernah	Tidak pernah	Tidak pernah	Tidak pernah	Tidak pernah	Tidak pernah
Serat, Film	Sekitar 1 - 5 cm	Produksi industri	Air Laut	Ya	Ya	Mulut (saluran c	1-3x sepekan	3	1-3x sepekan	3	6	>3x sehari	Tidak pernah	1-3x sepekan	4-6x sepekan	4-6x sepekan	1-3x sepekan	<1x sebulan	1-3x sehari	1-3x sepekan	Tidak pernah	>3x sehari
Serat	Kurang dari 1 m	Penggunaan pro	Air Tawar, Air La	Ya	Ya	Mulut (saluran c	1-3x sepekan	3	1-3x sepekan	3	6	>3x sehari	Tidak pernah	4-6x sepekan	4-6x sepekan	4-6x sepekan	4-6x sepekan	4-6x sepekan	4-6x sepekan	1-3x sepekan	<1x sebulan	<1x sebulan
Serat	Sekitar 1 - 5 cm	Produksi industri	Air Tawar	Ya	Tidak	Mulut (saluran c	1-3x sepekan	3	1-3x sepekan	3	6	4-6x sepekan	Tidak pernah	1-3x sepekan	1-3x sebulan	4-6x sepekan	1-3x sebulan	1-3x sebulan	1-3x sepekan	<1x sebulan	1-3x sepekan	1-3x sepekan
Partikel bulat	Kurang dari 1 m	Produksi industri	Air Tawar, Udara	Ya	Tidak	Mulut (saluran c	1-3x sepekan	3	1-3x sepekan	3	6	>3x sehari	Tidak pernah	Tidak pernah	1-3x sepekan	1-3x sepekan	Tidak pernah	Tidak pernah	Tidak pernah	Tidak pernah	Tidak pernah	Tidak pernah
Partikel tidak be	Kurang dari 1 m	Produksi industri	Air Tawar, Air La	Ya	Ya	Mulut (saluran c	1-3x sepekan	3	1-3x sepekan	3	6	1-3x sehari	>3x sehari	1-3x sepekan	1-3x sepekan	1-3x sepekan	1-3x sepekan	1-3x sepekan	1-3x sepekan	1-3x sepekan	1-3x sepekan	1-3x sepekan
Partikel tidak be	Kurang dari 1 m	Produksi industri	Tanah, Biota Lau	Ya	Ya	Mulut (saluran c	1-3x sepekan	3	1-3x sepekan	3	6	<1x sebulan	1-3x sepekan	1-3x sebulan	1-3x sehari	1-3x sebulan	1-3x sepekan	4-6x sepekan	4-6x sepekan	4-6x sepekan	<1x sebulan	<1x sebulan
Partikel tidak be	Kurang dari 1 m	Penggunaan pro	Air Tawar, Produ	Ya	Ya	Mulut (saluran c	1-3x sepekan	3	1-3x sepekan	3	6	>3x sehari	1-3x sehari	1-3x sepekan	1-3x sepekan	1-3x sepekan	1-3x sebulan	1-3x sebulan	1-3x sebulan	1-3x sebulan	1-3x sebulan	1-3x sepekan
Serat	Kurang dari 1 m	Pelepasan serat	Air Tawar, Air La	Ya	Tidak	Mulut (saluran c	1-3x sepekan	3	1-3x sepekan	3	6	1-3x sepekan	>3x sehari	Tidak pernah	1-3x sepekan	1-3x sehari	Tidak pernah	Tidak pernah	Tidak pernah	Tidak pernah	Tidak pernah	Tidak pernah
Serat	Kurang dari 1 m	Penggunaan pro	Udara, Biota Lau	Ya	Tidak	Mulut (saluran c	1-3x sepekan	3	1-3x sepekan	3	6	Tidak pernah	1-3x sehari	1-3x sepekan	<1x sebulan	1-3x sepekan	1-3x sepekan	1-3x sebulan	1-3x sebulan	1-3x sehari	<1x sebulan	>3x sehari
Serat	Kurang dari 1 m	Penggunaan pro	Air Laut	Ya	Ya	Mulut (saluran c	1-3x sepekan	3	1-3x sepekan	3	6	1-3x sepekan	Tidak pernah	Tidak pernah	<1x sebulan	1-3x sebulan	1-3x sebulan	<1x sebulan	1-3x sebulan	4-6x sepekan	1-3x sebulan	1-3x sebulan
Serat	Kurang dari 1 m	Produksi industri	Air Laut, Udara,	Ya	Ya	Mulut (saluran c	1-3x sepekan	3	1-3x sepekan	3	6	>3x sehari	>3x sehari	1-3x sepekan	1-3x sepekan	1-3x sepekan	1-3x sepekan	Tidak pernah	1-3x sepekan	4-6x sepekan	1-3x sebulan	1-3x sehari
Partikel Bulat	Kurang dari 1 m	Produksi industri	Air Tawar, Air La	Ya	Ya	Mulut (saluran c	1-3x sebulan	2	4-6x sepekan	4	6	1-3x sepekan	<1x sebulan	1-3x sehari	4-6x sepekan	1-3x sepekan	1-3x sebulan	1-3x sebulan	4-6x sepekan	1-3x sehari	1-3x sepekan	1-3x sepekan
Partikel tidak be	Kurang dari 1 m	Penggunaan pro	Air Tawar	Ya	Ya	Mulut (saluran c	1-3x sebulan	2	4-6x sepekan	4	6	1-3x sepekan	Tidak pernah	Tidak pernah	Tidak pernah	4-6x sepekan	4-6x sepekan	1-3x sebulan	<1x sebulan	Tidak pernah	Tidak pernah	Tidak pernah
Serat, Partikel b	Kurang dari 1 m	Produksi industri	Air Tawar, Air La	Ya	Ya	Mulut (saluran c	1-3x sebulan	2	4-6x sepekan	4	6	1-3x sepekan	1-3x sehari	1-3x sepekan	1-3x sepekan	1-3x sepekan	1-3x sebulan	<1x sebulan	1-3x sepekan	1-3x sehari	1-3x sebulan	4-6x sepekan
Partikel tidak be	Kurang dari 1 m	Produksi industri	Tanah	Ya	Tidak	Mulut (saluran c	1-3x sebulan	2	4-6x sepekan	4	6	1-3x sebulan	Tidak pernah	1-3x sepekan	1-3x sepekan	1-3x sepekan	4-6x sepekan	Tidak pernah	1-3x sepekan	1-3x sebulan	1-3x sebulan	1-3x sehari
Partikel tidak be	Kurang dari 1 m	Pelepasan serat	Air Laut	Tidak tahu	Tidak	Mulut (saluran c	1-3x sebulan	2	4-6x sepekan	4	6	4-6x sepekan	Tidak pernah	4-6x sepekan	4-6x sepekan	Tidak pernah	Tidak pernah	Tidak pernah	Tidak pernah	Tidak pernah	Tidak pernah	Tidak pernah
Partikel tidak be	Kurang dari 1 m	Produksi industri	Udara, Tanah	Ya	Tidak	Mulut (saluran c	1-3x sebulan	2	4-6x sepekan	4	6	1-3x sebulan	Tidak pernah	1-3x sepekan	Tidak pernah	1-3x sebulan	<1x sebulan	Tidak pernah	1-3x sepekan	1-3x sebulan	1-3x sebulan	<1x sebulan
Serat	Kurang dari 1 m	Penggunaan pro	Air Tawar, Air La	Tidak Tahu	Ya	Mulut (saluran c	<1x sebulan	1	1-3x sehari	5	6	>3x sehari	Tidak pernah	1-3x sebulan	1-3x sehari	1-3x sepekan	<1x sebulan	Tidak pernah	4-6x sepekan	1-3x sehari	>3x sehari	>3x sehari
Serat, Partikel b	Kurang dari 1 m	Penggunaan pro	Air Laut	Ya	Ya	Mulut (saluran c	<1x sebulan	1	1-3x sehari	5	6	Tidak pernah	<1x sebulan	1-3x sebulan	1-3x sebulan	1-3x sepekan	1-3x sepekan	1-3x sepekan	1-3x sepekan	1-3x sepekan	1-3x sepekan	1-3x sebulan
Partikel bulat	Lebih dari 10 cm	Produksi industri	Air Laut, Biota L	Ya	Ya	Mulut (saluran c	Tidak pernah	0	>3x sehari	6	6	1-3x sehari	1-3x sehari	<1x sebulan	<1x sebulan	Tidak pernah	Tidak pernah	Tidak pernah	1-3x sepekan	1-3x sehari	1-3x sebulan	<1x sebulan
Serat	Kurang dari 1 m	Produksi industri	Air Tawar, Air La	Ya	Tidak	Mulut (saluran c	>3x sehari	6	<1x sebulan	1	7	>3x sehari	Tidak pernah	1-3x sebulan	<1x sebulan	4-6x sepekan	1-3x sepekan	Tidak pernah	<1x sebulan	1-3x sebulan	Tidak pernah	>3x sehari
Film	Sekitar 1 - 5 cm	Produksi industri	Tanah	Ya	Ya	Mulut (saluran c	1-3x sehari	5	1-3x sebulan	2	7	1-3x sebulan	1-3x sebulan	<1x sebulan	<1x sebulan	<1x sebulan	<1x sebulan	<1x sebulan	1-3x sebulan	1-3x sebulan	<1x sebulan	1-3x sebulan
Serat, Partikel Ti	Kurang dari 1 m	Pelepasan serat	Air Tawar, Air La	Ya	Tidak	Mulut (saluran c	4-6x sepekan	4	1-3x sepekan	3	7	1-3x sebulan	1-3x sehari	4-6x sepekan	4-6x sepekan	1-3x sehari	4-6x sepekan	<1x sebulan	1-3x sehari	>3x sehari	1-3x sebulan	<1x sebulan
Serat	Kurang dari 1 m	Penggunaan pro	Air Tawar, Air La	Ya	Tidak	Mulut (saluran c	4-6x sepekan	4	1-3x sepekan	3	7	1-3x sehari	Tidak pernah	1-3x sehari	1-3x sebulan	1-3x sehari	1-3x sepekan	Tidak pernah	1-3x sehari	1-3x sehari	Tidak pernah	1-3x sehari
Serat, Partikel B	Kurang dari 1 m	Pelepasan serat	Air Laut	Ya	Ya	Hidung (Saluran	4-6x sepekan	4	1-3x sepekan	3	7	1-3x sepekan	4-6x sepekan	Tidak pernah	Tidak pernah	1-3x sepekan	Tidak pernah	1-3x sepekan	Tidak pernah	Tidak pernah	Tidak pernah	Tidak pernah
Serat, Partikel Ti	Kurang dari 1 m	Produksi industri	Air Tawar, Air La	Ya	Ya	Mulut (saluran c	4-6x sepekan	4	1-3x sepekan	3	7	1-3x sehari	1-3x sepekan	1-3x sebulan	4-6x sepekan	4-6x sepekan	1-3x sebulan	<1x sebulan	<1x sebulan	1-3x sehari	1-3x sebulan	1-3x sepekan
Serat	Kurang dari 1 m	Produksi industri	Air Tawar, Air La	Ya	Tidak	Mulut (saluran c	4-6x sepekan	4	1-3x sepekan	3	7	4-6x sepekan	Tidak pernah	Tidak pernah	1-3x sepekan	4-6x sepekan	4-6x sepekan	1-3x sepekan	1-3x sepekan	1-3x sebulan	1-3x sebulan	Tidak pernah
Serat, Partikel ti	Kurang dari 1 m	Pelepasan serat	Air Laut	Ya	Ya	Mulut (saluran c	4-6x sepekan	4	1-3x sepekan	3	7	1-3x sehari	1-3x sehari	>3x sehari	1-3x sehari	1-3x sehari	1-3x sehari	4-6x sepekan	1-3x sehari	4-6x sepekan	1-3x sebulan	1-3x sehari
Serat, Partikel ti	Kurang dari 1 m	Penggunaan pro	Air Tawar, Air La	Ya	Ya	Mulut (saluran c	4-6x sepekan	4	1-3x sepekan	3	7	>3x sehari	>3x sehari	1-3x sebulan	1-3x sepekan	1-3x sebulan	1-3x sebulan	1-3x sebulan	<1x sebulan	<1x sebulan	<1x sebulan	<1x sebulan
Serat	Kurang dari 1 m	Produksi industri	Air Tawar, Air La	Ya	Ya	Mulut (saluran c	4-6x sepekan	4	1-3x sepekan	3	7	>3x sehari	Tidak pernah	1-3x sepekan	1-3x sepekan	1-3x sepekan	1-3x sepekan	1-3x sepekan	1-3x sepekan	1-3x sepekan	1-3x sepekan	1-3x sepekan
Serat	Kurang dari 1 m	Produksi industri	Air Tawar, Tanah	Ya	Ya	Mulut (saluran c	4-6x sepekan	4	1-3x sepekan	3	7	4-6x sepekan	Tidak pernah	1-3x sebulan	4-6x sepekan	Tidak pernah	Tidak pernah	Tidak pernah	Tidak pernah	Tidak pernah	Tidak pernah	Tidak pernah
Serat	Kurang dari 1 m	Penggunaan pro	Tanah	Ya	Tidak	Mulut (saluran c	4-6x sepekan	4	1-3x sepekan	3	7	>3x sehari	<1x sebulan	1-3x sepekan	<1x sebulan	<1x sebulan	1-3x sepekan	<1x sebulan	1-3x sepekan	1-3x sepekan	1-3x sepekan	<1x sebulan
Partikel bulat, Pa	Kurang dari 1 m	Pelepasan serat	Air Laut	Ya	Ya	Mulut (saluran c	4-6x sepekan	4	1-3x sepekan	3	7	>3x sehari	Tidak pernah	<1x sebulan	<1x sebulan	1-3x sepekan	1-3x sepekan	Tidak pernah	1-3x sebulan	1-3x sepekan	1-3x sebulan	1-3x sepekan
Partikel tidak be	Kurang dari 1 m	Produksi industri	Air Tawar, Udara	Ya	Ya	Mulut (saluran c	4-6x sepekan	4	1-3x sepekan	3	7	>3x sehari	Tidak pernah	4-6x sepekan	4-6x sepekan	4-6x sepekan	1-3x sepekan	Tidak pernah	Tidak pernah	1-3x sepekan	Tidak pernah	Tidak pernah
Serat, Partikel ti	Kurang dari 1 m	Produksi industri	Air Tawar, Air La	Ya	Ya	Mulut (saluran c	4-6x sepekan	4	1-3x sepekan	3	7	Tidak pernah	>3x sehari	1-3x sebulan	1-3x sepekan	1-3x sepekan	1-3x sehari	>3x sehari	1-3x sebulan	<1x sebulan	<1x sebulan	Tidak pernah
Serat, Film	Kurang dari 1 m	Produksi industri	Air Tawar	Ya	Ya	Mulut (saluran c	4-6x sepekan	4	1-3x sepekan	3	7	4-6x sepekan	Tidak pernah	1-3x sepekan	1-3x sepekan	1-3x sebulan	1-3x sebulan	Tidak pernah	4-6x sepekan	4-6x sepekan	1-3x sepekan	1-3x sepekan
Serat, Partikel b	Kurang dari 1 m	Produksi industri	Air Tawar, Air La	Ya	Tidak	Mulut (saluran c	4-6x sepekan	4	1-3x sepekan	3	7	>3x sehari	Tidak pernah	1-3x sehari	1-3x sepekan	1-3x sepekan	1-3x sebulan	Tidak pernah	<1x sebulan	>3x sehari	<1x sebulan	1-3x sehari
Serat	Kurang dari 1 m	Produksi industri	Udara, Tanah	Ya	Tidak	Mulut (saluran c	4-6x sepekan	4	1-3x sepekan	3	7	4-6x sepekan	Tidak pernah	1-3x sepekan	1-3x sepekan	1-3x sebulan	1-3x sebulan	<1x sebulan	1-3x sepekan	4-6x sepekan	1-3x sepekan	4-6x sepekan
Partikel tidak be	Kurang dari 1 m	Produksi industri	Air Tawar, Air La	Ya	Ya	Mulut (saluran c	4-6x sepekan	4	1-3x sepekan	3	7	1-3x sepekan	>3x sehari	1-3x sepekan	1-3x sepekan	1-3x sebulan	<1x sebulan	Tidak pernah	1-3x sebulan	1-3x sepekan	<1x sebulan	<1x sebulan
Partikel tidak be	Kurang dari 1 m	Produksi industri	Air Tawar, Air La	Ya	Ya	Hidung (Saluran	>3x sehari	6	1-3x sehari	5	11	4-6x sepekan	1-3x sehari	1-3x sebulan	4-6x sepekan	1-3x sepekan	1-3x sepekan	1-3x sepekan	1-3x sepekan	1-3x sehari	1-3x sepekan	1-3x sepekan
Serat, Partikel B	Kurang dari 1 m	Produksi industri	Air Laut, Udara,	Ya	Ya	Mulut (saluran c	1-3x sepekan	3	4-6x sepekan	4	7	1-3x sebulan	Tidak pernah	1-3x sebulan	Tidak pernah	1-3x sebulan	<1x sebulan	<1x sebulan	1-3x sebulan	1-3x sebulan	<1x sebulan	1-3x sebulan
Serat, Partikel Ti	Kurang dari 1 m	Pelepasan serat	Air Tawar, Air La	Ya	Tidak	Mulut (saluran c	1-3x sepekan	3	4-6x sepekan	4	7	1-3x sebulan	Tidak pernah	<1x sebulan	1-3x sepekan	1-3x sebulan	1-3x sepekan	1-3x sepekan	1-3x sepekan	1-3x sepekan	1-3x sepekan	4-6x sepekan
Film	Kurang dari 1 m	Penggunaan pro	Air Laut, Tanah,	Ya	Tidak	Mulut (saluran c	1-3x sepekan	3	4-6x sepekan	4	7	>3x sehari	Tidak pernah	1-3x sepekan	4-6x sepekan	1-3x sebulan	1-3x sebulan	1-3x sebulan	1-3x sebulan	1-3x sebulan	1-3x sebulan	1-3x sebulan
Partikel bulat	Sekitar 1 - 5 cm	Produksi industri	Air Tawar, Tanah	Ya	Ya	Mulut (saluran c	1-3x sepekan	3	4-6x sepekan	4	7	>3x sehari	<1x sebulan	4-6x sepekan	>3x sehari	4-6x sepekan	4-6x sepekan	1-3x sepekan	1-3x sepekan	1-3x sepekan	<1x sebulan	<1x sebulan
Serat	Kurang dari 1 m	Produksi industri	Air Laut, Biota L	Ya	Ya	Mulut (saluran c	1-3x sepekan	3	4-6x sepekan	4	7	>3x sehari	>3x sehari	4-6x sepekan	1-3x sehari	1-3x sehari	4-6x sepekan	<1x sebulan	1-3x sehari	1-3x sehari	1-3x sepekan	>3x sehari
Serat, Partikel b	Kurang dari 1 m	Produksi industri	Air Tawar, Air La	Ya	Ya	Mulut (saluran c	1-3x sepekan	3	4-6x sepekan	4	7	Tidak pernah	Tidak pernah	1-3x sebulan	4-6x sepekan	1-3x sepekan	<1x sebulan	Tidak pernah	1-3x sepekan	4-6x sepekan	1-3x sepekan	<1x sebulan
Serat	Kurang dari 1 m	Produksi industri	Air Tawar, Air La	Ya	Tidak	Mulut (saluran c	1-3x sepekan	3	4-6x sepekan	4	7	>3x sehari	Tidak pernah	Tidak pernah	Tidak pernah	Tidak pernah	Tidak pernah	Tidak pernah	Tidak pernah	Tidak pernah	Tidak pernah	Tidak pernah
Partikel tidak be	Kurang dari 1 m	Produksi industri	Air Tawar, Air La	Ya	Ya	Mulut (saluran c	1-3x sepekan	3	4-6x sepekan	4	7	>3x sehari	4-6x sepekan	1-3x sebulan	1-3x sebulan	<1x sebulan	1-3x sebulan	4-6x sepekan	1-3x sepekan	1-3x sepekan	<1x sebulan	1-3x sehari
Partikel tidak be	Kurang dari 1 m	Produksi industri	Air Tawar, Air La	Ya	Ya	Mulut (saluran c	1-3x sepekan	3	4-6x sepekan	4	7	1-3x sebulan	Tidak pernah	4-6x sepekan	1-3x sepekan	1-3x sepekan	1-3x sebulan	<1x sebulan	4-6x sepekan	>3x sehari	>3x sehari	>3x sehari
Film	Kurang dari 1 m	Produksi industri	Air Tawar, Air La	Ya	Ya	Mulut (saluran c	1-3x sepekan	3	4-6x sepekan	4	7	<1x sebulan	>3x sehari	<1x sebulan	4-6x sepekan	Tidak pernah	<1x sebulan	Tidak pernah	<1x sebulan	4-6x sepekan	Tidak pernah	Tidak pernah
Partikel tidak be	Kurang dari 1 m	Produksi industri	Air Tawar, Air La	Ya	Ya	Mulut (saluran c	1-3x sepekan	3	4-6x sepekan	4	7	<1x sebulan	Tidak pernah	4-6x sepekan	1-3x sepekan	1-3x sepekan	4-6x sepekan	1-3x sebulan	1-3x sehari	>3x sehari	1-3x sebulan	1-3x sepekan
Serat	Kurang dari 1 m	Produksi industri	Air Tawar, Air La	Ya	Ya	Mulut (saluran c	1-3x sebulan	2	1-3x sehari	5	7	1-3x sebulan	Tidak pernah	1-3x sehari	>3x sehari	4-6x sepekan	<1x sebulan	<1x sebulan	1-3x sebulan	1-3x sehari	<1x sebulan	1-3x sebulan
Film	Kurang dari 1 m	Produksi industri	Air Tawar, Air La	Ya	Ya	Mulut (saluran c	1-3x sebulan	2	1-3x sehari	5	7	>3x sehari	Tidak pernah	1-3x sehari	1-3x sehari	1-3x sepekan	<1x sebulan	Tidak pernah	1-3x sehari	1-3x sehari	1-3x sepekan	1-3x sehari
Serat, Partikel ti	Kurang dari 1 m	Produksi industri	Air Tawar, Air La	Ya	Tidak	Mulut (saluran c	1-3x sebulan	2	1-3x sehari	5	7	1-3x sebulan	Tidak pernah	Tidak pernah	Tidak pernah	Tidak pernah	Tidak pernah	Tidak pernah	Tidak pernah	Tidak pernah	Tidak pernah	Tidak pernah
Partikel bulat	Kurang dari 1 m	Penggunaan pro	Air Tawar, Air La	Ya	Ya	Mulut (saluran c	1-3x sebulan	2	1-3x sehari	5	7	>3x sehari	Tidak pernah	1-3x sebulan	4-6x sepekan	Tidak pernah	Tidak pernah	Tidak pernah	Tidak pernah	Tidak pernah	Tidak pernah	Tidak pernah
Serat	Kurang dari 1 m	Penggunaan pro	Udara	Ya	Tidak	Hidung (Saluran	<1x sebulan	1	>3x sehari	6	7	Tidak pernah	>3x sehari	>3x sehari	Tidak pernah	1-3x sehari	Tidak pernah	Tidak pernah	Tidak pernah	>3x sehari	Tidak pernah	Tidak pernah
Film	Kurang dari 1 m	Produksi industri	Air Tawar, Air La	Ya	Ya	Mulut (saluran c	<1x sebulan	1	>3x sehari	6	7	>3x sehari	Tidak pernah	<1x sebulan	>3x sehari	>3x sehari	1-3x sepekan	Tidak pernah	>3x sehari	1-3x sebulan	Tidak pernah	1-3x sebulan
Partikel Bulat	Kurang dari 1 m	Produksi industri	Air Tawar, Air La	Ya	Ya	Mulut (saluran c	<1x sebulan	1	>3x sehari	6	7	>3x sehari	Tidak pernah	>3x sehari	1-3x sepekan	1-3x sebulan	4-6x sepekan	Tidak pernah	4-6x sepekan	>3x sehari	1-3x sebulan	>3x sehari
Film	Sekitar 1 - 5 cm	Produksi industri	Air Laut, Biota L	Ya	Tidak	Mulut (saluran c	<1x sebulan	1	>3x sehari	6	7	<1x sebulan	<1x sebulan	>3x sehari	>3x sehari	>3x sehari	>3x sehari	>3x sehari	>3x sehari	4-6x sepekan	1-3x sepekan	<1x sebulan
Serat, Partikel B	Kurang dari 1 m	Produksi industri	Air Laut, Biota L	Ya	Ya	Mulut (saluran c	>3x sehari	6	1-3x sebulan	2	8	1-3x sebulan	Tidak pernah	<1x sebulan	1-3x sebulan	1-3x sebulan	1-3x sebulan	Tidak pernah	1-3x sepekan	1-3x sepekan	<1x sebulan	Tidak pernah
Partikel bulat, Pa	Kurang dari 1 m	Produksi industri	Air Tawar, Air La	Ya	Ya	Mulut (saluran c	>3x sehari	6	1-3x sebulan	2	8	>3x sehari	<1x sebulan	4-6x sepekan	1-3x sebulan	1-3x sepekan	1-3x sepekan	1-3x sebulan	1-3x sepekan	4-6x sepekan	1-3x sebulan	1-3x sebulan
Serat	Kurang dari 1 m	Produksi industri	Air Tawar, Air La	Tidak tahu	Tidak	Hidung (Saluran	>3x sehari	6	1-3x sebulan	2	8	Tidak pernah	Tidak pernah	>3x sehari	>3x sehari	4-6x sepekan	>3x sehari	>3x sehari	>3x sehari	1-3x sepekan	>3x sehari	1-3x sepekan
Partikel tidak be	Kurang dari 1 m	Produksi industri	Air Tawar, Air La	Ya	Ya	Mulut (saluran c	>3x sehari	6	1-3x sebulan	2	8	>3x sehari	Tidak pernah	1-3x sebulan	1-3x sebulan	>3x sehari	1-3x sebulan	<1x sebulan	1-3x sebulan	1-3x sehari	1-3x sebulan	>3x sehari
Partikel bulat, Pa	Kurang dari 1 m	Produksi industri	Air Tawar, Air La	Ya	Tidak	Mulut (saluran c	>3x sehari	6	1-3x sebulan	2	8	>3x sehari	Tidak pernah	>3x sehari	1-3x sepekan	1-3x sepekan	1-3x sebulan	Tidak pernah	1-3x sebulan	>3x sehari	Tidak pernah	>3x sehari
Partikel tidak be	Lebih dari 10 cm	Penggunaan pro	Udara, Produk h	Ya	Ya	Mulut (saluran c	>3x sehari	6	1-3x sehari	5	11	1-3x sepekan	Tidak pernah	Tidak pernah	Tidak pernah	Tidak pernah	Tidak pernah	Tidak pernah	1-3x sepekan	1-3x sehari	Tidak pernah	1-3x sepekan
Partikel bulat	Sekitar 1 - 5 cm	Penggunaan pro	Produk hewani s	Ya	Ya	Mulut (saluran c	>3x sehari	6	1-3x sebulan	2	8	<1x sebulan	Tidak pernah	Tidak pernah	>3x sehari	Tidak pernah	Tidak pernah	Tidak pernah	Tidak pernah	Tidak pernah	Tidak pernah	Tidak pernah
Partikel Bulat	Kurang dari 1 m	Produksi industri	Air Tawar, Air La	Ya	Ya	Mulut (saluran c	1-3x sehari	5	1-3x sepekan	3	8	1-3x sehari	Tidak pernah	1-3x sepekan	1-3x sebulan	4-6x sepekan	1-3x sepekan	Tidak pernah	1-3x sepekan	1-3x sepekan	1-3x sepekan	1-3x sepekan
Film	Sekitar 1 - 5 cm	Penggunaan pro	Air Laut	Ya	Tidak	Mulut (saluran c	1-3x sehari	5	1-3x sepekan	3	8	Tidak pernah	Tidak pernah	1-3x sehari	4-6x sepekan	4-6x sepekan	1-3x sehari	Tidak pernah	>3x sehari	4-6x sepekan	Tidak pernah	1-3x sehari
Partikel tidak be	Kurang dari 1 m	Penggunaan pro	Air Laut	Ya	Tidak	Mulut (saluran c	1-3x sehari	5	1-3x sepekan	3	8	1-3x sehari	Tidak pernah	1-3x sepekan	<1x sebulan	<1x sebulan	<1x sebulan	Tidak pernah	Tidak pernah	1-3x sehari	<1x sebulan	1-3x sehari
Partikel tidak be	Kurang dari 1 m	Penggunaan pro	Udara	Ya	Ya	Mulut (saluran c	1-3x sehari	5	1-3x sepekan	3	8	>3x sehari	1-3x sehari	1-3x sepekan	4-6x sepekan	1-3x sehari	4-6x sepekan	1-3x sebulan	1-3x sepekan	1-3x sehari	<1x sebulan	<1x sebulan
Serat	Kurang dari 1 m	Produksi industri	Air Tawar, Air La	Ya	Ya	Mulut (saluran c	1-3x sehari	5	1-3x sepekan	3	8	1-3x sehari	1-3x sepekan	<1x sebulan	<1x sebulan	4-6x sepekan	>3x sehari	<1x sebulan	<1x sebulan	Tidak pernah	Tidak pernah	Tidak pernah
Serat, Partikel b	Sekitar 1 - 5 cm	Produksi industri	Air Tawar, Air La	Ya	Tidak	Mulut (saluran c	1-3x sehari	5	1-3x sepekan	3	8	>3x sehari	Tidak pernah	1-3x sepekan	1-3x sehari	1-3x sebulan	1-3x sebulan	Tidak pernah	1-3x sepekan	1-3x sehari	1-3x sepekan	1-3x sehari
Serat, Partikel ti	Sekitar 1 - 5 cm	Produksi industri	Air Tawar, Air La	Ya	Ya	Mulut (saluran c	1-3x sehari	5	1-3x sepekan	3	8	1-3x sehari	Tidak pernah	1-3x sehari	1-3x sehari	1-3x sehari	1-3x sebulan	<1x sebulan	1-3x sehari	4-6x sepekan	1-3x sehari	4-6x sepekan
Serat, Partikel ti	Kurang dari 1 m	Produksi industri	Air Tawar, Air La	Ya	Tidak	Mulut (saluran c	1-3x sehari	5	1-3x sepekan	3	8	1-3x sepekan	1-3x sehari	1-3x sebulan	4-6x sepekan	1-3x sehari	1-3x sepekan	<1x sebulan	<1x sebulan	4-6x sepekan	<1x sebulan	4-6x sepekan
Partikel tidak be	Kurang dari 1 m	Produksi industri	Air Laut, Udara,	Tidak tahu	Tidak	Mulut (saluran c	1-3x sehari	5	1-3x sepekan	3	8	1-3x sebulan	Tidak pernah	1-3x sebulan	1-3x sehari	1-3x sebulan	1-3x sehari	Tidak pernah	Tidak pernah	Tidak pernah	Tidak pernah	Tidak pernah
Partikel tidak be	Kurang dari 1 m	Penggunaan pro	Air Laut, Tanah,	Ya	Tidak	Mulut (saluran c	1-3x sehari	5	1-3x sepekan	3	8	>3x sehari	1-3x sebulan	1-3x sepekan	4-6x sepekan	<1x sebulan	<1x sebulan	<1x sebulan	<1x sebulan	4-6x sepekan	1-3x sebulan	>3x sehari
Partikel bulat, Pa	Kurang dari 1 m	Produksi industri	Air Laut, Udara	Ya	Ya	Mulut (saluran c	1-3x sehari	5	1-3x sepekan	3	8	<1x sebulan	1-3x sepekan	1-3x sepekan	4-6x sepekan	1-3x sebulan	1-3x sebulan	1-3x sebulan	1-3x sepekan	1-3x sebulan	1-3x sebulan	1-3x sebulan
Partikel tidak be	Kurang dari 1 m	Produksi industri	Air Tawar, Air La	Ya	Ya	Mulut (saluran c	1-3x sehari	5	1-3x sepekan	3	8	1-3x sepekan	Tidak pernah	1-3x sebulan	1-3x sepekan	1-3x sehari	1-3x sebulan	4-6x sepekan	1-3x sepekan	1-3x sebulan	<1x sebulan	Tidak pernah
Serat, Partikel b	Sekitar 1 - 5 cm	Pelepasan serat	Air Tawar, Air La	Ya	Ya	Mulut (saluran c	1-3x sehari	5	1-3x sepekan	3	8	1-3x sepekan	1-3x sehari	1-3x sehari	1-3x sehari	1-3x sehari	Tidak pernah	1-3x sehari	1-3x sepekan	1-3x sehari	Tidak pernah	1-3x sehari
Serat, Partikel B	Kurang dari 1 m	Produksi industri	Air Tawar, Air La	Tidak Tahu	Ya	Mulut (saluran c	4-6x sepekan	4	4-6x sepekan	4	8	>3x sehari	Tidak pernah	>3x sehari	4-6x sepekan	4-6x sepekan	1-3x sehari	1-3x sepekan	4-6x sepekan	1-3x sehari	1-3x sebulan	>3x sehari
Partikel Bulat, Pa	Kurang dari 1 m	Pencucian pakai	Air Laut, Udara,	Ya	Ya	Mulut (saluran c	4-6x sepekan	4	4-6x sepekan	4	8	1-3x sepekan	Tidak pernah	>3x sehari	4-6x sepekan	4-6x sepekan	4-6x sepekan	<1x sebulan	<1x sebulan	<1x sebulan	<1x sebulan	4-6x sepekan
Serat	Kurang dari 1 m	Produksi industri	Air Tawar, Air La	Ya	Ya	Mulut (saluran c	4-6x sepekan	4	4-6x sepekan	4	8	1-3x sehari	Tidak pernah	1-3x sepekan	1-3x sehari	1-3x sehari	1-3x sepekan	>3x sehari	1-3x sehari	4-6x sepekan	<1x sebulan	>3x sehari
Serat	Sekitar 1 - 5 cm	Penggunaan pro	Air Laut, Biota L	Ya	Ya	Mulut (saluran c	4-6x sepekan	4	4-6x sepekan	4	8	1-3x sebulan	1-3x sebulan	Tidak pernah	Tidak pernah	4-6x sepekan	4-6x sepekan	4-6x sepekan	4-6x sepekan	4-6x sepekan	4-6x sepekan	4-6x sepekan
Partikel Tidak Be	Kurang dari 1 m	Penggunaan pro	Biota Laut, Prod	Ya	Tidak	Mulut (saluran c	4-6x sepekan	4	4-6x sepekan	4	8	Tidak pernah	4-6x sepekan	1-3x sebulan	1-3x sepekan	4-6x sepekan	1-3x sepekan	1-3x sepekan	1-3x sepekan	1-3x sepekan	1-3x sepekan	1-3x sepekan
Partikel tidak be	Sekitar 1 - 5 cm	Produksi industri	Air Laut, Tanah,	Ya	Tidak	Mulut (saluran c	4-6x sepekan	4	4-6x sepekan	4	8	<1x sebulan	Tidak pernah	1-3x sehari	<1x sebulan	4-6x sepekan	<1x sebulan	<1x sebulan	1-3x sepekan	4-6x sepekan	<1x sebulan	<1x sebulan
Serat, Partikel b	Kurang dari 1 m	Produksi industri	Biota Laut	Tidak tahu	Tidak	Hidung (Saluran	4-6x sepekan	4	4-6x sepekan	4	8	>3x sehari	Tidak pernah	Tidak pernah	>3x sehari	<1x sebulan	4-6x sepekan	1-3x sepekan	1-3x sebulan	<1x sebulan	1-3x sepekan	1-3x sepekan
Serat, Partikel b	Kurang dari 1 m	Produksi industri	Air Tawar, Air La	Ya	Ya	Mulut (saluran c	4-6x sepekan	4	4-6x sepekan	4	8	4-6x sepekan	Tidak pernah	4-6x sepekan	1-3x sepekan	1-3x sepekan	1-3x sepekan	1-3x sepekan	1-3x sebulan	1-3x sehari	<1x sebulan	1-3x sepekan
Partikel tidak be	Kurang dari 1 m	Produksi industri	Air Tawar, Air La	Ya	Ya	Mulut (saluran c	4-6x sepekan	4	4-6x sepekan	4	8	1-3x sepekan	Tidak pernah	4-6x sepekan	4-6x sepekan	1-3x sebulan	4-6x sepekan	<1x sebulan	4-6x sepekan	4-6x sepekan	4-6x sepekan	4-6x sepekan
Partikel bulat, Pa	Kurang dari 1 m	Penggunaan pro	Tanah	Ya	Ya	Mulut (saluran c	4-6x sepekan	4	4-6x sepekan	4	8	4-6x sepekan	4-6x sepekan	4-6x sepekan	<1x sebulan	4-6x sepekan	4-6x sepekan	1-3x sepekan	Tidak pernah	Tidak pernah	Tidak pernah	Tidak pernah
Serat, Partikel b	Kurang dari 1 m	Pelepasan serat	Air Tawar	Tidak tahu	Tidak	Hidung (Saluran	4-6x sepekan	4	4-6x sepekan	4	8	1-3x sepekan	1-3x sehari	1-3x sepekan	1-3x sehari	>3x sehari	Tidak pernah	Tidak pernah	Tidak pernah	Tidak pernah	Tidak pernah	1-3x sebulan
Serat, Partikel b	Kurang dari 1 m	Penggunaan pro	Air Tawar, Air La	Ya	Tidak	Mulut (saluran c	4-6x sepekan	4	4-6x sepekan	4	8	Tidak pernah	4-6x sepekan	1-3x sebulan	4-6x sepekan	<1x sebulan	<1x sebulan	Tidak pernah	4-6x sepekan	4-6x sepekan	4-6x sepekan	1-3x sebulan
Partikel tidak be	Kurang dari 1 m	Penggunaan pro	Air Laut	Ya	Tidak	Mulut (saluran c	4-6x sepekan	4	4-6x sepekan	4	8	1-3x sepekan	Tidak pernah	1-3x sepekan	4-6x sepekan	1-3x sebulan	1-3x sebulan	1-3x sebulan	1-3x sebulan	Tidak pernah	Tidak pernah	Tidak pernah
Serat	Kurang dari 1 m	Produksi industri	Air Tawar, Air La	Ya	Ya	Mulut (saluran c	4-6x sepekan	4	4-6x sepekan	4	8	>3x sehari	Tidak pernah	4-6x sepekan	1-3x sebulan	>3x sehari	1-3x sehari	<1x sebulan	4-6x sepekan	<1x sebulan	Tidak pernah	1-3x sehari
Serat	Kurang dari 1 m	Penggunaan pro	Air Tawar, Air La	Tidak tahu	Ya	Mulut (saluran c	4-6x sepekan	4	4-6x sepekan	4	8	>3x sehari	Tidak pernah	4-6x sepekan	1-3x sehari	4-6x sepekan	4-6x sepekan	Tidak pernah	4-6x sepekan	4-6x sepekan	<1x sebulan	1-3x sehari
Serat, Partikel b	Kurang dari 1 m	Pelepasan serat	Air Tawar, Air La	Ya	Ya	Mulut (saluran c	1-3x sepekan	3	1-3x sepekan	3	6	>3x sehari	Tidak pernah	4-6x sepekan	1-3x sehari	4-6x sepekan	1-3x sepekan	Tidak pernah	4-6x sepekan	4-6x sepekan	1-3x sebulan	>3x sehari
Film	Kurang dari 1 m	Produksi industri	Air Laut	Ya	Tidak	Mulut (saluran c	4-6x sepekan	4	4-6x sepekan	4	8	>3x sehari	Tidak pernah	Tidak pernah	4-6x sepekan	Tidak pernah	Tidak pernah	Tidak pernah	Tidak pernah	Tidak pernah	Tidak pernah	Tidak pernah
Partikel tidak be	Kurang dari 1 m	Penggunaan pro	Air Tawar	Ya	Tidak	Mulut (saluran c	4-6x sepekan	4	4-6x sepekan	4	8	>3x sehari	1-3x sehari	4-6x sepekan	1-3x sepekan	1-3x sepekan	1-3x sepekan	Tidak pernah	4-6x sepekan	1-3x sehari	4-6x sepekan	1-3x sehari
Serat, Partikel ti	Kurang dari 1 m	Produksi industri	Air Laut, Biota L	Tidak tahu	Ya	Mulut (saluran c	4-6x sepekan	4	4-6x sepekan	4	8	>3x sehari	Tidak pernah	<1x sebulan	4-6x sepekan	1-3x sepekan	4-6x sepekan	4-6x sepekan	4-6x sepekan	4-6x sepekan	4-6x sepekan	4-6x sepekan
Partikel tidak be	Kurang dari 1 m	Penggunaan pro	Air Laut, Biota L	Ya	Ya	Mulut (saluran c	4-6x sepekan	4	4-6x sepekan	4	8	Tidak pernah	1-3x sebulan	1-3x sehari	4-6x sepekan	1-3x sepekan	1-3x sehari	Tidak pernah	1-3x sehari	1-3x sehari	1-3x sehari	<1x sebulan
Film	Kurang dari 1 m	Penggunaan pro	Air Laut	Ya	Ya	Mulut (saluran c	1-3x sepekan	3	1-3x sehari	5	8	1-3x sehari	1-3x sehari	1-3x sepekan	1-3x sehari	1-3x sepekan	1-3x sepekan	1-3x sepekan	1-3x sepekan	1-3x sepekan	1-3x sepekan	1-3x sepekan
Partikel Tidak Be	Kurang dari 1 m	Pelepasan serat	Air Tawar, Air La	Ya	Ya	Mulut (saluran c	1-3x sepekan	3	1-3x sehari	5	8	>3x sehari	Tidak pernah	1-3x sehari	1-3x sehari	1-3x sehari	1-3x sehari	1-3x sehari	1-3x sehari	4-6x sepekan	1-3x sepekan	1-3x sebulan
Serat	Kurang dari 1 m	Penggunaan pro	Tanah	Tidak tahu	Tidak	Kulit	1-3x sepekan	3	1-3x sehari	5	8	1-3x sepekan	1-3x sepekan	Tidak pernah	Tidak pernah	1-3x sepekan	1-3x sebulan	<1x sebulan	1-3x sepekan	<1x sebulan	<1x sebulan	1-3x sepekan
Serat	Kurang dari 1 m	Produksi industri	Air Tawar	Ya	Tidak	Mulut (saluran c	1-3x sepekan	3	1-3x sehari	5	8	<1x sebulan	Tidak pernah	<1x sebulan	Tidak pernah	1-3x sehari	<1x sebulan	Tidak pernah	1-3x sehari	<1x sebulan	<1x sebulan	<1x sebulan
Partikel tidak be	Kurang dari 1 m	Produksi industri	Air Tawar, Air La	Ya	Ya	Mulut (saluran c	1-3x sepekan	3	1-3x sehari	5	8	<1x sebulan	Tidak pernah	1-3x sebulan	1-3x sehari	1-3x sepekan	<1x sebulan	1-3x sebulan	4-6x sepekan	<1x sebulan	1-3x sebulan	<1x sebulan
Partikel bulat	Kurang dari 1 m	Produksi industri	Air Tawar, Air La	Ya	Tidak	Mulut (saluran c	1-3x sepekan	3	1-3x sehari	5	8	1-3x sebulan	>3x sehari	1-3x sepekan	1-3x sehari	1-3x sepekan	1-3x sebulan	Tidak pernah	4-6x sepekan	4-6x sepekan	<1x sebulan	1-3x sebulan

Serat, Partikel tid	Kurang dari 1 mi	Produksi industri	Air Laut, Tanah,	Ya	Tidak	Mulut (saluran c	1-3x sepekan	3	1-3x sehari	5	8	1-3x sebulan	1-3x sebulan	1-3x sebulan	1-3x sepekan	1-3x sepekan	1-3x sebulan	1-3x sebulan	1-3x sebulan	<1x sebulan	1-3x sebulan	1-3x sepekan
Partikel tidak ber	Kurang dari 1 mi	Produksi industri	Air Tawar, Air La	Ya	Ya	Mulut (saluran c	1-3x sepekan	3	1-3x sehari	5	8	>3x sehari	>3x sehari	4-6x sepekan	1-3x sehari	4-6x sepekan	1-3x sebulan	1-3x sehari	>3x sehari	1-3x sehari	1-3x sebulan	>3x sehari
Serat, Partikel bu	Kurang dari 1 mi	Produksi industri	Air Laut, Udara,	Ya	Tidak	Mulut (saluran c	1-3x sepekan	3	1-3x sehari	5	8	>3x sehari	Tidak pernah	1-3x sepekan	1-3x sebulan	1-3x sepekan	1-3x sepekan	1-3x sebulan	1-3x sehari	1-3x sepekan	1-3x sepekan	4-6x sepekan
Partikel tidak ber	Kurang dari 1 mi	Produksi industri	Air Laut, Tanah,	Ya	Ya	Mulut (saluran c	1-3x sebulan	2	>3x sehari	6	8	>3x sehari	Tidak pernah	4-6x sepekan	>3x sehari	1-3x sebulan	4-6x sepekan	>3x sehari	1-3x sebulan	>3x sehari	1-3x sebulan	>3x sehari
Serat	Kurang dari 1 mi	Penggunaan pro	Udara	Tidak Tahu	Tidak	Hidung (Saluran	>3x sehari	6	1-3x sepekan	3	9	>3x sehari	4-6x sepekan	1-3x sebulan	1-3x sebulan	>3x sehari	1-3x sepekan	<1x sebulan	1-3x sehari	1-3x sehari	1-3x sebulan	>3x sehari
Partikel tidak ber	Kurang dari 1 mi	Penggunaan pro	Air Laut	Ya	Ya	Mulut (saluran c	>3x sehari	6	1-3x sepekan	3	9	>3x sehari	Tidak pernah	1-3x sepekan	1-3x sebulan	1-3x sepekan	1-3x sebulan	<1x sebulan	1-3x sepekan	>3x sehari	<1x sebulan	>3x sehari
Partikel bulat, Pa	Kurang dari 1 mi	Produksi industri	Air Tawar, Air La	Ya	Ya	Mulut (saluran c	>3x sehari	6	1-3x sepekan	3	9	>3x sehari	Tidak pernah	<1x sebulan	<1x sebulan	<1x sebulan	4-6x sepekan	Tidak pernah	1-3x sebulan	4-6x sepekan	1-3x sebulan	>3x sehari
Partikel bulat	Kurang dari 1 mi	Penggunaan pro	Tanah	Ya	Ya	Kulit	>3x sehari	6	1-3x sepekan	3	9	>3x sehari	>3x sehari	4-6x sepekan	<1x sebulan	4-6x sepekan	<1x sebulan	Tidak pernah	1-3x sepekan	Tidak pernah	1-3x sepekan	4-6x sepekan
Serat	Kurang dari 1 mi	Produksi industri	Produk hewani s	Ya	Tidak	Hidung (Saluran	>3x sehari	6	1-3x sepekan	3	9	>3x sehari	Tidak pernah	1-3x sebulan	4-6x sepekan	1-3x sebulan	1-3x sebulan	1-3x sebulan	1-3x sebulan	1-3x sebulan	1-3x sebulan	1-3x sebulan
Partikel bulat	Kurang dari 1 mi	Produksi industri	Tanah	Ya	Tidak	Mulut (saluran c	>3x sehari	6	1-3x sepekan	3	9	>3x sehari	>3x sehari	1-3x sepekan	Tidak pernah	1-3x sehari	1-3x sepekan	1-3x sepekan	1-3x sebulan	Tidak pernah	Tidak pernah	<1x sebulan
Partikel tidak ber	Kurang dari 1 mi	Produksi industri	Air Laut, Tanah,	Ya	Ya	Mulut (saluran c	>3x sehari	6	1-3x sepekan	3	9	1-3x sepekan	>3x sehari	>3x sehari	1-3x sehari	1-3x sebulan	1-3x sepekan	1-3x sehari	1-3x sehari	1-3x sehari	1-3x sehari	1-3x sehari
Serat, Partikel bu	Kurang dari 1 mi	Produksi industri	Air Tawar, Air La	Ya	Ya	Mulut (saluran c	>3x sehari	6	1-3x sepekan	3	9	>3x sehari	Tidak pernah	1-3x sepekan	1-3x sepekan	1-3x sebulan	1-3x sebulan	<1x sebulan	1-3x sebulan	>3x sehari	1-3x sebulan	>3x sehari
Serat, Partikel bu	Kurang dari 1 mi	Produksi industri	Air Tawar, Air La	Ya	Ya	Mulut (saluran c	>3x sehari	6	1-3x sepekan	3	9	>3x sehari	<1x sebulan	1-3x sehari	4-6x sepekan	<1x sebulan	1-3x sebulan	<1x sebulan	1-3x sebulan	1-3x sehari	1-3x sebulan	>3x sehari
Partikel tidak ber	Kurang dari 1 mi	Produksi industri	Air Tawar, Udara	Ya	Tidak	Mulut (saluran c	>3x sehari	6	1-3x sepekan	3	9	>3x sehari	Tidak pernah	1-3x sepekan	1-3x sepekan	>3x sehari	1-3x sepekan	1-3x sebulan	4-6x sepekan	>3x sehari	1-3x sebulan	>3x sehari
Serat, Partikel bu	Kurang dari 1 mi	Produksi industri	Air Tawar, Udara	Ya	Ya	Mulut (saluran c	>3x sehari	6	1-3x sepekan	3	9	1-3x sehari	1-3x sebulan	1-3x sepekan	1-3x sepekan	1-3x sebulan	1-3x sepekan	<1x sebulan	1-3x sebulan	4-6x sepekan	1-3x sebulan	1-3x sebulan
Partikel tidak ber	Sekitar 1 - 5 cm	Penggunaan pro	Produk hewani s	Ya	Tidak	Kulit	>3x sehari	6	1-3x sehari	5	11	Tidak pernah	Tidak pernah	Tidak pernah	Tidak pernah	>3x sehari	1-3x sepekan	1-3x sehari	1-3x sepekan	Tidak pernah	<1x sebulan	Tidak pernah
Partikel bulat	Kurang dari 1 mi	Produksi industri	Air Laut, Udara	Ya	Ya	Mulut (saluran c	>3x sehari	6	1-3x sehari	5	11	Tidak pernah	1-3x sehari	>3x sehari	Tidak pernah	>3x sehari	Tidak pernah	Tidak pernah	Tidak pernah	Tidak pernah	Tidak pernah	Tidak pernah
Serat, Partikel tid	Kurang dari 1 mi	Pelepasan serat	Biota Laut	Ya	Ya	Mulut (saluran c	>3x sehari	6	1-3x sepekan	3	9	1-3x sepekan	1-3x sepekan	1-3x sepekan	Tidak pernah	1-3x sepekan	1-3x sebulan	<1x sebulan	4-6x sepekan	1-3x sepekan	1-3x sepekan	1-3x sepekan
Partikel Tidak Be	Kurang dari 1 mi	Produksi industri	Air Tawar, Air La	Ya	Tidak	Mulut (saluran c	1-3x sehari	5	4-6x sepekan	4	9	>3x sehari	Tidak pernah	4-6x sepekan	4-6x sepekan	4-6x sepekan	1-3x sepekan	1-3x sepekan	1-3x sehari	1-3x sehari	1-3x sepekan	1-3x sehari
Serat	Kurang dari 1 mi	Produksi industri	Air Tawar	Tidak	Ya	Mulut (saluran c	1-3x sehari	5	4-6x sepekan	4	9	1-3x sebulan	1-3x sebulan	Tidak pernah	4-6x sepekan	1-3x sepekan	1-3x sepekan	1-3x sepekan	1-3x sepekan	1-3x sepekan	1-3x sepekan	1-3x sepekan
Partikel bulat	Kurang dari 1 mi	Produksi industri	Air Laut	Ya	Ya	Hidung (Saluran	1-3x sehari	5	4-6x sepekan	4	9	4-6x sepekan	1-3x sebulan	1-3x sehari	1-3x sehari	1-3x sepekan	>3x sehari	>3x sehari	1-3x sehari	<1x sebulan	>3x sehari	4-6x sepekan
Serat	Kurang dari 1 mi	Pelepasan serat	Tanah	Ya	Tidak	Mulut (saluran c	1-3x sehari	5	4-6x sepekan	4	9	1-3x sepekan	Tidak pernah	Tidak pernah	1-3x sehari	1-3x sepekan	1-3x sebulan	Tidak pernah	1-3x sepekan	4-6x sepekan	Tidak pernah	1-3x sepekan
Serat, Partikel bu	Kurang dari 1 mi	Produksi industri	Air Tawar, Air La	Tidak tahu	Ya	Mulut (saluran c	1-3x sehari	5	4-6x sepekan	4	9	>3x sehari	<1x sebulan	4-6x sepekan	1-3x sepekan	1-3x sehari	1-3x sepekan	1-3x sebulan	4-6x sepekan	4-6x sepekan	1-3x sebulan	>3x sehari
Serat, Partikel bu	Kurang dari 1 mi	Produksi industri	Air Tawar, Air La	Ya	Ya	Mulut (saluran c	1-3x sehari	5	4-6x sepekan	4	9	>3x sehari	Tidak pernah	4-6x sepekan	1-3x sepekan	4-6x sepekan	1-3x sebulan	Tidak pernah	1-3x sepekan	1-3x sepekan	4-6x sepekan	4-6x sepekan
Serat	Kurang dari 1 mi	Produksi industri	Air Laut, Udara,	Ya	Tidak	Hidung (Saluran	1-3x sehari	5	4-6x sepekan	4	9	1-3x sebulan	Tidak pernah	1-3x sepekan	1-3x sebulan	1-3x sepekan	Tidak pernah	Tidak pernah	Tidak pernah	1-3x sebulan	1-3x sepekan	1-3x sebulan
Serat, Partikel bu	Sekitar 1 - 5 cm	Penggunaan pro	Air Tawar	Tidak tahu	Tidak	Mulut (saluran c	1-3x sehari	5	4-6x sepekan	4	9	>3x sehari	Tidak pernah	4-6x sepekan	4-6x sepekan	4-6x sepekan	4-6x sepekan	4-6x sepekan	4-6x sepekan	4-6x sepekan	4-6x sepekan	1-3x sebulan
Partikel tidak ber	Kurang dari 1 mi	Pelepasan serat	Air Tawar, Air La	Ya	Ya	Mulut (saluran c	1-3x sehari	5	4-6x sepekan	4	9	>3x sehari	Tidak pernah	1-3x sepekan	<1x sebulan	1-3x sepekan	1-3x sehari	Tidak pernah	1-3x sebulan	4-6x sepekan	1-3x sepekan	>3x sehari
Partikel bulat, Pa	Kurang dari 1 mi	Produksi industri	Air Tawar, Air La	Ya	Ya	Mulut (saluran c	1-3x sehari	5	4-6x sepekan	4	9	1-3x sebulan	>3x sehari	Tidak pernah	1-3x sepekan	4-6x sepekan	1-3x sepekan	Tidak pernah	1-3x sehari	1-3x sebulan	<1x sebulan	<1x sebulan
Serat, Partikel tid	Kurang dari 1 mi	Produksi industri	Air Tawar, Air La	Ya	Ya	Mulut (saluran c	1-3x sehari	5	4-6x sepekan	4	9	>3x sehari	>3x sehari	1-3x sepekan	4-6x sepekan	4-6x sepekan	1-3x sebulan	1-3x sepekan	4-6x sepekan	4-6x sepekan	1-3x sepekan	1-3x sehari
Partikel tidak ber	Kurang dari 1 mi	Pelepasan serat	Air Tawar, Air La	Ya	Ya	Mulut (saluran c	1-3x sehari	5	4-6x sepekan	4	9	1-3x sebulan	Tidak pernah	Tidak pernah	Tidak pernah	4-6x sepekan	1-3x sepekan	<1x sebulan	1-3x sehari	>3x sehari	1-3x sebulan	>3x sehari
Partikel Tidak Be	Kurang dari 1 mi	Produksi industri	Air Tawar, Air La	Ya	Tidak	Mulut (saluran c	4-6x sepekan	4	1-3x sehari	5	9	1-3x sehari	1-3x sehari	1-3x sehari	1-3x sehari	1-3x sepekan	4-6x sepekan	4-6x sepekan	1-3x sehari	1-3x sehari	4-6x sepekan	1-3x sehari
Serat, Partikel B	Kurang dari 1 mi	Pelepasan serat	Air Tawar, Air La	Ya	Ya	Mulut (saluran c	4-6x sepekan	4	1-3x sehari	5	9	>3x sehari	1-3x sehari	1-3x sepekan	1-3x sehari	4-6x sepekan	4-6x sepekan	4-6x sepekan	<1x sebulan	Tidak pernah	<1x sebulan	Tidak pernah
Partikel Bulat	Sekitar 1 - 5 cm	Produksi industri	Air Laut, Tanah,	Ya	Ya	Hidung (Saluran	4-6x sepekan	4	1-3x sehari	5	9	1-3x sehari	4-6x sepekan	1-3x sepekan	1-3x sehari	1-3x sebulan	1-3x sepekan	1-3x sepekan	1-3x sehari	1-3x sepekan	1-3x sepekan	1-3x sepekan
Serat, Partikel bu	Kurang dari 1 mi	Produksi industri	Air Laut	Tidak tahu	Tidak	Mulut (saluran c	4-6x sepekan	4	1-3x sehari	5	9	>3x sehari	Tidak pernah	4-6x sepekan	1-3x sepekan	1-3x sebulan	Tidak pernah	Tidak pernah	1-3x sebulan	4-6x sepekan	Tidak pernah	Tidak pernah
Serat, Partikel bu	Kurang dari 1 mi	Produksi industri	Air Tawar, Air La	Ya	Ya	Mulut (saluran c	4-6x sepekan	4	1-3x sehari	5	9	>3x sehari	Tidak pernah	1-3x sebulan	<1x sebulan	1-3x sehari	4-6x sepekan	Tidak pernah	<1x sebulan	4-6x sepekan	Tidak pernah	1-3x sepekan
Serat	Kurang dari 1 mi	Pencucian pakai	Air Tawar	Ya	Ya	Mulut (saluran c	4-6x sepekan	4	1-3x sehari	5	9	4-6x sepekan	Tidak pernah	Tidak pernah	1-3x sepekan	1-3x sepekan	1-3x sehari	1-3x sepekan	>3x sehari	1-3x sehari	>3x sehari	4-6x sepekan
Serat, Partikel bu	Kurang dari 1 mi	Produksi industri	Air Tawar, Air La	Ya	Ya	Mulut (saluran c	4-6x sepekan	4	1-3x sehari	5	9	1-3x sepekan	Tidak pernah	4-6x sepekan	1-3x sehari	1-3x sepekan	1-3x sehari	Tidak pernah	1-3x sebulan	4-6x sepekan	1-3x sehari	1-3x sebulan
Partikel bulat	Kurang dari 1 mi	Penggunaan pro	Air Laut	Ya	Ya	Mulut (saluran c	4-6x sepekan	4	1-3x sehari	5	9	>3x sehari	Tidak pernah	>3x sehari	>3x sehari	1-3x sepekan	1-3x sehari	1-3x sebulan	1-3x sepekan	>3x sehari	1-3x sebulan	1-3x sehari
Partikel bulat, Pa	Kurang dari 1 mi	Produksi industri	Air Tawar, Air La	Ya	Ya	Mulut (saluran c	1-3x sepekan	3	>3x sehari	6	9	1-3x sebulan	Tidak pernah	1-3x sepekan	1-3x sebulan	4-6x sepekan	Tidak pernah	Tidak pernah	Tidak pernah	>3x sehari	Tidak pernah	Tidak pernah
Partikel bulat, Pa	Kurang dari 1 mi	Produksi industri	Garam	Ya	Tidak	Mulut (saluran c	1-3x sepekan	3	>3x sehari	6	9	>3x sehari	Tidak pernah	4-6x sepekan	<1x sebulan	Tidak pernah	Tidak pernah	Tidak pernah	<1x sebulan	<1x sebulan	Tidak pernah	Tidak pernah
Partikel tidak ber	Kurang dari 1 mi	Produksi industri	Air Laut	Ya	Tidak	Mulut (saluran c	1-3x sepekan	3	>3x sehari	6	9	1-3x sepekan	1-3x sehari	>3x sehari	>3x sehari	>3x sehari	>3x sehari	>3x sehari	>3x sehari	>3x sehari	>3x sehari	>3x sehari
Partikel tidak ber	Kurang dari 1 mi	Penggunaan pro	Air Tawar, Air La	Ya	Ya	Mulut (saluran c	1-3x sepekan	3	>3x sehari	6	9	>3x sehari	<1x sebulan	1-3x sehari	>3x sehari	1-3x sepekan	1-3x sepekan	Tidak pernah	>3x sehari	1-3x sehari	1-3x sepekan	>3x sehari
Serat	Kurang dari 1 mi	Produksi industri	Air Tawar	Tidak	Ya	Mulut (saluran c	>3x sehari	6	4-6x sepekan	4	10	>3x sehari	Tidak pernah	Tidak pernah	Tidak pernah	Tidak pernah	Tidak pernah	Tidak pernah	Tidak pernah	Tidak pernah	1-3x sebulan	<1x sebulan
Serat	Kurang dari 1 mi	Pelepasan serat	Air Tawar, Air La	Ya	Ya	Mulut (saluran c	>3x sehari	6	4-6x sepekan	4	10	>3x sehari	Tidak pernah	>3x sehari	1-3x sepekan	1-3x sehari	1-3x sehari	4-6x sepekan	1-3x sehari	1-3x sehari	1-3x sepekan	>3x sehari
Partikel tidak ber	Kurang dari 1 mi	Penggunaan pro	Udara	Tidak tahu	Ya	Hidung (Saluran	>3x sehari	6	4-6x sepekan	4	10	>3x sehari	Tidak pernah	4-6x sepekan	>3x sehari	1-3x sepekan	Tidak pernah	Tidak pernah	>3x sehari	>3x sehari	4-6x sepekan	>3x sehari
Serat, Partikel Ti	Kurang dari 1 mi	Produksi industri	Air Tawar, Air La	Ya	Tidak	Mulut (saluran c	1-3x sehari	5	1-3x sehari	5	10	<1x sebulan	Tidak pernah	Tidak pernah	1-3x sehari	1-3x sehari	Tidak pernah	Tidak pernah	1-3x sehari	1-3x sehari	Tidak pernah	1-3x sehari
Serat	Sekitar 1 - 5 cm	Produksi industri	Tanah	Ya	Tidak	Hidung (Saluran	1-3x sehari	5	1-3x sehari	5	10	1-3x sepekan	1-3x sepekan	1-3x sepekan	1-3x sepekan	1-3x sepekan	1-3x sepekan	<1x sebulan	1-3x sehari	1-3x sehari	<1x sebulan	1-3x sepekan
Partikel Tidak Be	Sekitar 1 - 5 cm	Produksi industri	Air Tawar, Air La	Ya	Tidak	Mulut (saluran c	1-3x sehari	5	1-3x sehari	5	10	>3x sehari	Tidak pernah	1-3x sepekan	1-3x sehari	1-3x sehari	4-6x sepekan	4-6x sepekan	>3x sehari	1-3x sepekan	1-3x sehari	>3x sehari
Serat, Partikel B	Kurang dari 1 mi	Produksi industri	Air Tawar, Air La	Ya	Ya	Mulut (saluran c	1-3x sehari	5	1-3x sehari	5	10	>3x sehari	>3x sehari	1-3x sepekan	1-3x sepekan	1-3x sepekan	4-6x sepekan	1-3x sebulan	1-3x sepekan	1-3x sehari	<1x sebulan	1-3x sebulan
Serat, Partikel bu	Kurang dari 1 mi	Produksi industri	Air Tawar, Air La	Ya	Ya	Mulut (saluran c	1-3x sehari	5	1-3x sehari	5	10	1-3x sepekan	Tidak pernah	1-3x sehari	1-3x sehari	1-3x sehari	1-3x sehari	Tidak pernah	1-3x sehari	1-3x sehari	1-3x sepekan	1-3x sepekan
Serat	Sekitar 1 - 5 cm	Pelepasan serat	Garam	Ya	Ya	Kulit	1-3x sehari	5	1-3x sehari	5	10	Tidak pernah	Tidak pernah	1-3x sehari	4-6x sepekan	Tidak pernah	Tidak pernah	Tidak pernah	Tidak pernah	Tidak pernah	Tidak pernah	Tidak pernah
Serat, Partikel bu	Kurang dari 1 mi	Produksi industri	Air Tawar, Air La	Ya	Tidak	Mulut (saluran c	1-3x sehari	5	1-3x sehari	5	10	1-3x sebulan	>3x sehari	4-6x sepekan	1-3x sehari	1-3x sebulan	4-6x sepekan	Tidak pernah	Tidak pernah	Tidak pernah	Tidak pernah	Tidak pernah
Partikel tidak ber	Kurang dari 1 mi	Produksi industri	Air Laut, Biota La	Ya	Tidak	Mulut (saluran c	1-3x sehari	5	1-3x sehari	5	10	1-3x sehari	1-3x sehari	1-3x sepekan	1-3x sebulan	1-3x sehari	1-3x sehari	1-3x sehari	1-3x sehari	1-3x sehari	1-3x sehari	1-3x sehari
Serat, Partikel bu	Kurang dari 1 mi	Produksi industri	Air Tawar, Air La	Ya	Ya	Mulut (saluran c	1-3x sehari	5	1-3x sehari	5	10	1-3x sehari	Tidak pernah	>3x sehari	1-3x sepekan	1-3x sepekan	Tidak pernah	Tidak pernah	1-3x sepekan	>3x sehari	Tidak pernah	1-3x sehari
Partikel tidak ber	Lebih dari 10 cm	Produksi industri	Tanah	Tidak tahu	Tidak	Mulut (saluran c	1-3x sehari	5	1-3x sehari	5	10	Tidak pernah	1-3x sepekan	1-3x sehari	Tidak pernah	>3x sehari	<1x sebulan	Tidak pernah	1-3x sehari	Tidak pernah	Tidak pernah	Tidak pernah
Serat, Partikel bu	Kurang dari 1 mi	Produksi industri	Air Laut, Biota La	Ya	Ya	Mulut (saluran c	1-3x sehari	5	1-3x sehari	5	10	1-3x sebulan	1-3x sebulan	1-3x sehari	1-3x sehari	1-3x sehari	1-3x sepekan	1-3x sebulan	1-3x sehari	>3x sehari	1-3x sehari	1-3x sehari
Partikel tidak ber	Kurang dari 1 mi	Pelepasan serat	Air Tawar, Udara	Ya	Tidak	Mulut (saluran c	1-3x sehari	5	1-3x sehari	5	10	>3x sehari	>3x sehari	1-3x sehari	1-3x sehari	1-3x sehari	1-3x sehari	1-3x sepekan	>3x sehari	>3x sehari	1-3x sebulan	>3x sehari
Film	Sekitar 1 - 5 cm	Produksi industri	Air Tawar, Air La	Ya	Ya	Mulut (saluran c	1-3x sehari	5	1-3x sehari	5	10	1-3x sebulan	1-3x sebulan	1-3x sehari	1-3x sehari	1-3x sehari	1-3x sehari	1-3x sehari	1-3x sehari	<1x sebulan	<1x sebulan	<1x sebulan
Partikel tidak ber	Kurang dari 1 mi	Penggunaan pro	Udara, Tanah	Ya	Ya	Hidung (Saluran	1-3x sehari	5	1-3x sehari	5	10	>3x sehari	Tidak pernah	<1x sebulan	>3x sehari	1-3x sebulan	1-3x sebulan	<1x sebulan	>3x sehari	1-3x sehari	1-3x sebulan	1-3x sepekan
Serat, Partikel tid	Kurang dari 1 mi	Penggunaan pro	Air Tawar, Air La	Ya	Ya	Mulut (saluran c	1-3x sehari	5	1-3x sehari	5	10	>3x sehari	>3x sehari	>3x sehari	1-3x sehari	1-3x sehari	1-3x sepekan	1-3x sehari	1-3x sehari	1-3x sehari	1-3x sebulan	1-3x sehari
Film	Kurang dari 1 mi	Produksi industri	Air Tawar, Air La	Ya	Ya	Mulut (saluran c	1-3x sehari	5	1-3x sehari	5	10	>3x sehari	Tidak pernah	1-3x sehari	1-3x sepekan	4-6x sepekan	1-3x sehari	Tidak pernah	4-6x sepekan	>3x sehari	4-6x sepekan	>3x sehari
Partikel bulat	Kurang dari 1 mi	Produksi industri	Air Tawar, Air La	Ya	Ya	Mulut (saluran c	1-3x sehari	5	1-3x sehari	5	10	1-3x sepekan	1-3x sepekan	<1x sebulan	1-3x sehari	<1x sebulan	1-3x sebulan	1-3x sebulan	1-3x sebulan	1-3x sebulan	1-3x sebulan	1-3x sebulan
Partikel tidak ber	Kurang dari 1 mi	Produksi industri	Air Tawar	Tidak tahu	Tidak	Mulut (saluran c	1-3x sehari	5	1-3x sehari	5	10	1-3x sehari	Tidak pernah	<1x sebulan	<1x sebulan	<1x sebulan	<1x sebulan	<1x sebulan	<1x sebulan	1-3x sepekan	<1x sebulan	<1x sebulan
Serat, Partikel bu	Kurang dari 1 mi	Produksi industri	Air Tawar	Ya	Tidak	Mulut (saluran c	1-3x sehari	5	1-3x sehari	5	10	1-3x sepekan	>3x sehari	1-3x sehari	1-3x sehari	1-3x sehari	1-3x sehari	1-3x sepekan	1-3x sehari	>3x sehari	1-3x sepekan	1-3x sepekan
Serat	Kurang dari 1 mi	Pelepasan serat	Air Laut	Ya	Ya	Mulut (saluran c	1-3x sehari	5	1-3x sehari	5	10	>3x sehari	1-3x sehari	>3x sehari	>3x sehari	1-3x sehari	1-3x sehari	1-3x sebulan	1-3x sehari	1-3x sehari	1-3x sehari	>3x sehari
Serat	Kurang dari 1 mi	Produksi industri	Air Tawar	Ya	Ya	Kulit	1-3x sehari	5	1-3x sehari	5	10	1-3x sehari	1-3x sehari	1-3x sehari	1-3x sehari	>3x sehari	>3x sehari	>3x sehari	>3x sehari	>3x sehari	>3x sehari	<1x sebulan
Partikel bulat, Pa	Kurang dari 1 mi	Pelepasan serat	Udara, Tanah	Tidak tahu	Tidak	Mulut (saluran c	1-3x sehari	5	1-3x sehari	5	10	Tidak pernah	Tidak pernah	1-3x sebulan	1-3x sebulan	<1x sebulan	1-3x sehari	1-3x sebulan	4-6x sepekan	Tidak pernah	1-3x sepekan	1-3x sebulan
Serat	Kurang dari 1 mi	Pelepasan serat	Air Tawar, Air La	Ya	Ya	Mulut (saluran c	1-3x sehari	5	1-3x sehari	5	10	>3x sehari	1-3x sehari	Tidak pernah	Tidak pernah	1-3x sehari	Tidak pernah	Tidak pernah	Tidak pernah	Tidak pernah	Tidak pernah	Tidak pernah
Serat	Kurang dari 1 mi	Pelepasan serat	Air Tawar, Air La	Ya	Ya	Mulut (saluran c	1-3x sehari	5	1-3x sehari	5	10	1-3x sebulan	Tidak pernah	1-3x sepekan	1-3x sehari	1-3x sehari	1-3x sepekan	1-3x sepekan	1-3x sehari	1-3x sehari	1-3x sehari	4-6x sepekan
Serat	Kurang dari 1 mi	Pencucian pakai	Air Tawar, Air La	Ya	Ya	Mulut (saluran c	1-3x sehari	5	1-3x sehari	5	10	1-3x sehari	Tidak pernah	1-3x sehari	1-3x sehari	1-3x sehari	1-3x sehari	4-6x sepekan	1-3x sehari	1-3x sehari	4-6x sepekan	1-3x sehari
Serat	Kurang dari 1 mi	Penggunaan pro	Air Tawar, Air La	Ya	Ya	Mulut (saluran c	1-3x sehari	5	1-3x sehari	5	10	Tidak pernah	<1x sebulan	4-6x sepekan	4-6x sepekan	>3x sehari	1-3x sehari	Tidak pernah	<1x sebulan	<1x sebulan	Tidak pernah	1-3x sebulan
Partikel bulat, Pa	Kurang dari 1 mi	Produksi industri	Air Tawar, Air La	Ya	Ya	Mulut (saluran c	1-3x sehari	5	1-3x sehari	5	10	>3x sehari	>3x sehari	1-3x sehari	1-3x sehari	1-3x sepekan	1-3x sepekan	Tidak pernah	4-6x sepekan	>3x sehari	1-3x sehari	>3x sehari
Serat	Kurang dari 1 mi	Penggunaan pro	Tanah	Ya	Tidak	Mulut (saluran c	1-3x sehari	5	1-3x sehari	5	10	1-3x sehari	Tidak pernah	1-3x sehari	<1x sebulan	1-3x sepekan	1-3x sepekan	1-3x sepekan	1-3x sehari	1-3x sehari	1-3x sepekan	4-6x sepekan
Serat	Kurang dari 1 mi	Produksi industri	Air Tawar, Air La	Ya	Ya	Mulut (saluran c	1-3x sehari	5	1-3x sehari	5	10	>3x sehari	Tidak pernah	<1x sebulan	1-3x sehari	Tidak pernah	Tidak pernah	Tidak pernah	Tidak pernah	Tidak pernah	Tidak pernah	Tidak pernah
Partikel tidak ber	Kurang dari 1 mi	Penggunaan pro	Produk hewani s	Ya	Ya	Mulut (saluran c	1-3x sehari	5	1-3x sehari	5	10	1-3x sebulan	<1x sebulan	>3x sehari	>3x sehari	>3x sehari	1-3x sehari	1-3x sehari	1-3x sehari	1-3x sehari	1-3x sehari	Tidak pernah
Serat	Kurang dari 1 mi	Penggunaan pro	Air Tawar, Tanah	Ya	Ya	Mulut (saluran c	1-3x sehari	5	1-3x sehari	5	10	>3x sehari	>3x sehari	1-3x sehari	1-3x sepekan	4-6x sepekan	4-6x sepekan	4-6x sepekan	1-3x sehari	1-3x sehari	1-3x sehari	>3x sehari
Serat	Kurang dari 1 mi	Produksi industri	Air Laut, Biota La	Ya	Ya	Mulut (saluran c	1-3x sehari	5	1-3x sehari	5	10	4-6x sepekan	>3x sehari	1-3x sehari	4-6x sepekan	1-3x sehari	>3x sehari	>3x sehari	1-3x sehari	1-3x sehari	1-3x sehari	1-3x sehari
Film	Kurang dari 1 mi	Produksi industri	Air Laut, Tanah,	Ya	Ya	Mulut (saluran c	1-3x sehari	5	1-3x sehari	5	10	>3x sehari	>3x sehari	1-3x sehari	1-3x sehari	4-6x sepekan	1-3x sehari	4-6x sepekan	4-6x sepekan	1-3x sehari	4-6x sepekan	>3x sehari
Serat	Kurang dari 1 mi	Penggunaan pro	Udara	Ya	Tidak	Hidung (Saluran	1-3x sehari	5	1-3x sehari	5	10	<1x sebulan	>3x sehari	1-3x sepekan	1-3x sebulan	1-3x sehari	<1x sebulan	<1x sebulan	1-3x sebulan	1-3x sepekan	1-3x sepekan	1-3x sepekan
Partikel tidak ber	Kurang dari 1 mi	Penggunaan pro	Tanah	Ya	Ya	Mulut (saluran c	1-3x sehari	5	1-3x sehari	5	10	1-3x sebulan	<1x sebulan	4-6x sepekan	1-3x sepekan	1-3x sepekan	1-3x sebulan	1-3x sebulan	1-3x sebulan	1-3x sebulan	1-3x sepekan	1-3x sepekan
Serat	Kurang dari 1 mi	Produksi industri	Air Laut, Udara,	Ya	Ya	Mulut (saluran c	1-3x sehari	5	1-3x sehari	5	10	Tidak pernah	1-3x sebulan	1-3x sepekan	1-3x sebulan	1-3x sepekan	1-3x sepekan	1-3x sepekan	1-3x sepekan	1-3x sebulan	1-3x sepekan	1-3x sepekan
Partikel tidak ber	Kurang dari 1 mi	Produksi industri	Air Laut, Biota La	Ya	Ya	Mulut (saluran c	1-3x sebulan	2	1-3x sebulan	2	4	Tidak pernah	Tidak pernah	1-3x sehari	1-3x sehari	1-3x sehari	Tidak pernah	Tidak pernah	1-3x sehari	1-3x sepekan	Tidak pernah	Tidak pernah
Film	Kurang dari 1 mi	Produksi industri	Air Tawar, Air La	Ya	Ya	Mulut (saluran c	1-3x sehari	5	1-3x sehari	5	10	1-3x sepekan	>3x sehari	4-6x sepekan	1-3x sepekan	1-3x sepekan	1-3x sebulan	Tidak pernah	1-3x sepekan	1-3x sepekan	Tidak pernah	1-3x sepekan
Serat	Kurang dari 1 mi	Pelepasan serat	Air Tawar	Ya	Ya	Mulut (saluran c	1-3x sehari	5	1-3x sehari	5	10	>3x sehari	>3x sehari	1-3x sepekan	4-6x sepekan	1-3x sepekan	1-3x sepekan	1-3x sepekan	4-6x sepekan	1-3x sehari	1-3x sepekan	4-6x sepekan
Serat, Partikel bu	Kurang dari 1 mi	Produksi industri	Air Tawar, Air La	Ya	Ya	Mulut (saluran c	1-3x sehari	5	1-3x sehari	5	10	>3x sehari	1-3x sehari	1-3x sepekan	1-3x sepekan	1-3x sepekan	1-3x sebulan	<1x sebulan	<1x sebulan	<1x sebulan	<1x sebulan	1-3x sehari
Partikel bulat	Lebih dari 10 cm	Produksi industri	Air Tawar	Ya	Tidak	Mulut (saluran c	1-3x sehari	5	1-3x sehari	5	10	1-3x sepekan	1-3x sehari	Tidak pernah	>3x sehari	1-3x sehari	1-3x sehari	4-6x sepekan	>3x sehari	>3x sehari	1-3x sehari	4-6x sepekan
Partikel tidak ber	Kurang dari 1 mi	Produksi industri	Air Tawar, Air La	Ya	Ya	Mulut (saluran c	1-3x sehari	5	1-3x sehari	5	10	1-3x sebulan	1-3x sebulan	1-3x sepekan	1-3x sepekan	1-3x sepekan	1-3x sepekan	<1x sebulan	1-3x sebulan	1-3x sebulan	1-3x sebulan	<1x sebulan
Serat, Partikel bu	Kurang dari 1 mi	Penggunaan pro	Udara	Tidak tahu	Tidak	Kulit	4-6x sepekan	4	>3x sehari	6	10	1-3x sebulan	Tidak pernah	Tidak pernah	Tidak pernah	>3x sehari	1-3x sepekan	1-3x sebulan	4-6x sepekan	>3x sehari	Tidak pernah	>3x sehari
Serat, Partikel B	Lebih dari 10 cm	Produksi industri	Air Tawar, Air La	Ya	Ya	Mulut (saluran c	>3x sehari	6	1-3x sehari	5	11	>3x sehari	Tidak pernah	<1x sebulan	1-3x sehari	1-3x sepekan	4-6x sepekan	4-6x sepekan	1-3x sepekan	1-3x sehari	4-6x sepekan	1-3x sebulan
Partikel Tidak Be	Kurang dari 1 mi	Produksi industri	Air Tawar, Air La	Ya	Ya	Mulut (saluran c	>3x sehari	6	1-3x sehari	5	11	>3x sehari	<1x sebulan	1-3x sebulan	1-3x sebulan	1-3x sehari	1-3x sehari	Tidak pernah	1-3x sepekan	1-3x sepekan	Tidak pernah	1-3x sepekan
Serat	Kurang dari 1 mi	Penggunaan pro	Tanah	Ya	Tidak	Mulut (saluran c	>3x sehari	6	1-3x sehari	5	11	>3x sehari	Tidak pernah	<1x sebulan	<1x sebulan	<1x sebulan	<1x sebulan	<1x sebulan	<1x sebulan	<1x sebulan	<1x sebulan	<1x sebulan
Serat, Partikel bu	Kurang dari 1 mi	Produksi industri	Air Tawar, Air La	Ya	Ya	Mulut (saluran c	>3x sehari	6	1-3x sehari	5	11	>3x sehari	>3x sehari	>3x sehari	>3x sehari	1-3x sehari	1-3x sehari	1-3x sehari	>3x sehari	>3x sehari	1-3x sehari	>3x sehari
Partikel tidak ber	Kurang dari 1 mi	Penggunaan pro	Air Tawar, Air La	Ya	Ya	Mulut (saluran c	>3x sehari	6	1-3x sehari	5	11	Tidak pernah	1-3x sepekan	1-3x sebulan	4-6x sepekan	4-6x sepekan	1-3x sebulan	>3x sehari	1-3x sehari	>3x sehari	<1x sebulan	>3x sehari
Partikel tidak ber	Kurang dari 1 mi	Produksi industri	Air Tawar, Air La	Ya	Ya	Mulut (saluran c	>3x sehari	6	1-3x sehari	5	11	>3x sehari	Tidak pernah	1-3x sepekan	Tidak pernah	1-3x sehari	1-3x sehari	Tidak pernah	1-3x sepekan	1-3x sehari	1-3x sebulan	1-3x sehari
Partikel bulat	Kurang dari 1 mi	Produksi industri	Air Tawar, Air La	Ya	Ya	Mulut (saluran c	>3x sehari	6	1-3x sehari	5	11	>3x sehari	Tidak pernah	>3x sehari	>3x sehari	>3x sehari	1-3x sehari	Tidak pernah	>3x sehari	>3x sehari	Tidak pernah	>3x sehari
Serat	Kurang dari 1 mi	Produksi industri	Air Tawar, Air La	Ya	Tidak	Mulut (saluran c	>3x sehari	6	1-3x sehari	5	11	>3x sehari	Tidak pernah	>3x sehari	1-3x sebulan	Tidak pernah	1-3x sepekan	Tidak pernah	Tidak pernah	>3x sehari	<1x sebulan	>3x sehari
Serat	Kurang dari 1 mi	Produksi industri	Air Tawar, Air La	Ya	Ya	Mulut (saluran c	>3x sehari	6	1-3x sehari	5	11	>3x sehari	Tidak pernah	1-3x sehari	1-3x sehari	>3x sehari	1-3x sehari	1-3x sehari	4-6x sepekan	4-6x sepekan	4-6x sepekan	>3x sehari
Partikel tidak ber	Kurang dari 1 mi	Pelepasan serat	Air Tawar, Air La	Ya	Ya	Mulut (saluran c	>3x sehari	6	1-3x sehari	5	11	>3x sehari	Tidak pernah	<1x sebulan	4-6x sepekan	1-3x sepekan	1-3x sebulan	1-3x sebulan	1-3x sehari	>3x sehari	Tidak pernah	>3x sehari
Serat, Partikel bu	Kurang dari 1 mi	Produksi industri	Air Tawar, Air La	Ya	Ya	Mulut (saluran c	>3x sehari	6	1-3x sehari	5	11	>3x sehari	<1x sebulan	Tidak pernah	>3x sehari	1-3x sehari	4-6x sepekan	<1x sebulan	>3x sehari	1-3x sehari	1-3x sehari	>3x sehari
Serat	Kurang dari 1 mi	Produksi industri	Air Laut	Ya	Tidak	Mulut (saluran c	>3x sehari	6	1-3x sehari	5	11	>3x sehari	Tidak pernah	Tidak pernah	>3x sehari	1-3x sehari	1-3x sehari	Tidak pernah	1-3x sehari	1-3x sehari	1-3x sehari	>3x sehari
Partikel bulat, Pa	Kurang dari 1 mi	Pencucian pakai	Air Tawar, Produ	Ya	Tidak	Mulut (saluran c	>3x sehari	6	1-3x sehari	5	11	>3x sehari	Tidak pernah	Tidak pernah	>3x sehari	1-3x sehari	1-3x sepekan	<1x sebulan	1-3x sehari	1-3x sehari	4-6x sepekan	>3x sehari
Partikel bulat	Kurang dari 1 mi	Produksi industri	Air Tawar, Air La	Ya	Ya	Mulut (saluran c	>3x sehari	6	1-3x sehari	5	11	>3x sehari	1-3x sehari	>3x sehari	1-3x sepekan	4-6x sepekan	1-3x sehari	1-3x sepekan	4-6x sepekan	4-6x sepekan	1-3x sebulan	>3x sehari
Serat	Kurang dari 1 mi	Pelepasan serat	Garam	Ya	Tidak	Mulut (saluran c	>3x sehari	6	1-3x sehari	5	11	<1x sebulan	<1x sebulan	Tidak pernah	<1x sebulan	1-3x sepekan	<1x sebulan	<1x sebulan	<1x sebulan	<1x sebulan	<1x sebulan	<1x sebulan
Serat	Kurang dari 1 mi	Produksi industri	Air Tawar, Tanah	Ya	Ya	Mulut (saluran c	>3x sehari	6	1-3x sehari	5	11	Tidak pernah	Tidak pernah	1-3x sehari	1-3x sehari	>3x sehari	>3x sehari	1-3x sebulan	>3x sehari	1-3x sehari	<1x sebulan	>3x sehari
Serat	Kurang dari 1 mi	Pelepasan serat	Air Tawar	Ya	Ya	Mulut (saluran c	>3x sehari	6	1-3x sehari	5	11	Tidak pernah	Tidak pernah	>3x sehari	1-3x sebulan	1-3x sehari	1-3x sehari	1-3x sebulan	1-3x sebulan	1-3x sebulan	<1x sebulan	<1x sebulan
Partikel tidak ber	Kurang dari 1 mi	Produksi industri	Air Tawar, Produ	Tidak tahu	Ya	Mulut (saluran c	>3x sehari	6	1-3x sehari	5	11	>3x sehari	Tidak pernah	>3x sehari	Tidak pernah	<1x sebulan	<1x sebulan	1-3x sepekan	Tidak pernah	<1x sebulan	Tidak pernah	Tidak pernah
Serat	Kurang dari 1 mi	Produksi industri	Air Tawar	Ya	Ya	Mulut (saluran c	>3x sehari	6	1-3x sehari	5	11	>3x sehari	Tidak pernah	>3x sehari	<1x sebulan	Tidak pernah	Tidak pernah	Tidak pernah	Tidak pernah	Tidak pernah	Tidak pernah	Tidak pernah
Serat	Kurang dari 1 mi	Produksi industri	Air Tawar	Tidak	Ya	Mulut (saluran c	>3x sehari	6	1-3x sehari	5	11	1-3x sehari	1-3x sehari	1-3x sehari	1-3x sehari	>3x sehari	>3x sehari	>3x sehari	>3x sehari	>3x sehari	>3x sehari	>3x sehari
Serat	Kurang dari 1 mi	Produksi industri	Air Tawar, Air La	Ya	Ya	Mulut (saluran c	>3x sehari	6	1-3x sehari	5	11	>3x sehari	Tidak pernah	>3x sehari	1-3x sehari	>3x sehari	1-3x sehari	Tidak pernah	>3x sehari	1-3x sehari	<1x sebulan	>3x sehari
Partikel bulat	Sekitar 1 - 5 cm	Pelepasan serat	Air Tawar, Air La	Ya	Ya	Mulut (saluran c	>3x sehari	6	1-3x sehari	5	11	Tidak pernah	>3x sehari	<1x sebulan	Tidak pernah	>3x sehari	Tidak pernah	1-3x sehari	1-3x sepekan	>3x sehari	Tidak pernah	Tidak pernah
Serat, Partikel bu	Kurang dari 1 mi	Produksi industri	Air Tawar, Air La	Ya	Ya	Mulut (saluran c	>3x sehari	6	1-3x sehari	5	11	1-3x sepekan	Tidak pernah	1-3x sehari	1-3x sehari	>3x sehari	1-3x sehari	4-6x sepekan	4-6x sepekan	1-3x sebulan	1-3x sepekan	1-3x sepekan
Partikel Tidak Be	Kurang dari 1 mi	Pencucian pakai	Air Laut	Ya	Ya	Mulut (saluran c	1-3x sehari	5	>3x sehari	6	11	>3x sehari	1-3x sehari	1-3x sehari	4-6x sepekan	1-3x sehari	4-6x sepekan	1-3x sebulan	4-6x sepekan	>3x sehari	4-6x sepekan	4-6x sepekan
Serat	Kurang dari 1 mi	Produksi industri	Air Tawar, Air La	Ya	Tidak	Mulut (saluran c	1-3x sehari	5	>3x sehari	6	11	1-3x sebulan	Tidak pernah	>3x sehari	>3x sehari	>3x sehari	>3x sehari	>3x sehari	>3x sehari	1-3x sehari	4-6x sepekan	1-3x sebulan
Film	Kurang dari 1 mi	Penggunaan pro	Air Laut, Tanah,	Ya	Tidak	Mulut (saluran c	1-3x sehari	5	>3x sehari	6	11	>3x sehari	>3x sehari	Tidak pernah	Tidak pernah	Tidak pernah	Tidak pernah	Tidak pernah	Tidak pernah	Tidak pernah	<1x sebulan	Tidak pernah
Serat	Kurang dari 1 mi	Pencucian pakai	Produk hewani s	Tidak Tahu	Tidak	Mulut (saluran c	1-3x sehari	5	>3x sehari	6	11	1-3x sehari	1-3x sehari	>3x sehari	>3x sehari	>3x sehari	>3x sehari	Tidak pernah	1-3x sebulan	1-3x sebulan	Tidak pernah	4-6x sepekan
Serat, Partikel bu	Kurang dari 1 mi	Penggunaan pro	Air Laut	Ya	Ya	Mulut (saluran c	1-3x sehari	5	>3x sehari	6	11	>3x sehari	Tidak pernah	>3x sehari	>3x sehari	>3x sehari	>3x sehari	>3x sehari	1-3x sehari	1-3x sehari	1-3x sehari	>3x sehari
Serat	Kurang dari 1 mi	Produksi industri	Air Tawar, Air La	Ya	Ya	Mulut (saluran c	1-3x sehari	5	>3x sehari	6	11	4-6x sepekan	>3x sehari	1-3x sehari	4-6x sepekan	1-3x sepekan	1-3x sehari	1-3x sehari	4-6x sepekan	1-3x sehari	1-3x sepekan	1-3x sepekan
Partikel tidak ber	Kurang dari 1 mi	Penggunaan pro	Air Laut, Biota La	Ya	Tidak	Mulut (saluran c	1-3x sehari	5	>3x sehari	6	11	1-3x sehari	Tidak pernah	>3x sehari	1-3x sebulan	1-3x sebulan	1-3x sebulan	1-3x sebulan	1-3x sebulan	1-3x sebulan	1-3x sebulan	1-3x sebulan
Serat	Kurang dari 1 mi	Penggunaan pro	Air Tawar	Tidak tahu	Tidak	Mulut (saluran c	1-3x sehari	5	>3x sehari	6	11	>3x sehari	Tidak pernah	>3x sehari	1-3x sepekan	>3x sehari	1-3x sepekan	1-3x sepekan	>3x sehari	>3x sehari	4-6x sepekan	>3x sehari
Partikel tidak ber	Kurang dari 1 mi	Produksi industri	Udara	Ya	Ya	Mulut (saluran c	1-3x sehari	5	>3x sehari	6	11	>3x sehari	Tidak pernah	>3x sehari	Tidak pernah	1-3x sepekan	1-3x sepekan	1-3x sepekan	1-3x sehari	1-3x sepekan	1-3x sepekan	>3x sehari
Partikel tidak ber	Kurang dari 1 mi	Produksi industri	Air Laut, Udara	Ya	Tidak	Hidung (Saluran	1-3x sehari	5	>3x sehari	6	11	>3x sehari	Tidak pernah	>3x sehari	>3x sehari	>3x sehari	>3x sehari	>3x sehari	>3x sehari	>3x sehari	Tidak pernah	>3x sehari
Serat, Partikel bu	Kurang dari 1 mi	Pencucian pakai	Air Tawar, Air La	Ya	Tidak	Mulut (saluran c	1-3x sehari	5	>3x sehari	6	11	Tidak pernah	Tidak pernah	Tidak pernah	Tidak pernah	>3x sehari	1-3x sehari	<1x sebulan	>3x sehari	1-3x sehari	<1x sebulan	1-3x sebulan
Serat, Partikel tid	Kurang dari 1 mi	Produksi industri	Air Laut, Tanah,	Ya	Tidak	Mulut (saluran c	1-3x sehari	5	>3x sehari	6	11	1-3x sepekan	>3x sehari	1-3x sepekan	1-3x sepekan	1-3x sehari	1-3x sehari	1-3x sepekan	1-3x sehari	>3x sehari	1-3x sehari	>3x sehari
Partikel tidak ber	Kurang dari 1 mi	Produksi industri	Air Tawar, Air La	Ya	Ya	Mulut (saluran c	1-3x sehari	5	>3x sehari	6	11	1-3x sebulan	>3x sehari	4-6x sepekan	>3x sehari	1-3x sehari	1-3x sebulan	1-3x sepekan	>3x sehari	1-3x sepekan	4-6x sepekan	1-3x sebulan
Partikel tidak ber	Kurang dari 1 mi	Produksi industri	Air Tawar	Ya	Tidak	Mulut (saluran c	1-3x sehari	5	>3x sehari	6	11	1-3x sehari	Tidak pernah	Tidak pernah	>3x sehari	4-6x sepekan	1-3x sebulan	>3x sehari	Tidak pernah	1-3x sehari	4-6x sepekan	>3x sehari
Partikel tidak ber	Kurang dari 1 mi	Produksi industri	Air Tawar	Ya	Tidak	Mulut (saluran c	1-3x sehari	5	>3x sehari	6	11	Tidak pernah	>3x sehari	Tidak pernah	>3x sehari	4-6x sepekan	1-3x sebulan	>3x sehari	Tidak pernah	1-3x sehari	4-6x sepekan	<1x sebulan
Serat, Partikel tid	Sekitar 1 - 5 cm	Produksi industri	Air Tawar, Air La	Ya	Ya	Mulut (saluran c	1-3x sehari	5	>3x sehari	6	11	4-6x sepekan	1-3x sehari	4-6x sepekan	>3x sehari	>3x sehari	4-6x sepekan	4-6x sepekan	<1x sebulan	<1x sebulan	1-3x sepekan	1-3x sepekan
Partikel Tidak Be	Kurang dari 1 mi	Pelepasan serat	Air Laut, Tanah,	Ya	Tidak	Mulut (saluran c	>3x sehari	6	>3x sehari	6	12	1-3x sebulan	<1x sebulan	1-3x sehari	>3x sehari	1-3x sepekan	4-6x sepekan	Tidak pernah	1-3x sehari	Tidak pernah	1-3x sebulan	1-3x sebulan
Partikel Tidak Be	Sekitar 1 - 5 cm	Produksi industri	Air Laut	Ya	Tidak	Mulut (saluran c	>3x sehari	6	>3x sehari	6	12	<1x sebulan	<1x sebulan	4-6x sepekan	1-3x sehari	>3x sehari	>3x sehari	>3x sehari	>3x sehari	>3x sehari	>3x sehari	>3x sehari
Serat	Sekitar 1 - 5 cm	Penggunaan pro	Produk hewani s	Ya	Ya	Mulut (saluran c	>3x sehari	6	>3x sehari	6	12	>3x sehari	>3x sehari	>3x sehari	>3x sehari	>3x sehari	>3x sehari	>3x sehari	>3x sehari	>3x sehari	>3x sehari	>3x sehari
Serat, Partikel B	Sekitar 1 - 5 cm	Produksi industri	Tanah	Ya	Ya	Mulut (saluran c	>3x sehari	6	>3x sehari	6	12	<1x sebulan	<1x sebulan	<1x sebulan	<1x sebulan	<1x sebulan	<1x sebulan	<1x sebulan	<1x sebulan	<1x sebulan	<1x sebulan	<1x sebulan
Partikel Tidak Be	Kurang dari 1 mi	Produksi industri	Produk hewani s	Ya	Ya	Mulut (saluran c	>3x sehari	6	>3x sehari	6	12	1-3x sepekan	>3x sehari	1-3x sebulan	1-3x sepekan	1-3x sebulan	1-3x sebulan	1-3x sebulan	1-3x sebulan	1-3x sebulan	1-3x sebulan	1-3x sebulan
Serat	Kurang dari 1 mi	Produksi industri	Biota Laut	Ya	Tidak	Mulut (saluran c	>3x sehari	6	>3x sehari	6	12	1-3x sehari	Tidak pernah	>3x sehari	1-3x sepekan	4-6x sepekan	1-3x sebulan	Tidak pernah	1-3x sepekan	1-3x sehari	Tidak pernah	1-3x sebulan
Serat	Kurang dari 1 mi	Produksi industri	Udara, Tanah	Ya	Ya	Mulut (saluran c	>3x sehari	6	>3x sehari	6	12	>3x sehari	>3x sehari	>3x sehari	>3x sehari	>3x sehari	>3x sehari	>3x sehari	>3x sehari	>3x sehari	>3x sehari	>3x sehari
Partikel tidak ber	Kurang dari 1 mi	Penggunaan pro	Air Tawar	Ya	Ya	Mulut (saluran c	>3x sehari	6	>3x sehari	6	12	4-6x sepekan	4-6x sepekan	Tidak pernah	Tidak pernah	>3x sehari	4-6x sepekan	1-3x sebulan	1-3x sehari	>3x sehari	>3x sehari	4-6x sepekan
Serat, Partikel bu	Kurang dari 1 mi	Produksi industri	Air Tawar, Air La	Ya	Ya	Mulut (saluran c	>3x sehari	6	>3x sehari	6	12	>3x sehari	Tidak pernah	Tidak pernah	>3x sehari	>3x sehari	<1x sebulan	Tidak pernah	>3x sehari	>3x sehari	>3x sehari	>3x sehari
Serat	Kurang dari 1 mi	Penggunaan pro	Air Tawar, Air La	Ya	Tidak	Mulut (saluran c	>3x sehari	6	>3x sehari	6	12	4-6x sepekan	>3x sehari	1-3x sebulan	1-3x sepekan	>3x sehari	>3x sehari	4-6x sepekan	>3x sehari	>3x sehari	1-3x sebulan	1-3x sebulan
Serat, Partikel bu	Lebih dari 10 cm	Pelepasan serat	Garam	Ya	Ya	Mulut (saluran c	>3x sehari	6	>3x sehari	6	12	>3x sehari	>3x sehari	>3x sehari	>3x sehari	>3x sehari	>3x sehari	>3x sehari	>3x sehari	>3x sehari	>3x sehari	>3x sehari
Partikel tidak ber	Kurang dari 1 mi	Penggunaan pro	Produk hewani s	Ya	Tidak	Mulut (saluran c	>3x sehari	6	>3x sehari	6	12	>3x sehari	Tidak pernah	1-3x sepekan	4-6x sepekan	4-6x sepekan	1-3x sehari	<1x sebulan	>3x sehari	1-3x sepekan	<1x sebulan	>3x sehari
Partikel tidak ber	Kurang dari 1 mi	Produksi industri	Air Tawar, Udara	Tidak tahu	Tidak	Mulut (saluran c	>3x sehari	6	>3x sehari	6	12	>3x sehari	Tidak pernah	4-6x sepekan	4-6x sepekan	1-3x sehari	1-3x sehari	Tidak pernah	1-3x sehari	1-3x sehari	1-3x sehari	1-3x sehari
Partikel bulat	Sekitar 1 - 5 cm	Penggunaan pro	Produk hewani s	Ya	Ya	Mulut (saluran c	>3x sehari	6	>3x sehari	6	12	>3x sehari	>3x sehari	Tidak pernah	Tidak pernah	>3x sehari	>3x sehari	>3x sehari	>3x sehari	>3x sehari	>3x sehari	>3x sehari
Serat	Kurang dari 1 mi	Produksi industri	Air Tawar	Ya	Tidak	Mulut (saluran c	>3x sehari	6	>3x sehari	6	12	Tidak pernah	>3x sehari	>3x sehari	Tidak pernah	>3x sehari	Tidak pernah	Tidak pernah	Tidak pernah	>3x sehari	Tidak pernah	Tidak pernah
Serat, Partikel bu	Kurang dari 1 mi	Produksi industri	Air Tawar, Air La	Ya	Ya	Mulut (saluran c	>3x sehari	6	>3x sehari	6	12	4-6x sepekan	1-3x sebulan	>3x sehari	>3x sehari	>3x sehari	4-6x sepekan	1-3x sehari	4-6x sepekan	4-6x sepekan	1-3x sepekan	1-3x sepekan
Partikel tidak ber	Kurang dari 1 mi	Produksi industri	Air Tawar, Air La	Ya	Ya	Mulut (saluran c	>3x sehari	6	>3x sehari	6	12	>3x sehari	>3x sehari	1-3x sebulan	1-3x sebulan	4-6x sepekan	1-3x sepekan	4-6x sepekan	>3x sehari	4-6x sepekan	1-3x sepekan	>3x sehari
Partikel tidak ber	Kurang dari 1 mi	Pelepasan serat	Air Tawar, Tanah	Ya	Tidak	Mulut (saluran c	>3x sehari	6	>3x sehari	6	12	>3x sehari	>3x sehari	<1x sebulan	1-3x sebulan	1-3x sepekan	1-3x sebulan	1-3x sebulan	1-3x sebulan	1-3x sebulan	1-3x sebulan	<1x sebulan
Partikel bulat	Kurang dari 1 mi	Produksi industri	Air Tawar, Tanah	Ya	Tidak	Mulut (saluran c	>3x sehari	6	>3x sehari	6	12	4-6x sepekan	Tidak pernah	1-3x sehari	1-3x sebulan	1-3x sepekan	1-3x sehari	1-3x sebulan	1-3x sehari	1-3x sehari	1-3x sehari	1-3x sepekan
Film	Sekitar 1 - 5 cm	Penggunaan pro	Air Laut, Udara,	Ya	Tidak	Mulut (saluran c	>3x sehari	6	>3x sehari	6	12	<1x sebulan	>3x sehari	1-3x sebulan	1-3x sebulan	<1x sebulan	1-3x sebulan	1-3x sebulan	1-3x sehari	1-3x sepekan	1-3x sebulan	1-3x sebulan
Partikel tidak ber	Kurang dari 1 mi	Penggunaan pro	Produk hewani s	Ya	Tidak	Mulut (saluran c	>3x sehari	6	>3x sehari	6	12	>3x sehari	>3x sehari	1-3x sepekan	1-3x sehari	1-3x sepekan	4-6x sepekan	1-3x sepekan	4-6x sepekan	Tidak pernah	Tidak pernah	Tidak pernah
Serat	Kurang dari 1 mi	Produksi industri	Air Laut	Ya	Ya	Hidung (Saluran	>3x sehari	6	>3x sehari	6	12	>3x sehari	>3x sehari	1-3x sehari	>3x sehari	>3x sehari	1-3x sehari	1-3x sehari	1-3x sehari	1-3x sehari	1-3x sehari	1-3x sehari
Partikel bulat, Pa	Sekitar 1 - 5 cm	Produksi industri	Air Tawar, Air La	Ya	Ya	Mulut (saluran c	>3x sehari	6	>3x sehari	6	12	1-3x sebulan	Tidak pernah	Tidak pernah	<1x sebulan	1-3x sebulan	4-6x sepekan	1-3x sebulan	1-3x sepekan	4-6x sepekan	1-3x sepekan	1-3x sebulan
Serat	Kurang dari 1 mi	Penggunaan pro	Tanah	Ya	Tidak	Mulut (saluran c	>3x sehari	6	>3x sehari	6	12	>3x sehari	Tidak pernah	Tidak pernah	Tidak pernah	Tidak pernah	Tidak pernah	Tidak pernah	Tidak pernah	Tidak pernah	Tidak pernah	Tidak pernah
Serat	Kurang dari 1 mi	Produksi industri	Air Laut, Tanah	Ya	Tidak	Mulut (saluran c	>3x sehari	6	>3x sehari	6	12	>3x sehari	>3x sehari	>3x sehari	>3x sehari	>3x sehari	>3x sehari	>3x sehari	>3x sehari	>3x sehari	>3x sehari	>3x sehari
Serat	Kurang dari 1 mi	Produksi industri	Air Tawar, Udara	Ya	Ya	Mulut (saluran c	>3x sehari	6	>3x sehari	6	12	>3x sehari	4-6x sepekan	4-6x sepekan	4-6x sepekan	4-6x sepekan	1-3x sehari	4-6x sepekan	4-6x sepekan	1-3x sehari	4-6x sepekan	1-3x sebulan
Partikel tidak ber	Kurang dari 1 mi	Penggunaan pro	Air Laut	Tidak tahu	Tidak	Mulut (saluran c	>3x sehari	6	>3x sehari	6	12	4-6x sepekan	1-3x sehari	4-6x sepekan	Tidak pernah	4-6x sepekan	>3x sehari	1-3x sehari	1-3x sehari	>3x sehari	Tidak pernah	Tidak pernah
Serat, Partikel tid	Kurang dari 1 mi	Penggunaan pro	Air Tawar, Air La	Ya	Ya	Mulut (saluran c	>3x sehari	6	>3x sehari	6	12	>3x sehari	Tidak pernah	>3x sehari	>3x sehari	>3x sehari	>3x sehari	>3x sehari	>3x sehari	>3x sehari	>3x sehari	>3x sehari
Serat	Sekitar 1 - 5 cm	Produksi industri	Air Tawar, Air La	Ya	Ya	Mulut (saluran c	>3x sehari	6	>3x sehari	6	12	>3x sehari	Tidak pernah	1-3x sebulan	4-6x sepekan	1-3x sepekan	1-3x sehari	4-6x sepekan	1-3x sepekan	1-3x sepekan	1-3x sehari	4-6x sepekan
Partikel bulat	Kurang dari 1 mi	Produksi industri	Udara	Ya	Ya	Mulut (saluran c	>3x sehari	6	>3x sehari	6	12	Tidak pernah	>3x sehari	1-3x sehari	>3x sehari	>3x sehari	4-6x sepekan	<1x sebulan	>3x sehari	1-3x sebulan	Tidak pernah	<1x sebulan
Partikel tidak ber	Kurang dari 1 mi	Produksi industri	Air Laut	Ya	Ya	Mulut (saluran c	>3x sehari	6	>3x sehari	6	12	>3x sehari	<1x sebulan	<1x sebulan	<1x sebulan	<1x sebulan	<1x sebulan	<1x sebulan	<1x sebulan	<1x sebulan	<1x sebulan	<1x sebulan

Seberapa serin	Skor pengguna	Harga	Merek	Kualitas	Ketersediaan/k	Prefensi pribad	Kemasan	Dari mana Anda	Bagaimana And	Mikroplastik da	Mikroplastik tid	Mikroplastik me	Mikroplastik me	Mikroplastik me	Apakah Anda k	Apakah Anda k	Seberapa serin	Bagaimana sika	skor sikap	Apakah Anda m	Apakah Anda s	Menurut Anda,
Tidak pernah	20	4	3	2	4	3	1	Buku/Jurnal, Kor	Digunakan untuk	5	5	5	5	5	Ya	Ya	Terkadang (>50%	Mengurangi Pen	25	Ya	Ya	Ya
Tidak pernah	26	5	4	4	5	5	5	Televisi, Buku/Ju	Digunakan untuk	5	5	5	5	5	Ya	Ya	Selalu	Mengurangi Pen	25	Tidak	Ya	Ya
Tidak pernah	16	4	3	4	4	5	4	Televisi	Digunakan untuk	5	5	5	4	3	Ya	Ya	Jarang (<50%)	Mengurangi pen	22	Tidak	Ya	Ya
Tidak pernah	8	5	1	5	5	1	1	Televisi, Internet	Digunakan untuk	5	5	5	5	5	Ya	Ya	Jarang (<50%)	Menghentikan k	25	Ya	Ya	Ya
Tidak pernah	12	2	2	3	3	2	1	Televisi, Buku/Ju	Digunakan untuk	5	5	5	4	4	Ya	Ya	Selalu	Mengurangi pen	23	Tidak	Ya	Ya
Tidak pernah	12	5	5	5	5	5	5	Internet, Lebel p	Digunakan untuk	5	5	5	5	5	Ya	Ya	Selalu	Menghentikan k	25	Ya	Ya	Tidak
Tidak pernah	14	4	4	4	5	5	4	Televisi, Lebel p	Diisi ulang	4	5	4	4	4	Ya	Ya	Terkadang (>50%	Mengurangi pen	21	Ya	Ya	Ya
Tidak pernah	13	5	5	5	5	5	5	Buku/Jurnal, Kor	Digunakan untuk	5	5	5	5	5	Ya	Ya	Terkadang (>50%	Mengurangi pen	25	Ya	Ya	Ya
<1x sebulan	21	3	3	4	4	3	3	Internet, Teman/	Dibuang setelah	5	5	5	5	5	Ya	Ya	Terkadang (>50%	Mengurangi Pen	25	Tidak	Ya	Ya
Tidak pernah	16	2	5	5	4	4	5	Buku/Jurnal, Inte	Diisi ulang	5	5	5	5	5	Ya	Ya	Selalu	Mengurangi pen	25	Ya	Ya	Ya
Tidak pernah	8	4	4	5	5	5	4	Buku/Jurnal	Dibuang setelah	5	5	5	5	5	Ya	Ya	Terkadang (>50%	Menghentikan k	25	Ya	Ya	Ya
<1x sebulan	16	4	3	4	4	4	4	Internet	Dibuang setelah	4	4	3	3	4	Tidak tahu	Tidak tahu	Tidak pernah	Tetap mengguna	18	Tidak	Ya	Ya
<1x sebulan	18	5	2	4	4	4	2	Televisi, Internet	Dibuang setelah	4	3	4	4	4	Tidak tahu	Ya	Jarang (<50%)	Menghentikan k	19	Tidak	Ya	Ya
1-3x sepekan	25	4	4	5	5	5	5	Televisi, Internet	Digunakan untuk	5	5	5	5	5	Ya	Ya	Selalu	Mengurangi Pen	25	Ya	Ya	Ya
Tidak pernah	31	4	4	4	4	4	4	Televisi, Internet	Digunakan untuk	4	4	4	4	4	Ya	Ya	Jarang (<50%)	Mengurangi pen	20	Tidak	Ya	Tidak
Tidak pernah	36	4	4	5	4	4	4	Televisi, Internet	Dibuang setelah	5	5	5	5	5	Ya	Ya	Selalu	Mengurangi pen	25	Tidak	Ya	Ya
4-6x sepekan	33	5	5	5	5	5	3	Televisi, Internet	Diisi Ulang	5	5	5	5	5	Ya	Ya	Selalu	Mengurangi Pen	25	Ya	Ya	Ya
1-3x sebulan	17	5	3	3	5	3	3	Internet	Digunakan untuk	4	5	4	5	5	Ya	Tidak	Terkadang (>50%	Mengurangi Pen	23	Ya	Ya	Ya
<1x sebulan	18	4	5	5	5	5	3	Televisi, Internet	Digunakan untuk	5	5	5	5	3	Ya	Ya	Terkadang (>50%	Mengurangi pen	23	Ya	Ya	Ya
1-3x sebulan	20	3	3	4	4	4	4	Internet, Lebel p	Dibuang setelah	5	5	5	5	3	Ya	Ya	Jarang (<50%)	Mengurangi pen	23	Ya	Ya	Ya
1-3x sepekan	24	4	4	4	4	4	4	Internet, Lainnya	Digunakan untuk	4	4	4	4	4	Ya	Ya	Jarang (<50%)	Mengurangi pen	20	Ya	Ya	Ya
1-3x sebulan	13	1	2	2	3	1	3	Televisi, Internet	Dibuang setelah	5	5	5	5	3	Ya	Ya	Jarang (<50%)	Mengurangi pen	23	Ya	Ya	Ya
Tidak pernah	25	3	3	4	4	3	3	Televisi, Internet	Digunakan untuk	3	5	3	3	3	Ya	Ya	Terkadang (>50%	Mengurangi pen	17	Tidak	Ya	Ya
Tidak pernah	17	1	5	5	5	5	1	Internet, Lebel p	Dibuang setelah	5	5	5	5	5	Ya	Ya	Selalu	Mengurangi pen	25	Ya	Ya	Ya
Tidak pernah	19	5	5	5	5	4	5	Buku/Jurnal, Inte	Digunakan untuk	5	3	4	4	3	Ya	Ya	Selalu	Mengurangi pen	19	Ya	Ya	Tidak
1-3x sepekan	23	4	4	4	4	4	3	Televisi	Dibuang setelah	5	5	4	4	5	Tidak	Ya	Jarang (<50%)	Menghentikan k	23	Tidak	Ya	Tidak
Tidak pernah	22	5	5	5	5	4	5	Televisi, Internet	Dibuang setelah	5	5	5	5	5	Ya	Ya	Terkadang (>50%	Mengurangi pen	25	Ya	Ya	Ya
4-6x sepekan	33	4	4	4	4	5	3	Televisi, Internet	Dibuang setelah	4	4	4	4	2	Ya	Ya	Tidak pernah	Menghentikan k	18	Tidak	Ya	Ya
Tidak pernah	26	4	4	4	4	4	4	Televisi, Internet	Diisi ulang	4	5	4	5	4	Ya	Ya	Terkadang (>50%	Mengurangi pen	22	Tidak	Ya	Ya
<1x sebulan	9	3	5	5	5	5	4	Lainnya	Dibuang setelah	5	5	5	5	5	Ya	Tidak tahu	Tidak pernah	Mengurangi Pen	25	Tidak	Ya	Ya
1-3x sepekan	31	3	4	3	4	4	4	Internet, Lebel p	Dibuang setelah	4	4	5	4	4	Ya	Ya	Terkadang (>50%	Mengurangi pen	21	Tidak	Ya	Ya
Tidak pernah	23	5	3	4	5	4	3	Internet, Lebel p	Diisi ulang	4	4	4	4	3	Ya	Ya	Jarang (<50%)	Mengurangi pen	19	Tidak	Ya	Ya
1-3x sebulan	23	4	5	5	3	4	4	Televisi, Internet	Dibuang setelah	5	3	4	4	3	Ya	Ya	Jarang (<50%)	Mengurangi Pen	19	Ya	Ya	Ya
1-3x sebulan	21	3	3	4	4	3	3	Televisi, Internet	Dibuang setelah	4	4	4	4	3	Ya	Ya	Jarang (<50%)	Mengurangi Pen	19	Ya	Ya	Ya
<1x sebulan	16	4	5	5	5	4	4	Internet, Lebel p	Dibuang setelah	5	5	5	5	5	Ya	Ya	Tidak pernah	Mengurangi Pen	25	Tidak	Ya	Ya
<1x sebulan	25	3	4	5	5	4	4	Televisi, Internet	Digunakan untuk	5	5	5	5	4	Ya	Ya	Selalu	Mengurangi pen	24	Ya	Ya	Ya
<1x sebulan	24	5	5	5	5	5	5	Internet, Teman/	Digunakan untuk	5	5	5	5	5	Ya	Ya	Jarang (<50%)	Menghentikan k	25	Ya	Ya	Ya
1-3x sebulan	12	5	2	3	5	3	2	Televisi, Buku/Ju	Diisi ulang	4	4	4	4	4	Ya	Ya	Terkadang (>50%	Menghentikan k	20	Tidak	Ya	Ya
1-3x sebulan	23	2	4	5	3	3	2	Lebel pada botol	Dibuang setelah	4	5	4	3	3	Ya	Ya	Jarang (<50%)	Mengurangi pen	19	Ya	Ya	Ya
<1x sebulan	23	3	3	5	4	4	5	Televisi, Lainnya	Dibuang setelah	4	4	4	4	4	Ya	Ya	Terkadang (>50%	Mengurangi pen	20	Ya	Ya	Ya
1-3x sebulan	31	3	5	5	5	5	5	Buku/Jurnal, Inte	Digunakan untuk	5	5	5	5	3	Ya	Ya	Terkadang (>50%	Mengurangi pen	23	Tidak	Ya	Ya
<1x sebulan	18	3	5	5	5	5	5	Televisi, Buku/Ju	Digunakan untuk	5	5	5	5	5	Ya	Ya	Selalu	Menghentikan k	25	Ya	Ya	Ya
4-6x sepekan	38	4	4	4	4	3	4	Internet, Lebel p	Digunakan untuk	5	5	5	5	5	Ya	Ya	Terkadang (>50%	Menghentikan k	25	Ya	Ya	Ya
<1x sebulan	16	3	5	5	5	5	5	Televisi, Buku/Ju	Dibuang setelah	5	5	5	5	5	Ya	Ya	Terkadang (>50%	Mengurangi pen	25	Tidak	Ya	Ya
1-3x sebulan	31	3	3	3	3	5	5	Televisi, Internet	Diisi ulang	5	5	5	5	5	Ya	Ya	Selalu	Menghentikan k	25	Tidak	Ya	Ya
Tidak pernah	18	4	4	4	4	4	4	Lainnya	Dibuang setelah	5	4	5	5	4	Ya	Ya	Tidak pernah	Mengurangi pen	23	Tidak	Ya	Ya
4-6x sepekan	12	5	5	5	3	4	5	Televisi, Internet	Dibuang setelah	5	5	5	5	1	Ya	Ya	Tidak pernah	Mengurangi pen	21	Tidak	Tidak	Ya
Tidak pernah	24	4	4	4	4	3	3	Televisi, Internet	Dibuang setelah	5	5	5	5	5	Ya	Ya	Jarang (<50%)	Menghentikan k	25	Ya	Ya	Ya
>3x sehari	56	1	1	5	5	1	1	Buku/Jurnal, Inte	Digunakan untuk	5	5	5	5	5	Ya	Ya	Selalu	Mengurangi Pen	25	Ya	Ya	Ya
1-3x sehari	29	3	3	3	1	3	3	Televisi, Internet	Diisi ulang	1	5	5	5	3	Ya	Ya	Terkadang (>50%	Mengurangi pen	19	Ya	Ya	Tidak
1-3x sehari	13	5	5	5	5	5	5	Internet	Dibuang setelah	5	5	5	5	5	Ya	Ya	Selalu	Mengurangi pen	25	Ya	Ya	Ya
Tidak pernah	24	5	3	2	5	4	2	Internet, Lebel p	Diisi ulang	5	5	5	5	3	Ya	Ya	Terkadang (>50%	Mengurangi pen	23	Tidak	Ya	Ya
<1x sebulan	34	4	4	5	5	4	4	Televisi, Lebel p	Digunakan untuk	4	2	2	2	4	Ya	Ya	Jarang (<50%)	Menghentikan k	14	Ya	Ya	Ya
Tidak pernah	25	5	5	5	5	5	5	Buku/Jurnal, Inte	Digunakan untuk	5	5	5	4	4	Ya	Ya	Terkadang (>50%	Mengurangi Pen	23	Ya	Ya	Ya
1-3x sebulan	34	5	3	5	5	2	5	Internet	Diisi Ulang	5	5	5	5	5	Tidak	Ya	Jarang (<50%)	Mengurangi Pen	25	Tidak	Ya	Ya
Tidak pernah	29	5	5	5	5	4	4	Televisi, Internet	Digunakan untuk	5	4	5	4	3	Ya	Ya	Terkadang (>50%	Menghentikan k	21	Tidak	Ya	Ya
1-3x sepekan	28	5	5	5	5	5	5	Internet, Teman/	Dibuang setelah	5	4	4	3	4	Ya	Ya	Jarang (<50%)	Menghentikan k	20	Tidak	Ya	Ya
Tidak pernah	21	4	4	5	5	4	5	Televisi	Digunakan untuk	5	2	3	5	5	Tidak	Ya	Selalu	Menghentikan k	20	Tidak	Ya	Ya
1-3x sebulan	25	3	5	4	4	5	4	Teman/Kerabat	Dibuang setelah	3	4	4	4	4	Ya	Ya	Tidak pernah	Mengurangi pen	19	Tidak	Ya	Ya
4-6x sepekan	29	4	4	5	5	1	3	Buku/Jurnal, Inte	Dibuang setelah	5	5	5	5	5	Ya	Ya	Tidak pernah	Mengurangi pen	25	Tidak	Ya	Ya
>3x sehari	29	3	3	5	4	3	3	Televisi, Buku/Ju	Digunakan untuk	5	5	5	5	4	Ya	Ya	Jarang (<50%)	Mengurangi pen	24	Ya	Ya	Ya
<1x sebulan	26	4	4	5	4	3	3	Televisi, Internet	Dibuang setelah	4	4	4	5	4	Ya	Tidak tahu	Terkadang (>50%	Mengurangi Pen	21	Tidak	Ya	Ya
1-3x sepekan	11	1	3	3	4	3	4	Televisi, Internet	Dibuang setelah	5	4	5	5	5	Ya	Ya	Jarang (<50%)	Menghentikan k	24	Ya	Ya	Ya
1-3x sebulan	27	5	4	5	5	5	5	Televisi	Dibuang setelah	5	5	5	5	5	Ya	Ya	Terkadang (>50%	Mengurangi Pen	25	Ya	Ya	Ya
Tidak pernah	8	4	4	4	4	3	4	Televisi, Lebel p	Digunakan untuk	4	4	4	3	2	Ya	Ya	Terkadang (>50%	Menghentikan k	17	Ya	Ya	Ya
1-3x sepekan	20	4	4	5	5	5	5	Buku/Jurnal, Inte	Digunakan untuk	5	5	5	5	5	Ya	Ya	Terkadang (>50%	Mengurangi Pen	25	Ya	Ya	Ya
1-3x sebulan	24	5	4	5	5	5	5	Internet, Lebel p	Dibuang setelah	4	4	4	4	3	Ya	Ya	Terkadang (>50%	Menghentikan k	19	Ya	Ya	Ya
1-3x sepekan	26	5	4	4	4	3	3	Televisi, Internet	Diisi ulang	4	4	5	5	4	Ya	Ya	Jarang (<50%)	Mengurangi pen	22	Tidak	Ya	Ya
1-3x sebulan	27	4	5	5	4	3	3	Buku/Jurnal, Leb	Dibuang setelah	5	4	5	5	3	Ya	Ya	Terkadang (>50%	Mengurangi pen	22	Tidak	Ya	Ya
Tidak pernah	33	4	4	2	4	4	2	Televisi, Internet	Digunakan untuk	4	5	4	4	2	Ya	Ya	Terkadang (>50%	Mengurangi pen	19	Tidak	Ya	Ya
1-3x sepekan	25	3	4	4	4	4	4	Buku/Jurnal, Inte	Dibuang setelah	5	5	5	5	4	Ya	Ya	Selalu	Mengurangi pen	24	Ya	Ya	Ya
<1x sebulan	22	5	1	2	4	4	5	Buku/Jurnal, Inte	Diisi Ulang	5	5	5	5	5	Ya	Ya	Jarang (<50%)	Mengurangi Pen	25	Ya	Ya	Ya
Tidak pernah	20	4	5	5	5	3	3	Buku/Jurnal, Inte	Dibuang setelah	5	5	5	5	5	Ya	Ya	Tidak pernah	Mengurangi pen	25	Tidak	Ya	Ya
Tidak pernah	23	3	5	5	5	5	5	Teman/Kerabat	Digunakan untuk	4	5	5	5	5	Ya	Ya	Selalu	Mengurangi pen	24	Tidak	Ya	Ya
1-3x sebulan	25	4	4	5	5	4	4	Internet	Diisi ulang	5	4	5	5	5	Ya	Ya	Terkadang (>50%	Mengurangi pen	24	Tidak	Ya	Ya
<1x sebulan	19	5	5	5	5	5	5	Buku/Jurnal, Inte	Dibuang setelah	5	5	5	5	5	Ya	Ya	Jarang (<50%)	Mengurangi pen	25	Tidak	Ya	Ya
Tidak pernah	13	4	4	4	4	4	4	Internet, Lainnya	Dibuang setelah	4	4	4	4	4	Ya	Ya	Tidak pernah	Menghentikan k	20	Tidak	Ya	Ya
<1x sebulan	19	3	3	3	5	4	4	Televisi, Internet	Diisi Ulang	3	4	5	5	3	Ya	Ya	Jarang (<50%)	Mengurangi Pen	20	Tidak	Ya	Ya
1-3x sebulan	20	3	4	4	4	4	2	Koran, Lebel pad	Digunakan untuk	3	3	2	4	3	Tidak	Ya	Jarang (<50%)	Mengurangi pen	15	Tidak	Tidak	Tidak
1-3x sehari	17	5	5	5	5	5	4	Buku/Jurnal, Inte	Dibuang setelah	5	5	5	5	5	Ya	Ya	Jarang (<50%)	Mengurangi pen	25	Tidak	Ya	Ya
1-3x sepekan	32	1	5	5	5	5	5	Internet, Teman/	Digunakan untuk	5	5	5	5	5	Ya	Ya	Selalu	Mengurangi Pen	25	Ya	Ya	Ya
Tidak pernah	25	3	5	5	5	5	3	Televisi, Lebel p	Dibuang setelah	5	4	3	2	3	Tidak	Tidak	Jarang (<50%)	Menghentikan k	17	Tidak	Ya	Tidak
Tidak pernah	31	1	5	3	2	4	1	Internet, Lebel p	Dibuang setelah	5	5	5	5	4	Ya	Ya	Terkadang (>50%	Menghentikan k	24	Tidak	Ya	Ya
>3x sehari	32	3	4	4	5	3	3	Internet, Lebel p	Dibuang setelah	5	4	5	5	3	Ya	Ya	Terkadang (>50%	Mengurangi Pen	22	Ya	Ya	Ya
1-3x sebulan	23	2	3	3	3	3	2	Buku/Jurnal, Inte	Diisi ulang	5	5	5	4	3	Ya	Ya	Jarang (<50%)	Mengurangi pen	22	Tidak	Ya	Ya
Tidak pernah	8	5	3	5	5	5	5	Televisi, Internet	Digunakan untuk	5	5	5	5	5	Ya	Ya	Terkadang (>50%	Mengurangi pen	25	Ya	Ya	Ya
4-6x sepekan	34	5	3	5	5	3	3	Televisi, Internet	Diisi ulang	5	5	5	5	5	Ya	Ya	Jarang (<50%)	Mengurangi pen	25	Ya	Ya	Ya
1-3x sehari	34	3	5	5	5	5	5	Televisi, Internet	Dibuang setelah	5	5	5	5	5	Ya	Ya	Selalu	Mengurangi pen	25	Tidak	Ya	Ya
1-3x sepekan	29	4	5	5	5	4	5	Televisi, Internet	Dibuang setelah	5	4	5	5	5	Ya	Ya	Selalu	Menghentikan k	24	Tidak	Ya	Ya
>3x sehari	17	2	4	4	4	4	2	Internet	Dibuang setelah	4	2	4	4	2	Ya	Ya	Terkadang (>50%	Tetap mengguna	16	Tidak	Ya	Ya
1-3x sepekan	32	4	4	4	4	4	4	Internet, Lebel p	Dibuang setelah	5	5	5	5	5	Ya	Ya	Jarang (<50%)	Mengurangi pen	25	Tidak	Ya	Ya
1-3x sepekan	30	4	5	5	4	3	4	Buku/Jurnal, Inte	Dibuang setelah	5	4	4	5	3	Ya	Ya	Jarang (<50%)	Mengurangi pen	21	Ya	Ya	Ya
1-3x sepekan	27	4	3	4	3	3	4	Lebel pada botol	Diisi ulang	4	3	4	4	4	Ya	Ya	Terkadang (>50%	Mengurangi pen	19	Tidak	Ya	Ya
1-3x sehari	18	4	4	4	4	5	3	Televisi, Internet	Dibuang setelah	4	4	4	4	3	Ya	Tidak	Jarang (<50%)	Mengurangi pen	19	Tidak	Ya	Ya
Tidak pernah	29	4	5	5	5	5	5	Internet, Lebel p	Dibuang setelah	5	5	5	5	5	Tidak tahu	Tidak tahu	Jarang (<50%)	Mengurangi pen	25	Tidak	Ya	Ya
<1x sebulan	24	4	4	4	5	4	4	Televisi, Internet	Digunakan untuk	5	5	5	5	5	Ya	Ya	Selalu	Mengurangi pen	25	Tidak	Ya	Ya
1-3x sebulan	30	4	4	4	4	4	4	Internet	Dibuang setelah	5	5	5	5	3	Ya	Tidak	Jarang (<50%)	Tetap mengguna	23	Tidak	Ya	Ya
4-6x sepekan	34	2	5	5	5	4	5	Buku/Jurnal, Inte	Digunakan untuk	5	5	5	5	2	Ya	Ya	Jarang (<50%)	Mengurangi Pen	22	Tidak	Ya	Ya
Tidak pernah	19	2	2	1	1	2	2	Lebel pada botol	Dibuang setelah	1	1	1	2	2	Ya	Ya	Terkadang (>50%	Mengurangi pen	7	Tidak	Ya	Ya
4-6x sepekan	32	4	4	4	4	5	5	Lebel pada botol	Digunakan untuk	5	5	4	5	4	Ya	Ya	Selalu	Menghentikan k	23	Tidak	Ya	Ya
<1x sebulan	28	2	4	5	4	5	3	Lebel pada botol	Dibuang setelah	5	5	5	5	3	Ya	Ya	Jarang (<50%)	Mengurangi pen	23	Tidak	Ya	Tidak
Tidak pernah	8	4	4	4	5	5	5	Internet	Dibuang setelah	5	4	5	5	5	Ya	Tidak tahu	Jarang (<50%)	Menghentikan k	24	Tidak	Ya	Ya
Tidak pernah	19	5	5	5	4	5	5	Televisi, Lebel p	Dibuang setelah	4	5	5	5	5	Ya	Ya	Jarang (<50%)	Menghentikan k	24	Tidak	Ya	Ya
4-6x sepekan	37	3	4	5	3	5	3	Televisi, Internet	Dibuang setelah	3	1	5	5	5	Ya	Ya	Jarang (<50%)	Mengurangi Pen	19	Tidak	Ya	Ya
1-3x sebulan	30	3	3	3	4	2	1	Lebel pada botol	Diisi ulang	3	5	4	5	5	Ya	Ya	Selalu	Mengurangi pen	22	Ya	Ya	Ya
Tidak pernah	19	5	5	5	5	5	5	Internet, Lebel p	Diisi ulang	5	5	5	5	5	Ya	Ya	Terkadang (>50%	Mengurangi pen	25	Ya	Ya	Ya
Tidak pernah	24	5	3	5	5	5	3	Televisi, Internet	Dibuang setelah	5	5	5	5	5	Ya	Ya	Jarang (<50%)	Mengurangi pen	25	Tidak	Ya	Ya
Tidak pernah	18	5	5	5	5	5	5	Televisi, Internet	Diisi ulang	5	5	5	5	5	Ya	Ya	Terkadang (>50%	Mengurangi pen	25	Ya	Ya	Ya
1-3x sepekan	35	5	5	4	5	2	1	Televisi, Internet	Dibuang setelah	5	5	5	5	5	Ya	Ya	Jarang (<50%)	Mengurangi Pen	25	Ya	Ya	Ya
4-6x sepekan	35	5	4	3	3	4	3	Televisi, Internet	Dibuang setelah	4	4	5	3	3	Ya	Ya	Jarang (<50%)	Menghentikan k	19	Ya	Ya	Ya
Tidak pernah	14	3	5	5	5	5	5	Internet	Digunakan untuk	5	5	5	5	1	Ya	Ya	Tidak pernah	Menghentikan k	21	Ya	Ya	Ya
1-3x sepekan	29	5	2	3	5	4	1	Televisi, Internet	Diisi Ulang	4	4	3	4	5	Tidak tahu	Tidak tahu	Jarang (<50%)	Mengurangi Pen	20	Tidak	Ya	Ya
1-3x sebulan	28	3	3	5	3	3	5	Televisi, Internet	Dibuang setelah	5	5	5	4	4	Ya	Ya	Terkadang (>50%	Mengurangi Pen	23	Tidak	Ya	Ya
1-3x sehari	43	4	5	5	4	4	4	Televisi	Dibuang setelah	4	5	5	4	4	Tidak	Tidak	Terkadang (>50%	Mengurangi pen	22	Ya	Ya	Ya
<1x sebulan	19	5	3	4	5	5	1	Televisi	Dibuang setelah	5	4	4	4	2	Ya	Ya	Jarang (<50%)	Mengurangi pen	19	Tidak	Ya	Ya
1-3x sepekan	32	4	4	4	4	5	4	Internet, Lebel p	Dibuang setelah	4	4	4	4	4	Ya	Ya	Jarang (<50%)	Mengurangi pen	20	Tidak	Ya	Ya
Tidak pernah	8	4	3	4	4	3	4	Internet	Dibuang setelah	4	4	4	4	4	Ya	Ya	Jarang (<50%)	Menghentikan k	20	Tidak	Ya	Ya
4-6x sepekan	27	3	4	5	3	3	4	Lebel pada botol	Diisi ulang	3	3	4	3	3	Ya	Ya	Terkadang (>50%	Mengurangi pen	16	Tidak	Tidak	Ya
1-3x sebulan	26	4	4	4	4	4	4	Televisi, Internet	Dibuang setelah	4	4	4	4	4	Ya	Ya	Jarang (<50%)	Tetap mengguna	20	Tidak	Ya	Ya
1-3x sepekan	21	4	5	5	5	5	5	Internet	Dibuang setelah	5	4	5	5	4	Ya	Ya	Tidak pernah	Menghentikan k	23	Tidak	Ya	Ya
Tidak pernah	26	4	4	4	4	4	4	Televisi, Internet	Digunakan untuk	5	5	5	5	5	Ya	Ya	Selalu	Mengurangi pen	25	Ya	Ya	Ya
1-3x sepekan	29	4	4	5	5	5	5	Internet, Lebel p	Dibuang setelah	5	5	5	5	5	Ya	Ya	Jarang (<50%)	Menghentikan k	25	Ya	Ya	Ya
<1x sebulan	27	4	4	4	5	2	2	Internet	Dibuang setelah	4	4	4	4	4	Ya	Ya	Jarang (<50%)	Mengurangi pen	20	Tidak	Ya	Ya
4-6x sepekan	31	4	4	4	5	3	4	Internet	Dibuang setelah	4	4	5	4	4	Ya	Ya	Jarang (<50%)	Menghentikan k	22	Tidak	Ya	Ya
<1x sebulan	19	4	3	4	4	4	3	Internet, Lebel p	Dibuang setelah	5	5	5	5	5	Ya	Ya	Terkadang (>50%	Mengurangi pen	25	Ya	Ya	Ya
1-3x sepekan	32	5	5	5	5	5	5	Televisi	Diisi ulang	5	5	5	3	3	Ya	Ya	Terkadang (>50%	Mengurangi pen	21	Ya	Ya	Ya
<1x sebulan	20	5	4	5	5	5	5	Televisi, Internet	Digunakan untuk	5	5	5	5	5	Ya	Ya	Terkadang (>50%	Mengurangi Pen	25	Ya	Ya	Ya
1-3x sebulan	31	5	5	5	5	5	5	Televisi, Internet	Dibuang setelah	5	5	5	5	5	Ya	Ya	Tidak pernah	Menghentikan k	25	Tidak	Ya	Ya
1-3x sebulan	24	5	5	5	5	5	2	Internet, Lebel p	Dibuang setelah	5	5	5	5	5	Ya	Ya	Jarang (<50%)	Mengurangi pen	25	Tidak	Ya	Ya
<1x sebulan	28	5	5	5	5	4	4	Internet, Lebel p	Digunakan untuk	5	5	5	5	5	Ya	Ya	Terkadang (>50%	Mengurangi pen	25	Ya	Ya	Ya
>3x sehari	43	4	4	5	4	3	4	Internet, Lebel p	Digunakan untuk	5	4	5	5	4	Ya	Ya	Terkadang (>50%	Mengurangi pen	23	Tidak	Ya	Ya
1-3x sebulan	25	5	5	5	5	5	3	Buku/Jurnal, Inte	Dibuang setelah	5	5	5	5	1	Ya	Ya	Terkadang (>50%	Mengurangi pen	21	Tidak	Ya	Ya
Tidak pernah	8	5	4	5	5	5	5	Televisi	Dibuang setelah	3	3	3	3	3	Ya	Ya	Jarang (<50%)	Mengurangi pen	15	Tidak	Ya	Ya
1-3x sebulan	29	5	4	5	5	5	5	Internet	Dibuang setelah	5	4	5	5	5	Ya	Ya	Terkadang (>50%	Mengurangi pen	24	Tidak	Tidak	Ya
1-3x sebulan	38	5	2	3	5	1	2	Lainnya	Dibuang setelah	5	5	5	5	5	Ya	Ya	Tidak pernah	Mengurangi pen	25	Tidak	Ya	Ya
4-6x sepekan	18	3	4	4	5	3	3	Lebel pada botol	Dibuang setelah	5	4	5	5	3	Ya	Ya	Jarang (<50%)	Mengurangi pen	22	Tidak	Ya	Ya
4-6x sepekan	38	4	5	5	5	4	3	Buku/Jurnal, Inte	Dibuang setelah	5	4	5	4	2	Ya	Ya	Jarang (<50%)	Mengurangi pen	20	Tidak	Ya	Ya
Tidak pernah	24	4	3	4	4	4	4	Televisi, Buku/Ju	Diisi Ulang	4	4	4	4	4	Ya	Ya	Terkadang (>50%	Mengurangi Pen	20	Tidak	Ya	Ya
1-3x sepekan	33	5	2	5	4	4	2	Televisi, Internet	Dibuang setelah	5	5	5	4	3	Ya	Ya	Jarang (<50%)	Mengurangi Pen	22	Tidak	Ya	Ya
4-6x sepekan	12	5	5	5	5	5	5	Televisi, Internet	Dibuang setelah	5	3	2	5	5	Ya	Ya	Jarang (<50%)	Mengurangi pen	20	Ya	Ya	Ya
4-6x sepekan	12	3	5	5	5	4	1	Internet, Lainnya	Diisi ulang	3	5	5	5	5	Ya	Ya	Terkadang (>50%	Menghentikan k	23	Tidak	Ya	Ya
>3x sehari	25	3	3	5	5	5	5	Lebel pada botol	Dibuang setelah	5	5	5	5	3	Tidak tahu	Tidak	Tidak pernah	Mengurangi Pen	23	Ya	Ya	Ya
1-3x sebulan	29	5	5	5	5	4	5	Televisi, Internet	Digunakan untuk	5	5	5	5	5	Ya	Ya	Jarang (<50%)	Mengurangi pen	25	Ya	Ya	Ya
Tidak pernah	32	5	3	4	4	3	3	Internet, Lebel p	Digunakan untuk	4	5	4	5	5	Ya	Ya	Jarang (<50%)	Mengurangi Pen	23	Tidak	Ya	Ya
>3x sehari	46	3	5	5	4	4	4	Buku/Jurnal, Inte	Digunakan untuk	5	5	5	5	3	Ya	Ya	Terkadang (>50%	Mengurangi pen	23	Tidak	Ya	Ya
Tidak pernah	19	5	4	5	5	5	5	Televisi, Internet	Digunakan untuk	5	5	5	5	5	Ya	Ya	Terkadang (>50%	Mengurangi Pen	25	Tidak	Ya	Ya
<1x sebulan	28	4	4	4	4	4	4	Koran, Internet	Dibuang setelah	5	5	5	5	5	Ya	Ya	Tidak pernah	Mengurangi pen	25	Ya	Ya	Ya
>3x sehari	48	5	5	5	5	5	5	Televisi	Dibuang setelah	5	5	5	5	5	Ya	Ya	Selalu	Mengurangi pen	25	Ya	Ya	Ya
1-3x sebulan	34	5	3	5	4	2	3	Internet, Teman/	Diisi ulang	5	5	5	5	5	Ya	Ya	Selalu	Mengurangi pen	25	Ya	Ya	Ya
<1x sebulan	28	5	4	3	4	4	2	Internet	Dibuang setelah	4	4	4	4	4	Tidak tahu	Ya	Tidak pernah	Mengurangi pen	20	Ya	Ya	Ya
Tidak pernah	19	5	1	1	5	1	1	Lainnya	Dibuang setelah	5	5	5	1	1	Ya	Ya	Tidak pernah	Mengurangi pen	17	Tidak	Ya	Ya
>3x sehari	14	5	2	2	5	3	3	Televisi	Dibuang setelah	5	5	5	5	5	Ya	Ya	Tidak pernah	Mengurangi pen	25	Tidak	Ya	Ya
<1x sebulan	28	4	4	4	4	5	3	Televisi, Buku/Ju	Diisi Ulang	4	4	4	4	4	Ya	Ya	Terkadang (>50%	Mengurangi Pen	20	Tidak	Ya	Ya
Tidak pernah	32	4	2	4	4	3	3	Internet	Digunakan untuk	4	4	4	4	4	Ya	Ya	Terkadang (>50%	Mengurangi pen	20	Tidak	Ya	Ya
<1x sebulan	22	4	4	4	4	4	4	Internet, Lebel p	Dibuang setelah	4	4	4	4	4	Ya	Ya	Tidak pernah	Mengurangi pen	20	Tidak	Ya	Ya
<1x sebulan	30	5	5	5	5	5	3	Televisi, Koran, I	Digunakan untuk	5	4	5	5	4	Ya	Ya	Terkadang (>50%	Mengurangi pen	23	Ya	Ya	Ya
Tidak pernah	20	3	5	5	5	5	3	Buku/Jurnal, Inte	Digunakan untuk	4	3	4	4	3	Ya	Ya	Selalu	Mengurangi pen	18	Ya	Ya	Ya
<1x sebulan	29	5	5	5	5	5	5	Televisi, Internet	Diisi ulang	4	4	4	4	4	Ya	Ya	Terkadang (>50%	Mengurangi pen	20	Ya	Ya	Ya
Tidak pernah	34	4	5	5	5	5	5	Televisi, Internet	Dibuang setelah	5	5	4	4	4	Ya	Ya	Terkadang (>50%	Mengurangi pen	22	Ya	Ya	Ya
1-3x sehari	32	4	5	4	5	5	4	Lebel pada botol	Dibuang setelah	5	5	4	5	5	Ya	Ya	Terkadang (>50%	Tetap mengguna	24	Tidak	Tidak	Tidak
1-3x sehari	20	4	5	5	5	5	3	Internet, Teman/	Dibuang setelah	4	4	4	4	4	Ya	Ya	Tidak pernah	Tetap mengguna	20	Tidak	Tidak	Ya
1-3x sepekan	27	5	5	5	5	5	3	Internet	Dibuang setelah	4	5	5	5	5	Ya	Ya	Jarang (<50%)	Mengurangi pen	24	Tidak	Ya	Ya
1-3x sebulan	25	5	5	5	5	5	3	Televisi, Internet	Dibuang setelah	5	5	5	5	4	Tidak tahu	Ya	Jarang (<50%)	Mengurangi pen	24	Tidak	Ya	Ya
1-3x sebulan	27	5	4	5	4	5	4	Internet	Diisi ulang	5	5	5	5	2	Ya	Ya	Terkadang (>50%	Mengurangi pen	22	Ya	Ya	Ya
Tidak pernah	31	3	5	5	4	5	5	Televisi, Internet	Digunakan untuk	2	4	4	1	1	Ya	Ya	Terkadang (>50%	Tetap mengguna	12	Ya	Ya	Ya
4-6x sepekan	41	4	3	4	5	4	3	Televisi, Internet	Dibuang setelah	4	5	3	3	3	Ya	Ya	Jarang (<50%)	Mengurangi Pen	18	Tidak	Ya	Ya
4-6x sepekan	28	3	3	5	4	3	4	Televisi, Internet	Dibuang setelah	4	5	5	4	3	Ya	Ya	Terkadang (>50%	Mengurangi Pen	21	Tidak	Ya	Ya
1-3x sepekan	41	3	3	3	2	3	3	Televisi, Buku/Ju	Diisi Ulang	4	5	5	5	2	Ya	Ya	Terkadang (>50%	Tetap mengguna	21	Tidak	Tidak	Tidak
4-6x sepekan	40	5	5	5	3	1	5	Lainnya	Dibuang setelah	5	5	5	5	5	Ya	Ya	Selalu	Mengurangi Pen	25	Tidak	Ya	Ya
1-3x sepekan	33	2	3	5	5	2	3	Lebel pada botol	Diisi Ulang	3	5	4	5	3	Tidak tahu	Tidak tahu	Jarang (<50%)	Mengurangi Pen	20	Tidak	Ya	Ya
Tidak pernah	23	5	5	5	5	5	5	Internet, Lebel p	Digunakan untuk	5	5	5	5	5	Ya	Ya	Selalu	Menghentikan k	25	Ya	Ya	Ya
1-3x sepekan	28	3	3	3	3	3	3	Lebel pada botol	Dibuang setelah	4	4	4	3	4	Tidak tahu	Tidak tahu	Tidak pernah	Tetap mengguna	19	Ya	Tidak	Tidak
1-3x sebulan	30	4	4	5	3	5	5	Buku/Jurnal, Inte	Dibuang setelah	5	5	5	5	5	Ya	Ya	Jarang (<50%)	Mengurangi pen	25	Tidak	Ya	Ya
Tidak pernah	31	5	5	5	5	5	3	Internet	Dibuang setelah	5	5	5	5	5	Ya	Ya	Jarang (<50%)	Mengurangi pen	25	Tidak	Ya	Ya
<1x sebulan	20	5	5	5	5	5	5	Lebel pada botol	Digunakan untuk	5	5	5	5	5	Ya	Ya	Terkadang (>50%	Mengurangi pen	25	Ya	Ya	Ya
1-3x sehari	21	3	3	4	5	5	5	Televisi, Koran, I	Dibuang setelah	3	3	5	5	5	Ya	Ya	Tidak pernah	Menghentikan k	21	Tidak	Ya	Ya
4-6x sepekan	28	5	5	5	5	3	5	Televisi, Internet	Digunakan untuk	5	5	5	4	3	Ya	Ya	Jarang (<50%)	Menghentikan k	22	Tidak	Ya	Ya
1-3x sebulan	18	5	3	5	5	2	5	Buku/Jurnal, Inte	Dibuang setelah	5	5	5	5	2	Ya	Ya	Tidak pernah	Menghentikan k	22	Tidak	Ya	Ya
1-3x sebulan	32	3	4	4	5	2	4	Teman/Kerabat	Dibuang setelah	4	5	4	4	3	Ya	Ya	Jarang (<50%)	Mengurangi pen	20	Ya	Ya	Ya
1-3x sepekan	33	5	4	4	4	5	4	Televisi	Dibuang setelah	4	5	4	5	3	Ya	Ya	Terkadang (>50%	Mengurangi pen	21	Tidak	Ya	Ya
4-6x sepekan	35	3	4	5	5	5	3	Televisi, Internet	Dibuang setelah	5	5	5	5	4	Ya	Ya	Jarang (<50%)	Mengurangi pen	24	Tidak	Ya	Ya
4-6x sepekan	12	5	5	5	5	3	3	Buku/Jurnal, Inte	Diisi ulang	5	5	5	5	3	Ya	Ya	Jarang (<50%)	Menghentikan k	23	Tidak	Ya	Ya
1-3x sebulan	34	5	5	5	5	5	5	Televisi, Internet	Dibuang setelah	5	5	5	5	5	Ya	Ya	Jarang (<50%)	Mengurangi pen	25	Tidak	Ya	Ya
1-3x sepekan	38	5	3	4	4	4	3	Televisi, Internet	Digunakan untuk	4	4	4	4	3	Ya	Ya	Jarang (<50%)	Menghentikan k	19	Tidak	Ya	Ya
Tidak pernah	32	4	4	5	5	5	3	Televisi, Internet	Diisi ulang	4	4	4	4	4	Ya	Ya	Jarang (<50%)	Tetap mengguna	20	Tidak	Ya	Ya
1-3x sepekan	32	3	3	5	4	4	5	Teman/Kerabat,	Dibuang setelah	5	5	5	5	4	Ya	Ya	Jarang (<50%)	Menghentikan k	24	Ya	Ya	Ya
4-6x sepekan	41	4	4	5	5	4	4	Televisi, Lebel p	Dibuang setelah	5	4	3	4	4	Ya	Ya	Jarang (<50%)	Menghentikan k	20	Tidak	Ya	Ya
1-3x sebulan	24	4	3	3	4	3	3	Lebel pada botol	Dibuang setelah	3	4	4	3	3	Tidak tahu	Tidak tahu	Terkadang (>50%	Menghentikan k	17	Tidak	Ya	Ya
Tidak pernah	22	3	3	3	3	3	3	Televisi	Diisi ulang	5	5	5	5	3	Ya	Ya	Jarang (<50%)	Mengurangi pen	23	Tidak	Ya	Ya
4-6x sepekan	26	4	4	5	5	4	4	Televisi, Internet	Digunakan untuk	5	5	5	4	3	Ya	Ya	Terkadang (>50%	Mengurangi pen	22	Tidak	Ya	Ya
1-3x sebulan	26	2	5	5	4	5	3	Internet, Lebel p	Dibuang setelah	4	4	4	5	3	Ya	Ya	Jarang (<50%)	Mengurangi pen	20	Ya	Ya	Ya

1-3x sepekan	26	5	4	4	5	3	2	Televisi, Buku/Ju	Digunakan untuk	5	2	5	5	4	Ya	Ya	Terkadang (>50%	Menghentikan ko	21	Ya	Ya	Ya
1-3x sebulan	40	2	5	5	5	5	4	Buku/Jurnal, Inte	Dibuang setelah	5	5	5	5	5	Ya	Ya	Jarang (<50%)	Mengurangi pen	25	Tidak	Ya	Ya
1-3x sepekan	34	4	4	4	4	4	4	Internet, Lebel p	Dibuang setelah	5	5	5	5	5	Ya	Ya	Jarang (<50%)	Mengurangi pen	25	Tidak	Ya	Ya
>3x sehari	42	4	5	5	5	5	5	Lebel pada botol	Diisi ulang	5	5	5	5	5	Ya	Ya	Jarang (<50%)	Mengurangi pen	25	Tidak	Ya	Ya
<1x sebulan	37	3	3	4	4	3	3	Televisi, Lebel pa	Digunakan untuk	3	4	5	3	3	Ya	Tidak tahu	Selalu	Tetap mengguna	18	Tidak	Ya	Ya
>3x sehari	36	5	5	5	5	5	5	Internet	Diisi ulang	5	5	5	5	3	Ya	Ya	Terkadang (>50%	Mengurangi pen	23	Ya	Ya	Ya
<1x sebulan	28	2	2	5	1	5	5	Televisi, Buku/Ju	Dibuang setelah	5	5	4	5	5	Ya	Ya	Terkadang (>50%	Mengurangi pen	24	Ya	Ya	Ya
<1x sebulan	24	3	3	3	3	3	3	Internet	Diisi ulang	3	3	3	3	3	Ya	Ya	Terkadang (>50%	Tetap mengguna	15	Tidak	Ya	Ya
4-6x sepekan	26	4	3	4	4	4	4	Lainnya	Dibuang setelah	4	5	4	4	4	Ya	Ya	Terkadang (>50%	Mengurangi pen	21	Tidak	Ya	Ya
Tidak pernah	22	3	3	4	4	4	3	Internet	Digunakan untuk	4	4	4	4	4	Ya	Ya	Terkadang (>50%	Mengurangi pen	20	Ya	Ya	Ya
1-3x sehari	43	3	3	3	3	3	3	Televisi, Internet	Dibuang setelah	3	3	3	3	3	Ya	Ya	Terkadang (>50%	Tetap mengguna	15	Ya	Ya	Ya
1-3x sebulan	31	4	4	4	4	4	4	Televisi, Internet	Dibuang setelah	5	5	4	4	3	Ya	Ya	Selalu	Mengurangi pen	21	Tidak	Ya	Ya
<1x sebulan	28	5	4	4	5	2	3	Lebel pada botol	Dibuang setelah	4	5	5	5	5	Ya	Ya	Tidak pernah	Mengurangi pen	24	Tidak	Ya	Ya
1-3x sepekan	40	3	3	5	5	5	2	Televisi, Lebel pa	Diisi ulang	5	5	5	5	3	Ya	Ya	Jarang (<50%)	Mengurangi pen	23	Ya	Ya	Ya
1-3x sepekan	27	3	4	4	4	3	3	Televisi, Buku/Ju	Dibuang setelah	4	4	4	4	3	Tidak tahu	Ya	Jarang (<50%)	Mengurangi pen	19	Ya	Ya	Ya
Tidak pernah	26	5	5	5	5	5	1	Lainnya	Digunakan untuk	1	1	1	1	1	Tidak	Tidak	Selalu	Tetap mengguna	5	Tidak	Ya	Ya
Tidak pernah	14	4	1	4	3	3	1	Televisi	Dibuang setelah	5	5	5	5	1	Ya	Ya	Selalu	Mengurangi pen	21	Ya	Tidak	Ya
1-3x sepekan	30	4	4	4	4	4	4	Internet, Lebel p	Digunakan untuk	5	4	4	5	4	Ya	Ya	Terkadang (>50%	Menghentikan ko	22	Ya	Ya	Ya
Tidak pernah	36	5	4	3	4	4	3	Lebel pada botol	Dibuang setelah	5	5	5	5	4	Ya	Ya	Terkadang (>50%	Mengurangi Pen	24	Tidak	Ya	Ya
1-3x sepekan	32	4	4	4	4	4	4	Televisi, Internet	Digunakan untuk	3	3	3	3	3	Ya	Ya	Terkadang (>50%	Mengurangi Pen	15	Ya	Ya	Ya
1-3x sepekan	42	5	5	5	5	5	5	Internet, Lebel p	Digunakan untuk	5	5	5	5	5	Ya	Ya	Selalu	Mengurangi pen	25	Ya	Ya	Ya
1-3x sebulan	25	4	3	3	4	3	3	Televisi, Internet	Dibuang setelah	4	4	4	4	4	Ya	Ya	Jarang (<50%)	Mengurangi pen	20	Tidak	Ya	Tidak
<1x sebulan	35	4	5	4	3	4	4	Lebel pada botol	Dibuang setelah	2	5	2	2	1	Tidak tahu	Tidak tahu	Tidak pernah	Tetap mengguna	12	Ya	Ya	Ya
1-3x sebulan	30	4	4	5	5	5	5	Televisi, Buku/Ju	Digunakan untuk	4	4	4	4	3	Ya	Ya	Jarang (<50%)	Mengurangi pen	19	Ya	Ya	Ya
1-3x sebulan	20	5	3	4	4	3	3	Internet	Dibuang setelah	4	4	4	3	3	Ya	Ya	Terkadang (>50%	Mengurangi pen	18	Tidak	Ya	Ya
<1x sebulan	35	4	3	4	5	3	2	Internet	Diisi ulang	4	4	4	5	3	Ya	Ya	Terkadang (>50%	Mengurangi pen	20	Ya	Ya	Ya
<1x sebulan	32	3	4	4	5	4	3	Lebel pada botol	Dibuang setelah	3	4	4	4	3	Ya	Ya	Tidak pernah	Menghentikan ko	18	Tidak	Ya	Ya
1-3x sehari	29	3	4	4	5	4	5	Televisi, Internet	Diisi ulang	5	3	4	5	3	Ya	Ya	Selalu	Menghentikan ko	20	Tidak	Ya	Ya
1-3x sebulan	35	4	3	3	5	3	3	Internet, Lebel p	Digunakan untuk	5	5	4	5	4	Ya	Ya	Terkadang (>50%	Mengurangi pen	23	Ya	Ya	Ya
4-6x sepekan	39	5	5	5	4	4	4	Televisi, Internet	Dibuang setelah	5	5	3	5	2	Ya	Tidak	Terkadang (>50%	Tetap mengguna	20	Tidak	Ya	Ya
1-3x sehari	43	4	4	2	5	5	4	Televisi, Buku/Ju	Digunakan untuk	5	5	5	5	5	Ya	Ya	Jarang (<50%)	Mengurangi Pen	25	Ya	Ya	Ya
1-3x sehari	27	5	4	3	4	2	4	Lebel pada botol	Digunakan untuk	5	5	5	5	4	Ya	Ya	Jarang (<50%)	Mengurangi Pen	24	Tidak	Ya	Ya
1-3x sepekan	33	4	5	3	4	4	2	Televisi, Internet	Digunakan untuk	5	4	5	4	4	Ya	Ya	Jarang (<50%)	Mengurangi Pen	22	Ya	Ya	Ya
1-3x sebulan	18	4	5	5	4	4	4	Televisi, Internet	Dibuang setelah	4	4	4	5	3	Ya	Ya	Jarang (<50%)	Tetap mengguna	20	Tidak	Ya	Tidak
Tidak pernah	25	4	4	4	4	4	4	Lainnya	Dibuang setelah	5	4	4	5	3	Ya	Ya	Terkadang (>50%	Mengurangi pen	21	Ya	Ya	Ya
1-3x sehari	45	5	5	5	5	3	3	Televisi, Internet	Dibuang setelah	5	5	5	5	5	Ya	Ya	Jarang (<50%)	Mengurangi pen	25	Tidak	Ya	Ya
Tidak pernah	29	4	4	5	5	5	5	Televisi, Internet	Dibuang setelah	5	4	5	5	3	Ya	Ya	Terkadang (>50%	Mengurangi pen	22	Tidak	Ya	Ya
1-3x sebulan	36	4	4	4	4	5	4	Internet, Lebel p	Dibuang setelah	5	5	5	5	3	Ya	Ya	Jarang (<50%)	Tetap mengguna	23	Ya	Ya	Ya
1-3x sepekan	21	4	4	5	5	5	5	Televisi, Buku/Ju	Diisi ulang	5	5	5	5	5	Ya	Ya	Jarang (<50%)	Mengurangi pen	25	Tidak	Ya	Ya
<1x sebulan	11	2	2	1	3	1	1	Lebel pada botol	Dibuang setelah	5	5	5	5	3	Ya	Ya	Tidak pernah	Mengurangi pen	23	Tidak	Ya	Ya
>3x sehari	56	3	3	3	3	3	3	Televisi	Dibuang setelah	5	5	5	5	5	Ya	Ya	Jarang (<50%)	Menghentikan ko	25	Tidak	Ya	Ya
>3x sehari	40	1	5	5	5	3	3	Internet, Lebel p	Dibuang setelah	5	4	5	5	4	Ya	Ya	Tidak pernah	Mengurangi pen	23	Tidak	Tidak	Ya
<1x sebulan	12	5	5	5	5	5	5	Televisi	Diisi ulang	5	5	5	5	5	Ya	Ya	Selalu	Mengurangi pen	25	Ya	Ya	Ya
4-6x sepekan	45	4	4	5	4	4	4	Internet, Lebel p	Dibuang setelah	5	4	5	5	3	Ya	Ya	Terkadang (>50%	Mengurangi pen	22	Ya	Ya	Ya
>3x sehari	39	3	3	3	3	3	3	Internet, Lebel p	Digunakan untuk	3	3	4	3	3	Ya	Ya	Jarang (<50%)	Mengurangi pen	16	Ya	Tidak	Ya
1-3x sehari	33	5	4	4	5	4	4	Internet, Lainnya	Dibuang setelah	5	2	4	5	3	Tidak tahu	Ya	Tidak pernah	Mengurangi Pen	19	Tidak	Ya	Ya
<1x sebulan	30	4	4	5	4	3	4	Lebel pada botol	Dibuang setelah	5	4	4	4	3	Tidak tahu	Ya	Tidak pernah	Mengurangi Pen	20	Ya	Ya	Tidak
1-3x sehari	46	5	3	4	5	5	2	Koran, Teman/K	Dibuang setelah	4	3	4	3	2	Tidak	Tidak tahu	Tidak pernah	Tetap mengguna	16	Tidak	Tidak	Tidak
>3x sehari	34	4	4	4	4	4	4	Televisi, Internet	Diisi Ulang	5	5	5	5	5	Ya	Ya	Terkadang (>50%	Mengurangi Pen	25	Ya	Ya	Ya
1-3x sehari	39	4	4	4	4	4	4	Televisi, Internet	Dibuang setelah	4	4	3	4	3	Tidak	Tidak	Terkadang (>50%	Mengurangi pen	18	Tidak	Ya	Ya
Tidak pernah	8	3	3	3	3	3	3	Lainnya	Digunakan untuk	3	3	3	3	3	Ya	Ya	Terkadang (>50%	Mengurangi pen	15	Ya	Ya	Ya
1-3x sehari	19	4	4	5	5	3	3	Televisi, Koran, I	Dibuang setelah	4	4	4	4	3	Tidak tahu	Tidak tahu	Jarang (<50%)	Tetap mengguna	19	Ya	Tidak	Tidak
1-3x sehari	48	4	4	5	4	4	5	Televisi, Internet	Dibuang setelah	5	5	5	5	5	Ya	Ya	Jarang (<50%)	Mengurangi pen	25	Ya	Ya	Ya
Tidak pernah	25	5	5	5	5	5	5	Televisi, Buku/Ju	Dibuang setelah	5	5	5	5	5	Ya	Ya	Selalu	Mengurangi pen	25	Ya	Ya	Ya
>3x sehari	26	4	4	5	4	4	4	Televisi, Internet	Dibuang setelah	5	5	5	5	4	Ya	Ya	Jarang (<50%)	Mengurangi pen	24	Tidak	Ya	Ya
1-3x sepekan	42	4	4	5	5	5	4	Internet, Lebel p	Digunakan untuk	5	5	5	4	4	Ya	Ya	Terkadang (>50%	Mengurangi pen	23	Ya	Ya	Ya
Tidak pernah	41	5	4	3	5	4	3	Televisi, Internet	Dibuang setelah	5	5	5	5	5	Ya	Ya	Terkadang (>50%	Menghentikan ko	25	Tidak	Ya	Ya
1-3x sehari	36	3	5	5	5	4	4	Internet, Lebel p	Dibuang setelah	5	4	5	5	3	Ya	Ya	Terkadang (>50%	Mengurangi pen	22	Tidak	Ya	Ya
>3x sehari	35	5	5	5	5	5	5	Televisi, Internet	Digunakan untuk	5	5	5	5	5	Ya	Ya	Jarang (<50%)	Mengurangi pen	25	Ya	Ya	Ya
Tidak pernah	38	5	4	5	5	3	3	Internet, Lebel p	Dibuang setelah	5	5	5	5	3	Ya	Ya	Terkadang (>50%	Mengurangi pen	23	Ya	Ya	Tidak
1-3x sepekan	40	4	5	5	4	5	5	Internet, Lebel p	Dibuang setelah	5	5	5	3	3	Ya	Ya	Tidak pernah	Mengurangi pen	21	Ya	Ya	Ya
Tidak pernah	21	4	4	5	5	4	5	Televisi, Internet	Diisi ulang	4	4	3	3	3	Ya	Ya	Tidak pernah	Mengurangi pen	17	Tidak	Ya	Ya
<1x sebulan	18	5	5	5	5	5	5	Televisi	Dibuang setelah	5	5	4	4	3	Ya	Ya	Terkadang (>50%	Mengurangi pen	21	Ya	Ya	Ya
Tidak pernah	38	4	4	4	4	4	4	Internet	Digunakan untuk	4	4	4	4	4	Ya	Ya	Terkadang (>50%	Mengurangi pen	20	Tidak	Ya	Ya
1-3x sepekan	44	5	5	5	5	5	5	Televisi	Dibuang setelah	5	5	5	5	3	Ya	Ya	Terkadang (>50%	Mengurangi pen	23	Ya	Ya	Ya
<1x sebulan	46	5	5	5	5	5	5	Lainnya	Diisi ulang	5	5	5	5	5	Tidak	Tidak	Terkadang (>50%	Mengurangi pen	25	Tidak	Tidak	Tidak
1-3x sepekan	28	2	2	2	2	2	3	Lainnya	Dibuang setelah	2	3	2	3	3	Tidak	Tidak	Terkadang (>50%	Menghentikan ko	13	Tidak	Tidak	Ya
Tidak pernah	13	4	4	4	3	3	4	Lainnya	Dibuang setelah	4	4	4	4	3	Ya	Ya	Jarang (<50%)	Mengurangi pen	19	Ya	Ya	Ya
Tidak pernah	38	4	4	5	5	3	5	Televisi, Internet	Dibuang setelah	4	5	5	4	4	Ya	Ya	Selalu	Mengurangi pen	22	Ya	Ya	Ya
4-6x sepekan	45	5	3	5	5	5	5	Televisi, Koran	Diisi ulang	5	5	5	5	5	Ya	Ya	Jarang (<50%)	Mengurangi pen	25	Ya	Ya	Ya
1-3x sepekan	26	3	4	5	5	5	5	Internet	Dibuang setelah	5	4	3	5	5	Ya	Ya	Jarang (<50%)	Mengurangi pen	22	Ya	Ya	Ya
4-6x sepekan	39	4	4	4	5	2	2	Internet	Dibuang setelah	4	4	4	4	2	Ya	Ya	Jarang (<50%)	Mengurangi pen	18	Tidak	Ya	Ya
1-3x sebulan	36	5	5	5	5	5	5	Internet	Dibuang setelah	5	4	5	5	4	Ya	Ya	Jarang (<50%)	Mengurangi pen	23	Ya	Ya	Ya
1-3x sehari	13	4	2	5	5	3	3	Internet, Lebel p	Dibuang setelah	4	5	4	4	3	Ya	Ya	Jarang (<50%)	Menghentikan ko	20	Tidak	Ya	Ya
Tidak pernah	39	3	5	5	3	5	2	Lebel pada botol	Dibuang setelah	4	4	4	4	4	Ya	Ya	Jarang (<50%)	Mengurangi pen	20	Tidak	Ya	Ya
1-3x sepekan	44	4	5	5	5	5	4	Internet, Lebel p	Dibuang setelah	5	4	4	4	4	Ya	Ya	Jarang (<50%)	Mengurangi pen	21	Tidak	Ya	Ya
1-3x sehari	50	2	1	3	1	1	5	Internet, Lebel p	Digunakan untuk	5	5	5	5	5	Ya	Ya	Terkadang (>50%	Menghentikan ko	25	Ya	Ya	Ya
4-6x sepekan	44	4	3	4	4	4	4	Televisi, Internet	Dibuang setelah	4	4	4	4	3	Ya	Ya	Terkadang (>50%	Menghentikan ko	19	Ya	Ya	Ya
Tidak pernah	26	3	3	5	4	3	3	Televisi, Koran, I	Dibuang setelah	5	5	5	5	3	Ya	Ya	Jarang (<50%)	Mengurangi pen	23	Tidak	Ya	Ya
1-3x sebulan	27	4	5	5	4	5	4	Internet, Lebel p	Dibuang setelah	5	5	5	5	5	Ya	Ya	Jarang (<50%)	Mengurangi pen	25	Tidak	Ya	Ya
1-3x sepekan	31	5	4	5	5	5	4	Internet, Teman/	Dibuang setelah	5	5	5	5	5	Ya	Ya	Jarang (<50%)	Mengurangi pen	25	Tidak	Ya	Ya
1-3x sehari	26	5	5	5	5	4	4	Buku/Jurnal, Inte	Dibuang setelah	5	5	5	5	5	Ya	Ya	Tidak pernah	Mengurangi pen	25	Tidak	Ya	Ya
1-3x sebulan	24	5	5	4	5	4	5	Internet	Dibuang setelah	5	5	5	5	5	Ya	Ya	Jarang (<50%)	Mengurangi pen	25	Tidak	Ya	Ya
1-3x sepekan	36	5	5	4	4	3	4	Lebel pada botol	Dibuang setelah	5	5	5	5	4	Ya	Ya	Tidak pernah	Mengurangi pen	24	Tidak	Ya	Ya
<1x sebulan	23	5	5	5	4	4	5	Internet, Lebel p	Dibuang setelah	5	5	5	5	5	Ya	Ya	Terkadang (>50%	Mengurangi pen	25	Ya	Ya	Ya
>3x sehari	49	5	1	3	5	1	5	Televisi	Dibuang setelah	5	5	5	5	1	Ya	Ya	Tidak pernah	Tetap mengguna	21	Tidak	Ya	Ya
1-3x sepekan	25	3	5	5	5	5	4	Lebel pada botol	Dibuang setelah	3	5	4	4	5	Ya	Ya	Jarang (<50%)	Mengurangi pen	21	Ya	Ya	Ya
Tidak pernah	35	4	4	5	5	5	5	Televisi	Digunakan untuk	4	4	4	4	4	Ya	Ya	Jarang (<50%)	Mengurangi pen	20	Tidak	Ya	Ya
4-6x sepekan	37	4	4	5	4	4	4	Lebel pada botol	Diisi Ulang	3	5	4	4	4	Ya	Tidak	Terkadang (>50%	Mengurangi Pen	20	Tidak	Ya	Ya
1-3x sepekan	30	3	3	3	3	3	4	Internet	Dibuang setelah	5	5	5	5	5	Ya	Ya	Selalu	Mengurangi Pen	25	Tidak	Ya	Ya
1-3x sebulan	17	5	5	5	5	5	5	Internet	Dibuang setelah	3	5	5	5	3	Ya	Ya	Terkadang (>50%	Mengurangi pen	21	Tidak	Ya	Ya
1-3x sehari	51	4	5	5	5	5	5	Televisi, Internet	Digunakan untuk	5	5	5	5	4	Ya	Ya	Selalu	Mengurangi pen	24	Ya	Ya	Ya
Tidak pernah	38	4	1	3	5	3	3	Internet, Lebel p	Diisi ulang	5	4	5	5	4	Ya	Ya	Jarang (<50%)	Mengurangi pen	23	Tidak	Ya	Ya
1-3x sebulan		3	3	5	3	3	3	Buku/Jurnal, Inte	Digunakan untuk	5	5	5	5	5	Ya	Ya	Terkadang (>50%	Mengurangi pen	80	Ya	Ya	Ya
Tidak pernah	37	5	4	5	5	4	5	Lebel pada botol	Digunakan untuk	5	5	5	5	5	Ya	Ya	Terkadang (>50%	Mengurangi pen	25	Tidak	Ya	Ya
Tidak pernah	24	3	1	5	5	5	5	Televisi, Internet	Dibuang setelah	5	5	5	5	5	Ya	Ya	Jarang (<50%)	Mengurangi pen	25	Ya	Ya	Ya
Tidak pernah	42	4	4	4	4	3	5	Internet, Lebel p	Dibuang setelah	4	4	4	4	4	Ya	Ya	Jarang (<50%)	Mengurangi pen	20	Tidak	Ya	Ya
>3x sehari	38	4	4	5	5	2	4	Televisi, Buku/Ju	Dibuang setelah	4	4	4	4	4	Ya	Ya	Jarang (<50%)	Menghentikan ko	20	Tidak	Ya	Ya
>3x sehari	46	5	2	2	5	1	2	Internet	Dibuang setelah	5	5	5	5	5	Ya	Ya	Jarang (<50%)	Tetap mengguna	25	Tidak	Ya	Ya
<1x sebulan	40	4	4	4	4	4	4	Televisi	Dibuang setelah	4	4	4	4	4	Ya	Ya	Tidak pernah	Mengurangi pen	20	Tidak	Ya	Ya
1-3x sehari	42	5	4	4	5	2	3	Televisi, Buku/Ju	Diisi ulang	4	4	4	4	1	Ya	Ya	Selalu	Mengurangi pen	17	Tidak	Ya	Ya
1-3x sebulan	38	4	5	5	4	5	4	Internet, Lebel p	Digunakan untuk	5	5	5	4	4	Ya	Ya	Terkadang (>50%	Mengurangi pen	23	Ya	Ya	Ya
<1x sebulan	18	3	4	5	4	4	4	Lainnya	Diisi ulang	3	4	4	4	3	Tidak tahu	Tidak tahu	Tidak pernah	Mengurangi pen	18	Ya	Ya	Ya
4-6x sepekan	44	5	3	5	5	3	4	Televisi, Teman/I	Diisi ulang	5	5	5	5	5	Ya	Ya	Jarang (<50%)	Tetap mengguna	25	Tidak	Ya	Ya
<1x sebulan	27	4	5	5	5	5	4	Lebel pada botol	Digunakan untuk	5	5	5	4	4	Ya	Ya	Selalu	Mengurangi pen	23	Tidak	Ya	Ya
Tidak pernah	14	2	5	4	5	5	4	Buku/Jurnal, Inte	Dibuang setelah	5	4	4	4	3	Tidak	Ya	Terkadang (>50%	Menghentikan ko	20	Tidak	Ya	Ya
Tidak pernah	8	5	1	1	5	1	3	Lainnya	Dibuang setelah	5	5	5	5	5	Ya	Ya	Jarang (<50%)	Tetap mengguna	25	Tidak	Ya	Ya
>3x sehari	56	5	1	1	5	1	5	Televisi	Dibuang setelah	5	1	5	5	3	Ya	Ya	Tidak pernah	Mengurangi pen	19	Ya	Ya	Ya
1-3x sehari	42	5	4	5	5	4	4	Televisi, Internet	Dibuang setelah	5	5	5	5	5	Ya	Ya	Tidak pernah	Tetap mengguna	25	Tidak	Ya	Ya
Tidak pernah	28	3	4	5	4	4	2	Internet, Lebel p	Dibuang setelah	4	4	4	2	5	Ya	Ya	Tidak pernah	Menghentikan ko	19	Ya	Ya	Ya
1-3x sepekan	38	3	5	4	4	5	4	Internet	Dibuang setelah	5	5	5	5	5	Ya	Ya	Jarang (<50%)	Mengurangi pen	25	Tidak	Ya	Ya
1-3x sehari	42	1	2	5	5	5	3	Internet, Lebel p	Digunakan untuk	5	5	5	5	2	Ya	Ya	Selalu	Mengurangi Pen	22	Ya	Ya	Ya
<1x sebulan	44	5	5	5	5	5	3	Televisi, Buku/Ju	Digunakan untuk	5	5	4	5	3	Ya	Ya	Jarang (<50%)	Mengurangi Pen	22	Tidak	Ya	Ya
<1x sebulan	10	5	3	5	5	4	5	Koran, Internet, I	Digunakan untuk	5	5	5	5	5	Ya	Ya	Jarang (<50%)	Mengurangi Pen	25	Ya	Ya	Ya
1-3x sebulan	30	5	4	4	5	5	3	Televisi, Internet	Dibuang setelah	3	4	5	5	3	Tidak tahu	Tidak tahu	Jarang (<50%)	Mengurangi Pen	20	Tidak	Ya	Ya
>3x sehari	53	5	5	5	5	3	5	Lebel pada botol	Dibuang setelah	5	5	5	5	5	Ya	Ya	Tidak pernah	Mengurangi pen	25	Tidak	Ya	Tidak
1-3x sepekan	39	5	5	5	5	5	5	Internet, Lebel p	Digunakan untuk	5	5	5	5	4	Ya	Ya	Terkadang (>50%	Mengurangi pen	24	Tidak	Ya	Ya
1-3x sebulan	24	3	2	4	4	4	4	Buku/Jurnal, Inte	Dibuang setelah	5	5	5	5	3	Ya	Ya	Jarang (<50%)	Menghentikan ko	23	Ya	Ya	Ya
>3x sehari	48	5	5	4	5	5	3	Televisi, Internet	Dibuang setelah	3	5	4	3	3	Tidak	Ya	Jarang (<50%)	Mengurangi pen	18	Tidak	Ya	Ya
Tidak pernah	34	5	5	5	5	5	5	Lebel pada botol	Dibuang setelah	5	5	5	5	5	Ya	Ya	Tidak pernah	Menghentikan ko	25	Tidak	Ya	Ya
Tidak pernah	44	5	5	5	5	5	5	Televisi, Internet	Dibuang setelah	5	5	5	5	5	Ya	Ya	Jarang (<50%)	Mengurangi pen	25	Tidak	Tidak	Ya
>3x sehari	40	3	3	5	3	3	3	Internet, Lebel p	Diisi ulang	5	5	5	5	5	Ya	Ya	Jarang (<50%)	Mengurangi pen	25	Tidak	Ya	Ya
1-3x sehari	48	4	3	4	5	4	4	Televisi, Internet	Diisi ulang	4	4	4	4	3	Tidak tahu	Ya	Selalu	Menghentikan ko	19	Tidak	Ya	Ya
>3x sehari	39	5	5	5	5	5	5	Internet, Lebel p	Dibuang setelah	5	5	5	5	5	Ya	Ya	Jarang (<50%)	Mengurangi pen	25	Tidak	Ya	Ya
1-3x sebulan	37	5	5	5	3	5	4	Televisi	Dibuang setelah	5	5	5	1	1	Ya	Ya	Tidak pernah	Mengurangi pen	17	Tidak	Ya	Ya
1-3x sebulan	32	5	5	5	3	5	4	Televisi	Digunakan untuk	5	5	5	1	1	Ya	Ya	Selalu	Mengurangi pen	17	Tidak	Ya	Ya
1-3x sepekan	33	4	3	4	5	5	4	Televisi, Buku/Ju	Digunakan untuk	4	4	5	5	4	Ya	Ya	Selalu	Mengurangi pen	22	Ya	Ya	Ya
1-3x sehari	29	4	4	3	5	3	2	Televisi	Dibuang setelah	4	4	4	4	4	Ya	Ya	Jarang (<50%)	Menghentikan ko	20	Tidak	Ya	Ya
1-3x sehari	55	2	3	5	3	3	4	Buku/Jurnal, Inte	Dibuang setelah	4	4	4	4	4	Ya	Ya	Jarang (<50%)	Mengurangi Pen	20	Ya	Ya	Ya
>3x sehari	56	4	5	5	5	5	5	Televisi	Dibuang setelah	5	5	5	5	4	Ya	Ya	Selalu	Mengurangi Pen	24	Ya	Ya	Ya
1-3x sebulan	17	5	5	5	5	5	5	Koran, Internet, T	Digunakan untuk	4	3	3	3	3	Ya	Ya	Selalu	Mengurangi Pen	16	Ya	Ya	Ya
1-3x sebulan	24	3	5	5	5	5	5	Internet, Lebel p	Dibuang setelah	5	5	5	5	5	Ya	Ya	Jarang (<50%)	Mengurangi Pen	25	Ya	Ya	Ya
<1x sebulan	25	3	4	5	5	4	5	Televisi, Buku/Ju	Dibuang setelah	5	4	4	5	3	Ya	Ya	Terkadang (>50%	Menghentikan ko	21	Tidak	Ya	Ya
>3x sehari	56	1	2	5	5	5	5	Internet, Lebel p	Dibuang setelah	5	5	5	5	5	Ya	Ya	Terkadang (>50%	Menghentikan ko	25	Ya	Ya	Ya
1-3x sepekan	44	4	3	4	4	2	2	Lebel pada botol	Dibuang setelah	4	4	4	5	2	Ya	Ya	Jarang (<50%)	Mengurangi pen	19	Ya	Ya	Ya
>3x sehari	45	4	4	3	5	4	3	Televisi, Internet	Dibuang setelah	4	4	4	4	4	Ya	Ya	Tidak pernah	Mengurangi pen	20	Ya	Ya	Ya
<1x sebulan	41	5	5	5	5	5	5	Buku/Jurnal, Leb	Dibuang setelah	5	5	5	5	5	Ya	Ya	Jarang (<50%)	Menghentikan ko	25	Tidak	Ya	Ya
>3x sehari	56	4	4	4	4	4	4	Internet	Digunakan untuk	4	4	4	4	4	Ya	Ya	Selalu	Menghentikan ko	20	Ya	Ya	Ya
1-3x sehari	39	4	4	4	4	4	4	Televisi, Internet	Dibuang setelah	5	5	5	5	5	Ya	Ya	Tidak pernah	Mengurangi pen	25	Tidak	Ya	Ya
>3x sehari	44	5	3	5	5	5	5	Lebel pada botol	Digunakan untuk	5	3	4	4	3	Tidak tahu	Ya	Jarang (<50%)	Mengurangi pen	19	Tidak	Ya	Ya
>3x sehari	56	5	5	5	5	5	5	Televisi, Koran, I	Digunakan untuk	5	5	5	5	5	Tidak tahu	Ya	Terkadang (>50%	Mengurangi pen	25	Ya	Tidak	Ya
Tidak pernah	20	4	4	4	4	4	4	Televisi	Dibuang setelah	4	4	4	4	4	Ya	Ya	Jarang (<50%)	Tetap mengguna	20	Tidak	Ya	Ya
1-3x sepekan	40	5	4	5	5	5	5	Televisi, Internet	Digunakan untuk	5	5	5	5	5	Ya	Ya	Terkadang (>50%	Menghentikan ko	25	Ya	Ya	Ya
>3x sehari	44	5	5	5	5	3	3	Buku/Jurnal, Kor	Dibuang setelah	5	5	5	5	5	Ya	Ya	Terkadang (>50%	Menghentikan ko	25	Tidak	Ya	Ya
Tidak pernah	22	4	5	5	4	5	4	Televisi, Internet	Diisi ulang	4	5	4	4	3	Ya	Ya	Jarang (<50%)	Mengurangi pen	20	Tidak	Ya	Ya
Tidak pernah	36	4	4	4	4	3	3	Buku/Jurnal, Inte	Dibuang setelah	5	5	5	4	5	Ya	Ya	Jarang (<50%)	Menghentikan ko	24	Tidak	Ya	Ya
1-3x sebulan	27	3	3	3	4	5	3	Televisi, Internet	Dibuang setelah	5	5	5	5	5	Ya	Ya	Tidak pernah	Mengurangi pen	25	Tidak	Ya	Tidak
Tidak pernah	22	3	5	5	5	3	4	Internet	Dibuang setelah	4	4	5	5	2	Ya	Ya	Jarang (<50%)	Mengurangi pen	20	Tidak	Ya	Ya
1-3x sehari	49	3	3	3	3	3	3	Televisi, Buku/Ju	Dibuang setelah	3	3	3	3	3	Ya	Ya	Terkadang (>50%	Mengurangi pen	15	Ya	Ya	Ya
Tidak pernah	28	4	4	4	4	4	4	Televisi, Internet	Dibuang setelah	5	5	5	5	5	Ya	Ya	Jarang (<50%)	Menghentikan ko	25	Ya	Ya	Ya
Tidak pernah	8	5	4	4	4	4	4	Televisi, Internet	Dibuang setelah	4	4	4	4	4	Ya	Ya	Tidak pernah	Mengurangi pen	20	Ya	Ya	Ya
>3x sehari	56	4	4	4	4	4	4	Lebel pada botol	Dibuang setelah	4	4	4	4	4	Tidak tahu	Tidak tahu	Jarang (<50%)	Mengurangi pen	20	Tidak	Tidak	Tidak
1-3x sepekan	39	4	4	5	5	5	4	Internet	Dibuang setelah	5	5	5	5	5	Ya	Ya	Tidak pernah	Tetap mengguna	25	Tidak	Ya	Ya
Tidak pernah	34	4	5	5	5	4	3	Internet	Dibuang setelah	4	3	3	3	2	Ya	Ya	Jarang (<50%)	Mengurangi pen	15	Ya	Ya	Ya
>3x sehari	56	4	4	4	4	4	4	Televisi, Internet	Diisi ulang	5	5	5	5	5	Ya	Ya	Jarang (<50%)	Mengurangi pen	25	Tidak	Ya	Ya
1-3x sehari	40	4	4	4	4	4	4	Internet	Dibuang setelah	4	4	3	4	4	Ya	Ya	Tidak pernah	Mengurangi pen	19	Ya	Ya	Ya
Tidak pernah	28	5	4	5	5	3	4	Televisi	Dibuang setelah	5	5	5	3	4	Ya	Ya	Terkadang (>50%	Mengurangi pen	22	Tidak	Ya	Ya
<1x sebulan	16	3	4	5	4	4	4	Internet, Lainnya	Dibuang setelah	5	5	5	5	5	Ya	Ya	Terkadang (>50%	Menghentikan ko	25	Ya	Ya	Ya

Jenis plastik se	Menghentikan p	Mendorong ind	Menerapkan cu	Menerapkan sta	Menerapkan sta	Meningkatkan t	Industri perlu tr	Memperbaiki si	skor sikap kebi	1. Apakah Anda	2. Apakah Anda	3. Apakah Anda	4. Apakah Anda	5. Apakah Anda	6. Apakah Anda	7. Apakah perut	8. Apakah Anda	9. Apakah Anda	10. Apakah And	11. Apakah And	12. Saat Anda n	13. Apakah And
Kantong Plastik,	5	5	5	5	5	3	5	5	38													
Kantong Plastik,	5	5	5	5	5	5	5	5	40													
Sterofoam	4	4	5	5	5	5	5	5	38	1	2	2	2	1	1	1	1	2	1	1	1	1
Kantong plastik	5	5	1	5	5	5	5	5	36	1	1	1	1	1	1	1	1	1	1	1	1	1
Kantong plastik,	5	5	5	5	5	5	5	5	40	1	1	3	1	1	1	1	1	1	1	1	1	1
Kantong plastik,	5	5	5	5	5	5	5	5	40	1	2	1	1	1	1	1	1	1	1	1	1	1
Kantong plastik	4	5	5	5	5	5	5	5	39	3	1	1	1	1	1	1	1	1	1	1	1	1
Kantong plastik,	5	5	5	5	5	5	5	5	40	4	4	6	6	3	3	5	6	3	1	5	6	1
Kantong Plastik,	4	5	4	5	5	5	5	5	38													
Kantong plastik,	5	5	5	5	5	5	5	5	40	3	1	1	1	1	2	5	2	3	1	1	1	1
Kantong plastik	5	5	4	4	5	4	5	5	37	1	1	1	2	1	1	1	1	1	1	1	1	1
Kantong plastik,	3	3	3	3	3	3	3	3	24	1	1	1	4	1	1	1	1	1	1	1	1	1
Kantong plastik,	3	5	2	4	4	5	5	5	33	1	1	1	1	1	1	1	1	3	2	1	1	2
Kantong Plastik,	5	5	5	5	5	5	5	5	40													
Kantong plastik,	4	4	4	4	4	4	4	4	32	6	5	5	6	1	1	1	1	1	1	1	1	1
Kantong plastik,	4	4	4	4	4	4	4	4	32	2	2	2	2	2	2	2	2	2	2	2	2	2
Kantong Plastik,	5	5	5	5	5	5	5	5	40													
Kantong Plastik,	3	3	4	5	5	5	5	5	35													
Kantong plastik,	3	4	3	5	5	5	5	5	35	3	3	4	4	4	3	5	5	5	4	1	1	4
Kantong plastik,	5	5	5	5	5	5	5	5	40	1	1	1	1	1	1	1	1	1	1	1	1	1
Sterofoam	4	4	4	4	4	4	4	4	32	5	6	4	4	4	4	4	5	5	5	5	4	5
Kantong plastik,	4	4	3	4	4	5	5	5	34													
Kantong plastik,	3	5	3	5	5	5	5	5	36	1	1	1	3	1	1	1	1	1	1	1	1	1
Kantong plastik,	5	5	5	5	5	5	5	5	40	1	1	1	1	1	1	1	1	1	1	2	1	1
Kantong plastik,	4	5	3	5	5	5	4	5	36	1	1	5	5	5	1	1	1	1	1	5	5	6
Sterofoam, Boto	5	2	2	4	4	2	5	5	29	1	1	1	1	1	4	1	1	1	1	5	4	1
Kantong plastik	5	5	5	5	5	5	5	5	40	1	7	3	7	1	7	7	7	7	2	2	1	5
Kantong plastik	5	4	3	4	4	5	5	5	35	1	1	1	2	1	1	5	5	2	3	2	2	2
Kantong plastik,	5	5	5	5	5	5	5	5	40													
Kantong Plastik,	4	5	5	5	5	5	5	5	39													
Kantong plastik,	4	4	4	4	4	4	5	5	34	1	1	1	1	1	1	1	1	1	1	1	1	1
Kantong plastik,	4	4	3	4	5	4	5	5	34	4	1	2	5	2	3	4	2	4	2	1	1	3
Kantong Plastik,	3	4	4	4	4	4	4	5	32													
Kantong Plastik,	4	4	4	4	4	4	4	4	32													
Kantong Plastik,	5	5	5	5	5	5	5	5	40													
Kantong plastik,	5	5	4	5	5	5	5	5	39	1	1	1	2	1	2	1	1	1	2	1	1	1
Sterofoam, Kem	5	5	5	5	5	5	5	5	40	1	1	1	1	1	1	1	2	1	1	1	1	2
Sterofoam, Kem	4	4	4	4	4	4	4	4	32	1	1	1	1	1	1	2	1	1	1	1	1	1
Kantong plastik,	4	5	5	3	3	2	5	1	28	1	1	1	1	1	1	1	1	1	4	1	1	4
Kantong plastik,	4	5	5	5	5	5	5	5	39	4	2	3	3	1	3	4	3	2	2	1	1	1
Kantong plastik,	4	5	5	5	5	5	5	5	39	4	4	1	7	1	7	7	7	7	7	7	5	2
Kantong Plastik,	5	5	5	5	5	5	5	5	40													
Kantong plastik,	5	5	5	5	5	5	5	5	40	5	6	6	4	3	3	5	6	6	6	1	1	5
Sterofoam, Kem	5	5	5	5	5	5	5	5	40	1	1	2	1	1	1	1	1	1	1	1	1	1
Kantong plastik	5	5	5	5	5	5	5	5	40	1	1	1	1	1	1	1	1	1	1	1	1	1
Kantong plastik,	5	5	5	5	5	5	5	5	40	4	5	5	5	3	5	5	5	3	3	3	3	3
Tidak tahu	1	5	5	3	5	5	5	5	34	1	1	1	7	1	7	5	1	7	1	1	1	1
Kantong Plastik,	5	5	3	4	4	4	4	4	33													
Kantong Plastik,	5	5	5	5	5	5	5	5	40													
Kantong plastik	5	5	1	5	5	5	5	5	36	3	1	4	3	1	2	2	5	5	3	1	3	2
Kantong plastik,	5	5	1	5	5	5	5	5	36	3	1	3	1	1	1	2	2	2	1	1	1	1
Kantong plastik	5	5	5	5	5	4	5	5	39	1	1	1	1	1	1	1	1	1	1	1	1	1
Kantong plastik,	3	4	4	5	5	5	4	5	35	2	2	1	2	3	4	3	2	3	1	1	1	3
Kantong Plastik,	5	5	4	5	4	5	5	5	38													
Kantong Plastik,	5	5	5	5	5	5	5	5	40													
Kantong plastik,	3	4	5	5	5	5	4	5	36													
Tidak tahu	4	5	4	5	5	5	5	5	38	2	5	6	2	4	3	1	4	1	3	1	1	1
Kantong plastik,	4	4	4	4	4	4	4	4	32	3	2	3	3	3	3	3	3	3	3	3	3	3
Kantong plastik,	2	4	2	3	4	4	4	4	27	1	1	1	5	1	1	1	1	1	1	6	1	1
Sterofoam	5	5	5	5	5	5	5	5	40	1	1	1	1	1	1	1	1	1	1	1	1	1
Kantong plastik,	5	5	5	5	5	5	5	5	40	2	1	1	1	5	1	1	2	1	1	1	1	1
Kantong Plastik,	4	4	4	4	4	4	4	4	32													
Kantong Plastik,	4	5	5	5	5	5	5	5	39													
Kantong Plastik,	5	5	5	5	5	5	5	5	40													
Kantong Plastik,	3	4	4	4	4	4	4	4	31													
Kantong Plastik,	5	5	5	5	5	5	5	5	40													
Kantong plastik,	4	5	3	4	4	5	5	5	35													
Tidak tahu	5	4	5	5	5	5	5	5	39	3	4	3	4	3	3	3	3	3	5	3	3	6
Sterofoam, Kem	4	4	5	5	4	5	5	4	36	1	1	1	4	1	4	1	1	7	1	1	1	4
Kantong plastik	5	5	4	4	4	4	3	5	34	1	1	1	2	1	1	1	1	5	1	1	1	1
Kantong plastik,	5	5	5	5	5	5	5	5	40	1	1	1	1	1	1	1	1	1	1	1	1	1
Kantong Plastik,	5	5	5	5	5	5	5	5	40													
Kantong plastik,	4	5	5	5	5	5	5	5	39	1	1	3	1	1	3	5	4	5	6	1	1	5
Sterofoam	5	5	5	5	5	5	5	5	40	1	1	1	1	1	1	1	1	1	2	1	1	2
Kantong plastik,	5	5	4	5	5	5	5	5	39	3	2	2	3	2	1	3	3	3	1	1	1	3
Kantong plastik,	5	4	5	5	5	5	5	4	38													
Sterofoam, Tidak	4	4	4	4	4	5	5	5	35	1	1	1	2	1	1	1	1	1	1	1	1	1
Kantong Plastik,	4	4	4	4	4	4	5	5	34													
Kemasan plastik	4	3	3	2	3	2	3	3	23	4	4	3	5	2	4	4	4	3	4	3	5	3
Sterofoam, Kem	5	5	5	5	5	5	5	5	40	1	1	3	1	2	1	2	2	2	1	1	1	1
Kantong Plastik,	5	5	5	5	5	5	5	5	40													
Sterofoam, botol	3	3	3	4	4	5	5	5	32													
Kantong Plastik,	5	5	5	5	5	2	3	2	32													
Kantong Plastik,	5	5	5	5	5	5	5	5	40													
Kantong plastik,	5	5	3	5	5	5	5	5	38													
Kantong plastik,	5	5	5	5	5	5	5	5	40	1	1	2	2	1	2	2	2	1	1	1	1	1
Kantong plastik,	5	5	5	5	5	5	4	5	39	2	1	3	5	1	1	3	1	1	1	1	1	1
Kantong plastik,	5	4	4	5	5	5	5	5	38	1	1	1	2	1	1	1	1	1	1	1	1	1
Kantong plastik,	4	5	4	4	4	4	5	4	34	4	6	6	6	6	6	6	5	6	6	5	6	5
Sterofoam, Kem	4	4	2	4	4	4	4	4	30	2	2	2	2	2	2	2	2	2	2	2	2	2
Kantong plastik,	5	5	5	5	5	5	5	5	40	1	1	1	3	1	1	2	1	2	2	1	1	2
Kantong plastik	5	5	5	5	4	4	4	4	36	1	1	1	1	1	1	1	1	1	1	1	1	2
Kantong plastik,	5	3	4	4	5	4	4	5	34	1	1	1	1	1	1	2	1	1	3	1	1	3
Kantong plastik,	4	4	4	5	5	5	5	4	36	1	1	1	1	1	1	1	1	1	1	1	1	1
Kantong plastik,	5	5	5	5	5	5	5	5	40	4	1	3	1	1	1	3	3	3	1	3	4	1
Kantong plastik,	5	5	5	5	5	5	5	5	40	1	1	1	1	1	1	1	1	1	1	1	1	1
Kantong plastik,	4	5	4	5	5	5	5	5	38	3	1	4	4	2	4	3	3	2	2	1	1	1
Kantong Plastik	5	5	5	5	5	5	5	5	40													
Kantong plastik,	2	2	3	1	1	1	2	2	14	3	3	2	3	5	5	3	3	5	3	4	6	2
Kantong plastik,	4	5	4	5	5	5	5	5	38	2	1	1	4	1	1	3	3	2	1	2	2	2
Sterofoam	3	5	5	5	5	5	5	5	38	3	3	5	3	1	2	3	1	1	5	1	1	3
Kantong plastik,	5	5	5	5	5	5	5	5	40	1	1	1	1	1	1	1	1	1	1	1	1	1
Kantong plastik	4	4	4	5	5	5	5	5	37	1	1	4	5	4	4	5	6	4	4	4	4	5
Tidak tahu	5	5	5	5	5	5	5	5	40													
Kantong plastik	5	5	5	5	5	5	5	5	40	2	3	2	2	2	2	3	1	1	3	3	4	1
Kantong plastik,	5	5	5	5	5	5	5	5	40	7	7	7	7	7	7	7	7	7	7	7	7	7
Kantong plastik,	5	5	5	5	5	5	5	5	40	1	1	1	1	1	1	1	1	1	1	1	1	1
Kantong plastik,	5	5	3	5	5	5	5	5	38	7	7	7	7	1	3	7	4	4	4	1	2	1
Kantong Plastik,	5	5	5	5	5	5	5	5	40													
Kantong Plastik	4	4	4	4	4	4	4	4	32													
Kantong Plastik,	5	5	5	1	5	5	5	5	36													
Tidak tahu	5	4	2	4	4	5	5	5	34													
Kantong Plastik,	4	5	2	5	5	4	5	5	35													
Kantong plastik,	4	4	4	4	4	4	4	5	33													
Sterofoam, Kem	5	5	5	5	5	5	5	5	40	2	2	2	2	3	1	2	4	3	1	1	3	2
Sterofoam, Kem	4	4	4	4	4	4	4	4	32	4	2	2	5	1	3	1	1	1	1	3	1	1
Sterofoam	4	5	5	5	5	5	5	5	39	3	3	2	4	2	2	2	2	2	2	2	2	2
Sterofoam	3	3	3	3	3	3	3	3	24	4	4	4	4	3	3	3	2	4	1	1	1	1
Kantong plastik,	4	4	3	4	4	4	4	4	31	2	1	4	4	4	2	3	1	1	1	1	1	1
Kantong plastik	3	5	4	5	5	5	5	5	37	1	1	1	1	1	1	1	1	1	1	1	1	1
Kantong plastik,	3	4	5	4	5	5	5	5	36	1	3	5	4	3	4	5	5	5	2	1	5	5
Kantong plastik,	5	5	5	5	5	5	5	5	40	5	1	3	6	1	6	2	5	5	2	4	2	3
Kantong plastik,	4	5	5	5	5	5	5	4	38	2	5	5	3	3	2	2	5	3	2	1	1	1
Kantong plastik,	4	5	4	5	4	4	5	5	36	2	1	5	5	1	6	6	2	5	6	2	2	7
Kantong plastik	4	5	4	5	5	5	5	5	38	1	1	1	5	1	1	1	1	3	1	1	1	1
Kantong plastik,	5	5	5	5	5	5	5	5	40	1	1	1	1	1	1	1	1	1	1	1	1	1
Kantong Plastik	5	5	5	5	5	5	5	5	40													
Kantong Plastik,	5	5	5	5	5	5	5	5	40													
Kantong plastik,	5	5	4	4	5	5	5	5	38	6	6	6	6	5	5	5	5	5	2	2	2	5
Kantong plastik	5	5	5	5	5	5	5	5	40	5	7	7	5	5	5	6	5	6	5	3	3	4
Kantong plastik,	5	5	5	5	5	5	5	5	40	2	2	3	2	1	1	1	1	1	2	1	2	1
Kantong plastik,	3	2	5	5	5	5	5	5	35	5	2	2	1	3	1	5	2	5	5	6	4	4
Kantong plastik	5	5	5	5	5	5	5	5	40	5	5	5	6	3	3	1	1	1	1	1	1	1
Kantong plastik,	4	5	5	5	5	5	5	5	39	5	6	5	6	6	6	7	7	5	6	1	1	5
Kantong plastik,	5	5	5	5	5	5	5	5	40	1	1	1	1	1	1	1	1	1	1	2	2	1
Kantong plastik,	4	5	4	3	4	5	4	5	34	2	1	1	5	1	4	2	3	6	2	2	1	1
Kantong plastik,	5	5	5	5	5	5	5	5	40	4	1	1	1	1	1	1	1	1	1	6	6	1
Tidak tahu	5	5	5	5	5	5	5	5	40													
Kantong Plastik	5	5	4	5	5	5	5	5	39													
Kantong plastik,	5	5	5	5	5	5	5	5	40	1	1	1	3	1	5	5	5	5	6	1	1	7
Kantong plastik	5	5	5	5	5	5	5	5	40	1	1	1	3	1	1	2	1	1	1	1	1	1
Kantong Plastik,	5	5	5	5	5	5	5	5	40													
Kantong Plastik	5	5	5	5	5	5	5	5	40													
Kantong Plastik,	4	5	4	5	4	5	5	5	37													
Kantong plastik,	5	5	5	5	5	5	5	5	40	1	1	1	3	1	3	1	1	1	3	1	3	3
Kantong Plastik	5	5	5	5	5	5	5	5	40													
Kantong plastik,	1	4	1	5	5	5	5	5	31	1	2	2	4	1	2	4	4	4	4	1	1	5
Kantong plastik	4	4	4	4	4	4	4	4	32	4	4	4	4	4	4	4	4	4	4	4	4	4
Kantong plastik	5	5	5	5	5	5	5	5	40	6	6	6	7	1	7	6	1	6	1	7	7	1
Kantong plastik	3	4	1	5	5	5	5	5	33	5	5	5	5	1	1	1	1	5	1	1	1	1
Sterofoam	5	5	5	5	5	5	5	5	40	4	3	1	1	3	1	2	1	3	2	3	5	1
Kantong plastik,	5	5	5	5	5	3	2	4	34	3	1	1	1	1	1	1	1	2	1	1	1	1
Kantong Plastik,	4	4	4	4	4	5	5	5	35													
Kantong plastik,	3	3	3	4	4	4	4	4	29	1	2	2	3	2	2	1	1	3	3	1	1	2
Kantong plastik,	4	4	4	4	4	4	4	4	32	1	1	2	2	1	1	1	1	1	1	1	1	1
Sterofoam	5	3	4	5	4	5	4	4	34	1	3	6	6	5	4	4	6	6	5	1	2	4
Kantong plastik,	3	5	4	5	5	5	5	5	37	1	1	1	1	1	1	1	1	1	1	1	1	1
Kantong plastik,	4	4	4	4	4	4	4	4	32	1	2	2	4	2	2	4	4	2	5	1	1	4
Kantong plastik,	4	5	4	4	4	4	5	5	35	2	2	2	4	1	1	1	1	1	2	1	1	1
Kantong plastik,	3	4	2	3	4	5	5	4	30	4	5	3	3	4	2	2	2	3	5	4	3	5
Kantong plastik	2	4	2	4	4	4	5	5	30	2	2	2	2	2	2	2	2	2	2	2	2	2
Kantong plastik,	5	5	5	5	5	5	5	5	40	1	1	1	4	1	1	4	6	1	1	1	1	1
Kantong plastik,	5	5	5	5	5	5	5	5	40	5	5	5	2	1	2	3	5	4	4	1	1	4
Kantong plastik,	5	5	5	5	5	5	5	5	40	1	1	2	2	1	2	1	1	2	1	1	1	1
Kemasan plastik	1	4	1	5	5	5	5	5	31	1	1	1	1	1	3	1	1	3	1	1	1	4
Kantong Plastik,	4	5	5	4	4	5	5	4	36													
Kantong Plastik,	4	3	3	4	3	3	4	5	29													
Kantong Plastik,	1	5	1	2	2	5	5	5	26													
Kemasan Plastik	5	5	5	5	5	5	5	5	40													
Kantong Plastik,	3	5	1	5	5	5	5	5	34													
Kantong plastik,	5	5	4	5	5	5	5	5	39	1	1	1	1	1	1	1	1	1	1	1	1	1
Kemasan plastik	2	1	2	2	3	3	5	1	19	1	1	1	1	1	1	1	1	1	1	1	3	1
Kantong plastik,	5	5	2	5	5	5	5	5	37	1	1	2	1	1	1	2	1	1	1	1	1	1
Kantong plastik	5	5	4	5	5	5	5	5	39	3	3	7	5	3	3	5	5	4	3	1	1	1
Kantong plastik,	5	1	5	5	5	5	5	5	36	1	1	1	1	1	4	4	4	4	6	1	1	5
Kantong plastik	5	5	5	5	5	5	5	4	39	3	3	6	7	5	7	7	3	3	1	1	1	1
Kantong plastik,	3	3	3	5	5	5	5	5	34	1	1	1	6	1	4	1	1	1	4	1	1	1
Tidak tahu	5	5	5	5	5	5	5	5	40	1	1	1	1	1	1	1	1	1	1	1	1	1
Kantong plastik	4	4	4	4	4	5	5	5	35	3	3	4	5	3	3	5	5	3	2	2	2	2
Kantong plastik,	3	4	2	4	5	5	5	5	33	6	6	7	6	7	1	4	5	1	1	7	5	1
Tidak tahu	5	5	5	5	5	5	5	5	40	1	1	3	3	2	1	1	2	1	4	1	1	1
Kantong plastik	3	5	3	5	5	5	5	3	34	1	1	1	1	1	1	1	1	2	1	1	1	1
Kantong plastik,	5	5	5	5	5	5	5	5	40	1	1	1	5	1	6	6	7	1	6	1	1	6
Kantong plastik,	4	4	5	4	4	5	5	4	35	1	1	1	4	1	2	1	1	5	1	4	2	2
Tidak tahu	3	5	4	4	4	5	5	5	35	1	1	1	1	1	1	1	1	2	2	1	2	2
Kantong Plastik,	4	5	4	5	5	5	5	5	38													
Sterofoam	5	4	2	4	5	5	4	4	33													
Tidak tahu	3	4	4	4	4	4	5	5	33	3	3	5	4	2	5	5	3	3	3	2	3	1
Kantong plastik	5	5	5	5	5	5	5	5	40	1	1	1	1	1	1	1	1	1	1	1	1	1
Kantong plastik	3	4	4	5	5	5	5	5	36	2	1	2	3	1	2	3	4	4	2	1	2	4
Sterofoam	3	4	3	5	5	5	5	4	34	5	1	5	3	5	1	6	5	6	1	5	5	1

Kantong plastik,	5	4	3	2	1	4	4	4	27	7	5	6	6	6	6	4	5	3	4	6	7	5
Kantong plastik	5	5	5	5	5	5	5	5	40	1	1	1	1	1	1	1	1	1	1	1	1	1
Kantong plastik,	5	5	3	5	5	5	5	5	38	1	1	1	1	1	1	1	1	1	1	1	1	1
Kantong plastik,	5	5	5	5	5	5	5	5	40	1	1	1	1	2	1	1	1	1	1	3	2	1
Kantong Plastik,	4	4	3	3	3	3	3	3	26													
Kantong plastik,	5	5	5	5	5	5	5	5	40	6	7	6	7	7	7	4	4	7	7	2	3	7
Kantong plastik,	5	5	5	5	5	5	5	5	40	1	1	5	1	3	3	5	6	4	1	5	6	1
Kantong plastik,	3	3	3	3	3	3	3	3	24	1	5	6	6	6	6	6	6	6	6	1	1	2
Kantong plastik,	4	4	4	4	4	4	4	4	32	1	1	1	3	1	2	1	1	1	1	1	1	1
Kantong plastik,	4	4	4	4	5	4	4	4	33	4	3	5	4	2	4	5	3	4	3	2	3	3
Kantong plastik,	3	3	3	3	3	3	3	3	24	4	4	4	4	4	4	4	4	4	4	4	4	4
Kantong plastik,	4	4	4	5	5	5	5	5	37	1	1	1	1	1	1	1	1	1	1	1	1	1
Kantong plastik,	5	4	3	5	5	5	5	5	37	1	1	1	1	1	1	1	1	1	1	3	1	1
Kantong plastik	5	5	5	5	5	5	5	5	40	1	1	1	3	4	4	1	1	1	1	5	5	1
Kantong plastik,	4	5	5	5	5	4	5	5	38	3	3	4	5	5	4	3	3	6	6	4	3	4
Tidak tahu	3	3	3	3	3	3	3	3	24	1	1	1	1	1	1	1	1	1	1	1	1	1
Kantong plastik,	2	1	5	4	4	4	4	5	29	1	2	1	1	1	1	3	1	2	1	1	1	1
Kantong plastik,	5	5	5	5	5	5	5	5	40	1	1	1	5	1	6	5	4	6	1	1	2	1
Sterofoam, kema	5	5	5	5	5	5	5	5	40													
Kantong Plastik,	4	4	3	3	3	3	3	3	26													
Kantong plastik,	5	5	5	5	5	5	5	5	40	3	4	5	4	6	4	6	3	6	4	6	6	7
Kantong plastik	4	4	4	4	4	4	4	4	32	5	5	6	6	6	6	6	6	5	4	4	4	4
Tidak tahu	4	5	3	3	4	4	4	5	32	5	1	2	3	6	6	6	7	5	1	6	6	1
Kantong plastik,	4	4	5	5	5	5	5	5	38	2	2	1	4	1	4	3	3	3	2	3	3	2
Kemasan plastik	4	4	4	4	4	4	4	4	32	1	1	2	2	1	1	2	1	1	1	1	1	1
Sterofoam	3	5	3	4	4	4	4	4	31	1	1	1	1	1	3	3	1	3	1	1	1	1
Kantong plastik,	3	4	3	4	4	4	4	5	31	5	5	5	4	4	2	3	4	3	2	4	4	1
Sterofoam, Tidak	3	5	3	5	5	5	5	5	36	1	1	1	1	1	1	1	1	1	1	1	1	1
Kantong plastik,	4	5	5	5	5	5	5	5	39	3	1	3	3	2	4	4	6	6	4	2	2	4
Kantong plastik,	5	5	5	5	5	5	5	5	40	1	1	1	1	1	1	1	1	1	1	1	1	1
Kantong Plastik,	4	5	5	5	5	5	5	5	39													
Kantong Plastik,	5	5	5	5	5	4	5	5	39													
Sterofoam	4	4	3	4	4	4	3	4	30													
Sterofoam	3	5	3	4	4	3	5	5	32	1	1	1	1	1	1	2	2	1	1	1	1	1
Kantong plastik,	5	5	5	5	5	5	5	5	40	2	2	2	2	2	2	1	4	1	1	1	1	1
Sterofoam, Kem	5	5	5	5	5	5	5	5	40	4	4	5	6	3	3	3	4	4	4	6	3	3
Kantong plastik,	5	4	4	4	5	4	5	5	36	1	1	1	2	2	2	2	2	2	2	2	2	1
Sterofoam	5	5	5	5	5	5	5	5	40	1	1	2	3	1	3	2	2	2	1	1	2	1
Kantong plastik,	3	5	5	5	5	5	4	5	37	3	4	5	2	1	5	4	6	3	3	1	1	3
Sterofoam	3	3	4	4	4	4	5	5	32	1	1	1	3	1	5	1	2	1	5	1	3	4
Kantong plastik	5	5	5	5	5	5	5	5	40	4	4	4	4	4	4	4	4	4	4	4	4	4
Kantong plastik	3	5	4	5	5	5	5	5	37	1	1	1	5	1	3	2	1	1	1	1	1	1
Kantong plastik,	5	5	5	5	5	5	5	5	40	7	7	7	7	7	7	7	7	7	7	7	7	7
Kantong plastik,	4	5	4	4	5	5	5	4	36	2	1	1	4	1	2	1	3	3	3	3	4	2
Kantong plastik,	3	3	2	2	3	3	3	3	22	4	3	4	4	3	3	3	4	4	4	2	2	3
Kantong Plastik,	4	5	3	4	4	5	5	4	34													
Sterofoam	3	4	3	4	4	4	4	4	30													
Kantong Plastik	1	2	1	4	4	5	3	5	25													
Kantong Plastik,	5	5	2	5	5	5	5	5	37													
Kantong plastik,	4	4	4	4	4	4	4	4	32	1	1	1	5	1	1	1	1	1	1	1	1	1
Sterofoam	3	3	3	3	3	3	3	3	24	3	4	3	3	3	3	3	3	3	3	3	3	3
Kantong plastik,	3	4	2	4	4	5	5	5	32	1	1	1	3	2	1	1	1	1	1	1	1	1
Kantong plastik	5	5	5	5	5	5	5	5	40	1	1	5	5	4	5	3	1	1	1	1	1	1
Kantong plastik,	5	5	5	5	5	5	5	5	40	7	7	7	7	3	2	7	3	4	7	4	5	5
Kantong plastik,	4	4	4	4	4	4	4	4	32	1	1	1	1	1	1	1	1	1	1	1	1	1
Kantong plastik,	5	4	4	4	5	4	4	5	35	3	3	4	5	3	5	4	4	3	4	2	2	3
Sterofoam	3	4	5	5	5	5	5	5	37	1	1	1	3	1	1	1	1	4	1	1	1	2
Kantong plastik,	4	4	4	5	5	5	5	5	37	4	4	4	6	1	6	6	3	2	2	2	2	2
Kantong plastik,	5	5	5	5	5	5	5	5	40	1	1	1	3	1	5	2	2	1	3	1	1	3
Kantong plastik,	3	2	3	5	5	5	5	5	33	3	3	3	3	3	1	2	1	1	1	1	3	1
Kantong plastik,	5	3	5	5	5	5	5	5	38	1	1	1	3	1	1	1	1	3	1	1	1	1
Kantong plastik,	4	5	4	5	5	5	5	5	38	1	1	1	1	1	1	1	1	1	1	1	1	1
Kantong plastik	4	4	3	3	3	3	3	3	26	1	1	1	1	1	1	1	1	1	1	1	1	1
Sterofoam, Boto	4	4	4	4	4	4	4	4	32	5	2	2	4	2	2	2	2	2	2	2	2	2
Kantong plastik	5	5	5	5	5	5	5	5	40	1	1	1	1	1	1	1	1	1	1	1	1	1
Tidak tahu	5	5	5	5	5	5	5	5	40	7	7	7	7	7	7	7	7	7	7	7	7	7
Kantong plastik	3	2	2	2	2	2	2	2	17	3	3	3	3	3	3	3	3	3	3	3	3	3
Kantong plastik,	4	4	4	4	4	4	4	4	32	1	3	3	3	1	1	1	1	1	1	1	1	2
Kantong plastik,	4	5	5	5	4	5	5	5	38	2	1	2	6	1	4	5	1	1	1	3	3	1
Kantong plastik,	5	5	5	5	5	4	4	5	38	5	6	1	6	7	6	7	6	4	7	5	5	4
Kantong plastik,	5	5	5	5	5	5	5	5	40	7	2	2	5	1	3	3	2	2	1	5	6	1
Kantong plastik,	4	5	5	5	5	5	5	5	39	2	1	1	1	2	1	1	2	1	1	1	1	1
Kantong plastik	5	5	4	5	5	5	5	5	39	4	1	4	4	4	4	4	4	2	1	1	1	1
Kantong plastik,	4	5	5	5	5	5	5	5	39	6	5	5	3	1	2	3	1	3	1	5	5	3
Kantong plastik,	4	5	4	4	4	4	4	5	34	1	1	1	3	1	2	3	1	2	1	1	1	2
Kantong plastik	4	4	4	4	4	4	5	5	34	4	6	6	3	5	4	5	5	5	3	1	1	2
Kantong plastik,	5	5	5	5	5	5	5	5	40	3	4	1	3	1	5	1	1	4	1	1	6	5
Kantong plastik,	4	4	4	4	4	5	4	4	33	4	5	5	5	5	6	6	4	6	6	6	4	6
Kantong plastik	5	5	5	5	5	5	5	5	40	1	1	1	1	1	1	1	1	1	1	4	4	1
Kantong plastik,	5	4	5	5	5	5	5	5	39	5	5	6	6	5	5	2	4	3	2	2	2	1
Kantong plastik,	5	5	5	5	5	5	5	5	40	1	1	1	3	1	1	1	3	3	5	2	3	5
Kantong plastik	5	5	5	5	5	5	5	5	40	4	1	6	7	7	1	7	1	1	7	1	1	7
Kantong plastik,	4	4	3	4	4	4	4	4	31	1	1	1	5	3	4	4	1	1	1	1	1	1
Kantong plastik,	5	5	5	5	5	5	5	4	39	5	5	7	6	5	5	5	5	5	2	1	4	4
Kantong plastik,	5	5	5	5	5	5	5	5	40	4	1	1	1	1	1	4	3	3	3	1	1	1
Tidak tahu	5	5	5	5	5	5	5	5	40	4	3	4	5	1	2	4	6	1	1	2	1	1
Kantong plastik,	5	5	5	5	5	5	5	5	40	3	1	2	2	2	2	4	2	2	2	2	3	1
Sterofoam	4	5	3	4	4	5	5	5	35	1	1	1	1	1	1	1	1	1	1	1	1	1
Tidak tahu	4	4	1	5	5	5	5	5	34													
Kantong Plastik	5	5	5	5	5	5	5	5	40													
Kantong plastik,	5	5	5	5	5	5	5	5	40													
Kantong plastik	5	5	3	5	5	5	5	5	38	2	1	2	3	1	1	2	1	2	1	1	1	1
Kantong plastik	5	5	5	5	5	5	5	5	40	2	1	3	1	1	3	2	3	4	2	1	1	1
Kantong plastik,	3	5	4	5	5	5	5	5	80	2	2	5	7	5	5	5	3	3	3	3	1	2
Sterofoam	5	4	5	5	5	5	5	5	39	1	4	1	1	1	5	4	5	3	5	1	1	4
Kantong plastik,	5	5	5	5	5	5	5	5	40	4	2	4	5	1	2	1	1	1	1	1	1	1
Kantong plastik,	4	4	4	4	4	4	4	4	32	2	2	2	3	1	1	1	1	1	1	1	1	1
Kantong plastik,	4	4	4	4	4	4	4	4	32	1	1	1	1	1	1	1	1	1	1	1	1	1
Tidak tahu	2	5	4	5	5	5	5	5	36	6	6	6	2	5	1	4	6	6	1	6	6	1
Kantong plastik,	4	3	4	4	3	5	5	5	33	1	1	1	3	1	2	2	2	2	1	1	1	1
Kantong plastik,	5	5	4	5	5	5	5	5	39	2	2	1	5	2	2	2	2	2	2	2	2	2
Kantong plastik,	4	5	5	5	5	5	5	5	39	1	1	4	2	4	3	4	5	3	1	1	1	1
Kantong plastik	4	4	4	4	4	4	4	4	32	3	3	3	4	3	3	3	2	4	3	3	2	4
Kantong plastik,	5	5	5	5	5	5	5	5	40	1	4	4	4	1	1	4	1	3	1	1	1	1
Kantong plastik,	4	5	5	4	4	4	5	4	35	1	2	3	2	1	2	2	3	3	4	1	1	2
Kantong plastik,	5	5	5	5	5	5	5	5	40	1	1	1	5	1	5	1	1	1	1	1	1	2
Tidak tahu	5	5	5	5	5	5	5	5	40	1	1	1	1	1	1	1	1	1	1	1	1	1
Kantong plastik	5	5	5	5	5	5	5	5	40	2	2	3	3	1	4	2	3	1	2	2	2	1
Sterofoam, Kem	5	5	5	5	5	5	5	5	40	4	1	4	1	1	1	1	2	1	1	1	1	1
Kantong plastik,	2	4	1	4	4	5	4	4	28	5	1	5	1	1	1	7	1	1	1	1	1	4
Kantong plastik	5	5	5	5	5	5	5	5	40	1	1	4	4	4	1	1	1	1	1	1	1	1
Kantong Plastik,	5	5	5	5	5	5	5	5	40													
Kantong Plastik,	5	5	1	5	5	5	5	5	36													
Kantong Plastik,	5	5	5	5	5	5	5	4	39													
Kantong Plastik,	5	5	4	5	5	5	5	5	39													
Tidak tahu	5	1	5	5	5	5	5	5	36	5	6	7	5	2	1	4	5	4	3	5	5	5
Kantong plastik,	4	5	5	5	5	5	5	5	39	1	1	1	3	1	3	3	3	3	1	1	1	1
Kantong plastik,	5	5	5	5	5	5	5	5	40	1	1	1	2	1	2	1	2	1	1	2	2	2
Kantong plastik,	5	5	5	5	5	5	5	5	40	1	1	4	5	1	3	1	3	3	1	1	4	1
Kantong plastik	5	5	5	5	5	5	5	5	40	3	4	4	4	1	3	4	2	4	1	1	4	1
Kantong plastik,	5	5	5	5	5	5	5	5	40	1	1	4	5	1	4	1	1	3	4	1	1	3
Kantong plastik,	5	5	5	5	5	5	5	5	40	2	1	3	3	3	3	4	5	1	1	1	1	1
Sterofoam	3	4	4	4	4	5	5	5	34	5	5	5	5	3	4	6	2	6	5	3	3	4
Kantong plastik,	5	5	5	5	5	5	5	5	40	4	5	6	6	4	4	5	5	6	2	4	5	1
Kantong plastik,	1	5	1	5	5	5	5	5	32	5	1	1	1	1	1	1	1	1	1	1	1	1
Kantong plastik,	1	5	1	5	5	5	5	5	32	5	1	2	2	1	1	1	1	1	1	1	1	1
Kantong plastik,	4	5	5	5	5	4	4	5	37	5	4	4	5	6	7	5	6	6	5	5	6	6
Tidak tahu	3	4	4	4	4	5	5	5	34													
Kantong Plastik	4	3	2	4	4	4	5	5	31													
Sterofoam	5	5	5	5	5	5	5	5	40													
botol minuman b	4	4	4	4	4	4	4	4	32													
Kantong Plastik,	5	5	5	5	5	5	5	5	40													
Kantong Plastik	4	5	4	5	5	5	5	5	38													
kemasan plastik	5	5	5	5	5	5	5	5	40													
Sterofoam	4	4	4	4	4	4	4	4	32													
Kantong plastik,	3	3	3	3	3	3	3	3	24													
Kantong plastik,	5	5	5	5	5	5	5	5	40													
Kantong plastik,	4	4	4	4	4	4	4	4	32	3	3	3	3	1	3	3	3	3	3	3	3	3
Kantong plastik	5	5	5	5	5	5	5	5	40	3	4	6	6	2	2	4	6	4	2	3	3	1
Sterofoam	4	5	4	4	4	4	4	5	34	2	3	4	6	1	6	3	4	7	7	1	7	4
Sterofoam, Kem	5	5	5	5	5	5	5	5	40	7	7	5	7	7	7	5	5	5	6	7	7	7
Sterofoam	4	4	4	4	4	4	4	4	32	3	3	3	3	3	3	3	3	3	3	3	3	3
Kantong plastik,	5	5	5	5	5	5	5	5	40	7	7	7	7	7	7	7	7	7	7	7	7	7
Sterofoam	5	5	5	5	5	5	5	5	40	7	1	1	1	1	4	3	4	3	2	1	1	2
Kantong plastik,	4	5	3	5	5	5	5	5	37	1	1	3	3	4	4	1	4	4	4	2	1	1
Kantong plastik,	4	5	4	5	5	5	5	5	38	1	1	1	1	1	1	1	1	1	1	1	1	1
Tidak tahu	3	5	3	5	5	5	5	5	36	1	1	3	4	1	1	1	1	1	1	1	1	2
Kantong plastik	5	5	5	5	5	5	5	5	40	2	1	4	5	1	3	3	2	4	2	1	1	1
Kantong plastik,	3	3	3	3	3	3	3	3	24	1	1	1	1	1	1	1	1	1	1	1	1	1
Kantong plastik,	5	5	5	5	5	5	5	5	40	2	2	2	2	2	1	1	1	1	1	1	1	1
Sterofoam	4	4	4	4	4	4	4	4	32	1	1	1	1	1	1	1	1	1	1	1	1	1
Kemasan plastik	4	4	4	4	4	4	4	4	32	1	1	2	2	2	2	2	2	2	2	1	1	1
Kantong plastik	5	5	5	5	5	5	5	5	40	1	1	1	1	1	1	1	1	1	1	1	1	1
Sterofoam	3	5	5	5	4	5	5	5	37	6	6	7	7	6	5	5	6	4	6	6	6	4
Sterofoam, Kem	5	5	5	4	5	5	5	5	39	4	5	4	5	5	5	5	2	5	5	1	1	5
Kantong plastik,	4	4	5	4	5	4	5	5	36	3	2	2	2	2	3	3	2	2	3	3	2	2
Sterofoam	3	3	4	4	4	4	4	4	30	1	1	3	3	4	2	2	1	1	1	1	1	4
Kantong plastik	5	5	5	5	5	5	5	5	40	1	1	2	2	1	2	1	1	1	1	1	1	1

14. Apakah And	Skor GSRS (Ga	Apakah terdapa	Apakah terdapa	HeartScore (jan	1. Secara umur	2. Seberapa ser	3. Dalam 2 ming	4. Secara umun	5. Berapa banya	6. Seberapa ser	7. Seberapa bal	8. Secara umun	9. Seberapa ser	10. Seberapa se	11. Seberapa se	12. Seberapa se	13. Seberapa se	14. Seberapa se	15. Seberapa se	16. Seberapa se	17. Seberapa se	18. Seberapa se
		Chest pain atipik	Tidak ada faktor	0	2	2	2	2	6	2	2	2	2	2	2	2	2	1	2	1	1	2
		Sangat curiga ch	Tidak ada faktor	2	5	2	4	1	6	6	4	4	2	2	2	2	2	2	2	4	3	2
1	16	Nyeri dada atipik	Tidak ada faktor	0	2	2	5	2	4	2	3	2	2	1	2	1	2	1	2	2	2	1
1	13	Tidak ada nyeri (	Tidak ada faktor	0	1	1	1	1	1	1	1	1	1	1	1	1	1	1	1	1	1	1
1	15	Sangat curiga ny	Tidak ada faktor	2	2	1	3	1	3	2	5	1	1	1	1	1	1	1	1	3	1	1
1	14	Curiga nyeri dad	Tidak ada faktor	1	2	2	2	1	4	2	7	2	1	1	1	1	1	1	2	1	1	1
1	15	Curiga nyeri dad	Tidak ada faktor	1	3	3	3	3	3	5	6	1	1	1	1	3	2	2	1	4	1	1
1	48	Tidak ada nyeri (	1 atau 2 faktor ri	1	1	3	5	7	3	7	4	7	1	7	1	4	4	1	4	1	1	6
		Chest pain atipik	>2 faktor risiko a	2	2	1	1	1	6	2	2	1	1	1	1	1	1	2	2	1	1	1
1	23	Curiga nyeri dad	Tidak ada faktor	1	5	2	4	4	2	5	4	5	1	5	1	3	5	1	5	5	5	2
1	13	Nyeri dada atipik	1 atau 2 faktor ri	1	1	1	1	2	4	2	6	1	1	1	1	2	2	1	1	2	1	1
1	13	Nyeri dada atipik	1 atau 2 faktor ri	1	1	1	1	1	1	1	1	1	1	1	1	1	1	1	1	1	1	1
2	18	Nyeri dada atipik	1 atau 2 faktor ri	1	2	3	2	2	2	2	2	5	2	4	3	4	4	3	5	1	1	2
		Chest pain atipik	Tidak ada faktor	0	5	7	7	5	7	7	7	7	7	7	7	7	7	1	1	1	1	1
1	26	Nyeri dada atipik	Tidak ada faktor	0	6	6	4	5	2	6	7	6	6	6	6	6	6	6	6	6	6	6
2	26	Tidak ada nyeri (	Tidak ada faktor	0	2	2	2	2	2	2	2	2	2	2	2	2	2	2	2	2	2	2
		Sangat curiga ch	1 atau 2 faktor ri	3	7	7	7	6	6	6	6	7	5	5	7	6	6	6	6	6	6	5
		Curiga chest pai	Tidak ada faktor	1	3	1	1	1	6	2	1	1	5	1	1	1	1	2	1	2	5	1
2	44	Nyeri dada atipik	1 atau 2 faktor ri	1	3	3	4	4	2	4	5	4	3	6	3	5	3	3	6	5	4	5
1	13	Nyeri dada atipik	Tidak ada faktor	0	1	1	1	1	1	1	1	1	1	1	1	1	1	1	1	1	1	1
5	61	Sangat curiga ny	1 atau 2 faktor ri	3	5	5	5	5	5	5	5	5	5	5	5	5	4	5	4	4	4	4
		Nyeri dada atipik	Tidak ada faktor	0	2	1	1	1	7	2	1	1	1	1	1	1	1	1	1	2	1	1
1	13	Sangat curiga ny	1 atau 2 faktor ri	3	4	1	2	2	2	3	2	2	1	1	1	1	1	1	1	1	1	1
1	14	Sangat curiga ny	Tidak ada faktor	2	3	1	1	1	5	2	1	1	1	1	1	1	1	1	1	3	1	1
5	38	Curiga nyeri dad	Tidak ada faktor	1	4	6	4	5	6	6	5	5	6	5	4	5	5	5	6	5	6	5
1	23	Tidak ada nyeri (	1 atau 2 faktor ri	1	2	2	1	1	7	4	4	4	1	4	1	7	1	1	1	4	1	4
4	54	Sangat curiga ny	Tidak ada faktor	2	7	7	7	7	7	7	6	7	6	7	6	7	6	7	7	7	6	6
1	27	Nyeri dada atipik	1 atau 2 faktor ri	1	2	3	2	1	4	4	2	2	2	2	1	2	1	1	2	5	1	2
		Nyeri dada atipik	Tidak ada faktor	0	2	1	1	1	1	1	6	1	1	1	1	1	1	1	1	1	1	1
		Chest pain atipik	Tidak ada faktor	0	3	2	5	2	5	2	6	2	2	4	1	1	1	1	2	1	1	2
1	13	Nyeri dada atipik	1 atau 2 faktor ri	1	5	1	5	2	5	4	5	3	1	1	1	1	1	1	1	1	1	1
1	30	Tidak ada nyeri (	1 atau 2 faktor ri	1	7	5	4	4	3	5	5	7	4	6	2	2	1	2	5	6	4	4
		Chest pain atipik	1 atau 2 faktor ri	1	3	2	5	2	4	2	6	3	4	5	2	1	3	4	2	6	1	1
		Curiga chest pai	Tidak ada faktor	1	2	2	2	1	6	1	2	3	1	1	1	1	1	1	1	2	1	1
		Chest pain atipik	Tidak ada faktor	0	4	1	1	1	7	3	2	3	2	2	1	1	1	1	1	1	1	1
1	15	Nyeri dada atipik	Tidak ada faktor	0	2	1	7	1	7	1	1	1	1	1	1	1	1	1	1	1	1	1
1	15	Nyeri dada atipik	Tidak ada faktor	0	1	2	7	1	6	4	5	2	1	3	1	2	1	2	2	1	1	2
1	14	Tidak ada nyeri (	Tidak ada faktor	0	1	1	1	3	4	5	4	1	1	4	1	1	1	1	1	1	1	1
4	22	Tidak ada nyeri (	Tidak ada faktor	0	1	4	1	3	5	4	4	5	3	4	1	1	3	1	3	4	1	2
4	31	Nyeri dada atipik	Tidak ada faktor	0	5	5	2	6	3	5	5	4	3	4	2	2	2	2	6	6	4	4
7	66	Nyeri dada atipik	>2 faktor risiko a	2	3	3	1	3	2	3	4	4	5	3	2	3	3	4	3	4	2	3
		Curiga chest pai	1 atau 2 faktor ri	2	5	4	5	4	5	5	4	3	5	3	1	4	1	1	5	5	1	4
5	58	Nyeri dada atipik	Tidak ada faktor	0	7	7	4	7	3	7	5	5	3	4	2	2	2	1	5	2	1	1
1	14	Curiga nyeri dad	1 atau 2 faktor ri	2	2	2	1	1	7	2	7	1	1	1	1	2	1	1	1	2	1	1
1	13	Tidak ada nyeri (	Tidak ada faktor	0	5	5	4	4	3	5	3	4	5	6	1	1	1	1	3	5	1	1
2	49	Sangat curiga ny	1 atau 2 faktor ri	3	3	3	5	3	5	5	4	4	5	3	3	2	3	3	3	3	2	3
1	29	Tidak ada nyeri (	Tidak ada faktor	0	3	1	1	1	4	1	7	1	1	1	1	1	1	1	1	1	2	1
		Chest pain atipik	Tidak ada faktor	0	2	3	4	4	4	4	4	4	4	3	3	3	3	3	3	3	3	3
		Sangat curiga ch	Tidak ada faktor	2	4	4	4	4	4	4	4	4	4	4	4	4	4	4	4	4	4	4
3	35	Sangat curiga ny	Tidak ada faktor	2	4	4	6	4	5	3	4	4	2	2	1	3	1	1	1	2	1	2
1	20	Nyeri dada atipik	1 atau 2 faktor ri	1	1	1	1	1	7	1	4	1	1	1	1	1	1	1	1	1	1	1
1	13	Tidak ada nyeri (	1 atau 2 faktor ri	1	3	2	7	3	5	3	4	3	1	2	1	1	1	1	1	1	1	3
3	29	Tidak ada nyeri (	1 atau 2 faktor ri	1	2	4	3	5	2	5	3	4	2	4	3	5	4	2	3	4	2	4
		Sangat curiga ch	Tidak ada faktor	2	2	2	1	1	5	2	2	2	1	4	1	1	1	2	1	4	1	3
		Chest pain atipik	1 atau 2 faktor ri	1	1	4	1	3	2	5	4	4	2	1	1	1	1	1	1	5	1	1
		Curiga nyeri dad	1 atau 2 faktor ri	2	6	4	4	5	7	4	2	5	3	2	1	4	5	5	4	3	3	5
1	33	Nyeri dada atipik	1 atau 2 faktor ri	1	4	2	5	1	6	4	6	2	2	5	1	1	1	3	4	1	1	3
3	38	Nyeri dada atipik	Tidak ada faktor	0	5	5	5	5	5	5	5	5	5	5	5	5	5	5	5	5	5	5
1	18	Nyeri dada atipik	>2 faktor risiko a	2	6	5	4	2	5	3	2	2	1	2	1	1	1	1	2	1	1	2
1	13	Tidak ada nyeri (	Tidak ada faktor	0	2	1	2	1	6	1	1	2	1	1	1	1	1	1	1	1	1	3
1	19	Tidak ada nyeri (	Tidak ada faktor	0	1	3	7	1	7	1	1	1	1	1	1	1	1	1	1	1	1	1
		Sangat curiga ch	Tidak ada faktor	2	4	6	4	4	4	6	4	5	1	2	2	1	1	1	3	2	1	1
		Curiga chest pai	1 atau 2 faktor ri	2	2	1	1	1	1	2	1	1	1	1	1	3	1	2	1	3	1	1
		Chest pain atipik	Tidak ada faktor	0	1	2	7	1	7	1	1	7	1	2	1	1	1	1	1	1	1	1
		Curiga chest pai	>2 faktor risiko a	3	4	3	4	2	2	3	3	3	2	3	2	2	2	2	3	2	2	2
		Chest pain atipik	1 atau 2 faktor ri	1	4	5	4	6	4	6	4	5	5	4	5	3	4	6	4	5	4	4
		Curiga nyeri dad	1 atau 2 faktor ri	2	3	2	2	1	5	4	3	2	1	2	1	1	1	1	1	3	1	2
3	45	Sangat curiga ny	1 atau 2 faktor ri	3	4	4	3	4	3	4	4	4	4	5	3	3	3	3	2	4	3	4
4	28	Nyeri dada atipik	Tidak ada faktor	0	2	2	7	3	5	3	1	2	1	2	1	1	1	2	2	2	1	2
1	17	Nyeri dada atipik	1 atau 2 faktor ri	1	3	4	7	4	2	5	5	3	3	4	2	5	1	1	5	4	1	3
1	13	Tidak ada nyeri (	1 atau 2 faktor ri	1	5	1	1	1	7	1	1	1	5	1	1	1	1	1	1	1	1	1
		Curiga chest pai	>2 faktor risiko a	3	5	7	2	7	1	7	7	7	3	7	1	7	7	1	7	1	1	7
1	37	Nyeri dada atipik	Tidak ada faktor	0	1	6	1	6	5	5	4	1	3	6	1	1	1	2	5	3	1	5
1	15	Tidak ada nyeri (	1 atau 2 faktor ri	1	3	3	1	1	6	4	1	2	4	1	1	1	1	1	1	4	2	1
1	26	Sangat curiga ny	1 atau 2 faktor ri	3	3	3	4	3	3	3	4	3	2	5	1	1	1	3	3	5	1	4
		Nyeri dada atipik	Tidak ada faktor	0	6	6	7	2	5	4	6	6	1	7	1	1	1	1	5	2	4	6
1	13	Curiga nyeri dad	1 atau 2 faktor ri	2	4	4	3	4	4	5	3	1	2	3	1	2	1	2	5	3	1	3
		Curiga chest pai	Tidak ada faktor	1	4	3	5	3	4	4	4	2	2	5	3	4	3	1	2	2	2	2
4	47	Curiga nyeri dad	1 atau 2 faktor ri	2	3	4	3	1	1	1	1	1	1	2	2	1	1	1	1	1	4	2
1	19	Tidak ada nyeri (	1 atau 2 faktor ri	1	2	2	1	1	7	1	2	2	2	7	1	1	1	1	1	1	1	1
		Sangat curiga ch	>2 faktor risiko a	4	1	1	4	1	7	1	7	7	1	1	1	1	1	1	1	1	1	1
		Sangat curiga ch	Tidak ada faktor	2	1	1	3	1	7	4	4	2	1	1	1	1	1	1	2	1	1	1
		Curiga chest pai	1 atau 2 faktor ri	2	5	5	3	5	4	6	4	6	1	5	1	5	1	1	6	1	1	6
		Curiga chest pai	1 atau 2 faktor ri	2	3	7	4	4	3	6	5	6	4	1	3	1	2	2	4	5	2	2
		Curiga nyeri dad	1 atau 2 faktor ri	2	3	3	2	3	6	4	2	2	1	3	1	4	1	2	2	3	1	1
1	17	Curiga nyeri dad	1 atau 2 faktor ri	2	3	3	7	7	7	5	1	2	1	2	1	1	1	2	1	3	2	2
1	18	Nyeri dada atipik	Tidak ada faktor	0	6	1	1	1	1	6	3	1	2	3	1	1	1	2	1	5	1	2
1	13	Nyeri dada atipik	>2 faktor risiko a	2	2	1	1	1	1	1	1	1	1	1	1	1	1	1	1	3	1	1
6	73	Nyeri dada atipik	Tidak ada faktor	0	4	5	6	5	5	5	5	5	5	6	5	5	5	5	5	4	4	4
2	26	Nyeri dada atipik	1 atau 2 faktor ri	1	4	2	6	2	6	2	6	2	2	2	2	2	2	2	2	2	2	2
1	17	Nyeri dada atipik	1 atau 2 faktor ri	1	2	2	2	1	4	2	4	4	2	1	1	1	1	1	2	2	1	3
2	15	Tidak ada nyeri (	Tidak ada faktor	0	2	4	3	3	5	3	2	3	1	2	1	2	1	2	3	2	2	3
1	18	Tidak ada nyeri (	Tidak ada faktor	0	1	1	1	1	5	1	5	1	1	1	1	1	1	1	1	3	1	1
1	13	Tidak ada nyeri (	Tidak ada faktor	0	2	2	1	1	7	1	2	1	1	1	1	1	1	1	1	1	1	1
2	30	Tidak ada nyeri (	>2 faktor risiko a	2	2	1	4	1	5	1	1	1	3	1	1	1	1	1	1	5	1	1
1	13	Tidak ada nyeri (	Tidak ada faktor	0	1	1	2	1	2	2	3	2	3	1	1	1	1	1	1	2	1	2
1	28	Tidak ada nyeri (	1 atau 2 faktor ri	1	2	3	4	3	3	5	4	5	3	4	1	3	3	1	3	5	2	3
		Sangat curiga ch	1 atau 2 faktor ri	3	5	2	5	2	4	2	4	2	6	4	2	1	1	1	1	2	3	2
2	46	Nyeri dada atipik	>2 faktor risiko a	2	3	3	2	3	3	6	5	6	5	2	2	3	4	4	4	5	2	6
1	22	Nyeri dada atipik	Tidak ada faktor	0	3	5	2	2	4	3	6	3	2	2	1	2	1	1	6	3	1	4
1	30	Tidak ada nyeri (	>2 faktor risiko a	2	3	3	4	1	2	6	4	2	1	2	1	2	2	1	1	6	1	2
1	13	Tidak ada nyeri (	Tidak ada faktor	0	1	2	1	1	1	1	1	1	1	1	1	1	1	1	1	5	1	1
4	50	Tidak ada nyeri (	1 atau 2 faktor ri	1	4	4	2	1	5	5	3	5	1	5	1	1	1	1	5	4	1	2
		Chest pain atipik	1 atau 2 faktor ri	1	4	5	4	6	3	7	6	5	3	5	1	1	1	1	6	7	2	5
2	29	Curiga nyeri dad	1 atau 2 faktor ri	2	1	4	4	1	4	4	7	5	1	5	1	5	1	1	5	3	2	3
7	91	Nyeri dada atipik	Tidak ada faktor	0	7	7	7	7	7	7	7	7	7	7	7	7	7	7	7	7	7	7
1	13	Tidak ada nyeri (	1 atau 2 faktor ri	1	1	1	1	1	7	2	1	1	1	2	1	1	1	1	1	1	1	1
4	52	Nyeri dada atipik	Tidak ada faktor	0	1	1	1	1	7	5	4	1	1	4	1	1	1	1	4	6	1	3
		Curiga chest pai	>2 faktor risiko a	3	6	7	5	5	5	7	2	6	4	6	4	6	6	5	6	6	2	5
		Curiga chest pai	Tidak ada faktor	1	1	4	5	4	5	4	4	4	2	1	1	1	1	1	4	3	1	5
		Sangat curiga ch	Tidak ada faktor	2	2	3	1	1	7	2	1	1	1	2	1	1	2	3	1	3	1	1
		Chest pain atipik	Tidak ada faktor	0	3	2	1	2	5	3	2	3	2	2	1	1	1	1	2	1	1	2
		Chest pain atipik	Tidak ada faktor	0	1	1	3	2	7	1	1	1	1	1	1	1	1	2	1	1	1	1
		Sangat curiga ny	Tidak ada faktor	2	2	2	1	2	5	2	6	2	1	1	2	1	1	1	1	1	1	1
1	27	Curiga nyeri dad	1 atau 2 faktor ri	2	5	2	3	1	4	5	3	3	5	2	1	1	1	3	5	4	3	2
1	22	Nyeri dada atipik	Tidak ada faktor	0	2	1	1	1	1	1	1	1	1	1	1	1	1	1	1	1	1	1
2	28	Nyeri dada atipik	1 atau 2 faktor ri	1	2	2	2	2	2	2	5	2	2	3	2	2	2	2	2	2	2	2
2	33	Nyeri dada atipik	Tidak ada faktor	0	6	6	4	6	1	7	6	6	4	5	3	6	2	1	4	6	1	4
1	23	Tidak ada nyeri (	1 atau 2 faktor ri	1	4	1	2	1	6	4	2	1	1	5	1	2	1	1	1	1	1	1
1	13	Tidak ada nyeri (	1 atau 2 faktor ri	1	2	1	1	1	6	2	1	1	1	1	1	1	1	1	1	4	1	1
1	45	Sangat curiga ny	>2 faktor risiko a	4	4	6	5	7	3	5	6	6	1	6	4	1	6	2	7	5	5	6
2	41	Tidak ada nyeri (	Tidak ada faktor	0	3	2	2	1	5	3	7	2	3	1	1	1	1	1	1	1	1	1
1	33	Tidak ada nyeri (	1 atau 2 faktor ri	1	3	5	5	4	5	5	3	4	3	4	2	3	2	1	5	4	1	1
4	49	Tidak ada nyeri (	1 atau 2 faktor ri	1	4	5	4	5	4	5	5	5	3	6	3	2	1	1	4	5	1	4
1	15	Tidak ada nyeri (	Tidak ada faktor	0	5	1	1	1	4	5	5	1	1	5	1	1	1	1	1	1	1	1
1	13	Tidak ada nyeri (	Tidak ada faktor	0	1	1	1	1	1	1	1	1	1	1	1	1	1	1	1	5	1	1
		Chest pain atipik	Tidak ada faktor	0	1	3	7	2	7	3	1	2	2	3	1	1	1	3	1	3	1	2
		Curiga chest pai	>2 faktor risiko a	3	4	4	4	4	5	4	4	4	5	2	4	6	4	2	4	5	5	4
4	58	Nyeri dada atipik	1 atau 2 faktor ri	1	3	1	4	1	5	5	1	1	4	1	1	1	2	3	1	3	1	1
3	64	Nyeri dada atipik	1 atau 2 faktor ri	1	6	6	6	7	3	6	3	6	6	7	7	7	5	5	6	5	6	7
1	19	Curiga nyeri dad	Tidak ada faktor	1	5	5	3	5	5	5	6	5	2	2	2	3	2	2	5	1	1	4
4	48	Nyeri dada atipik	Tidak ada faktor	0	2	3	2	2	6	5	4	3	2	5	2	5	1	2	2	3	1	1
1	29	Nyeri dada atipik	Tidak ada faktor	0	5	2	1	1	7	4	4	4	4	4	4	4	4	4	4	4	4	4
2	62	Nyeri dada atipik	>2 faktor risiko a	2	6	2	7	4	6	6	7	1	1	1	1	1	1	1	5	7	1	4
1	15	Tidak ada nyeri (	1 atau 2 faktor ri	1	1	1	1	1	3	1	3	1	1	3	1	1	1	1	3	1	1	1
1	27	Tidak ada nyeri (	1 atau 2 faktor ri	1	1	1	2	1	5	4	6	4	2	1	4	1	4	1	1	2	1	2
1	26	Tidak ada nyeri (	Tidak ada faktor	0	1	1	1	1	7	3	1	4	3	5	7	1	1	1	1	1	1	3
		Curiga chest pai	1 atau 2 faktor ri	2	3	2	2	2	5	6	5	5	1	5	1	1	1	1	3	2	1	3
		Chest pain atipik	1 atau 2 faktor ri	1	5	6	4	6	4	5	4	6	3	6	2	7	5	3	7	4	3	5
1	40	Nyeri dada atipik	Tidak ada faktor	0	5	1	1	1	3	2	7	1	1	1	1	1	1	1	1	2	1	1
1	14	Tidak ada nyeri (	1 atau 2 faktor ri	1	2	5	5	5	5	6	4	2	2	1	1	1	1	1	2	1	1	2
		Chest pain atipik	1 atau 2 faktor ri	1	7	7	1	7	4	4	7	4	1	7	1	1	1	1	7	1	1	3
		Curiga chest pai	>2 faktor risiko a	3	7	7	1	7	1	1	7	7	7	7	7	1	7	5	7	6	4	7
		Chest pain atipik	Tidak ada faktor	0	3	3	4	3	4	5	5	4	2	3	1	2	1	2	3	4	2	2
3	23	Sangat curiga ny	>2 faktor risiko a	4	3	3	3	3	3	3	3	3	3	3	3	3	3	3	3	3	3	3
		Chest pain atipik	1 atau 2 faktor ri	1	5	1	1	1	4	6	3	1	1	1	1	1	1	1	1	4	1	1
1	32	Curiga nyeri dad	Tidak ada faktor	1	2	3	3	2	5	2	3	3	2	4	1	1	1	1	2	1	1	2
4	52	Sangat curiga ny	>2 faktor risiko a	4	4	4	4	4	4	4	4	4	4	4	4	4	4	4	4	4	4	4
7	62	Tidak ada nyeri (	>2 faktor risiko a	2	6	2	4	2	5	7	4	4	2	2	1	1	1	1	7	7	2	4
1	29	Tidak ada nyeri (	Tidak ada faktor	0	2	2	1	1	5	5	5	2	1	2	1	1	1	1	1	2	1	1
1	30	Tidak ada nyeri (	1 atau 2 faktor ri	1	1	1	7	1	5	1	3	1	1	1	1	1	1	1	1	6	1	1
1	16	Tidak ada nyeri (	Tidak ada faktor	0	1	1	1	1	1	1	1	1	1	1	1	1	1	1	1	3	1	1
		Curiga chest pai	1 atau 2 faktor ri	2	4	5	5	6	7	3	7	7	4	3	3	2	2	2	1	2	2	2
1	22	Nyeri dada atipik	>2 faktor risiko a	2	4	3	4	3	1	4	5	1	1	2	1	1	1	1	1	5	1	1
1	14	Nyeri dada atipik	Tidak ada faktor	0	2	1	2	1	6	4	1	1	1	3	1	1	1	1	1	1	1	1
4	51	Sangat curiga ny	Tidak ada faktor	2	2	4	3	1	2	1	6	1	1	2	1	4	5	1	2	6	2	2
1	13	Curiga nyeri dad	1 atau 2 faktor ri	2	1	3	3	4	6	4	1	4	1	3	4	4	4	1	1	3	1	1
2	32	Nyeri dada atipik	1 atau 2 faktor ri	1	4	2	5	4	4	5	4	3	2	2	2	2	2	2	2	4	2	2
2	18	Nyeri dada atipik	1 atau 2 faktor ri	1	3	2	4	5	2	5	1	3	2	4	1	3	1	1	2	5	1	2
3	45	Tidak ada nyeri (	1 atau 2 faktor ri	1	5	6	3	5	5	5	4	5	5	2	4	3	4	2	5	3	4	5
2	26	Nyeri dada atipik	1 atau 2 faktor ri	1	2	2	5	2	5	2	4	2	3	2	2	2	2	2	2	2	2	2
1	21	Tidak ada nyeri (	Tidak ada faktor	0	7	3	4	4	4	4	4	5	1	3	1	1	1	1	4	1	1	4
1	41	Tidak ada nyeri (	Tidak ada faktor	0	4	1	5	1	6	1	6	1	1	1	1	1	7	1	1	1	1	1
3	18	Tidak ada nyeri (	1 atau 2 faktor ri	1	2	3	2	2	3	2	2	1	1	2	1	1	1	1	2	1	1	1
1	20	Tidak ada nyeri (	1 atau 2 faktor ri	1	4	2	2	1	6	4	6	2	1	3	1	1	1	1	1	1	1	1
		Chest pain atipik	Tidak ada faktor	0	5	4	5	3	2	5	4	5	3	1	3	3	1	2	3	4	1	3
		Chest pain atipik	Tidak ada faktor	0	6	6	4	6	3	6	5	6	6	6	6	3	4	4	4	5	2	6
		Chest pain atipik	>2 faktor risiko a	2	1	4	4	5	5	6	3	5	5	1	2	1	1	5	5	7	6	6
		Sangat curiga ch	1 atau 2 faktor ri	3	5	5	3	7	3	4	3	3	3	4	2	1	1	1	1	2	1	1
		Chest pain atipik	Tidak ada faktor	0	7	7	2	7	1	7	1	7	2	7	1	2	3	4	3	7	2	4
1	13	Nyeri dada atipik	1 atau 2 faktor ri	1	3	2	4	1	5	4	5	2	5	3	1	1	1	1	2	5	1	2
3	17	Curiga nyeri dad	Tidak ada faktor	1	6	5	3	6	1	1	4	7	1	4	3	5	5	4	4	4	3	3
2	16	Nyeri dada atipik	>2 faktor risiko a	2	5	3	7	1	6	4	6	2	4	5	1	1	1	1	3	3	1	1
3	42	Nyeri dada atipik	1 atau 2 faktor ri	1	5	5	3	5	1	5	1	5	5	1	1	1	3	3	5	5	1	4
5	38	Nyeri dada atipik	Tidak ada faktor	0	1	3	1	1	4	3	3	3	1	1	1	2	4	1	3	1	1	1
1	42	Curiga nyeri dad	Tidak ada faktor	1	7	3	3	3	3	7	5	7	4	7	4	4	3	1	5	7	7	5
4	22	Tidak ada nyeri (	Tidak ada faktor	0	5	5	1	5	2	7	7	6	2	7	1	4	1	1	7	2	1	5
1	13	Tidak ada nyeri (	Tidak ada faktor	0	1	1	1	1	5	4	2	1	1	1	1	5	1	1	1	1	1	2
2	39	Tidak ada nyeri (	Tidak ada faktor	0	3	3	4	3	2	5	5	3	5	2	2	2	2	2	2	7	4	3
5	56	Nyeri dada atipik	1 atau 2 faktor ri	1	7	6	6	7	3	7	3	7	1	7	1	2	1	2	4	2	1	4
1	20	Curiga nyeri dad	1 atau 2 faktor ri	2	2	1	1	1	5	4	4	3	3	2	1	1	1	2	2	3	1	1
2	15	Tidak ada nyeri (	1 atau 2 faktor ri	1	3	3	1	2	6	2	1	1	1	1	1	2	1	1	2	4	4	1
2	40	Tidak ada nyeri (	1 atau 2 faktor ri	1	1	1	1	3	5	1	1	3	1	6	1	1	1	1	2	1	1	1
5	27	Curiga nyeri dad	1 atau 2 faktor ri	2	2	6	3	3	5	7	3	3	3	6	1	3	1	1	5	6	1	3
2	18	Tidak ada nyeri (	Tidak ada faktor	0	1	1	1	1	7	1	1	1	1	1	1	1	1	1	1	1	1	1
		Curiga chest pai	Tidak ada faktor	1	2	1	3	1	5	2	2	3	2	1	1	2	1	1	2	1	1	1
		Curiga chest pai	1 atau 2 faktor ri	2	6	6	4	5	4	7	2	4	4	6	6	5	5	7	6	6	5	6
1	39	Curiga nyeri dad	1 atau 2 faktor ri	2	3	1	3	1	4	4	1	2	4	3	2	2	1	2	2	3	3	3
1	13	Nyeri dada atipik	1 atau 2 faktor ri	1	5	1	1	1	4	4	4	4	4	4	4	4	4	4	4	4	4	4
4	32	Sangat curiga ny	Tidak ada faktor	2	4	3	2	2	3	4	2	2	1	4	1	2	2	2	2	2	2	3
1	47	Tidak ada nyeri (	1 atau 2 faktor ri	1	4	4	6	5	3	7	3	3	5	5	1	1	1	1	3	5	1	2

4	68	Curiga nyeri dad	1 atau 2 faktor ri	2	6	6	5	4	3	5	6	6	5	4	4	4	6	4	4	3	6	3
1	13	Tidak ada nyeri (	Tidak ada faktor	0	3	3	2	5	4	4	4	5	2	1	1	1	1	2	3	1	1	1
1	13	Tidak ada nyeri (	Tidak ada faktor	0	4	5	7	5	4	5	2	2	1	5	2	2	2	1	4	2	1	2
1	17	Tidak ada nyeri (	Tidak ada faktor	0	1	3	1	1	2	3	1	1	1	1	1	1	1	1	1	2	1	1
		Curiga chest pai	1 atau 2 faktor ri	2	3	2	4	2	4	2	4	3	4	2	2	2	2	3	2	3	2	3
7	74	Curiga nyeri dad	Tidak ada faktor	1	6	5	5	4	4	6	4	6	4	6	4	4	2	4	4	6	4	6
5	46	Nyeri dada atipik	Tidak ada faktor	0	3	3	2	2	6	6	6	2	1	2	1	7	3	1	5	1	2	5
2	54	Curiga nyeri dad	Tidak ada faktor	1	7	7	2	7	1	7	2	7	7	6	3	4	4	5	6	7	3	7
1	14	Nyeri dada atipik	Tidak ada faktor	0	1	1	1	1	3	3	2	1	1	3	1	1	1	2	1	1	1	1
3	44	Sangat curiga ny	Tidak ada faktor	2	5	5	4	5	3	5	3	5	3	7	4	6	6	4	6	3	1	4
4	52	Nyeri dada atipik	Tidak ada faktor	0	4	4	4	4	4	4	4	4	4	4	4	4	4	4	4	4	4	4
1	13	Tidak ada nyeri (	1 atau 2 faktor ri	1	4	5	6	5	4	5	3	5	1	4	1	2	1	1	5	3	1	2
1	15	Tidak ada nyeri (	1 atau 2 faktor ri	1	5	6	1	4	4	6	6	2	3	3	1	2	1	1	6	1	1	2
1	27	Tidak ada nyeri (	>2 faktor risiko a	2	3	6	1	4	4	4	4	4	1	6	1	1	1	1	4	2	1	7
5	53	Tidak ada nyeri (	1 atau 2 faktor ri	1	4	3	4	5	2	5	3	4	5	4	3	5	3	3	3	6	3	5
1	13	Tidak ada nyeri (	1 atau 2 faktor ri	1	1	1	1	1	1	3	1	1	1	1	1	1	1	1	1	1	1	1
1	17	Tidak ada nyeri (	Tidak ada faktor	0	1	1	1	1	2	4	1	1	3	1	1	1	1	1	1	3	1	1
2	32	Tidak ada nyeri (	1 atau 2 faktor ri	1	5	7	1	5	5	5	4	4	3	7	1	2	1	4	4	6	2	5
		Chest pain atipik	1 atau 2 faktor ri	1	5	1	4	1	5	4	4	5	3	2	2	2	3	3	1	3	2	1
		Curiga chest pai	1 atau 2 faktor ri	2	4	4	4	4	4	4	4	4	4	4	4	4	4	4	4	4	4	4
5	65	Nyeri dada atipik	1 atau 2 faktor ri	1	6	4	3	4	3	5	3	6	6	6	3	5	5	3	5	5	3	4
5	66	Curiga nyeri dad	1 atau 2 faktor ri	2	6	6	5	5	4	6	5	5	4	4	4	4	4	4	5	5	4	5
5	57	Nyeri dada atipik	Tidak ada faktor	0	1	2	1	1	3	4	3	4	2	1	1	1	1	1	1	1	1	1
2	31	Nyeri dada atipik	Tidak ada faktor	0	3	2	6	2	4	3	5	2	1	1	1	6	1	1	2	2	1	3
1	15	Nyeri dada atipik	1 atau 2 faktor ri	1	5	1	2	1	5	2	4	2	3	1	2	3	3	3	3	3	3	3
1	19	Nyeri dada atipik	Tidak ada faktor	0	1	1	1	1	1	1	1	1	1	1	1	1	1	1	1	1	1	1
1	43	Tidak ada nyeri (	Tidak ada faktor	0	3	3	4	2	4	6	4	2	2	5	1	2	2	1	5	2	1	2
1	13	Tidak ada nyeri (	>2 faktor risiko a	2	1	1	1	1	7	5	6	1	1	1	1	1	1	1	1	2	1	1
5	46	Tidak ada nyeri (	1 atau 2 faktor ri	1	5	5	5	5	4	5	3	4	5	4	4	3	2	3	4	5	4	5
1	13	Curiga nyeri dad	Tidak ada faktor	1	3	5	7	2	4	1	3	3	1	5	1	2	1	1	4	1	1	2
		Chest pain atipik	1 atau 2 faktor ri	1	3	1	1	3	5	2	1	3	2	2	2	2	3	3	3	2	1	2
		Chest pain atipik	1 atau 2 faktor ri	1	5	6	6	6	4	4	5	6	5	3	1	1	1	1	6	2	1	6
		Curiga chest pai	1 atau 2 faktor ri	2	6	6	5	5	6	7	7	4	4	6	5	6	3	6	3	6	7	5
5	19	Nyeri dada atipik	Tidak ada faktor	0	6	5	1	2	2	7	5	5	1	5	1	1	1	1	3	6	4	4
1	21	Nyeri dada atipik	Tidak ada faktor	0	3	2	4	2	2	6	4	2	2	3	1	3	1	1	1	1	1	1
4	50	Nyeri dada atipik	>2 faktor risiko a	2	6	6	6	6	6	5	6	5	6	6	6	6	6	6	6	6	6	6
1	21	Nyeri dada atipik	Tidak ada faktor	0	4	1	4	1	6	4	6	4	1	1	2	3	1	1	2	2	3	1
1	20	Curiga nyeri dad	1 atau 2 faktor ri	2	4	4	6	4	4	4	2	5	2	5	1	1	1	1	4	2	1	3
4	43	Nyeri dada atipik	1 atau 2 faktor ri	1	2	1	1	2	5	2	2	1	1	2	1	1	1	1	1	2	1	2
1	27	Tidak ada nyeri (	Tidak ada faktor	0	2	2	2	1	4	4	3	1	2	1	1	1	1	1	1	3	1	2
4	52	Tidak ada nyeri (	Tidak ada faktor	0	3	3	3	3	3	3	3	3	3	3	3	3	3	3	3	3	3	3
1	16	Tidak ada nyeri (	1 atau 2 faktor ri	1	2	2	4	1	6	4	5	2	1	1	1	1	1	1	2	1	1	2
7	91	Sangat curiga ny	>2 faktor risiko a	4	7	7	7	7	7	7	7	7	7	7	7	7	7	7	7	7	7	7
2	28	Tidak ada nyeri (	Tidak ada faktor	0	3	3	2	3	5	4	5	4	2	1	4	4	3	1	3	1	1	3
4	43	Nyeri dada atipik	>2 faktor risiko a	2	3	2	4	4	4	5	4	4	4	4	2	2	3	3	3	5	3	4
		Curiga chest pai	1 atau 2 faktor ri	2	5	6	6	6	5	7	6	6	5	6	5	6	6	5	6	5	5	5
		Chest pain atipik	1 atau 2 faktor ri	1	2	3	5	2	5	4	4	2	1	4	1	1	2	1	2	2	1	2
		Curiga chest pai	1 atau 2 faktor ri	2	3	2	5	3	6	4	5	2	4	1	3	1	1	1	1	3	3	1
		Curiga chest pai	>2 faktor risiko a	3	5	5	2	5	3	5	3	3	4	5	4	4	4	4	6	4	1	3
1	13	Curiga nyeri dad	Tidak ada faktor	1	3	3	1	3	6	3	6	3	1	1	1	1	1	1	3	1	1	1
3	40	Sangat curiga ny	Tidak ada faktor	2	3	3	3	3	3	3	3	3	3	3	3	3	3	3	3	3	3	3
1	14	Curiga nyeri dad	1 atau 2 faktor ri	2	3	4	6	3	6	4	5	3	1	2	1	2	1	1	4	4	1	3
1	26	Sangat curiga ny	1 atau 2 faktor ri	3	7	7	7	6	6	6	4	5	6	6	2	2	5	5	6	5	1	4
2	63	Sangat curiga ny	Tidak ada faktor	2	7	7	7	7	1	7	7	7	7	7	2	1	1	7	7	7	5	7
1	13	Sangat curiga ny	Tidak ada faktor	2	1	1	1	1	7	7	7	1	1	1	1	1	1	1	1	2	1	1
3	43	Curiga nyeri dad	1 atau 2 faktor ri	2	4	2	5	2	5	3	5	6	2	5	2	1	2	2	2	4	2	2
2	18	Nyeri dada atipik	Tidak ada faktor	0	4	3	5	1	5	4	5	4	3	1	1	2	2	1	3	5	1	3
2	40	Nyeri dada atipik	1 atau 2 faktor ri	1	2	2	4	2	4	3	3	2	3	1	2	2	2	2	2	1	1	2
2	24	Curiga nyeri dad	Tidak ada faktor	1	7	2	1	1	5	2	3	3	1	4	1	1	1	2	1	3	1	1
1	24	Nyeri dada atipik	1 atau 2 faktor ri	1	5	2	4	3	4	6	3	5	2	5	1	1	1	2	2	6	1	4
1	15	Nyeri dada atipik	>2 faktor risiko a	2	5	3	6	3	3	3	3	1	1	1	1	1	1	1	1	1	1	1
1	13	Nyeri dada atipik	Tidak ada faktor	0	1	1	1	1	6	2	6	2	1	1	1	1	1	1	1	1	1	1
1	13	Nyeri dada atipik	1 atau 2 faktor ri	1	1	1	1	1	6	1	2	2	1	1	2	2	2	2	2	2	2	2
2	29	Nyeri dada atipik	1 atau 2 faktor ri	1	6	6	6	6	3	6	5	5	1	1	1	6	4	1	5	6	1	6
1	13	Nyeri dada atipik	1 atau 2 faktor ri	1	2	1	1	1	4	4	1	1	3	1	1	1	1	1	1	1	1	1
7	91	Curiga nyeri dad	1 atau 2 faktor ri	2	7	7	7	7	7	7	7	7	7	7	7	7	7	7	7	7	7	7
3	39	Nyeri dada atipik	Tidak ada faktor	0	4	4	3	3	3	3	3	3	3	3	3	3	3	3	3	3	3	3
1	18	Nyeri dada atipik	Tidak ada faktor	0	2	2	2	2	4	5	4	3	3	3	2	2	2	2	2	2	2	3
1	26	Nyeri dada atipik	Tidak ada faktor	0	2	5	3	3	1	6	5	5	1	3	1	1	2	1	3	1	5	3
4	67	Nyeri dada atipik	Tidak ada faktor	0	7	6	3	5	4	5	5	4	5	3	5	6	5	6	6	5	6	5
1	36	Nyeri dada atipik	1 atau 2 faktor ri	1	5	1	3	1	1	7	3	2	2	2	2	2	1	1	1	1	1	1
1	16	Nyeri dada atipik	Tidak ada faktor	0	2	3	2	2	4	4	4	2	1	3	1	1	1	1	2	1	1	2
1	32	Tidak ada nyeri (	Tidak ada faktor	0	3	2	3	1	4	2	2	2	1	3	1	1	1	1	2	2	1	2
3	43	Tidak ada nyeri (	>2 faktor risiko a	2	6	2	2	2	5	3	2	1	1	2	1	1	2	1	2	2	1	1
1	18	Tidak ada nyeri (	Tidak ada faktor	0	3	4	1	2	6	4	4	3	2	2	1	2	1	1	2	2	1	2
3	50	Tidak ada nyeri (	1 atau 2 faktor ri	1	4	1	5	5	4	6	4	5	5	2	2	4	3	2	1	5	2	2
4	37	Tidak ada nyeri (	Tidak ada faktor	0	6	6	4	5	6	7	4	6	7	3	4	6	7	1	7	4	5	3
6	69	Tidak ada nyeri (	1 atau 2 faktor ri	1	4	5	5	4	4	4	3	3	5	5	5	5	4	5	2	5	1	1
4	22	Tidak ada nyeri (	1 atau 2 faktor ri	1	1	1	1	1	4	4	2	1	1	1	1	1	1	1	1	1	4	1
2	44	Tidak ada nyeri (	Tidak ada faktor	0	2	2	2	2	4	3	4	3	3	4	2	4	3	1	2	3	2	2
3	30	Tidak ada nyeri (	1 atau 2 faktor ri	1	3	5	2	3	4	4	4	4	2	3	1	2	1	1	2	5	1	3
7	51	Tidak ada nyeri (	1 atau 2 faktor ri	1	1	1	1	1	6	5	1	1	1	3	1	1	1	1	1	5	1	3
1	21	Heart rate cende	1 atau 2 faktor ri	1	4	4	7	5	1	7	5	6	5	1	1	4	1	1	4	7	1	5
4	57	Nyeri dada atipik	1 atau 2 faktor ri	1	5	3	4	1	6	6	1	2	1	5	1	1	1	1	1	6	1	1
3	27	Tidak ada nyeri (	1 atau 2 faktor ri	1	3	2	2	3	3	2	2	1	1	4	1	2	1	1	1	1	1	3
1	31	Tidak ada nyeri (	>2 faktor risiko a	2	1	1	1	1	1	2	1	1	3	1	1	1	1	1	1	6	1	1
2	28	Tidak ada nyeri (	1 atau 2 faktor ri	1	1	1	1	1	1	1	1	1	1	1	1	1	1	1	1	1	1	1
1	13	Nyeri dada atipik	Tidak ada faktor	0	1	2	7	1	1	2	6	2	2	2	2	7	7	1	1	5	1	1
		Sangat curiga ch	1 atau 2 faktor ri	3	5	5	5	5	4	5	5	5	1	1	1	1	3	1	4	5	3	3
		Chest pain atipik	1 atau 2 faktor ri	1	5	3	4	3	4	4	4	3	2	2	1	2	1	1	2	2	1	2
		Nyeri dada atipik	Tidak ada faktor	0	4	3	4	2	6	3	4	2	2	1	1	2	2	2	2	2	2	2
1	17	Nyeri dada atipik	Tidak ada faktor	0	1	1	4	1	5	3	6	1	1	2	1	1	1	1	1	3	1	1
1	25	Nyeri dada atipik	1 atau 2 faktor ri	1	5	6	3	6	5	6	4	2	1	1	1	1	1	1	5	5	3	4
1		Nyeri dada atipik	Tidak ada faktor risiko		2	4	4	4	5	4	6	3	2	7	2	1	1	2	2	4	1	3
1	36	Curiga nyeri dad	>2 faktor risiko a	3	5	5	7	3	6	5	6	2	2	2	3	5	2	2	4	1	1	3
1	21	Nyeri dada atipik	Tidak ada faktor	0	2	1	7	1	5	6	2	1	1	2	1	1	1	1	1	1	1	1
1	16	Nyeri dada atipik	>2 faktor risiko a	2	1	1	1	1	6	2	1	1	1	1	1	1	1	1	1	1	1	1
1	13	Nyeri dada atipik	Tidak ada faktor	0	1	1	1	1	1	1	1	1	1	1	1	1	1	1	1	1	1	1
1	55	Nyeri dada atipik	>2 faktor risiko a	2	5	5	6	2	5	6	5	1	1	1	1	5	1	1	1	1	1	1
1	17	Nyeri dada atipik	>2 faktor risiko a	2	3	2	5	2	5	3	1	2	1	3	1	1	1	1	2	2	2	2
2	25	Nyeri dada atipik	Tidak ada faktor	0	5	1	6	4	1	6	2	5	1	3	1	3	1	1	1	6	1	1
1	30	Tidak ada nyeri (	Tidak ada faktor	0	3	2	4	3	5	2	5	2	1	2	2	2	2	2	2	2	1	2
3	39	Tidak ada nyeri (	1 atau 2 faktor ri	1	3	4	4	5	3	5	6	5	6	4	4	3	4	3	4	4	3	4
1	24	Tidak ada nyeri (	1 atau 2 faktor ri	1	4	1	4	1	5	3	2	5	4	1	1	1	1	1	1	5	1	1
1	26	Tidak ada nyeri (	1 atau 2 faktor ri	1	3	3	2	3	2	6	4	4	2	5	1	2	1	2	2	2	1	3
1	18	Tidak ada nyeri (	>2 faktor risiko a	2	5	3	4	2	4	5	5	4	1	3	1	1	1	1	1	1	1	1
1	13	Tidak ada nyeri (	Tidak ada faktor	0	3	1	1	1	7	4	7	1	1	1	1	1	1	1	1	1	1	1
3	28	Tidak ada nyeri (	1 atau 2 faktor ri	1	1	2	1	3	4	2	5	3	1	4	1	7	1	1	1	6	5	1
1	20	Tidak ada nyeri (	1 atau 2 faktor ri	1	6	1	1	1	6	5	7	1	1	4	1	3	1	1	1	5	7	2
1	30	Tidak ada nyeri (	1 atau 2 faktor ri	1	1	1	7	1	4	1	3	1	1	1	1	1	1	1	3	1	1	1
1	19	Tidak ada nyeri (	1 atau 2 faktor ri	1	4	6	4	6	2	6	2	6	6	6	4	3	6	2	7	4	2	6
		Sangat curiga ch	1 atau 2 faktor ri	3	7	3	3	4	3	7	6	3	3	5	6	2	1	1	1	1	6	5
		Curiga chest pai	Tidak ada faktor	1	1	1	1	1	7	2	1	1	1	1	1	1	1	1	1	3	1	1
		Sangat curiga ch	1 atau 2 faktor ri	3	5	5	5	6	4	6	5	5	1	7	1	1	6	1	6	4	1	6
		Curiga chest pai	1 atau 2 faktor ri	2	6	7	4	6	7	6	6	6	5	4	3	2	3	3	6	5	2	3
5	57	Nyeri dada atipik	1 atau 2 faktor ri	1	5	5	6	6	6	6	7	7	7	7	6	7	5	5	5	5	5	5
1	21	Curiga nyeri dad	Tidak ada faktor	1	6	5	3	4	4	5	4	4	4	5	5	3	3	3	3	3	3	4
2	19	Sangat curiga ny	>2 faktor risiko a	4	3	2	2	6	3	4	3	3	4	2	2	3	2	3	3	3	3	3
1	25	Curiga nyeri dad	Tidak ada faktor	1	2	1	7	1	6	6	4	1	3	1	1	1	1	1	1	3	1	1
2	34	Nyeri dada atipik	Tidak ada faktor	0	7	1	7	1	4	5	2	7	4	1	1	2	4	3	4	4	4	6
1	26	Nyeri dada atipik	Tidak ada faktor	0	5	5	1	1	3	6	2	5	1	6	1	1	1	1	3	4	2	3
1	27	Nyeri dada atipik	Tidak ada faktor	0	1	1	1	2	4	4	4	4	1	1	1	1	1	1	1	1	1	5
4	55	Tidak ada nyeri (	>2 faktor risiko a	2	6	5	2	4	3	6	4	2	1	5	2	2	2	1	4	5	1	3
3	54	Tidak ada nyeri (	1 atau 2 faktor ri	1	5	7	2	7	3	7	3	7	7	7	1	7	1	1	7	7	1	7
1	17	Tidak ada nyeri (	Tidak ada faktor	0	1	1	1	1	1	1	1	1	1	1	1	1	1	1	1	2	1	1
1	18	Tidak ada nyeri (	Tidak ada faktor	0	1	1	1	1	1	2	1	1	1	1	1	1	1	1	1	3	1	1
6	71	Curiga nyeri dad	>2 faktor risiko a	3	5	6	5	3	3	3	2	2	2	1	2	2	2	4	4	1	3	3
		Chest pain atipik	>2 faktor risiko a	2	5	2	4	3	5	5	5	5	1	5	1	1	1	1	1	5	1	1
		Chest pain atipik	>2 faktor risiko a	2	7	7	6	6	1	7	7	7	6	7	5	5	3	1	4	7	3	7
		Chest pain atipik	Tidak ada faktor	0	5	5	5	1	7	4	1	5	1	5	1	1	1	1	1	4	1	4
		Chest pain atipik	1 atau 2 faktor ri	1	3	3	3	3	3	3	3	3	3	3	3	3	3	3	3	3	3	3
		Chest pain atipik	Tidak ada faktor	0	7	4	4	4	4	6	4	4	4	4	4	4	4	4	4	4	4	4
		Chest pain atipik	1 atau 2 faktor ri	1	6	6	4	6	4	6	5	6	5	6	3	4	2	2	6	4	5	6
		Sangat curiga ch	>2 faktor risiko a	4	5	7	5	4	2	7	5	4	5	3	3	4	4	3	2	4	2	2
		Nyeri dada atipik	1 atau 2 faktor ri	1	4	3	4	4	5	6	3	3	5	5	3	4	4	4	4	4	4	4
		Nyeri dada atipik	1 atau 2 faktor ri	1	5	4	5	5	5	5	5	5	5	3	3	3	3	1	4	3	2	2
		Curiga nyeri dad	>2 faktor risiko a	3	6	6	4	6	5	5	3	4	4	4	2	7	4	4	4	7	2	5
3	37	Curiga nyeri dad	1 atau 2 faktor ri	2	1	1	1	1	1	1	1	1	1	1	1	1	1	1	1	1	1	1
3	43	Sangat curiga ny	>2 faktor risiko a	4	6	7	5	6	3	6	2	6	4	6	5	6	4	2	2	4	2	6
3	52	Curiga nyeri dad	1 atau 2 faktor ri	2	5	6	3	4	5	7	5	5	1	7	1	1	2	3	3	4	1	5
7	82	Nyeri dada atipik	>2 faktor risiko a	2	7	7	5	7	4	7	7	7	7	4	4	7	7	5	7	7	7	7
3	39	Nyeri dada atipik	Tidak ada faktor	0	3	3	7	1	3	1	3	3	3	3	1	1	1	1	4	4	4	4
7	91	Nyeri dada atipik	1 atau 2 faktor ri	1	7	7	7	7	7	7	7	7	7	7	7	7	7	7	7	7	7	7
1	31	Nyeri dada atipik	Tidak ada faktor	0	4	4	3	7	3	4	4	4	2	7	1	4	1	1	7	7	1	7
4	34	Curiga nyeri dad	>2 faktor risiko a	3	4	2	4	3	6	4	2	1	1	1	1	1	1	1	3	5	1	4
1	13	Curiga nyeri dad	>2 faktor risiko a	3	4	1	1	1	4	4	4	3	1	4	1	1	1	1	1	4	1	2
1	16	Nyeri dada atipik	Tidak ada faktor	0	4	4	7	2	4	4	7	2	1	4	1	5	1	1	1	1	1	1
2	27	Sangat curiga ny	>2 faktor risiko a	4	4	5	5	6	2	4	6	5	5	4	4	3	3	4	5	5	4	5
1	13	Nyeri dada atipik	>2 faktor risiko a	2	3	3	3	3	5	3	4	4	2	2	2	2	3	2	2	2	2	2
1	17	Nyeri dada atipik	Tidak ada faktor	0	3	2	1	1	4	4	2	2	2	2	2	2	2	2	2	2	1	2
1	13	Nyeri dada atipik	1 atau 2 faktor ri	1	2	2	1	1	2	2	2	2	2	2	2	2	2	2	2	2	2	2
2	21	Nyeri dada atipik	Tidak ada faktor	0	6	2	2	2	6	2	6	2	2	2	2	2	2	2	2	2	2	2
1	13	Nyeri dada atipik	Tidak ada faktor	0	3	1	1	1	1	1	1	1	1	1	1	1	1	1	1	1	1	1
6	73	Tidak ada nyeri (	Tidak ada faktor	0	5	5	3	5	4	6	3	5	6	7	4	5	1	1	5	5	1	5
4	51	Curiga nyeri dad	1 atau 2 faktor ri	2	3	4	4	4	2	6	3	5	6	5	5	4	5	5	4	7	6	3
2	31	Tidak ada nyeri (	>2 faktor risiko a	2	6	2	3	2	2	3	2	2	3	2	2	2	2	2	2	3	2	2
3	25	Curiga nyeri dad	1 atau 2 faktor ri	2	4	3	2	1	1	2	2	2	1	5	2	2	1	4	2	2	1	4
1	15	Tidak ada nyeri (	1 atau 2 faktor ri	1	3	3	5	3	2	5	3	5	3	5	1	1	1	1	1	1	1	1

19. Seberapa se	20. Secara umu	21. Seberapa se	22. Seberapa se	23. Seberapa se	24. Seberapa se	25. Seberapa se	26. Secara umu	27. Seberapa se	Suasana hati ya	Merasa cemas	Kegelisahan da	Nafsu makan m	Berat badan na	Pembengkakan	Menstruasi sak	Keluhan sebelu	Perdarahan me	Fantasi seksual	Kepuasan seks	Berkurangnya g	Nyeri di bagian	Perdarahan ber
2	1	2	2	1	1	1	1	1	2	2	2	2	2	2	2	2	1	3	3	3	2	2
2	2	5	2	2	2	2	2	2	1	1	1	1	1	1	1	1	1	1	1	1	1	1
2	1	2	2	1	1	1	1	1	1	2	1	2	2	1	2	2	3	2	3	2	2	1
1	1	1	1	1	1	1	1	1	1	1	1	1	1	1	1	1	1	1	1	1	1	1
1	1	2	1	1	1	1	1	1	1	1	1	3	1	1	1	1	1	1	1	1	2	1
1	1	1	1	1	1	1	1	1	1	1	5	5	1	1	1	1	1	1	1	1	1	1
1	1	1	1	1	1	1	1	1	1	1	1	1	1	1	4	4	2	1	1	1	2	1
1	1	1	1	1	1	1	1	1	1	4	4	5	3	1	1	1	1	1	1	1	1	1
1	1	1	4	1	1	1	1	1	1	1	1	3	2	1	1	1	1	1	1	1	1	1
2	2	2	2	5	1	1	1	1	3	3	4	1	2	1	4	4	4	2	2	2	4	1
1	1	1	2	1	1	1	1	1	1	1	1	1	2	1	1	1	1	1	1	1	1	1
1	1	1	1	1	1	1	1	1	1	1	1	1	1	1	1	1	1	1	1	1	1	1
1	1	4	1	3	1	1	1	1	3	1	2	2	1	1	1	2	1	3	3	3	1	1
1	1	1	1	1	1	1	1	1	3	3	3	3	3	1	1	1	1	1	1	1	2	1
6	6	6	6	6	6	6	6	6	4	4	4	4	4	4	3	4	3	1	1	1	4	4
2	2	3	2	2	2	2	2	2	2	2	2	2	2	2	2	2	2	2	2	2	2	2
6	6	6	5	6	6	5	6	5	5	4	5	5	5	5	4	1	4	5	4	4	5	4
1	2	1	1	1	1	1	1	1	3	1	1	3	3	3	2	2	1	1	1	1	1	1
5	3	4	4	5	3	2	3	3	4	4	5	3	3	2	4	4	3	4	4	4	4	1
1	1	1	1	1	1	1	1	1	1	1	1	1	1	1	1	1	1	1	1	1	1	1
5	4	4	4	4	4	4	4	4	3	4	4	4	4	4	4	4	4	4	4	4	4	4
1	1	1	1	1	1	1	1	1	1	1	1	2	2	1	1	1	1	1	1	1	1	1
2	1	1	1	1	1	1	1	1	1	1	2	1	2	1	1	3	2	2	1	1	1	1
2	1	6	1	1	1	1	1	1	1	1	2	1	1	1	3	2	1	1	1	1	1	1
6	6	6	6	6	1	1	1	1	4	4	4	4	3	3	1	1	1	1	1	1	3	1
1	1	1	1	1	1	1	1	1	1	1	1	1	1	1	1	1	1	1	1	1	1	1
7	7	7	6	7	7	7	6	7	5	5	5	5	5	5	5	5	5	5	5	5	5	5
2	1	2	2	2	1	1	1	1	1	2	2	4	4	3	1	1	1	2	2	2	2	1
4	1	1	1	1	1	1	1	1	2	1	2	1	1	1	1	1	1	1	1	3	3	1
1	1	4	1	1	1	1	1	4	1	2	1	2	1	1	1	1	2	3	3	3	2	1
1	1	1	1	1	1	1	1	1	1	1	1	1	1	1	1	1	1	1	1	1	1	1
2	4	2	1	2	1	1	1	1	4	4	5	2	3	1	5	5	5	1	1	1	5	1
6	1	2	3	4	1	1	1	1	4	2	3	5	3	1	4	3	2	2	2	2	4	1
1	1	3	1	1	1	1	1	1	1	1	1	3	2	1	1	1	1	1	1	1	1	1
1	1	1	1	1	1	1	1	1	3	1	1	2	2	1	1	1	1	4	4	4	1	1
1	1	1	1	1	1	1	1	1	1	1	1	1	1	1	1	2	2	1	1	1	1	1
2	1	1	3	3	1	1	1	1	1	1	1	1	1	1	4	3	1	1	1	1	1	1
1	1	5	1	3	1	1	1	1	4	3	3	3	3	3	1	1	1	1	1	1	1	1
1	1	1	2	3	1	1	3	1	1	1	2	2	1	1	1	1	1	1	1	1	1	1
6	4	6	4	1	2	2	4	4	4	4	4	3	3	1	1	1	1	4	3	4	4	1
6	3	3	2	5	3	3	3	1	1	1	1	1	1	1	1	1	1	1	1	1	1	1
4	1	7	1	2	1	1	1	1	3	3	3	3	4	1	1	1	1	1	1	1	1	1
1	1	1	1	6	1	1	1	1	5	5	5	2	2	1	3	4	3	3	1	2	3	1
1	1	1	2	1	1	1	1	1	1	1	1	3	3	1	1	1	1	1	1	2	1	3
1	1	1	1	1	1	1	1	1	4	2	4	4	4	1	1	1	1	4	4	4	3	1
3	3	3	3	3	3	3	3	2	2	2	2	2	4	2	3	3	2	2	2	3	2	1
1	1	1	3	1	1	1	1	1	1	3	1	1	3	1	1	1	1	2	2	3	1	1
3	3	3	3	3	3	3	3	3	3	3	3	3	3	3	3	3	3	3	3	3	3	3
4	4	4	4	4	4	4	4	4	4	4	4	4	4	4	4	4	4	4	4	4	4	4
2	1	4	1	3	1	1	1	1	2	2	2	3	1	1	2	2	1	2	3	3	2	2
1	1	4	1	1	1	1	1	1	1	2	1	1	1	1	1	1	1	1	1	1	1	1
1	1	1	1	3	1	1	1	1	1	1	1	1	1	1	1	1	1	1	1	1	1	1
1	3	6	6	5	1	1	2	3	4	4	3	4	3	1	1	1	1	1	1	1	1	1
4	1	1	1	3	1	1	1	1	2	2	1	3	2	1	1	1	1	1	1	1	1	1
1	1	4	1	1	1	1	1	1	2	1	1	3	2	1	1	1	1	1	2	1	1	1
4	2	5	6	7	2	3	2	3	1	2	3	4	3	1	1	1	1	3	3	3	2	1
5	1	3	1	2	1	1	1	1	2	3	3	3	3	1	2	5	1	2	4	3	2	1
5	5	5	6	5	5	5	5	5	3	3	3	3	3	3	3	3	3	3	3	3	3	3
2	1	2	1	2	1	1	1	1	2	1	2	1	1	1	3	1	1	1	1	1	3	1
1	1	1	1	1	1	1	1	1	1	1	1	1	2	1	1	1	1	1	1	3	1	1
1	1	4	1	1	1	1	1	1	1	1	1	3	1	1	1	1	1	1	1	1	1	1
1	1	1	1	1	1	1	1	1	3	4	4	3	1	1	1	1	1	2	2	2	1	1
1	1	3	1	1	1	1	1	1	1	1	1	1	1	1	1	1	1	1	1	1	1	1
1	1	1	1	1	1	1	1	1	2	2	2	1	1	1	3	3	2	2	2	1	2	1
2	2	2	2	2	2	2	2	2	2	2	2	3	3	2	2	2	2	2	2	2	2	2
3	4	3	3	5	4	4	4	3	4	4	4	3	3	3	1	1	1	3	3	3	1	1
1	1	3	1	1	1	1	1	1	2	2	2	3	3	2	1	2	1	1	2	2	2	1
3	2	6	3	6	3	2	3	2	1	1	1	1	1	1	1	1	1	1	1	1	1	1
3	1	1	1	3	1	1	1	1	2	2	1	3	2	1	1	1	1	2	1	2	1	1
4	1	6	2	4	1	1	1	1	4	4	4	4	4	1	2	2	2	2	1	4	3	1
1	1	5	1	1	1	1	1	1	3	4	4	5	3	1	1	1	1	1	1	1	1	1
5	1	1	7	7	1	1	1	1	5	5	4	4	5	1	1	1	1	1	1	1	1	1
3	1	5	1	5	1	1	1	1	5	4	3	4	4	4	3	3	4	1	1	1	3	1
2	1	7	1	1	1	1	1	1	1	1	3	1	3	1	1	2	3	3	1	3	1	1
2	1	4	2	1	1	1	2	1	2	2	2	2	2	3	3	3	1	1	1	1	4	1
4	1	5	4	6	2	1	1	1	2	4	4	1	1	1	3	4	1	1	1	1	3	1
1	1	6	4	4	1	1	1	1	4	2	4	5	5	1	1	1	1	1	1	1	1	1
3	2	1	3	3	2	1	1	2	2	4	3	2	2	2	2	3	1	2	2	2	2	1
3	3	2	3	2	3	2	2	3	2	2	2	2	2	2	2	2	2	2	2	2	3	2
2	1	1	1	2	1	1	1	1	2	2	2	2	1	1	1	1	1	1	1	1	1	1
1	1	1	1	1	1	1	1	1	1	1	1	3	3	1	3	3	1	1	1	1	5	1
3	1	2	2	1	1	1	1	1	1	1	1	4	2	1	1	1	1	1	1	1	1	1
1	1	4	1	6	1	1	1	1	4	3	4	1	2	1	1	1	1	3	2	3	1	1
3	1	5	1	3	1	1	2	1	3	3	4	4	4	1	1	1	1	1	3	2	3	1
3	1	6	1	2	1	1	1	1	3	3	2	2	2	1	1	1	1	2	1	1	2	1
2	1	2	4	4	1	1	1	1	1	2	2	2	2	1	1	1	1	1	1	1	1	1
5	1	1	2	4	2	1	1	1	2	2	3	4	4	1	2	1	1	3	3	3	4	4
1	1	3	1	1	1	1	1	1	1	1	1	1	2	1	1	1	1	2	1	2	1	1
5	5	5	4	4	4	4	4	4	3	3	4	4	4	3	3	3	3	3	3	3	3	3
2	2	2	2	2	2	2	2	2	2	2	2	2	2	2	2	2	2	2	2	2	2	2
1	1	1	1	2	1	1	1	1	2	2	2	2	2	1	2	2	1	1	1	1	2	1
2	1	2	1	2	1	1	1	2	2	3	2	2	2	1	1	1	1	1	1	2	1	1
1	1	3	1	1	1	1	1	1	1	1	1	1	3	1	2	1	1	1	1	1	2	1
1	1	1	1	1	1	1	1	1	1	1	1	1	1	1	1	1	1	1	1	1	1	1
1	1	6	1	1	1	1	1	1	1	1	2	4	4	3	3	4	3	3	2	1	5	1
2	1	1	1	1	1	1	1	1	1	2	2	2	3	1	3	2	1	1	1	1	2	1
4	1	5	4	2	1	1	1	1	4	4	3	3	3	1	3	3	2	3	3	3	3	1
3	1	2	2	2	2	1	1	1	4	3	3	4	2	1	4	4	2	3	3	3	2	1
5	3	4	5	5	2	1	1	3	4	3	4	4	1	1	1	1	1	3	2	3	3	1
2	1	6	1	4	1	1	1	1	2	3	1	2	2	1	1	1	1	1	1	1	1	1
1	1	3	1	1	1	1	1	1	2	3	3	4	4	1	2	1	1	1	3	3	2	1
1	1	1	1	1	1	1	1	1	1	1	1	1	3	1	1	1	1	1	1	1	1	1
6	1	5	3	1	1	1	1	1	3	3	3	4	3	1	4	4	3	1	1	1	3	1
1	1	3	1	5	1	1	1	1	4	4	5	3	2	1	5	3	1	1	1	1	5	1
2	1	2	1	5	1	1	1	1	3	4	3	3	3	2	1	1	1	3	3	3	1	1
7	7	7	7	7	7	7	7	7	5	5	5	5	5	5	5	5	5	5	5	5	5	5
1	1	3	1	1	1	1	1	1	1	1	1	1	2	1	1	1	1	1	1	1	1	1
5	1	4	1	1	1	1	1	1	5	5	5	5	5	3	5	5	1	5	5	5	5	1
1	1	3	1	1	1	1	1	1	5	5	5	5	3	1	4	5	2	3	3	3	4	1
4	1	5	1	7	1	1	1	1	2	2	1	3	4	1	1	1	1	1	1	1	1	1
1	1	1	1	1	1	1	1	1	1	1	1	1	1	1	1	1	1	1	1	1	1	1
2	1	3	2	1	1	1	1	1	2	1	2	3	1	1	1	1	1	1	1	1	1	1
1	1	1	2	1	1	1	1	1	2	2	2	3	2	1	1	1	1	1	1	1	1	1
2	1	2	2	1	1	1	1	1	1	1	1	2	2	1	1	1	1	1	1	1	1	1
4	2	5	1	5	1	1	4	2	4	3	5	4	4	3	3	4	2	3	3	3	3	1
1	1	1	1	1	1	1	1	1	1	1	1	2	1	1	1	1	1	1	1	1	2	1
2	2	2	2	2	2	2	2	2	2	2	2	2	2	2	3	2	2	2	2	2	2	2
1	1	4	4	4	1	1	1	1	3	3	3	3	3	3	3	3	3	3	3	3	3	3
3	1	1	1	1	1	1	1	1	1	1	3	1	1	1	2	2	1	1	1	1	2	1
2	1	1	1	1	1	1	1	1	1	1	1	2	2	1	1	1	1	1	1	1	1	1
1	2	6	5	6	1	1	5	1	1	1	1	1	1	1	1	1	1	1	1	1	1	1
1	1	1	1	1	1	1	1	1	1	1	1	3	2	1	1	1	1	1	1	1	1	1
4	1	5	1	4	1	1	1	1	3	4	1	1	1	1	3	3	2	2	2	2	1	1
1	1	2	1	4	1	1	1	1	3	3	4	3	4	1	5	4	5	4	4	3	2	1
4	1	1	6	1	1	1	1	1	2	2	2	4	4	1	1	1	1	1	1	1	2	1
1	1	3	1	1	1	1	1	1	1	1	1	1	1	1	1	1	1	1	1	1	1	1
1	1	4	1	1	1	1	1	1	2	2	2	4	3	1	1	1	1	1	1	1	2	1
6	4	5	4	4	2	2	2	2	3	3	3	4	4	2	1	2	1	1	1	1	2	1
3	4	6	1	1	2	1	3	1	1	1	1	2	2	1	3	3	1	1	1	1	3	2
6	6	6	5	5	6	6	5	6	4	4	5	4	4	2	5	5	5	4	4	4	5	1
1	1	1	2	3	1	1	1	1	1	1	1	1	1	1	1	1	1	1	1	1	1	1
6	1	3	1	1	1	1	1	1	3	3	3	4	4	1	1	1	2	1	3	1	2	1
4	4	4	4	4	4	4	4	4	1	4	1	1	4	1	1	1	1	1	1	1	3	1
4	1	1	1	4	1	1	1	1	1	2	4	4	3	1	1	1	1	3	3	2	5	1
1	1	1	1	3	1	1	1	1	1	2	1	5	4	1	1	1	1	1	1	1	1	1
1	1	2	1	2	1	1	1	1	1	1	2	4	2	1	1	1	1	2	2	1	1	1
1	1	1	1	1	1	1	1	1	4	2	5	5	1	1	5	2	1	2	2	2	5	1
3	1	1	1	2	1	1	1	1	3	3	4	3	3	1	2	2	3	1	1	1	2	1
2	1	7	1	5	3	2	3	1	4	5	4	3	1	1	1	1	1	2	2	3	4	1
1	1	1	1	1	1	1	1	1	2	1	2	3	3	1	1	1	1	1	1	1	1	1
1	1	7	1	2	1	1	1	1	2	2	2	3	3	1	1	1	1	1	3	1	1	1
1	1	1	1	7	1	1	1	1	5	5	5	3	1	1	2	2	3	1	1	5	1	1
7	5	5	7	1	1	1	1	1	5	5	5	5	1	4	1	1	1	1	1	1	1	3
3	1	6	1	2	1	1	1	1	4	3	3	3	3	1	1	1	1	3	3	3	2	1
3	3	3	3	3	3	3	3	3	1	2	1	3	2	1	1	1	1	1	1	1	1	1
1	1	6	1	3	1	1	1	1	1	1	1	2	1	1	1	1	1	2	1	1	1	1
1	1	1	1	1	1	1	1	1	2	2	3	3	2	1	1	2	2	1	2	2	2	1
4	4	4	4	4	4	4	4	4	2	2	2	2	2	1	2	2	1	1	1	1	2	1
2	1	7	1	1	1	1	1	1	4	4	4	5	5	1	3	5	3	1	1	1	4	1
1	1	6	1	1	1	1	1	1	2	2	2	4	2	3	1	3	1	1	1	1	2	1
3	1	1	1	1	1	1	1	1	1	1	1	1	1	1	1	1	1	1	1	1	1	1
1	1	1	1	1	1	1	1	1	1	1	1	3	3	1	1	1	1	1	1	1	1	1
2	2	2	1	3	1	1	2	2	3	3	4	3	4	2	1	1	1	3	3	2	1	1
1	1	1	1	1	1	1	1	1	1	1	1	1	1	1	1	1	1	1	1	1	1	1
1	1	5	2	2	1	1	1	1	1	1	2	2	2	1	3	4	3	2	3	3	4	1
1	1	1	4	7	1	1	1	1	1	1	2	1	1	1	1	1	1	3	4	4	1	1
2	1	1	1	2	1	1	1	1	1	1	1	1	1	1	1	1	1	1	1	1	1	1
2	2	1	2	2	2	2	2	2	2	2	3	4	3	1	1	1	2	2	2	2	2	2
3	1	1	2	2	1	1	1	1	2	2	2	2	2	1	3	4	1	1	1	2	1	1
6	4	7	2	5	4	4	5	4	3	3	4	4	4	3	1	1	1	1	2	2	2	2
2	2	2	2	2	2	2	2	2	2	2	2	2	2	2	2	2	2	2	2	2	2	2
1	1	2	5	4	1	1	1	1	2	5	5	2	2	1	4	3	2	3	1	3	2	1
1	1	1	1	1	1	1	1	1	1	1	1	3	3	1	1	1	1	1	1	1	2	1
3	1	7	3	2	1	1	1	1	2	2	3	4	4	1	5	3	5	1	1	1	5	1
1	1	1	3	1	1	1	1	1	3	1	1	1	3	1	1	1	4	5	1	4	1	1
5	1	2	2	1	1	1	2	1	4	3	3	2	1	1	1	1	1	2	2	2	1	1
5	5	3	4	5	3	3	4	2	4	4	4	3	4	3	1	1	1	3	3	3	1	1
6	6	6	1	4	1	1	1	3	3	3	4	4	4	5	1	1	1	2	2	2	5	1
2	1	1	1	1	1	1	1	1	2	2	2	4	3	1	1	1	1	3	3	3	1	1
3	5	4	3	5	4	5	4	5	5	5	5	1	5	3	1	1	1	5	5	5	5	1
3	1	2	1	1	1	1	1	1	2	3	3	4	4	1	2	2	1	1	1	1	3	1
4	6	4	6	4	5	6	5	2	4	4	4	1	1	1	1	1	1	3	4	3	1	1
2	1	2	1	1	1	1	1	1	1	2	2	3	2	1	1	1	1	2	2	2	1	1
5	1	5	1	5	1	1	1	1	5	5	5	5	5	1	5	5	3	1	1	1	5	1
1	1	1	1	1	1	1	2	1	1	1	1	1	1	1	1	1	1	1	1	1	1	1
7	4	4	1	3	1	1	1	1	1	3	5	1	1	1	2	2	1	1	1	1	3	1
1	1	6	3	7	1	1	1	1	4	4	4	4	4	1	5	1	4	1	4	3	5	4
1	1	7	1	1	1	1	1	1	1	1	1	1	1	1	1	1	1	1	1	1	1	1
7	3	6	2	2	2	2	3	4	3	2	4	5	5	2	1	1	1	4	4	4	4	1
7	1	3	1	1	1	1	1	1	5	5	5	4	4	3	5	5	5	2	3	2	4	1
3	1	7	1	2	1	1	1	1	1	1	1	3	3	1	1	1	1	1	1	1	1	1
1	1	4	2	2	1	1	1	1	1	1	2	2	2	1	1	1	1	2	2	2	1	1
1	1	1	1	1	1	1	1	1	1	1	2	4	1	1	2	2	2	1	1	1	3	1
1	1	1	2	2	1	1	1	1	1	4	3	4	3	1	4	4	2	1	1	5	5	1
1	1	1	6	3	1	1	1	1	1	1	1	1	1	1	2	1	2	1	1	1	2	1
1	1	2	1	1	1	1	1	1	1	2	2	2	1	1	1	1	1	1	1	1	1	1
6	1	7	2	5	1	1	6	4	5	4	4	3	5	4	5	5	3	3	3	3	4	1
3	2	2	1	3	1	1	2	1	1	2	2	3	2	1	1	1	1	3	3	3	2	1
4	4	4	4	4	4	4	4	4	4	3	3	3	1	3	2	2	2	2	2	2	2	2
2	1	1	1	2	1	1	1	1	2	2	2	3	1	1	2	2	1	1	1	2	2	1
3	2	4	1	2	1	1	1	1	3	3	3	3	2	1	1	1	1	4	3	2	3	1

3	5	6	7	5	5	5	5	4	5	3	3	3	4	5	2	4	3	4	3	3	3	4
1	1	1	1	1	1	1	1	1	1	1	1	1	1	1	1	1	1	1	1	1	1	1
2	1	1	2	2	1	1	1	1	2	2	3	4	4	2	4	4	3	1	1	1	4	1
2	1	1	1	1	1	1	1	1	1	1	2	3	1	1	2	1	1	1	1	1	1	1
2	2	2	3	2	4	3	2	1	3	2	2	3	3	2	4	4	3	2	2	2	4	1
6	4	2	4	4	4	4	4	4	4	4	4	2	2	1	4	3	2	3	2	3	3	3
4	1	6	5	6	1	1	1	1	1	4	3	2	4	1	1	1	1	1	1	1	4	1
5	3	5	6	7	3	3	3	6	5	5	5	3	2	2	1	3	1	5	4	5	3	1
1	1	1	7	5	1	1	1	1	1	1	1	3	2	1	4	3	3	3	1	2	3	1
3	1	3	5	7	1	1	1	1	3	4	5	3	3	4	3	1	3	3	3	3	3	3
4	4	4	4	4	4	4	4	4	3	3	3	3	3	3	3	3	3	3	3	3	3	3
2	1	1	1	5	1	1	1	1	4	3	4	1	2	1	4	4	1	1	1	4	5	1
1	1	6	1	1	1	1	1	1	4	2	3	4	4	1	2	2	2	1	1	1	3	1
1	1	1	1	7	1	1	1	1	3	3	3	4	4	1	2	1	2	3	1	3	2	1
4	3	4	3	3	3	3	3	3	3	4	4	5	5	3	3	3	2	2	3	3	4	2
1	1	1	1	1	1	1	1	1	1	1	1	1	1	1	1	1	1	1	1	1	3	1
2	1	1	1	1	1	1	1	1	1	1	1	1	1	1	1	1	1	1	1	1	1	1
6	2	3	2	1	1	1	1	1	2	4	3	4	3	1	1	2	1	1	1	1	2	1
1	1	2	3	1	1	1	2	1	3	3	2	3	5	3	3	3	4	3	1	1	2	1
4	4	4	4	4	4	4	4	4	3	3	3	3	3	3	3	3	3	3	3	3	3	3
2	7	5	6	5	7	5	6	5	2	2	2	3	3	3	3	4	4	3	4	3	4	4
4	4	4	4	4	3	3	3	3	4	4	4	3	3	1	3	3	2	2	2	3	4	1
1	1	1	1	1	1	1	1	1	2	3	1	1	1	1	1	1	1	1	1	1	1	1
1	1	3	1	1	1	1	1	1	1	1	1	1	1	1	1	1	1	1	1	1	1	1
3	2	3	2	3	2	3	2	3	2	2	2	1	3	1	1	1	1	1	1	1	1	1
1	1	1	1	1	1	1	1	1	1	1	1	2	2	1	1	1	1	1	1	1	1	1
3	1	6	2	5	1	1	1	1	4	4	1	4	3	1	5	4	4	3	3	3	5	1
7	1	1	2	1	1	1	1	1	1	2	1	2	2	1	2	2	2	4	2	4	1	1
5	4	5	2	2	2	1	3	1	1	1	1	1	1	1	1	1	1	1	1	1	1	1
1	1	3	1	1	1	1	1	1	2	4	1	3	2	1	1	1	1	1	1	3	1	1
2	1	6	2	3	1	1	1	1	2	3	3	2	2	1	1	1	1	1	1	1	1	1
2	1	4	1	6	1	1	1	2	4	5	4	2	4	2	1	1	1	1	1	1	3	1
6	6	3	5	5	6	5	4	6	4	5	3	4	4	3	3	4	4	3	3	3	4	4
3	2	2	1	2	1	1	1	1	3	3	4	3	1	1	1	1	5	4	3	3	2	1
5	1	5	1	1	1	1	1	1	1	1	1	1	1	1	1	1	1	1	1	1	1	1
6	6	6	6	6	6	6	6	6	4	3	4	5	5	4	4	4	4	4	5	5	5	4
1	1	1	1	1	1	1	1	1	1	1	1	4	1	1	1	1	1	1	1	1	1	1
2	1	4	1	1	1	1	1	1	1	1	1	1	1	1	1	1	1	1	1	1	1	1
1	1	1	1	1	1	1	1	1	1	1	1	1	2	3	1	1	1	1	2	1	3	1
1	1	1	1	1	1	1	1	1	2	3	2	2	1	1	1	2	1	1	1	1	1	1
3	3	3	3	3	3	3	3	3	5	4	4	4	3	4	4	4	4	4	4	4	4	4
1	1	6	1	2	1	1	1	1	2	2	1	2	2	1	2	1	1	3	1	1	3	1
7	7	7	7	7	7	7	7	7	5	5	5	5	5	5	5	5	5	5	5	5	5	5
2	1	5	5	4	1	1	1	1	2	2	2	3	2	1	1	1	1	2	2	2	1	1
4	3	6	1	1	1	1	1	1	3	3	3	3	2	2	2	2	2	2	2	2	2	2
6	4	7	5	6	4	3	3	3	4	4	4	3	4	2	1	2	1	1	1	1	2	1
1	1	2	1	2	1	1	1	1	3	3	2	1	1	1	1	1	1	1	1	1	1	1
1	4	2	2	1	1	1	2	1	2	2	2	4	4	1	1	1	1	1	1	1	1	2
2	1	1	1	6	3	1	2	1	5	5	5	2	3	3	1	1	1	3	3	3	3	1
2	1	1	1	1	1	1	1	1	2	3	3	3	2	1	1	1	1	1	1	1	1	1
3	3	3	3	3	3	3	3	3	1	1	2	1	1	1	1	1	1	1	1	4	1	1
2	1	1	1	1	1	1	1	1	2	2	3	1	3	4	1	1	1	1	2	1	2	1
6	1	6	1	6	1	1	1	1	5	5	5	3	3	3	4	4	4	4	4	4	4	1
7	5	7	1	3	1	1	1	1	5	5	5	5	5	5	1	5	1	5	5	5	3	1
1	1	1	1	1	1	1	1	1	1	1	1	3	2	1	1	1	1	1	1	1	1	1
3	2	2	1	2	3	2	2	2	2	2	1	3	2	2	3	4	2	2	2	2	2	1
1	1	1	1	2	1	1	1	1	2	1	2	3	1	1	1	1	1	1	3	1	1	1
1	1	1	1	4	1	1	1	1	2	2	1	5	3	1	1	1	1	1	3	3	2	1
2	1	2	1	1	1	1	1	1	1	1	2	3	3	1	1	1	1	2	3	1	1	1
3	2	4	2	2	1	1	1	1	3	4	4	2	2	1	4	4	2	2	2	3	3	1
1	1	1	1	1	1	1	1	1	1	1	3	2	4	2	1	1	1	1	1	1	2	1
1	1	1	1	1	1	1	1	1	1	1	1	1	3	1	1	1	1	1	1	1	1	1
2	2	2	2	2	2	2	2	2	1	1	1	2	2	1	1	1	1	1	1	1	1	1
6	1	6	2	5	1	1	1	1	4	4	4	3	4	2	4	4	2	3	3	3	4	1
1	1	1	1	1	1	1	1	1	1	1	1	1	1	1	3	1	1	1	1	1	1	1
7	7	7	7	7	7	7	7	7	5	5	5	5	5	5	5	5	5	5	5	5	5	5
3	3	3	3	3	3	3	3	3	3	3	3	4	3	3	3	3	3	3	3	3	3	3
3	3	3	3	3	3	1	1	1	2	2	2	3	2	1	1	1	1	1	1	1	1	1
1	1	5	1	3	1	1	1	1	2	2	1	1	1	1	1	1	1	2	1	1	2	1
5	6	6	4	5	5	4	4	6	2	4	4	4	4	3	5	4	5	4	4	3	4	4
5	1	1	1	1	1	1	1	1	1	1	2	3	3	1	1	1	1	1	1	1	1	1
2	1	1	1	1	1	1	1	1	2	2	2	1	1	1	1	3	4	3	3	3	4	1
2	1	1	1	2	1	1	1	1	1	2	2	2	1	1	3	3	1	1	1	1	3	1
2	1	1	1	1	1	1	1	1	1	1	3	3	2	1	1	1	1	1	1	1	1	1
2	1	2	1	3	1	1	1	1	2	2	2	2	2	1	2	1	1	1	1	1	1	1
2	1	4	5	1	1	1	1	1	1	2	2	2	3	2	1	2	1	1	1	1	2	2
4	1	3	1	6	2	1	1	1	2	5	4	5	5	5	1	1	1	1	1	1	1	1
4	1	4	1	5	1	1	1	1	1	1	3	1	3	1	1	1	1	1	1	1	4	1
1	1	4	1	1	1	1	2	1	1	1	1	3	2	1	1	1	1	1	1	1	2	1
4	2	3	2	3	1	1	1	1	2	2	2	4	3	1	2	1	2	1	1	1	3	1
4	2	2	2	4	1	1	1	1	3	3	3	3	3	1	3	3	1	3	3	3	3	1
6	1	1	1	1	1	1	1	1	1	2	1	5	5	1	4	4	1	3	3	1	4	1
3	1	4	1	5	1	1	1	1	5	5	5	5	5	1	1	1	1	1	1	2	1	1
5	1	6	1	1	1	1	1	1	1	1	1	5	5	1	5	5	5	3	3	3	5	5
2	1	4	2	4	1	1	1	1	3	2	3	2	1	1	1	1	2	2	3	1	2	1
4	1	1	1	1	1	1	1	1	1	3	3	1	1	1	1	1	1	1	1	1	1	1
1	1	1	1	1	1	1	1	1	4	4	4	3	3	2	2	2	1	1	1	1	2	1
1	1	5	1	1	1	1	1	1	1	1	1	1	1	1	3	3	3	1	1	3	1	1
1	1	1	4	3	4	3	1	1	4	4	4	3	3	1	1	1	1	3	3	3	1	1
2	1	2	1	2	1	1	1	1	3	2	2	3	3	1	1	2	2	2	2	2	2	2
3	2	2	2	1	1	1	2	2	1	1	1	2	2	1	1	1	1	2	2	3	1	1
4	1	3	2	1	1	1	1	1	1	1	2	3	4	1	1	1	1	3	3	3	1	1
3	1	5	1	4	2	2	2	2	1	1	1	1	1	1	1	1	1	1	1	1	1	1
3	1	1	1	7	1	1	1	1	4	2	1	4	1	1	5	5	2	1	1	1	3	1
5	1	4	1	1	1	1	1	1	1	1	1	1	1	1	1	1	1	1	1	1	1	1
3	1	7	1	1	1	1	1	1	1	1	1	3	2	1	1	3	2	1	1	1	3	1
1	1	1	1	1	1	1	1	1	1	1	1	2	1	1	3	1	1	1	1	1	2	1
1	1	1	1	1	1	1	1	1	1	1	1	1	1	1	1	1	1	1	1	1	1	1
1	1	7	1	1	1	1	1	1	1	2	4	4	4	1	1	1	1	1	1	1	5	1
1	1	6	1	1	1	1	1	1	1	2	2	3	3	1	2	3	2	1	1	1	2	1
1	1	2	1	1	1	1	1	1	4	4	5	5	2	4	5	4	2	2	2	2	5	2
1	1	6	1	2	1	1	1	1	1	1	1	3	2	1	1	1	1	1	1	1	1	1
4	3	3	3	3	3	3	3	3	1	1	1	1	1	1	1	1	1	1	1	1	1	1
1	1	5	1	1	1	1	1	1	1	4	3	4	3	1	3	2	1	1	1	1	3	1
6	2	4	1	1	1	1	1	1	3	3	3	2	2	3	3	3	2	2	2	2	3	2
1	1	1	1	1	1	1	1	1	2	2	1	2	1	1	1	1	1	4	1	3	1	1
1	1	1	1	1	1	1	1	1	1	1	1	3	1	1	1	1	1	1	1	1	1	1
3	1	1	1	1	1	1	1	1	1	2	1	1	1	1	1	1	1	1	1	1	1	1
6	1	1	1	1	1	1	1	1	1	1	1	3	1	1	1	1	1	5	5	5	1	1
1	1	1	5	1	1	1	1	1	1	1	1	4	4	1	4	4	1	1	1	1	4	1
4	2	6	2	7	1	1	2	2	5	5	2	2	3	1	1	1	1	3	3	4	2	1
2	1	1	1	1	1	1	1	1	1	1	2	5	4	1	1	1	1	3	3	1	1	1
1	1	1	1	1	1	1	1	1	1	1	1	4	3	1	1	1	1	1	1	1	1	1
4	1	4	6	7	1	1	1	1	4	4	4	4	3	1	1	1	1	1	1	1	1	1
4	2	7	1	7	2	2	2	1	5	5	4	5	3	2	1	1	1	1	1	1	3	1
5	5	6	6	5	6	5	6	7	4	4	3	3	1	1	1	1	1	1	1	1	4	1
4	2	2	2	2	3	2	1	1	3	3	4	3	2	1	1	1	1	5	5	5	1	1
3	3	3	3	3	3	3	3	3	1	1	1	1	1	1	1	1	1	1	1	1	1	1
1	1	1	7	1	1	1	1	1	1	1	2	2	1	1	1	1	1	1	1	1	2	1
5	1	7	7	7	1	1	1	1	3	3	4	4	4	3	1	1	1	1	1	1	2	1
3	1	1	1	3	1	1	1	1	1	1	1	1	1	1	3	3	1	1	1	1	3	1
1	1	7	1	1	1	1	1	1	3	3	3	1	1	1	1	1	1	1	1	1	3	1
4	1	5	3	5	1	1	1	1	2	3	4	4	5	2	1	2	3	2	2	2	2	1
4	4	7	7	7	1	1	1	1	5	5	5	3	1	1	4	1	1	1	5	3	3	1
4	1	3	1	1	1	1	1	1	1	1	1	1	1	1	2	1	1	1	1	1	2	1
1	1	4	1	1	1	1	1	1	1	1	1	1	1	2	2	1	2	1	1	1	2	1
3	4	4	3	3	4	4	3	3	3	4	3	3	4	3	2	3	3	3	3	4	4	4
1	1	1	1	1	1	1	1	1	2	1	1	3	3	1	4	4	1	3	3	3	4	3
7	4	7	6	5	1	1	1	1	4	5	5	1	5	3	4	3	4	3	3	3	3	3
2	1	1	1	1	1	1	1	1	1	1	1	1	1	1	1	1	1	1	1	1	3	1
3	3	3	3	3	3	3	3	3	3	3	3	3	3	3	3	3	3	3	3	3	3	3
4	4	4	4	4	4	4	4	4	3	3	3	3	3	3	3	3	3	3	3	3	3	3
4	3	6	2	6	3	6	3	1	4	5	4	3	2	1	3	4	2	3	3	4	4	1
2	3	2	1	2	3	1	3	2	2	2	2	3	4	1	2	2	1	1	1	2	3	1
4	4	4	4	4	3	5	4	4	3	4	4	3	2	2	2	3	3	3	4	4	4	4
2	2	4	1	1	1	1	1	1	4	4	4	4	2	2	1	1	1	1	3	4	3	1
4	2	3	1	2	2	2	2	2	5	5	5	3	3	1	3	4	2	3	5	5	3	1
1	1	1	1	1	1	1	1	1	1	1	1	1	1	1	1	1	1	1	1	1	1	1
3	2	6	2	6	2	2	2	2	5	5	4	4	4	2	4	5	4	2	3	2	4	4
1	1	3	1	4	1	1	1	1	4	5	5	5	2	1	1	3	2	3	2	5	3	1
1	7	7	7	7	7	7	7	7	1	5	5	5	5	3	1	1	1	5	5	5	5	1
4	4	4	4	4	4	4	4	4	2	2	2	4	3	3	1	1	1	3	3	3	4	1
7	7	7	7	7	7	7	7	7	5	5	5	5	5	5	5	5	5	5	5	5	5	5
1	1	1	1	4	1	1	1	1	5	5	5	3	5	5	1	1	2	1	3	3	1	1
4	4	6	1	4	4	1	2	1	1	3	3	3	3	1	1	1	1	3	3	3	1	1
1	1	1	1	1	1	1	1	1	1	2	3	3	3	1	1	1	1	1	1	1	1	1
1	1	1	1	1	1	1	1	1	3	1	3	2	1	1	2	1	2	5	5	5	2	1
4	4	5	3	4	3	2	4	3	4	4	4	4	4	1	1	1	1	3	3	4	3	2
2	2	2	2	2	2	2	2	2	3	3	2	3	3	1	1	2	2	1	1	1	3	1
2	1	2	2	2	2	2	2	2	1	1	1	2	2	1	1	1	1	1	1	1	1	1
2	2	2	2	2	2	2	2	2	4	4	4	1	1	1	1	1	1	1	1	1	1	1
2	2	2	2	2	2	2	2	2	2	2	2	4	4	2	2	2	2	2	2	2	2	2
1	1	1	1	1	1	1	1	1	1	1	1	5	5	1	1	1	1	1	1	1	1	1
6	1	7	6	7	1	1	1	2	4	5	5	5	5	3	5	5	4	1	3	2	5	1
6	3	7	5	6	4	3	3	3	4	5	4	5	4	2	5	5	3	2	2	3	5	1
2	2	2	2	2	2	2	2	2	2	1	1	3	4	1	1	1	1	1	1	1	1	1
4	1	1	1	4	2	1	1	1	1	1	1	1	1	1	1	1	1	1	1	1	2	1
5	1	1	1	1	1	1	1	1	4	3	3	4	4	1	1	3	1	1	1	1	1	1

Keram pada pe	SHE (Short Ter	Apakah Anda m	Apakah Anda s	Apakah Anda m	Apakah Anda m	Apakah Anda lu	Apakah Anda m	Apakah Anda m	Apakah Anda m	Skor AD8-INA (j
2	32	Tidak	Tidak	Tidak	Tidak	Tidak	Tidak	Tidak	Tidak	0
1	15	Tidak	Tidak	Tidak	Tidak	Tidak	Tidak	Tidak	Tidak	0
1	27	Tidak	Tidak	Tidak	Tidak	Tidak	Tidak	Tidak	Tidak	0
1	15	Tidak	Tidak	Tidak	Tidak	Tidak	Tidak	Tidak	Tidak	0
1	18	Tidak	Ya	Ya	Ya	Tidak	Tidak	Tidak	Ya	4
1	23	Tidak	Ya	Tidak	Tidak	Tidak	Tidak	Tidak	Tidak	1
1	23	Tidak	Ya	Tidak	Tidak	Tidak	Tidak	Tidak	Tidak	1
1	27	Tidak	Ya	Ya	Tidak	Tidak	Ya	Tidak	Tidak	3
1	18	Tidak	Tidak	Tidak	Tidak	Tidak	Tidak	Tidak	Tidak	0
3	40	Ya	Tidak	Ya	Tidak	Tidak	Tidak	Tidak	Ya	3
1	16	Tidak	Tidak	Tidak	Tidak	Tidak	Tidak	Tidak	Tidak	0
1	15	Tidak	Tidak	Tidak	Tidak	Tidak	Tidak	Tidak	Tidak	0
2	27	Ya	Ya	Ya	Tidak	Tidak	Ya	Ya	Ya	6
3	28	Ya	Tidak	Ya	Ya	Tidak	Tidak	Ya	Ya	5
4	49	Tidak	Tidak	Tidak	Tidak	Tidak	Tidak	Tidak	Tidak	0
2	30	Tidak	Tidak	Tidak	Tidak	Tidak	Tidak	Tidak	Tidak	0
5	65	Ya	Ya	Ya	Ya	Ya	Ya	Ya	Ya	8
1	25	Tidak	Tidak	Tidak	Tidak	Tidak	Tidak	Tidak	Tidak	0
3	52	Tidak	Ya	Tidak	Tidak	Tidak	Ya	Tidak	Ya	3
1	15	Tidak	Tidak	Tidak	Tidak	Tidak	Tidak	Tidak	Tidak	0
4	59	Ya	Ya	Tidak	Ya	Ya	Tidak	Ya	Tidak	5
1	17	Tidak	Tidak	Tidak	Tidak	Tidak	Tidak	Tidak	Tidak	0
1	21	Tidak	Tidak	Tidak	Tidak	Tidak	Tidak	Tidak	Tidak	0
1	19	Tidak	Tidak	Tidak	Tidak	Tidak	Tidak	Tidak	Tidak	0
1	33	Ya	Ya	Ya	Tidak	Ya	Ya	Tidak	Ya	6
1	15	Tidak	Tidak	Tidak	Tidak	Tidak	Tidak	Tidak	Tidak	0
5	75	Ya	Ya	Ya	Ya	Tidak	Ya	Ya	Ya	7
2	30	Tidak	Tidak	Ya	Tidak	Tidak	Tidak	Tidak	Ya	2
2	22	Tidak	Ya	Tidak	Tidak	Tidak	Tidak	Tidak	Tidak	1
1	25	Tidak	Tidak	Tidak	Tidak	Tidak	Tidak	Tidak	Tidak	0
1	15	Tidak	Tidak	Tidak	Tidak	Tidak	Tidak	Tidak	Tidak	0
4	47	Tidak	Ya	Ya	Tidak	Tidak	Ya	Tidak	Ya	4
5	43	Ya	Tidak	Ya	Tidak	Tidak	Ya	Tidak	Tidak	3
1	18	Tidak	Tidak	Tidak	Tidak	Tidak	Tidak	Tidak	Tidak	0
1	28	Ya	Ya	Ya	Tidak	Tidak	Tidak	Tidak	Ya	4
1	17	Tidak	Tidak	Tidak	Tidak	Tidak	Tidak	Tidak	Tidak	0
1	20	Tidak	Ya	Tidak	Tidak	Tidak	Tidak	Tidak	Tidak	1
1	28	Tidak	Ya	Ya	Tidak	Tidak	Tidak	Tidak	Tidak	2
1	17	Tidak	Tidak	Ya	Ya	Tidak	Tidak	Tidak	Tidak	2
1	39	Tidak	Ya	Tidak	Tidak	Tidak	Tidak	Tidak	Ya	2
1	15	Ya	Tidak	Ya	Tidak	Tidak	Ya	Ya	Ya	5
1	26	Ya	Tidak	Tidak	Tidak	Tidak	Tidak	Tidak	Tidak	1
1	41	Ya	Ya	Ya	Tidak	Ya	Tidak	Tidak	Tidak	4
1	22	Tidak	Tidak	Tidak	Tidak	Tidak	Tidak	Tidak	Tidak	0
2	40	Tidak	Tidak	Tidak	Tidak	Tidak	Tidak	Tidak	Tidak	0
2	34	Tidak	Tidak	Tidak	Tidak	Tidak	Tidak	Tidak	Tidak	0
1	23	Tidak	Tidak	Tidak	Ya	Tidak	Tidak	Tidak	Tidak	1
3	45	Ya	Ya	Tidak	Tidak	Tidak	Ya	Ya	Tidak	4
4	60	Ya	Ya	Ya	Ya	Ya	Ya	Ya	Ya	8
2	30	Tidak	Tidak	Tidak	Tidak	Tidak	Ya	Tidak	Tidak	1
1	16	Tidak	Ya	Tidak	Tidak	Tidak	Tidak	Tidak	Tidak	1
1	15	Tidak	Tidak	Tidak	Tidak	Tidak	Tidak	Tidak	Tidak	0
1	28	Ya	Tidak	Ya	Tidak	Tidak	Ya	Tidak	Ya	4
1	20	Tidak	Ya	Tidak	Tidak	Tidak	Tidak	Tidak	Tidak	1
1	20	Tidak	Tidak	Tidak	Tidak	Tidak	Tidak	Tidak	Tidak	0
1	30	Tidak	Ya	Ya	Tidak	Tidak	Tidak	Tidak	Tidak	2
1	36	Tidak	Tidak	Ya	Tidak	Tidak	Tidak	Ya	Ya	3
3	45	Ya	Tidak	Tidak	Ya	Tidak	Ya	Ya	Tidak	4
2	22	Ya	Tidak	Tidak	Tidak	Tidak	Tidak	Tidak	Ya	2
1	18	Tidak	Tidak	Tidak	Tidak	Tidak	Tidak	Tidak	Tidak	0
1	17	Tidak	Tidak	Tidak	Tidak	Tidak	Tidak	Tidak	Tidak	0
1	28	Ya	Ya	Ya	Tidak	Tidak	Tidak	Tidak	Tidak	3
1	15	Tidak	Tidak	Tidak	Ya	Tidak	Tidak	Tidak	Tidak	1
2	27	Tidak	Tidak	Tidak	Tidak	Tidak	Tidak	Tidak	Tidak	0
2	32	Tidak	Tidak	Tidak	Tidak	Tidak	Ya	Tidak	Tidak	1
1	36	Ya	Ya	Tidak	Tidak	Tidak	Ya	Tidak	Tidak	3
1	27	Tidak	Tidak	Tidak	Tidak	Tidak	Ya	Tidak	Ya	2
1	15	Tidak	Tidak	Tidak	Tidak	Tidak	Tidak	Tidak	Tidak	0
1	22	Tidak	Ya	Ya	Tidak	Tidak	Ya	Tidak	Tidak	3
1	39	Tidak	Tidak	Tidak	Tidak	Tidak	Tidak	Ya	Tidak	1
1	29	Tidak	Ya	Ya	Tidak	Tidak	Ya	Ya	Ya	5
1	33	Ya	Ya	Ya	Tidak	Tidak	Ya	Ya	Ya	6
4	45	Tidak	Tidak	Tidak	Tidak	Tidak	Tidak	Tidak	Tidak	0
1	26	Tidak	Ya	Tidak	Tidak	Tidak	Ya	Tidak	Tidak	2
4	32	Tidak	Tidak	Ya	Tidak	Tidak	Tidak	Tidak	Ya	2
3	31	Tidak	Ya	Ya	Tidak	Tidak	Ya	Ya	Tidak	4
1	30	Ya	Ya	Tidak	Tidak	Tidak	Ya	Tidak	Ya	4
1	31	Tidak	Tidak	Tidak	Tidak	Tidak	Tidak	Tidak	Tidak	0
2	31	Tidak	Tidak	Ya	Tidak	Tidak	Tidak	Tidak	Tidak	1
1	19	Tidak	Ya	Tidak	Tidak	Tidak	Tidak	Tidak	Ya	2
2	28	Tidak	Tidak	Tidak	Tidak	Tidak	Tidak	Tidak	Tidak	0
1	19	Tidak	Tidak	Ya	Tidak	Tidak	Tidak	Tidak	Ya	2
1	29	Tidak	Ya	Ya	Tidak	Tidak	Tidak	Tidak	Tidak	2
1	33	Tidak	Ya	Tidak	Tidak	Tidak	Tidak	Ya	Ya	3
1	24	Tidak	Tidak	Tidak	Tidak	Tidak	Ya	Ya	Tidak	2
1	19	Ya	Tidak	Tidak	Tidak	Tidak	Tidak	Ya	Tidak	2
3	40	Ya	Ya	Ya	Tidak	Tidak	Tidak	Tidak	Tidak	3
1	18	Tidak	Tidak	Tidak	Tidak	Tidak	Tidak	Tidak	Tidak	0
3	48	Ya	Ya	Ya	Tidak	Tidak	Ya	Tidak	Tidak	4
2	30	Tidak	Tidak	Tidak	Tidak	Tidak	Tidak	Tidak	Tidak	0
1	23	Tidak	Ya	Tidak	Tidak	Tidak	Tidak	Tidak	Tidak	1
1	22	Ya	Tidak	Ya	Tidak	Tidak	Tidak	Tidak	Tidak	2
2	20	Tidak	Tidak	Tidak	Tidak	Tidak	Tidak	Tidak	Tidak	0
1	15	Tidak	Tidak	Tidak	Tidak	Tidak	Tidak	Tidak	Tidak	0
2	39	Tidak	Ya	Ya	Tidak	Tidak	Tidak	Tidak	Tidak	2
1	24	Tidak	Ya	Ya	Tidak	Tidak	Ya	Tidak	Tidak	3
1	40	Tidak	Ya	Tidak	Tidak	Tidak	Tidak	Tidak	Ya	2
2	41	Tidak	Tidak	Tidak	Tidak	Tidak	Ya	Ya	Ya	3
3	35	Tidak	Ya	Ya	Tidak	Tidak	Ya	Tidak	Ya	4
2	21	Ya	Tidak	Ya	Tidak	Tidak	Tidak	Tidak	Ya	3
1	32	Ya	Ya	Ya	Tidak	Tidak	Tidak	Tidak	Ya	4
1	17	Tidak	Tidak	Tidak	Tidak	Tidak	Tidak	Tidak	Tidak	0
3	38	Ya	Tidak	Tidak	Tidak	Tidak	Tidak	Tidak	Tidak	1
5	42	Tidak	Ya	Ya	Tidak	Tidak	Tidak	Tidak	Ya	3
1	33	Tidak	Ya	Tidak	Tidak	Tidak	Ya	Tidak	Tidak	2
5	75	Ya	Ya	Ya	Tidak	Tidak	Tidak	Ya	Tidak	4
1	16	Tidak	Tidak	Tidak	Tidak	Tidak	Tidak	Tidak	Tidak	0
5	65	Ya	Ya	Ya	Tidak	Tidak	Ya	Tidak	Ya	5
1	50	Tidak	Ya	Ya	Ya	Tidak	Ya	Tidak	Ya	5
1	22	Tidak	Tidak	Tidak	Tidak	Tidak	Tidak	Tidak	Tidak	0
1	15	Tidak	Ya	Tidak	Tidak	Tidak	Ya	Tidak	Tidak	2
1	19	Tidak	Tidak	Tidak	Tidak	Tidak	Tidak	Tidak	Tidak	0
1	21	Tidak	Tidak	Tidak	Tidak	Tidak	Tidak	Tidak	Tidak	0
1	17	Tidak	Tidak	Tidak	Tidak	Tidak	Tidak	Tidak	Tidak	0
2	47	Tidak	Ya	Tidak	Tidak	Tidak	Tidak	Tidak	Ya	2
1	17	Tidak	Tidak	Tidak	Tidak	Tidak	Tidak	Tidak	Tidak	0
2	31	Tidak	Tidak	Tidak	Tidak	Tidak	Tidak	Tidak	Tidak	0
3	45	Tidak	Ya	Tidak	Tidak	Tidak	Tidak	Tidak	Tidak	1
1	20	Tidak	Tidak	Tidak	Tidak	Tidak	Tidak	Tidak	Tidak	0
1	17	Tidak	Tidak	Tidak	Tidak	Tidak	Tidak	Tidak	Tidak	0
1	15	Ya	Ya	Ya	Tidak	Tidak	Ya	Ya	Ya	6
1	18	Tidak	Tidak	Tidak	Tidak	Tidak	Tidak	Tidak	Tidak	0
1	28	Tidak	Ya	Tidak	Tidak	Tidak	Tidak	Tidak	Tidak	1
4	50	Tidak	Ya	Ya	Tidak	Tidak	Tidak	Tidak	Ya	3
2	26	Tidak	Tidak	Tidak	Tidak	Tidak	Ya	Tidak	Ya	2
1	15	Tidak	Tidak	Tidak	Tidak	Tidak	Tidak	Tidak	Tidak	0
3	26	Tidak	Ya	Tidak	Tidak	Tidak	Tidak	Tidak	Ya	2
1	30	Tidak	Ya	Tidak	Tidak	Tidak	Tidak	Tidak	Tidak	1
1	24	Tidak	Tidak	Tidak	Tidak	Tidak	Tidak	Tidak	Tidak	0
3	59	Tidak	Ya	Ya	Ya	Tidak	Tidak	Tidak	Ya	4
1	15	Ya	Tidak	Tidak	Tidak	Tidak	Tidak	Tidak	Ya	2
3	33	Tidak	Tidak	Tidak	Tidak	Tidak	Tidak	Tidak	Tidak	0
1	23	Tidak	Ya	Tidak	Tidak	Tidak	Tidak	Tidak	Tidak	1
3	35	Tidak	Ya	Tidak	Tidak	Tidak	Tidak	Tidak	Ya	2
1	23	Tidak	Tidak	Tidak	Tidak	Tidak	Tidak	Tidak	Tidak	0
1	22	Tidak	Tidak	Tidak	Tidak	Tidak	Tidak	Tidak	Tidak	0
5	43	Ya	Ya	Tidak	Tidak	Tidak	Tidak	Tidak	Tidak	2
1	31	Tidak	Ya	Ya	Tidak	Ya	Tidak	Tidak	Ya	4
1	34	Tidak	Ya	Ya	Tidak	Tidak	Tidak	Tidak	Ya	3
1	21	Tidak	Tidak	Tidak	Tidak	Tidak	Tidak	Tidak	Tidak	0
1	24	Tidak	Ya	Tidak	Ya	Tidak	Ya	Tidak	Tidak	3
2	38	Tidak	Tidak	Tidak	Tidak	Tidak	Tidak	Tidak	Tidak	0
1	36	Ya	Tidak	Ya	Tidak	Tidak	Ya	Tidak	Ya	4
1	33	Tidak	Ya	Tidak	Ya	Tidak	Tidak	Tidak	Tidak	2
1	19	Tidak	Tidak	Ya	Ya	Tidak	Ya	Tidak	Ya	4
1	17	Tidak	Ya	Ya	Tidak	Tidak	Tidak	Tidak	Tidak	2
1	27	Tidak	Tidak	Tidak	Tidak	Tidak	Tidak	Tidak	Tidak	0
1	23	Ya	Tidak	Tidak	Tidak	Tidak	Tidak	Tidak	Tidak	1
3	45	Ya	Tidak	Ya	Tidak	Tidak	Tidak	Tidak	Ya	3
1	27	Ya	Tidak	Tidak	Tidak	Tidak	Tidak	Tidak	Ya	2
1	15	Tidak	Tidak	Tidak	Tidak	Tidak	Tidak	Tidak	Tidak	0
1	19	Tidak	Tidak	Tidak	Tidak	Tidak	Tidak	Tidak	Tidak	0
1	33	Tidak	Tidak	Ya	Tidak	Tidak	Ya	Tidak	Tidak	2
1	15	Tidak	Tidak	Tidak	Tidak	Tidak	Tidak	Tidak	Tidak	0
1	33	Tidak	Tidak	Tidak	Tidak	Tidak	Tidak	Tidak	Tidak	0
1	24	Tidak	Ya	Ya	Tidak	Tidak	Tidak	Tidak	Tidak	2
1	15	Tidak	Tidak	Tidak	Tidak	Tidak	Tidak	Tidak	Tidak	0
2	31	Tidak	Tidak	Tidak	Tidak	Tidak	Tidak	Tidak	Tidak	0
1	26	Tidak	Ya	Tidak	Tidak	Tidak	Tidak	Tidak	Ya	2
2	35	Ya	Ya	Ya	Tidak	Tidak	Ya	Ya	Ya	6
2	30	Tidak	Ya	Tidak	Tidak	Tidak	Tidak	Tidak	Tidak	1
1	37	Ya	Tidak	Tidak	Tidak	Tidak	Tidak	Tidak	Ya	2
1	20	Tidak	Tidak	Tidak	Tidak	Tidak	Tidak	Tidak	Tidak	0
5	43	Tidak	Tidak	Tidak	Tidak	Tidak	Tidak	Tidak	Tidak	0
1	29	Tidak	Tidak	Tidak	Tidak	Tidak	Ya	Tidak	Tidak	1
1	26	Tidak	Tidak	Tidak	Ya	Tidak	Tidak	Tidak	Ya	2
1	37	Tidak	Ya	Ya	Tidak	Tidak	Tidak	Ya	Ya	4
1	39	Ya	Ya	Tidak	Tidak	Tidak	Tidak	Tidak	Ya	3
2	30	Tidak	Tidak	Tidak	Tidak	Tidak	Tidak	Tidak	Tidak	0
1	49	Ya	Ya	Ya	Tidak	Ya	Ya	Ya	Ya	7
3	32	Tidak	Tidak	Tidak	Tidak	Tidak	Tidak	Tidak	Tidak	0
1	31	Tidak	Ya	Tidak	Tidak	Tidak	Ya	Tidak	Ya	3
1	23	Tidak	Tidak	Tidak	Tidak	Tidak	Tidak	Tidak	Tidak	0
3	51	Ya	Ya	Ya	Tidak	Tidak	Ya	Tidak	Ya	5
1	15	Tidak	Tidak	Ya	Tidak	Tidak	Tidak	Tidak	Ya	2
1	25	Ya	Ya	Ya	Tidak	Tidak	Ya	Ya	Ya	6
5	53	Tidak	Ya	Tidak	Tidak	Tidak	Tidak	Tidak	Tidak	1
1	15	Tidak	Ya	Tidak	Tidak	Tidak	Tidak	Tidak	Tidak	1
4	45	Tidak	Ya	Tidak	Tidak	Tidak	Tidak	Tidak	Tidak	1
4	57	Ya	Ya	Ya	Tidak	Tidak	Ya	Ya	Ya	6
1	19	Tidak	Ya	Tidak	Tidak	Tidak	Ya	Tidak	Tidak	2
1	21	Tidak	Tidak	Tidak	Tidak	Tidak	Tidak	Tidak	Tidak	0
1	24	Tidak	Ya	Tidak	Tidak	Tidak	Tidak	Ya	Ya	3
5	44	Tidak	Ya	Ya	Tidak	Tidak	Ya	Tidak	Ya	4
2	19	Ya	Ya	Ya	Tidak	Tidak	Ya	Tidak	Ya	5
1	18	Tidak	Tidak	Tidak	Tidak	Tidak	Tidak	Tidak	Tidak	0
1	53	Ya	Ya	Ya	Tidak	Tidak	Tidak	Tidak	Ya	4
1	27	Tidak	Tidak	Ya	Tidak	Tidak	Tidak	Tidak	Tidak	1
2	35	Tidak	Tidak	Tidak	Tidak	Tidak	Tidak	Tidak	Tidak	0
1	24	Ya	Ya	Ya	Tidak	Tidak	Ya	Ya	Ya	6
1	32	Tidak	Tidak	Ya	Tidak	Tidak	Tidak	Tidak	Ya	2

2	51	Tidak	Ya	Tidak	Tidak	Tidak	Ya	Tidak	Tidak	2
1	15	Ya	Ya	Tidak	Tidak	Tidak	Tidak	Tidak	Ya	3
4	40	Ya	Tidak	Tidak	Tidak	Tidak	Tidak	Tidak	Tidak	1
1	19	Ya	Tidak	Tidak	Tidak	Tidak	Tidak	Tidak	Tidak	1
2	39	Tidak	Ya	Ya	Tidak	Tidak	Tidak	Tidak	Ya	3
3	43	Ya	Ya	Tidak	Tidak	Tidak	Ya	Tidak	Tidak	3
2	28	Ya	Ya	Tidak	Ya	Tidak	Tidak	Tidak	Ya	4
1	46	Ya	Ya	Tidak	Tidak	Tidak	Ya	Ya	Ya	5
1	30	Tidak	Tidak	Tidak	Tidak	Tidak	Ya	Tidak	Tidak	1
2	46	Ya	Ya	Ya	Tidak	Tidak	Ya	Tidak	Tidak	4
3	45	Tidak	Ya	Tidak	Tidak	Tidak	Tidak	Tidak	Tidak	1
3	39	Tidak	Tidak	Tidak	Tidak	Tidak	Tidak	Tidak	Tidak	0
1	32	Tidak	Tidak	Tidak	Tidak	Tidak	Tidak	Ya	Ya	2
1	34	Ya	Tidak	Ya	Tidak	Tidak	Tidak	Ya	Ya	4
2	48	Tidak	Ya	Ya	Tidak	Tidak	Tidak	Ya	Ya	4
1	17	Tidak	Ya	Tidak	Ya	Tidak	Tidak	Tidak	Tidak	2
1	15	Tidak	Tidak	Tidak	Tidak	Tidak	Tidak	Tidak	Tidak	0
2	29	Ya	Tidak	Ya	Tidak	Tidak	Ya	Tidak	Ya	4
2	39	Tidak	Tidak	Tidak	Tidak	Tidak	Ya	Tidak	Ya	2
3	45	Ya	Ya	Ya	Ya	Tidak	Tidak	Tidak	Tidak	4
4	48	Ya	Ya	Ya	Ya	Tidak	Tidak	Tidak	Tidak	4
3	42	Tidak	Ya	Ya	Tidak	Tidak	Ya	Ya	Ya	5
1	18	Tidak	Tidak	Tidak	Tidak	Tidak	Tidak	Tidak	Tidak	0
1	15	Tidak	Tidak	Tidak	Tidak	Tidak	Tidak	Tidak	Tidak	0
1	20	Tidak	Tidak	Tidak	Tidak	Tidak	Tidak	Tidak	Tidak	0
1	17	Tidak	Tidak	Tidak	Tidak	Tidak	Tidak	Tidak	Tidak	0
1	46	Tidak	Ya	Ya	Tidak	Tidak	Tidak	Tidak	Tidak	2
1	28	Tidak	Tidak	Tidak	Tidak	Tidak	Tidak	Tidak	Tidak	0
1	15	Tidak	Tidak	Ya	Tidak	Tidak	Tidak	Tidak	Tidak	1
1	24	Tidak	Ya	Ya	Tidak	Tidak	Tidak	Ya	Tidak	3
1	22	Ya	Tidak	Ya	Tidak	Tidak	Tidak	Tidak	Tidak	2
4	35	Ya	Tidak	Ya	Tidak	Tidak	Ya	Tidak	Tidak	3
4	55	Ya	Ya	Ya	Tidak	Tidak	Tidak	Tidak	Tidak	3
1	36	Tidak	Ya	Tidak	Tidak	Tidak	Tidak	Tidak	Tidak	1
1	15	Tidak	Tidak	Tidak	Tidak	Tidak	Tidak	Tidak	Tidak	0
4	64	Ya	Tidak	Ya	Ya	Ya	Ya	Ya	Ya	7
1	18	Tidak	Tidak	Tidak	Tidak	Tidak	Tidak	Tidak	Tidak	0
1	15	Ya	Ya	Tidak	Tidak	Tidak	Ya	Tidak	Tidak	3
1	21	Tidak	Tidak	Tidak	Tidak	Tidak	Tidak	Tidak	Tidak	0
2	22	Ya	Tidak	Tidak	Tidak	Tidak	Tidak	Tidak	Tidak	1
4	60	Ya	Ya	Ya	Ya	Tidak	Tidak	Tidak	Tidak	4
1	24	Tidak	Ya	Tidak	Tidak	Tidak	Tidak	Tidak	Tidak	1
5	75	Ya	Ya	Ya	Ya	Ya	Ya	Ya	Ya	8
1	24	Tidak	Tidak	Ya	Tidak	Tidak	Tidak	Tidak	Tidak	1
2	34	Ya	Tidak	Tidak	Tidak	Tidak	Ya	Ya	Tidak	3
1	32	Tidak	Ya	Ya	Tidak	Tidak	Tidak	Ya	Ya	4
1	20	Tidak	Tidak	Tidak	Tidak	Tidak	Ya	Tidak	Tidak	1
2	26	Tidak	Tidak	Tidak	Tidak	Tidak	Tidak	Tidak	Tidak	0
3	42	Ya	Ya	Ya	Tidak	Tidak	Tidak	Ya	Ya	5
1	23	Tidak	Tidak	Tidak	Tidak	Tidak	Tidak	Tidak	Tidak	0
2	20	Tidak	Ya	Ya	Tidak	Tidak	Tidak	Tidak	Tidak	2
1	26	Tidak	Tidak	Tidak	Tidak	Tidak	Tidak	Tidak	Tidak	0
1	54	Ya	Tidak	Ya	Tidak	Tidak	Ya	Tidak	Tidak	3
3	59	Ya	Ya	Ya	Tidak	Tidak	Ya	Tidak	Ya	5
1	18	Tidak	Ya	Tidak	Tidak	Tidak	Tidak	Tidak	Tidak	1
1	31	Tidak	Tidak	Ya	Tidak	Tidak	Tidak	Tidak	Tidak	1
1	21	Ya	Tidak	Tidak	Tidak	Tidak	Ya	Tidak	Tidak	2
1	28	Tidak	Ya	Tidak	Ya	Tidak	Ya	Tidak	Tidak	3
1	23	Tidak	Tidak	Tidak	Tidak	Tidak	Tidak	Tidak	Tidak	0
4	41	Tidak	Tidak	Tidak	Tidak	Tidak	Ya	Tidak	Tidak	1
1	23	Tidak	Tidak	Tidak	Tidak	Tidak	Tidak	Tidak	Tidak	0
1	17	Tidak	Tidak	Tidak	Tidak	Tidak	Tidak	Tidak	Tidak	0
1	17	Tidak	Tidak	Tidak	Tidak	Tidak	Tidak	Tidak	Tidak	0
1	46	Ya	Ya	Tidak	Tidak	Tidak	Tidak	Tidak	Tidak	2
1	17	Tidak	Tidak	Tidak	Tidak	Tidak	Tidak	Tidak	Tidak	0
5	75	Tidak	Tidak	Tidak	Tidak	Tidak	Tidak	Tidak	Tidak	0
3	46	Ya	Ya	Ya	Ya	Ya	Ya	Ya	Ya	8
1	21	Tidak	Ya	Tidak	Tidak	Tidak	Tidak	Tidak	Tidak	1
1	19	Tidak	Tidak	Tidak	Ya	Tidak	Tidak	Tidak	Tidak	1
4	58	Ya	Ya	Ya	Tidak	Ya	Ya	Ya	Ya	7
1	20	Ya	Tidak	Tidak	Tidak	Tidak	Tidak	Tidak	Ya	2
2	33	Tidak	Tidak	Tidak	Tidak	Tidak	Ya	Tidak	Ya	2
2	25	Tidak	Tidak	Tidak	Tidak	Tidak	Tidak	Tidak	Tidak	0
1	20	Tidak	Tidak	Tidak	Tidak	Tidak	Tidak	Tidak	Tidak	0
1	21	Tidak	Tidak	Ya	Tidak	Ya	Tidak	Tidak	Tidak	2
2	25	Tidak	Tidak	Tidak	Tidak	Tidak	Tidak	Tidak	Tidak	0
1	35	Ya	Ya	Ya	Ya	Ya	Ya	Ya	Ya	8
1	22	Tidak	Tidak	Tidak	Tidak	Tidak	Tidak	Tidak	Tidak	0
1	19	Tidak	Tidak	Tidak	Tidak	Tidak	Tidak	Tidak	Tidak	0
1	27	Tidak	Tidak	Tidak	Tidak	Tidak	Ya	Tidak	Tidak	1
1	37	Tidak	Ya	Tidak	Tidak	Tidak	Ya	Ya	Ya	4
4	40	Tidak	Tidak	Tidak	Tidak	Tidak	Tidak	Ya	Ya	2
1	36	Tidak	Tidak	Ya	Tidak	Tidak	Ya	Ya	Ya	4
5	53	Tidak	Ya	Tidak	Tidak	Tidak	Tidak	Tidak	Tidak	1
1	26	Tidak	Tidak	Tidak	Tidak	Tidak	Tidak	Tidak	Tidak	0
1	19	Ya	Ya	Tidak	Tidak	Ya	Tidak	Ya	Tidak	4
2	33	Ya	Ya	Tidak	Tidak	Tidak	Tidak	Tidak	Tidak	2
3	25	Tidak	Ya	Ya	Tidak	Tidak	Tidak	Tidak	Tidak	2
1	34	Ya	Tidak	Tidak	Tidak	Tidak	Ya	Tidak	Tidak	2
1	30	Tidak	Ya	Tidak	Tidak	Tidak	Tidak	Tidak	Ya	2
1	21	Tidak	Tidak	Tidak	Tidak	Tidak	Tidak	Tidak	Tidak	0
1	27	Tidak	Ya	Tidak	Tidak	Tidak	Tidak	Tidak	Tidak	1
1	15	Tidak	Tidak	Tidak	Tidak	Tidak	Tidak	Tidak	Ya	1
1		Tidak	Tidak	Tidak	Tidak	Tidak	Tidak	Tidak	Tidak	
1	15	Tidak	Tidak	Ya	Tidak	Tidak	Tidak	Tidak	Tidak	1
3	25	Tidak	Tidak	Tidak	Tidak	Tidak	Tidak	Tidak	Tidak	0
2	20	Tidak	Tidak	Tidak	Tidak	Tidak	Tidak	Tidak	Ya	1
1	15	Tidak	Tidak	Tidak	Tidak	Tidak	Tidak	Tidak	Tidak	0
4	32	Tidak	Tidak	Tidak	Tidak	Tidak	Tidak	Tidak	Ya	1
1	26	Tidak	Tidak	Tidak	Tidak	Tidak	Tidak	Tidak	Tidak	0
4	52	Tidak	Ya	Ya	Tidak	Tidak	Tidak	Ya	Ya	4
1	18	Tidak	Ya	Tidak	Tidak	Tidak	Tidak	Tidak	Tidak	1
1	15	Ya	Tidak	Ya	Ya	Tidak	Tidak	Tidak	Tidak	3
2	31	Tidak	Tidak	Tidak	Tidak	Tidak	Tidak	Tidak	Tidak	0
3	38	Ya	Tidak	Tidak	Tidak	Tidak	Ya	Tidak	Ya	3
1	23	Tidak	Ya	Tidak	Tidak	Tidak	Ya	Tidak	Tidak	2
1	17	Tidak	Tidak	Tidak	Tidak	Tidak	Tidak	Tidak	Tidak	0
1	16	Ya	Tidak	Ya	Ya	Ya	Ya	Ya	Ya	7
1	29	Tidak	Tidak	Tidak	Tidak	Tidak	Ya	Tidak	Tidak	1
1	30	Tidak	Ya	Tidak	Ya	Tidak	Tidak	Tidak	Tidak	2
3	37	Tidak	Ya	Tidak	Tidak	Tidak	Tidak	Tidak	Tidak	1
1	27	Ya	Tidak	Tidak	Ya	Tidak	Ya	Ya	Ya	5
1	20	Tidak	Tidak	Ya	Tidak	Tidak	Ya	Tidak	Tidak	2
1	29	Ya	Ya	Ya	Tidak	Tidak	Ya	Tidak	Tidak	4
3	37	Ya	Ya	Ya	Tidak	Ya	Ya	Ya	Ya	7
4	31	Tidak	Tidak	Tidak	Tidak	Tidak	Tidak	Tidak	Tidak	0
1	37	Tidak	Tidak	Ya	Tidak	Tidak	Ya	Tidak	Ya	3
1	15	Ya	Tidak	Ya	Tidak	Tidak	Ya	Ya	Ya	5
1	18	Tidak	Tidak	Ya	Tidak	Ya	Ya	Ya	Ya	5
1	31	Tidak	Tidak	Ya	Tidak	Tidak	Tidak	Ya	Ya	3
1	21	Tidak	Tidak	Tidak	Tidak	Tidak	Tidak	Tidak	Tidak	0
1	23	Tidak	Tidak	Tidak	Tidak	Tidak	Tidak	Tidak	Tidak	0
1	36	Tidak	Ya	Tidak	Tidak	Tidak	Tidak	Ya	Ya	3
1	40	Ya	Ya	Ya	Ya	Ya	Tidak	Ya	Ya	7
1	17	Tidak	Tidak	Tidak	Tidak	Ya	Tidak	Ya	Ya	3
1	19	Tidak	Tidak	Tidak	Tidak	Tidak	Tidak	Tidak	Tidak	0
4	50	Ya	Ya	Ya	Ya	Ya	Ya	Ya	Ya	8
4	40	Tidak	Tidak	Tidak	Tidak	Tidak	Tidak	Tidak	Tidak	0
3	52	Tidak	Tidak	Tidak	Tidak	Tidak	Ya	Tidak	Tidak	1
1	17	Tidak	Ya	Tidak	Tidak	Tidak	Tidak	Tidak	Tidak	1
3	45	Tidak	Tidak	Tidak	Tidak	Tidak	Tidak	Tidak	Tidak	0
3	45	Tidak	Tidak	Tidak	Tidak	Tidak	Tidak	Tidak	Tidak	0
4	47	Ya	Tidak	Ya	Tidak	Tidak	Ya	Ya	Ya	5
2	29	Tidak	Ya	Tidak	Tidak	Tidak	Tidak	Tidak	Tidak	1
4	49	Ya	Tidak	Tidak	Tidak	Tidak	Ya	Ya	Ya	4
1	36	Tidak	Tidak	Tidak	Tidak	Tidak	Tidak	Tidak	Tidak	0
3	51	Ya	Tidak	Ya	Tidak	Tidak	Ya	Tidak	Ya	4
1	15	Ya	Ya	Ya	Ya	Ya	Ya	Ya	Ya	8
5	57	Tidak	Tidak	Tidak	Tidak	Tidak	Ya	Tidak	Tidak	1
1	43	Ya	Tidak	Tidak	Tidak	Tidak	Ya	Tidak	Ya	3
5	53	Ya	Ya	Ya	Ya	Tidak	Tidak	Ya	Ya	6
3	36	Ya	Ya	Tidak	Tidak	Tidak	Ya	Tidak	Tidak	3
5	75	Tidak	Tidak	Tidak	Tidak	Tidak	Tidak	Tidak	Tidak	0
1	42	Ya	Ya	Ya	Tidak	Tidak	Tidak	Tidak	Ya	4
1	29	Tidak	Ya	Ya	Tidak	Ya	Ya	Ya	Ya	6
1	22	Tidak	Tidak	Tidak	Tidak	Tidak	Tidak	Tidak	Tidak	0
1	35	Tidak	Ya	Ya	Tidak	Ya	Tidak	Tidak	Ya	4
2	41	Ya	Ya	Ya	Tidak	Tidak	Ya	Ya	Ya	6
1	28	Tidak	Tidak	Ya	Tidak	Tidak	Ya	Tidak	Ya	3
1	17	Tidak	Tidak	Tidak	Tidak	Tidak	Tidak	Tidak	Tidak	0
1	24	Ya	Ya	Tidak	Ya	Tidak	Tidak	Tidak	Tidak	3
2	34	Tidak	Tidak	Tidak	Tidak	Tidak	Tidak	Tidak	Tidak	0
1	23	Tidak	Tidak	Tidak	Tidak	Tidak	Tidak	Tidak	Tidak	0
1	54	Tidak	Tidak	Ya	Tidak	Tidak	Ya	Tidak	Tidak	2
4	54	Tidak	Ya	Ya	Tidak	Tidak	Tidak	Ya	Ya	4
1	21	Tidak	Tidak	Tidak	Tidak	Tidak	Tidak	Tidak	Tidak	0
1	16	Ya	Ya	Ya	Tidak	Tidak	Tidak	Tidak	Tidak	3
1	30	Ya	Ya	Ya	Ya	Tidak	Tidak	Tidak	Tidak	4
