# Supplementary material for: Frequent Plastic Usage Behavior and Lack of Microplastic Awareness Correlates with Cognitive Decline: A Cross-Sectional Survey
Source: Int J Environ Res Public Health. 2026 Jan 1;23(1):67. doi: 10.3390/ijerph23010067 (PMC12841581; doi:10.3390/ijerph23010067)
Supplement: Supplementary file 1 [file ijerph-23-00067-s001.zip › supplementary tables.pdf]

Supplementary table S1

No	AD-8 Questions	n (%)
1	Do you have difficulty making decisions? For example: unable to give appropriate advice, unable to manage finances, giving inappropriate gifts, or trouble thinking?	Yes: 144 (25.62)<br>No: 418 (74.38)
2	Have you stopped engaging in hobbies/activities you previously enjoyed? For example: knitting, gardening, baking, reading, playing chess, music, or singing?	Yes: 216 (38.43)<br>No: 346 (61.57)
3	Do you repeat questions, stories, or statements?	Yes: 169 (30.07)<br>No: 393 (69.93)
4	Do you have difficulty learning how to use tools or appliances? Such as TV, radio, computer, microwave, remote control, stove, iron, or blender?	Yes: 51 (9.07)<br>No: 511 (90.93)
5	Do you forget the name of the month or the year?	Yes: 26 (4.63)<br>No: 536 (95.37)
6	Do you have difficulty handling finances? For example: paying utility bills, writing checks, paying taxes, or withdrawing pension money from the bank?	Yes: 134 (23.84)<br>No: 428 (76.16)
7	Do you have trouble remembering appointments or promises made to others?	Yes: 78 (13.88)<br>No: 484 (86.12)
8	Do you experience consistent memory/thinking problems in daily activities? For example: forgetting where you put your glasses, car keys, or other belongings?	Yes: 167 (29.72)<br>No: 395 (70.28)

Supplementary table S2

**Question**	**n (%) or median [IQR]**
Are you concerned about the negative impact of bottled drinking water in plastic packaging on the environment?	518 (92.17)
Yes (1)	16 (2.85)
No (-1)	28 (4.98)
I don't know (0)	
Are you concerned about the negative impact of bottled drinking water/food in plastic packaging on your health?	
Yes	520 (92.53)
No	20 (3.91)
I don't know (0)	20 (3.56)
How often do you manage the waste from plastic-packaged drinking water/food**?**	
Always (2)	72 (12.81)
Sometimes (1)	190 (33.81)
Rarely (0)	221 (39.32)
Never (-1)	79 (14.06)
What is your attitude after knowing that microplastics have been found in daily essential products?	
Stopped consuming products suspected to contain microplastics (1)	104 (18.51)
Reduced plastic usage (0)	421 (74.91)
Continue using/consuming the product (-1)	37 (6.58)

Supplementary table S3

**Question**	**n (%) or median [IQR]**
Microplastics may contain and accumulate hazardous chemicals (1–5)	5 (4–5)
Microplastics do not degrade in the environment (1–5)	5 (4–5)
Microplastics are harmful to human health through ingestion and inhalation (1–5)	5 (4–5)
Microplastics are harmful to animal health (1–5)	5 (4–5)
Microplastics cause economic harm (1–5)	4 (3–5)
